# Viral metagenomics revealed diverse CRESS-DNA virus genomes in faeces of forest musk deer

**DOI:** 10.1186/s12985-020-01332-y

**Published:** 2020-04-25

**Authors:** Qi Liu, Hao Wang, Yu Ling, Shi-Xing Yang, Xiao-Chun Wang, Rui Zhou, Yu-Qing Xiao, Xu Chen, Jie Yang, Wei-Guo Fu, Wen Zhang, Gui-Lan Qi

**Affiliations:** 1 Department of Microbiology, School of Medicine, Jiangsu University, 301 Xuefu Road, Zhenjiang, Jiangsu 212013 China; 2 Agricultural Engineering Research Institute, Jiangsu University, Zhenjiang, Jiangsu 212013 China; 3 Institute of Animal Husbandry, Chengdu Academy of Agriculture and Forestry Sciences, Chengdu, Sichuan 611130 China

**Keywords:** Forest musk deer, Faeces, Viral metagenomics, CRESS-DNA virus, Complete genome

## Abstract

**Background:**

Musk deer can produce musk which has high medicinal value and is closely related to human health. Viruses in forest musk deer both threaten the health of forest musk deer and human beings.

**Methods:**

Using viral metagenomics we investigated the virome in 85 faeces samples collected from forest musk deer.

**Results:**

In this article, eight novel CRESS-DNA viruses were characterized, whole genomes were 2148 nt–3852 nt in length. Phylogenetic analysis indicated that some viral genomes were part of four different groups of CRESS-DNA virus belonging in the unclassified CRESS-DNA virus, *Smacoviridae*, pCPa-like virus and pPAPh2-like virus. UJSL001 (MN621482), UJSL003 (MN621469) and UJSL017 (MN621476) fall into the branch of unclassified CRESS-DNA virus (CRESSV1–2), UJSL002 (MN621468), UJSL004 (MN621481) and UJSL007 (MN621470) belong to the cluster of *Smacoviridae*, UJSL005 (MN604398) showing close relationship with pCPa-like (pCRESS4–8) clusters and UJSL006 (MN621480) clustered into the branch of pPAPh2-like (pCRESS9) virus, respectively.

**Conclusion:**

The virome in faeces samples of forest musk deer from Chengdu, Sichuan province, China was revealed, which further characterized the diversity of viruses in forest musk deer intestinal tract.

## Introduction

Forest musk deer is a national protected animal, mainly distributed in Sichuan province, Guangxi province and other places, China [1, 2]. The death of forest musk deer occurs mainly in the young musk deer. Diseases were the most important factor in causing fawn death [3]. There have been studies on the diagnosis and prevention of some known diseases [4–7], but there is a lack of research on the unknown etiology.

Viruses with small circular rep-encoding ssDNA (CRESS-DNA) genomes encode a replication associated protein (Rep), mainly includes *Circoviridae* [8], *Genomoviridae.*

[9], *Smacoviridae* [10], *Geminiviridae*, *Nanoviridae* and *Bacilladnaviridae* [11]. These are widely found in various environments [12, 13], plant samples [14–17], dragonflies and damselflies [18–21], mosquitoes [22], rats [23], bats [24], duck [25], cattle [26], pigs [27, 28], dogs [29], human [30–33], and turkey [34]. CRESS-DNA genomes typically encode a replication initiator protein (Rep) and a capsid protein (Cap) [35].

In this study, the virus community in the intestinal tract of forest musk deer was analyzed by virus metagenomics. The results of this study put forward for the first time on CRESS-DNA viruses propagating among forest musk deer.

## Materials and methods

### Samples

In 2016, 85 forest musk deer faeces samples were collected from Chengdu, Sichuan province, China. Samples were collected by disposable materials and transported to the laboratory on dry-ice and store in the − 80 °C refrigerator. Samples were put into 1.5 ml tubes containing phosphate buffered saline (PBS). The supernatants of fecal samples were collected after vigorous eddy current for 5 min and centrifugation for 10 min (15,000 g) [36, 37].

### Viral metagenomic analysis

500 μl of supernatant was filtered through a 0.45 μm filter (Millipore) to remove eukaryotic and bacterial cell sized particles. The viral particle enrichment filtrate was then treated with uncleases to digest nonparticle protected nucleic acid at 37 °C for 90 min [38]. Remaining total nucleic acid, protected from digestion with in viral capsids, were then extracted using the QiaAmp Mini Viral RNA kit (Qianen) according to manufacturer’s protocol [37, 39, 40]. Eight separate pools of nucleic acids from 85 faecal specimens were generated randomly, of which six contained ten faecal apecimens, the other one contained 12 faecal specimens and another one contained 13 faecal specimens. These eight viral nucleic acid pools, containing both DNA and RNA viral sequences, were then subjected to RT reactions with SuperScript III reverse transcriptase (Invitrogen) and 100 pmol of a random hexamer primer, followed by a single round of DNA synthesis using Klenow fragment polymerase [37, 41]. Eight libraries were constructed using Nextera XT DNA Sample Preparation Kit (Illumina) and sequenced using the MiSeq Illumina platform with 250 bases paired ends with dual barcoding for each library. The data is processed using an internal analysis pipeline running on a 32-nodes Linux cluster. Clonal reads were removed, and low quality sequence tails were trimmed with Phred quality score ten as the threshold. The adapter is trimmed using of VecScreen’s default parameters, NCBI BLASTn, with specialized parameters designed for adapter removal [42]. After deleting repeated reads and reads less than 50 in length followed by de novo assembly [43]. The contigs and singlets were matched against an internal viral proteome database using BLASTx with an E-value cutoff of < 10–5. BLASTx were used to identify viral sequences in order to annotated viral proteins available in GenBank’s viral RefSeq database [44].

### Genome acquisition and PCR screening

**Fig. 1:**
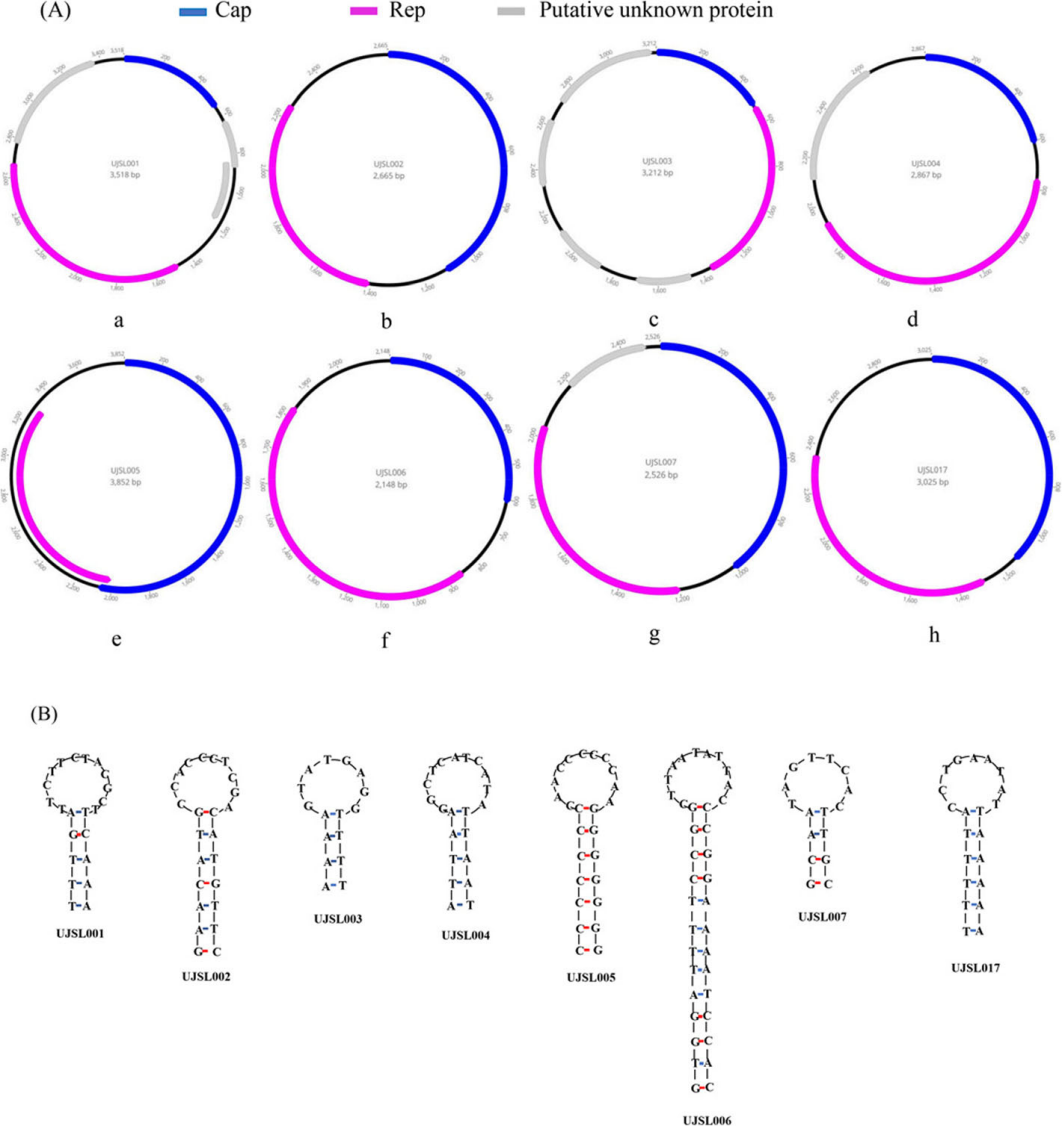
The whole genome sequence structure and the stem-loop structures of the eight CRESS-DNA viruses isolated from forest musk deer. **a**. The genomic organization of the eight CRESS-DNA viruses identified in the forest musk deer faeces samples. **b**. The stem-loop structures of the eight CRESS-DNA viruses isolated from forest musk deer

Putative open reading frames (ORFs) in the circular genomes were predicted by Geneious software version 2019.0.3 [45], and the stem-loop in the circular genomes were located through the The Mfold [24] (Table 1 and Fig. 1b). If the whole genome sequence of the virus was not obtained through sequence reads analysis, inverse PCR was needed. Two whole genomes of UJSL004 and UJSL005 were acquired by screen PCR and inverse PCR. Primers are shown in an additional file (see Additional file 1). The PCR conditions in screen PCR were: 95 °C for 5 min, 31 cycles 95 °C for 30 s, 50 °C (for the first round) or 57 °C (for the second round) for 30 s and 72 °C for 40 s, a final extension at 72 °C for 5 min, resulting in an expected amplicon of 300 bp–500 bp. The PCR conditions in inverse PCR of UJSL004 were: 95 °C for 5 min, 35 cycles 95 °C for 30 s, 50 °C (for the first round) or 55 °C (for the second round) for 30 s and 72 °C for 1.5 min, a final extension at 72 °C for 5 min, resulting in an expected amplicon of 1000 bp. The PCR conditions in inverse PCR of UJSL005 were: 95 °C for 5 min, 35 cycles 95 °C for 30 s, 50 °C (for the first round) or 51 °C (for the second round) for 30 s and 72 °C for 1.5 min, a final extension at 72 °C for 5 min, resulting in an expected amplicon of 1000 bp.

**Table 1:** Loop sequences of these CRESS-DNA virus CRESS-DNA, small circular rep-encoding ssDNA

strain name	Loop sequence	sequence length
UJSL001	ATTCTTCTACGCTT	14
UJSL002	GCCACCCTCGAC	12
UJSL003	AGTATGAGGT	10
UJSL004	AGGCTCATCATAT	13
UJSL005	CCAACCCCCCAAG	13
UJSL006	GCTTAATATTACC	13
UJSL007	ATAGTTCACT	10
UJSL017	ACCTGAATATT	11

### Phylogenetic analysis

The Rep protein sequences of these novel virus were homology alignment with the reference sequences in GenBank using the ClustalW program in MEGA7.0. Phylogenetic analyses were constructed using full-length rep protein of novel virus and other genetically close relatives [22, 46]. Save the aligned sequence as a Nexus form file, which was used to construct the phylogenetic tree using Bayes’ theorem in Mrbayes3.2.7 program. Using mixed models and Markov chain Monte Carlo (MCMC) methods. In phylogenetic analyses, tree samples are typically most divergent, so we introduced the average standard deviation of split frequencies (ASDSF) in MrBayes to allow quantitative evaluation of similarity among these samples. MrBayes allow users to set cut-off frequency (default value 0.10, [47–49]). We used the “sump” and “sumt” commands to get more detailed diagnostic information after the run has completed.

## Results

The 85 faeces samples of the eight libraries generated a total of 6, 153, 736 unique sequence reads using illumine Miseq sequencing runs with 250 base pair terminals. The Ensemble program was used to read the de novo assembly sequence [43] and BLASTx was used to compare it with Genbank’s non-redundant protein database. The results indicated that CRESS-DNA virus accounted for the main part of the total mammalian virus readings, with 4, 775 reads showing sequence similarity to the CRESS-DNA virus, 462 reads related to viruses from *Smacoviridae* and 473 reads sequence similar to the virus of *Circoviridae*. Table 2 list the detailed information.

**Table 2:** Characterization of the viral sequence reads in forest musk deer faeces samples. *nt* nucleotides, *aa* amino acids

Library ID (Total unique reads)	Family	Genus	Strain name	Accession number	No. of nt in the genome	No. of aa in protein		GC-content(%)	GenBank no. of the matches	Aa identities with the match(s)	Max_Contig	Total reads
						Rep (Direction)	Cap (Direction)					
L1(1136670)	Circoviridae	Circovirus	UJSL017	MN621476	3025	355(−)	380(+)	42.6	KU043411.1	62.54%	2497	1123
L2(991928)	None	None	UJSL001	MN621482	3518	399(−)	190(+)	41	KY487934.1	48.76%	3448	583
L3(307679)	Circoviridae	None	UJSL002	MN621468	2665	283(−)	372(+)	50.8	NC_030125.1	69.09%	3919	205
	None	None	UJSL003	MN621469	3212	281(−)	173(+)	46.5	MH617688.1	44.00%	3330	206
L5(777594)	Circoviridae	None	UJSL004	MN621481	2866	390(−)	204(+)	40.6	NC_039054.1	75.39%	2053	41
L6(530538)	Circoviridae	None	UJSL005	MN604398	3852	428(−)	692(+)	42.4	NC_026635.1	32.63%	3155	473
L7(1237606)	None	None	UJSL006	MN621480	2148	327(+)	203(+)	32.4	MK858258.1	58.86%	2253	2863
L8(802429)												
L9(369292)	None	None	UJSL007	MN621470	2526	280(−)	337(+)	45.6	MH500284.1/MH500317.1	95.54%	2672	216
Total(6153736)												

### CRESS-DNA genomes

Four complete CRESS-DNA genomes showing the highest sequence identity to CRESS-DNA virus. Genomes were 3518 nt (UJSL001, from library 2), 3212 nt (UJSL003, from library 3), 2148 nt (UJSL006, from library 7) and 3025 nt (UJSL017, from library 1) in length. As shown in Fig. 1a, the genomes of UJSL001, UJSL003 and UJSL017 contained two bidirectional ORFs while UJSL006 is in the same direction, encoding the putative Rep and Cap proteins. BLASTp search in GenBank based on the protein sequence of Rep showed UJSL001 shared the highest identity of 48.76% to unclassified circular virus (KY487934.1), UJSL003 shared the highest sequence identity of 44.00% to unclassified ssDNA viruses (MH617688.1), UJSL006 shared the highest sequence identity of 58.86% to an unclassified circular DNA viruses (MK858258.1) and UJSL017 shared the highest identity of 62.54% to unclassified ssDNA viruses (KU043411.1) (Table 2).

Three complete CRESS-DNA genomes showing the highest identity to Smacovirus. Genomes were 2665 nt (UJSL002, from library 3), 2866 nt (UJSL004, from library 5) were obtained through inverse PCR, and 2526 nt (UJSL007, from library 9) in length, respectively. Figure 1a manifested the genomic organization of UJSL002, UJSL004 and UJSL007, where the predicted Rep and Cap of the three viruses are differently arranged. BLASTp search in GenBank based on the protein sequence of Rep showed UJSL002 shared the highest sequence identity of 69.09% to a Bovine faeces associated smacovirus5 (NC_030125.1), UJSL004 shared the highest sequence identity of 75.39% to a Bovismacovirus (NC_039054.1) and UJSL007 shared the highest sequence identity of 95.54% to two Porprismacovirus (MH500284.1 and MH500317.1) (Table 2).

A complete CRESS-DNA genome showing the highest sequence identity to *Circoviridae*. Genome was 3852 nt (UJSL005, from library 6) in length. UJSL005 genome was acquired through inverse PCR based on a large contigs from library 6 and Sanger sequencing. Figure 1a indicated the genomic organization of UJSL005, where the predicted Rep and Cap of the UJSL005 in the opposite direction. BLASTp search in GenBank based on the protein sequence of Rep showed UJSL005 shared the highest sequence identity of 32.63% to unclassified *Circoviridae* (NC_026635.1) (Table 2).

**Fig. 2:**
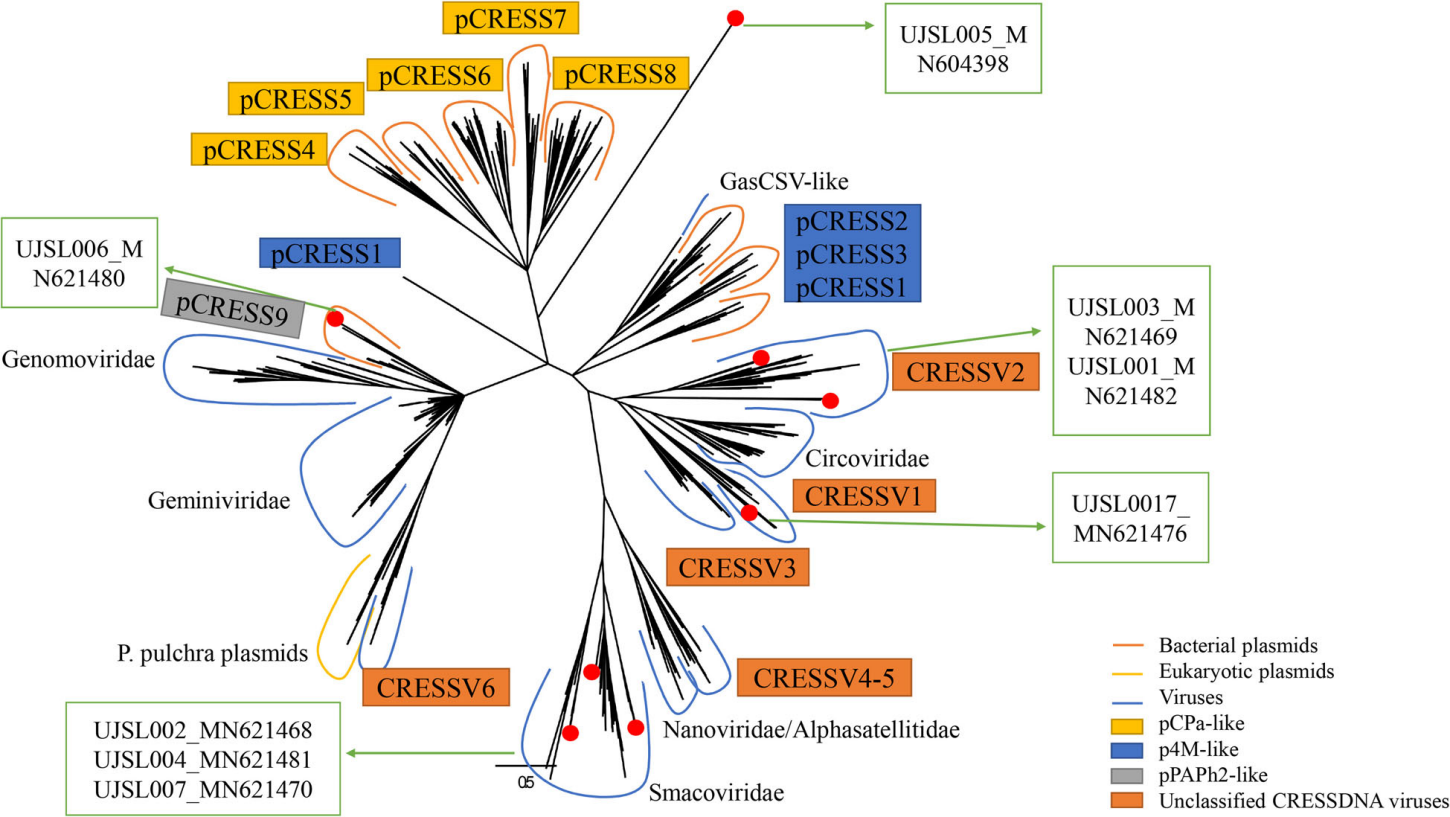
Phylogenetic analysis and genomic organization of the novel CRESS-DNA virus identified in forest musk deer. Phylogenetic analysis was performed based on the amino acid sequence of Rep protein. A total of 672 alignment sequences were included 8 CRESS-DNA virus identified here, their best BLASTp matches in GenBank based on the Rep proteins, and the representative of all classified families of CRESS-DNA virus as well as 6 groups of unclassified CRESS-DNA virus (CRESSV1–6), two GasCSV-like viruses, Bacterial plasmids (pCRESS1–9) and a small group of Eukaryotic plasmids (*P. pulchra* plasmids). All clads are shown with curves and the names are shown beside the corresponding clades. Viruses identified in this study were labeled with red colored dots and the virus names and sequence accession numbers are shown in green arrow and text box beside their corresponding

Based on the alignment of the Rep amino acid sequences herein detected with the best matches of BLASTp search in GenBank and those of representative CRESS-DNA genomes including 6 groups of unclassified CRESS-DNA virus (CRESSV1–6), two GasCSV-like viruses, Bacterial plasmids (pCRESS1–9) and a small group of Eukaryotic plasmids (*P. pulchra* plasmids) from GenBank, a phylogenetic tree was constructed [50–52]. For phylogenetic analyses, we used a dataset with 672 sequences of the Rep amino acid (Fig. 2) (Additional file 2).

**Fig. 3:**
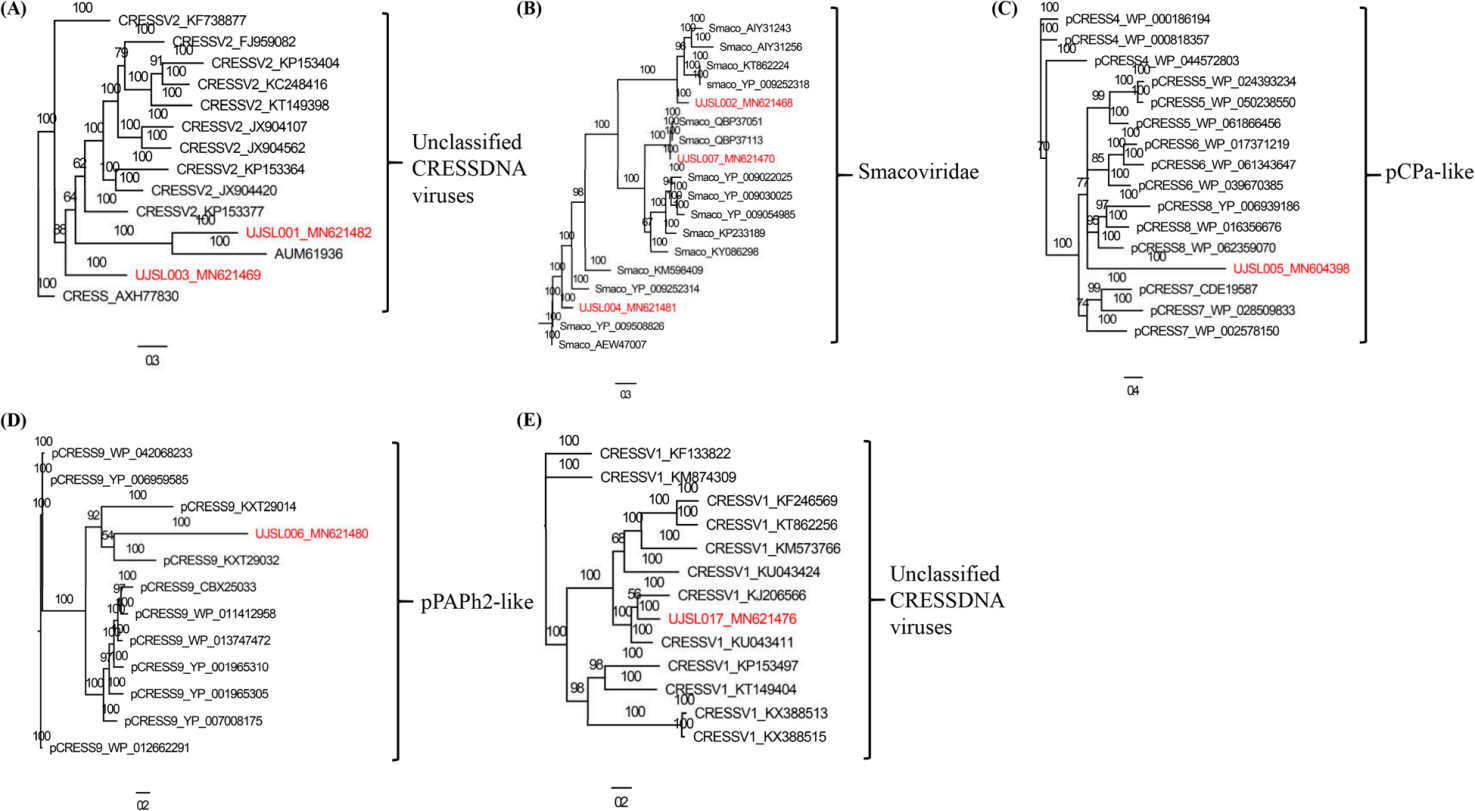
Phylogenetic analysis of novel virus detected from forest musk deer in different clads. The accession clusters name is shown on the right side of the tree and the novel virus name are marked with red color. **a**. Phylogenetic analysis were based on the Rep protein amino acid sequence of UJSL001, UJSL003 and 12 similar viral amino acid sequences in the clads of unclassified CRESS-DNA virus (CRESSV2). **b**. Phylogenetic trees were constructed with the Rep protein amino acid sequence of UJSL002, UJSL004, UJSL007 and 15 similar viral amino acid sequences in the clads of *Smacoviridae*. **c**. Phylogenetic trees were constructed used the Rep protein amino acid sequence of UJSL005 and 15 the similar viral amino acid sequences into pCPa-like (Pcress4–8) clusters. **d**. Phylogenetic trees were constructed with UJSL006 and 11 similar viral amino acid sequences into pPAPh2-like (Pcress9) clusters. **e**. Phylogenetic were constructed by Rep protein amino acid sequence of UJSL017 and 12 viruses amino acid sequences that are relatively close together in the clads of unclassified CRESS-DNA virus (CRESSV1)

UJSL001, UJSL003 and UJSL017 fall into the branch of unclassified CRESS-DNA virus (CRESSV1–2), UJSL001 and UJSL003 belong to the cluster of CRESSV2, UJSL001 showing close relationship with CRESS_AUM21936, UJSL003 showing close relationship with CRESS_AXH77830 (Fig. 3a) (see Additional file 3) and UJSL017 belong to the cluster of CRESSV1, showing close relationship with CRESSV1_KJ206566 and CRESSV1_KU043411 (Fig. 3e) (see Additional file 7). UJSL002, UJSL004 and UJSL007 belong to the cluster of *Smacoviridae* (Fig. 3b) (see Additional file 4), UJSL005 fall into the branch showing close relationship with pCPa-like (pCRESS4–8) clusters (Fig. 3c) (see Additional file 5) and UJSL006 fall into pPAPh2-like (pCRESS9) clusters, showing close relationship with pCRESS9_KXT29032 (Fig. 3d) (see Additional file 6).

### Nucleotide sequence accession numbers

The viral genomes described in detail here were deposited in GenBank under the following accession numbers: MN604398, MN621468- MN621470, MN621480- MN621482 and MN621476.

## Discussion

Our report describes viral nucleic acids enriched in forest musk deer faeces, shows that CRESS-DNA virus sequences are present in all libraries and have the most reads compared to other viruses. This suggests that these viruses are likely to replicate in forest musk deer host cells, but there is no evidence for this. Based on phylogenetic analysis, four different groups of CRESS-DNA genomes in forest musk deer faeces were detected, which belonged to CRESS-DNA virus, *Smacoviridae*, pCPa-like virus (pCRESS4–8) and pPAPh2-like (pCRESS9). For the first time, CRESS-DNA virus in the faeces of forest musk deer was mentioned, which was beneficial to further understanding of the genetic and evolutionary diversity of these viruses.

CRESS-DNA viruses with small, circular replication-associated protein (Rep)-encoding single stranded (CRESS) DNA genomes, are largely identified based on conserved rolling circle replication proteins [11]. It consists of a large group of highly specific viruses that can infect many types of host [53]. These virus included: *Circoviridae* [39], which can infect vertebrates, *Geminiviridae* [14] and *Nanoviridae* [54] which can infect plants. The genomes of *Circoviridae* range in size from 1.7 to 2.1 kb and contain two major ORFs, which encode Rep and Cap proteins. According to the International Committee on Taxonomy of Viruses (ICTV), the ssDNA has genomes between 1.7-6 kb. Eight CRESS-DNA virus extracted in this study, the genomes range in size from 2.1 kb to 3.5 kb. Previous research on the stem-loop structure of diverse circovirus and cycloviruses, a highly conserved stem-loop structure is also found [31, 52, 55], because they study multiple viruses of the same genus. Eight viruses in our study located in different genera based on rep protein phylogenetic analysis, so the stem-loop structure sequences are different from each other.

In the recent years, a large number of CRESS-DNA genomes have been determined in human and any other mammals, birds, insects, plants, fungi, and environment samples which bringing to light a high level of genetic diversity among these virus [25, 26, 31, 33, 52, 56]. Although use metagenomics to identify these viruses from forest musk deer faeces, we cannot rule out that they may also represent food contaminants and environmental pollution [57]. These viruses exploit host polymerases for DNA synthesis and code for proteins that modulate the host’s cell cycle favourably for virus multiplication [58]. There are reports that the virus is associated with disease, but it has not been proven to cause the disease directly [59, 60]. And the effects and disease correlation of these viruses on the health of forest musk deer need further study.

In conclusion, this study is the first to discover a variety of new CRESS-DNA viruses in the intestinal tract of forest musk deer and analyze their genomic characteristics, which is of great significance for the study of forest musk deer virus and the genetic and evolutionary diversity of CRESS-DNA virus. At the same time, the host adaptability and pathogenicity of the new CRESS-DNA virus need further study.

## Conclusions

The virome in faeces samples of forest musk deer from Chengdu included the viruses showing sequence similarity to CRESS-DNA viruses, where eight divergent genomes of CRESS-DNA viruses were identified in detail. The contents include genome protein structure, stem-loop structure and rep protein phylogenetic analysis. Although CRESS-DNA virus is prevalent in forest musk deer, its pathogenicity has not been known. This study increased the knowledge of the diversity of viruses in forest musk deer faeces.

## Supplementary information

**Additional file 1.**

**Additional file 2.**

**Additional file 3.**

**Additional file 4.**

**Additional file 5.**

**Additional file 6.**

**Additional file 7.**

## Data Availability

The viral genomes described in detail here were deposited in GenBank under the following accession numbers: MN604398, MN621468- MN621470, MN621480- MN621482 and MN621476.

## Abbreviations

BLAST: Basic local alignment search tool
CRESS: Circular rep-encoding single stranded
GasCSV: Gastropod-associated circular ssDNA virus
NCBI: National Center for Biotechnology Information
ORF: Open reading frame
pCPa: Circular plasmids from plant-pathogenic
PCR: Polymerase chain reaction
pPAPh2: Plasmids Plant Pathogens of Phytoplasma
*P. pulchra* plasmids: Pyropia pulchra plasmids
