# Supplementary material for: Viral metagenomics revealed diverse CRESS-DNA virus genomes in faeces of forest musk deer
Source: Virol J. 2020 Apr 25;17:61. doi: 10.1186/s12985-020-01332-y (PMC7183601; doi:10.1186/s12985-020-01332-y)
Supplement: Supplementary file 1 — Additional file 1. [file 12985_2020_1332_MOESM1_ESM.pdf]

supplementary table 1. Primers of Screen PCR

Species	UJSL004	UJSL005
F1	GATTGTATCGGGGCTAAGTCT	TACTTAAACAGCCTGGTTACGA
R1	CATCTTAGAAGCCAGGGGAAT	AGGCTATCATTTCATCGAGGT
F2	GGGGAGCCTAGTATTACCTTG	CCATCCTCTGCCTATATGTGC
R2	AACGGCTATATTCATGTTCGC	CGTTATTAGGTACAGGGGAGG

supplementary table 2. Primers of Inverse PCR

Species	UJSL004	UJSL005
F1	TTTTATGGAGGAAAAGTCGCG	TTTGTCCTCCTCTTGTCTGTG
R1	GTTCAACATTATAAGCGGGGG	GGACTGCACCTTCTTAGTAGT
F2	AAATGCAGGTTTAGGACGAGT	CCTCCTCTAGCAACTAACGG
R2	ATCAGCAAAAGAAGTGGTAGG	TCCTTAAGAAACTTCCCTCCG
