# Supplementary material for: Viral metagenomics revealed diverse CRESS-DNA virus genomes in faeces of forest musk deer
Source: Virol J. 2020 Apr 25;17:61. doi: 10.1186/s12985-020-01332-y (PMC7183601; doi:10.1186/s12985-020-01332-y)
Supplement: Supplementary file 2 — Additional file 2. [file 12985_2020_1332_MOESM2_ESM.pdf]

>AUM61936
NSDFIKVDDDRINMKQVFNFMNMIINQTNKVTKAHPEVTPFYKYIMFGGIEFGKK-----
TNRMHVHGYVYCTKQKSIKE
LKKHWG---------TEAHFDVALMCDPIYEE-------GERPKQGNRSDIASAIEECETIGELMDSNPELYCKYRNGLK
DIYARKEALKPKTYEQ-----------------PEIIWNYGQTGLGKTRMAFEDEECVN-------YDNSFFSDWHES--
-KVISLEEFNGKIPYKT---LLQLTDGYH--NYYRINIKGGDKLVDLKRIYISSSVHPRDIYRQQDMKE---NEGGIDQL
MRRITKINHF-
>Circo_AAZ78351
KRWVFTLNNPSEDERKK---IRELPIS--EEGNEE-GTPHLQGFANFVKKQTFNK----VKWYFG-AR----CHIEKAKG
-TDQQ----------NKEYCSKEGS--------------QGQRSDLSTAVSTLLES-G-SLVTVAEQHPVTFVRNFRGLA
ELLKVSGKMQKRDWK------------------TNVHVIVGPPGCGKSKWAANFAT-------YWKPRNKWWDGYHGE--
-EVVVIDDF-YGWLPWD--DLLRLCDRYP---LTVETKGG-TVPFLARSILITSNQTPLEWYSSTA------VP-AVEAL
YRRITSLVFW-
>Circo_ABU48445
KRWVFTINNPTFEDYVS---VIEFCTA-EEEKGEKEGTPHLQGFLSLRKNARAAA----LEENLG-GR----AWLSRAVG
-SDEE----------NEEYCSKETR--------------KGRSSDLNAAASEVLAG-A-LMTDVARKYPTTYIMFGRGLE
RLRQLIVETP-RDWK------------------TEVIVLIGPPGSGKSRYAFEFK--------YYKARGKWWDGYEGN--
-DVVVMDDF-YGWLPYD--DLLRICDRYP---IRVEYKGG-MTQFVAKTLIITSNREPREWYKCEV------DC---TAL
YRRIDRYLVM-
>Circo_ADD62451
RRFCFTWNDHPVEAYEK---CEKFIEK-FEEYAPTTGMPHLQGFCNLNKPTRFST----IKKHLD-NS----IHIEKANG
-TDEQ----------NQKYCSKSGK--------------QGQRNDLQSLVEFIHEKRP-TIRDIATEHPTTYIRYFRGIE
RMLQLVNPIKQRDFK------------------TEVYYYWGPPGTGKSRRALEEAQ-----AIYYKPRGLWWDGYEQQ--
-DSVIIDDF-YGWIKYD--EMLKIMDRYP---YKVQVKGA-FQEFTSKKIFITSNVDTDELYKFVG------YT--TAAF
ERRITNKEYM-
>Circo_ADD62453
RRFCFTWNNYTVADTLT---VKDYFVK-YEELAPETGTPHLQGFCNLRKPQRFSA----IKKHLS-DR----IHIEKANG
-SDEQ----------NQSYCKKAGK--------------QGDRSDLKSVVSTIADGAN-TAQAIAEKHPVEFIKYYKGIS
EYLRLLAPIAPRKFK------------------TYVYYYWGPPGSGKSRRALEEAE-----SIYYKPRGLWWDGYRQQ--
-QSVIIDDF-YGWIKYD--ELLKITDRYP---YKVQVKGG-FEEFTSTRIWFTSNVDTDRLYRFDG------FT--PEAF
ERRITAKIHI-
>Circo_ADD62455
RRFCFTWNNYPDTAYEK---CETFIQK-FEELAPTTGTPHLQGFCNLRKPTRFST----IKKHLD-NS----IHIEKANG
-SDEQ----------NQAYCSKTGK--------------QGQRNDLQLLVDDVGHPDI-KTKDIAAKYPSTFVRYFRGIK
ELQRVVHPVAERMFK------------------TEVYYYWGPPGTGKSRRALEEAQ-----AIYYKPRGLWWDGYEQQ--
-DSVIIDDF-YGWIKYD--ELLKITDRYP---YKVQIKGG-FEEFTSKKIFITSNVDTDELYKFNG------YN--TAAL
ERRLTVKEYM-
>Circo_ADD62457
RRFCFTWNNYTELNYAL---CQEFIKK-YKELAPTTNTPHLQGFCNLQKPMRFST----IKKRLD-NG----IHIEKSMG
-SDTQ----------NQTYCSKSGC--------------QGKRNDLQSVVDTIQAGNG-SLSSIANEHPTAYIRYFRGIQ
EYIKTVRPIPPRYHK------------------TEVRYYHGPPGSGKSRRALEEAT-----AIYYKPRGTWWDGYKQQ--
-SCVIIDDF-YGWIKYD--EMLKICDRYP---YKVQIKGG-FEEFTSKYIWITSNIDTNLLYKFND------YN--DTAF
VRRIEIKLLI-
>Circo_ADD62461
RKFCFTWNNYEFDAYAK---CETFLNN-FEELCPSTGTPHLQGYVNLIKPTRFST----IKKHLH-NA----IHIEKANG
-SDEQ----------NQTYCRKSGK--------------QGQRTDLQLLVKDTMEPSN-TLKDIATKHPIAYIRYFRGIQ
ELRRMVLPVPPRNYP------------------TEVRYYWGPPGSGKSRRALQEAT-----EIYYKPRGQWWDGYEQQ--
-SCVIIDDF-YGWIKYD--EILKICDRYP---YKVQIKGG-YEEFTSTHIWFTSNVDTDLLYKFNN------YI--NTAF
ERRITIKEHI-

>Circo_ADD62471
RRFVFTLNNYTEDQYQK---CVEFIST-KKEVGEENGTPHLQGFCNLHKPMRFGT----IKKSID-NA----IHIEKANG
-SDID----------NQKYCSKAGQ--------------QGRRTDLESLVAGIAAGAN-TTSKVATEFPAMYIKYHRGIN
EYLKLAFPIKPRTEK------------------TWVYYYWGPTGSGKSRRALEEAQ-----SIYYKPRGLWWDGYHQQ--
-ENVIIDDF-YGWIKYD--ELLKITDRYP---YKVQVKGG-FEEFTSTRIWITSEKDTCDLYKYEG------YN--PASF
ERRITCKINI-
>Circo_ADD62473
RRFCFTWNNYSELEYVL---CCEFIKK-YKEIAPNTGTHHLQGFCNLEKPMRFST----IKKRLD-SR----IHIEKAAG
-SDSE----------NQTYCSKTGQ--------------QGRRTDLQAVVSAIQNGEITTPKMVAELHGAVYIKYHRGIE
KILHQLHPVPPRDIP------------------TELRVYWGPPGSGKSRRALEEAR-----TIYYKPRGLWWDGYEQQ--
-NCVIIDDF-YGWIKYD--ELLKICDRYP---YKVQIKGS-FQEFTSKYIFITSNVDTCDWYKFAG------YN--VNAI
ERRITIKEYM-
>Circo_ADD62475
KRWCFTLNNYTQLEAIT---IEQLLCT-EEEIG-ENGTPHLQGFFNLKKKKRLTS----LKAWLN-DR----AHYEEAKG
-SDEQ----------NRRYCSKSGK--------------QGQRNDLVLAAELLESG-G-NMAAVADLYPSAVIRYGRGLQ
QYWQLIGFS-ARDFK------------------TEVFVYVGPPGCGKSRAAAELG-A----AVYYKPRGEWWDGYNGE--
-ATVIIDDF-YGWLKYD--ELLRLCDRYP---HRVPVKGG-FVQFCSKRIILTSNIHVWMWYRFES------YD--ASAL
MRRINVYKLW-
>Circo_ADD62477
RRFCWTLNNYTEEDVTT---LQKDLAE-LRETCPNTGTKHLQGFCNLQRPKRFSS----IRKLFK-ER----AHIEKAKG
-SDFD----------NKAYCSKSGS--------------QGARNDLQEVVSVIEGGER-NIKAVALQFPTTYIKYFKGIE
QYIRICHSSAERDFA------------------TQVSFFWGPTGSGKSRRAYEEAK-----AIYYKPRGEWWDGYCGH--
-ANVIIDDF-YGWLKYD--ELLKICDRYP---YRVPVKGG-YENFVTKRIWITSEKPLEQIYRFIG------YD--CSSI
RRRLNTELYI-
>Circo_ADI48251
RRFIFTWNNYTDDDYDK---TCAFIKG-NKEHAPTTGVPHLQGFCNLSKPMRFSA----IKKCLS-NS----IHIEKAIG
-SDEQ----------NQKYCSKAGT--------------QGKRTDLEAVVRDMEQGTVR-LEDIAAKHPCAFIKYHRGID
QLLKLIHPIKPRDFQ------------------TYVYYYWGEPGTGKSRRAKSEAD-----AIYYKPRGLWWDGYRQQ--
-ENVIIDDF-YGWIKYD--ELLKICDRYP---YKVQIKGG-FEEFTSKRIWITSNVDIYSLYKFEG------YN--AEAF
KRRITCMVEM-
>Circo_ADU76993
RRFCWTLNNYTEDDVDQ---LQKDLPE-LREVCPTTGTKHLQGFCNLQRPKRFNN----IREIFK-GR----AHIEGAKG
-SDQD----------NQRYCSKSGN--------------QGARSDLEEVVSIIKGGER-DVKAVALQCPTAYIKYFKGIE
NYIRIYHATPERDFK------------------TEVYFFWGPTGAGKSRTAREQAL-----AVYYKPRGDWWDGYNGH--
-ECVIFDDF-YGWIKYD--EVLKICDRYP---YRVPVKGG-YENFIAKKIWFTSNKPLEQIYKFID------YE--PSAW
RRRLTVEREF-
>Circo_ADU77009
KRWCFTLNNPTEDEIKS---LDSWL---LSKEVGEQGTPHLQGFIHLKQKKRLSQ----LKQLFN--R----AHWEKARG
-SDED----------NEKYCSKEGR--------------GNR-SDLSGAVAAVKAG-R-AMTEVAREFSEIYVKYGRGLR
DLKLLIGQ-QPRDFK------------------TEVIVITGPPGCGKSRWAA-EYK-------FYKMKGEWWDGYDHQ--
-DVVVIDDF-YGWLPFC--ELLRVTDRYP---HKVPVKGA-FVEFTSRVIIVTSNSPPDAWYSEEK------CC--VQAL
FRRINKWLVW-
>Circo_ADU77011
RRFVFTWNNYPIEAYDK---CEKYLTK-FEEIAPETGTPHLQGFCNLHKPTRFST----IKKHLD-NS----IHIEKANG
-SDID----------NQKYCSKSGK--------------QGQRSDLAVVSNKILEGST-TIRDIATQHPETYIRYFRGIR
ELQRLVNPISPRDFP------------------TQVYYYYGPPGTGKSRTALEEAK-----KIYYKPRGLWWDGYTNQ--
-KCVIIDDF-YGWIKYD--ELLKICDRYP---YKVQIKGG-FEEFNSTHIWITSNVDTDLLYKFTG------YC--NAAF
ERRITIKKYF-
>Circo_ADY17982

RRFVFTWNNYTPSDFET---CITFLDN-FKEKCPTTQTPHIQGFCNLSKPMRFNN----IKKHLH-NS----IHIEKANG
-SDEQ----------NKIYCSKSGK--------------QGKRNDLDAVVLTIQNGTN-TISNVAKLHPISFIKYHKGIK
EYINHVNPIRPRHYK------------------SEVYYYWGPPGTGKSKTALEKAT-----AIYYKPRGLWWDGYQQQ--
-TSVIIDDF-YGWIKYD--ELLKICDRYP---YKVQIKGG-FEEFTSKYIFITSNVDTCDLYKFRN------YN--TDAI
ERRITEKIHF-
>Circo_AEL28794
KRWCFTLNNPTEQEVQT---CSRFV---EAREVGEQGTPHLQGFLHFRAKKRLSA----LKKLLQ--R----AHWEKARG
-SDAE----------NKEYCSKEGG--------------ANTTSDLAEAVAAVHAG-R-RMIEIARDFSEAYVKYGAGLH
RLHLMIGS-RPRDFK------------------TEVTVLWGPPGKGKSRWAA-DLK-------YYKMKGDWWDGYTGE--
-EVVVIDDF-YGWLPYC--ELLRLMDRYP---HKVPFKGG-YMEFTSKHIVITSNSPPNEWYKS-I------EN--KAAM
YRRFTRVLTW-
>Circo_AEL87786
RRFVFTLNNYTDEHYTK---CCAFITD-HKEVG-EKGTPHLQGFCNLTKPMRFNA----IKQHLH-NT----IHLEKANG
-SDED----------NQRYCSKAGV--------------QGQRSDLSCVVDRIRDGKT--ITDIATEFPTQFIRYHRGIR
ELLQVIKPIPPRDFK------------------TYVYYYWGPPGSGKSRRALEEAR-----AIYYKPRGLWWDGYKQQ--
-KNVIIDDF-YGWIKYD--EMLKICDRYP---YKVQVKGG-FEEFTSTRIWITSNVDIYSLYHFAG------YN--AEAI
KRRCTSIIEI-
>Circo_AEL87790
RRFVFTWNNYCDEDFVK---SCNYIKE-FKELCPTTGTRHLQGFCNLIKPTRFST----IKQRLH-NT----IHIEKANG
-SDEQ----------NQNYCKKSGT--------------QGQRTDLKLLVADIEQGIT-NIKDIASRHPTAFIKYHRGID
TYLKLTHPIQPRNYK------------------TWVYYYWGPPGSGKSKRALEEAQ-----SIYYKPRGLWWDGYKQQ--
-ENVIVDDF-YGWIKYD--ELLKICDRYP---YKVQIKGG-FEEFTSTRIWFTSNVDTDLLYKFDN------YN--VAAF
ERRITCKINI-
>Circo_AEL87792
RRFCFTWNNPTELDFAL---CSEFIKK-YKEIAPTTGTLHLQGFCNLQKPMRFST----IKQRLD-NR----IHLEKANG
-SDEE----------NQKYCSKSGF--------------PGRRTDLESLVSSIKNREVTTLKGVAEMHGACFIKYHRGIQ
AYMQQMVPIQPRDFK------------------TEVYFYWGPPGTGKSRRAAEEAR-----TVYYKPRGLWWDGYEQQ--
-KAVVIDDF-YGWIKYD--EMLKICDRYP---YKVQIKGG-FQEFKAEAIWITSNVSIDLLYRFTG------YD--PAAL
QRRMTCIDYM-
>Circo_AFL02442
RRWCFTLNNPTDGEIEF---VRTLG---PDREKGEQGTPHLQGYFHFKNKKRLSA----LKKMLP--R----AHFERAKG
-SDAD----------NEKYCSKEGR--------------DGH-RAFDGAVAAVMTG-C-KMKEVAREFPHIYVRHGRGLH
NLSLLVGS-RPRDFK------------------TEVDVIYGPPGCGKSRWAN-EQK-------YYKMRGEWWDGYDGE--
-DVVVLDDF-YGWLPYC--EMLRLCDRYP---HKVPVKGA-FVEFTSKRIIITSNKPPETWYKE-D------CD--PKPL
FRRFTRVWWY-
>Circo_AFS65290
RRFCFTVNNYDAETELK---VKNFLTN-NRELCPTTKTPHLQGFANLSKPMRFRK----IKESLC-DT----AHIEKANG
-SDED----------NKTYCSKSGQ--------------QGRRSDLDAVVATISTGER-DIRRIAEKHPNCYIRYGRGIR
SYLELVNPIPPRYFK------------------TRVVFFYGPPGSGKSRRALAEAQ-----AIYYKPRGEWWDGYHQQ--
-TSVIIDDF-YGWIKYD--DLLKICDRYP---YKVPIKGG-FQEFTSKHIWITSNVDTPLLYKFEN------YN--VAAL
ERRLEIKELI-
>Circo_AGJ74756
RRFVFTLNNYSERELNQ---ALFFINN-YEEIAPTTETPHLQGFCNLTKPMRFST----IKKHLS-DR----IHIEKAAG
-SDKD----------NQKYCSKAGS--------------QGERNDLAAVVDTIQSG-A-NIKSVAEHHPAAFIKYHRGIK
EYIRTIRPVPVRDFK------------------TEVFYFWGEPGTGKSRTALAEAK-----GIYYKPRGLWWDGYEQQ--
-ECVIIDDF-YGWIKYD--EMLKIMDRYP---YKVQIKGG-FEEFTSKYIWITSNINTDDLYKFEG------YV--NTAF
DRRLSIKKHF-
>Circo_AGJ74758
RRFCFTWNNYPLEAYTK---CEEFIKN-KEEICPNTGTLHLQGFCNLHKPTRFNA----IKRDLD-NS----IHIEKANG

-SDID----------NQTYCSKSGK--------------QGKRSDLMQVVETITKSEVPTLEDIATKHPVEYIRYFRGIE
KLQSILRPVCARRFK------------------TDVYYYWGAPGTGKSRTALEEAE-----KIYYKPRGLWWDGYRQQ--
-TCVIIDDF-YGWIKYD--EMLKIMDRYP---YKVQVKGG-FEEFTSKHIWITSNIDTDLLYKFSN------YS--NVAF
ERRLTIKKHF-
>Circo_AGJ74760
RKFCFTWNNYTEHDENK---CKDFIAQ-YKELAPTTNTPHLQGYCNLSKPMRFST----IKKHLH-NS----IHIEKANG
-SDEQ----------NKEYCSKSGK--------------RGQRTDLQSLLADIQDGNR-NIQTLAQSHPTTYIRYFRGIH
TYLNLVHPIAPRNFK------------------TDTYYYWGPPGSGKSRRALEEAT-----AIYYKPRGQWWDGYHQQ--
-EGVIIDDF-YGWIKYD--ELLKVTDRYP---YKVQVKGS-FEEFTSKHIWITSNVDTCDLYKFIG------YC--TDAI
ERRITLKSYM-
>Circo_AGL09969
ERYCFTINNYSEEDIEA---VKAFLVP-DKEKGEN-GTPHLQGFVNLKKKMRFNP----FKAALG-GR----AHIEQARG
-TDLD----------NKRYCSKGGK--------------QGKRSDLKEAVTLLNSG-A-NMTAVARAYPEVFIRYGRGLR
DYVITAGLSQQRAWK------------------TEVHVIVGVPGVGKSRHVSEQHI-------YWKPRGKWWDGYCNQ--
-EVVCLDDY-YGWIPYD--DLLRLCDRYP---LRVETKGG-TVSFVAKKIYITSNKQIKDWYNFEE------LKVDPRAL
YRRVTSYKVM-
>Circo_AHK80894
KRWVFTINNPTFDDYVH---VLEFCTL-DEEKGAN-GTPHLQGFLNLRSNARAAA----LEESLG-GR----AWLSRARG
-SDED----------NEEYCSKESG--------------KGRSSQLADATSAVLAG-L-PLTDVARKYPTTYVIFGRGLE
RLRHLIVETQ-RDWK------------------TEVIVLIGPPGTGKSRYAFEFK--------YYKPRGKWWDGYSGN--
-DVVVMDDF-YGWVPYD--DLLRITDRYP---LRVEFKGG-MTQFAAKTLIITSNKEPRDWYRSEF------DL---SAL
YRRINKYLVY-
>Circo_AIF76248
YRWCFTLNNWTEEEYGL---IESTC---RSKEVGESGTPHLQGFVNFKKKLRLSA----LKSLPGFTR----AHVESARG
-TDVE----------NQKYCRKQGS--------------RGKSSTLTEAVATLQNN-G-DLRAVAQMYPEVYIRHGRGLK
DYVMTAGLVGQRSWK------------------THVTVLIGRPGVGKTRYVNREVV-------YWKPRGPWWDGYIQQ--
-EVVVFDDF-YGWVTFD--ELLRVCDRYP---LKVPVKGA-FVEFVARAIYFTSNKPPEEWYDKEN------IKGSIEAF
FRRVNEYLVV-
>Circo_AIF76249
RRFCFTWNDYTLEDCAT---VEKFITE-YEEFAPTTNQPHIQGFCSLHKPMRFST----IKKRLH-NT----IHIEKANG
-SDEQ----------NQTYCRKSGF--------------QGQRSDLRAVVSAIQSGKN--AAQVAKEYPLEYIRYFRGIQ
ELDLITNAKPPRFYK------------------TWVYYYWGPPGSGKSKRALQEAQ-----SIYYKPRGKWWDGYNGQ--
-DCVIIDDF-YGWIKYD--ELLKITDRYP---YKVQVKGG-FREFTSRRIWFTSNVDTHELYKFIG------YN--NTAF
ERRITCKINI-
>Circo_AIF76251
SRWCFTVNNYTTEHIEQ---LRKLGSTLLYENAPTTNTKHLQGYIRLNKPKLFRQ----LQLLLP-PA----THIEATKG
-TERQ----------NFRYCTKSGT--------------QGQRTDLEQAVHELHENGT--ILQVATNNPTVYVRNWRGLH
ELRNLLIPVKPRDFR------------------SNIYILYGPTRTGKSRAAQKIAG------DYYKNRSNWWHGYHQE--
-ETVIIDDF-YGWIKWD--ELLKLCDRYP---YKVETKGG-YEEFTSKTIIITSNLPPEKWYKFEN------FD--PTPL
SDRINAIVET-
>Circo_AIF76252
RRFCFTLNNYTEAEYQH---IVEFINA-KKEVADTTGTLHLQGFCNLSKPMRFSA----IKKSLN-NR----IHIEKANG
-SDEQ----------NQKYCSKAGR--------------QGQRTDLDKLVRAIQNGCN-TTQMVAKEFPTAYIRYHRGIS
EYLKLSHPIKPRDEK------------------TWVYYYWGPTGSGKSRRALEEAR-----AIYYKPRGLWWDGYHQQ--
-DCVIIDDF-YGWIKYD--ELLKICDRYP---YKVQVKGG-FEEFTSKRIWITSEKDTDRLYKFED------YN--PASF
ERRITCKIFI-
>Circo_AIF76253
YRWCFTLNNWTEEEYGL---IESTL---RSKEVGESGTPHLQGFANFKKKLRLST----LKKLPGFTR----AHVQPANG
-TDLQ----------NQKYCQKEKR--------------RGRSGELSKAVDLLKNG-G-NLSEVADVYPEVYIRHGRGLR

DYVTAACLSGQRDWK------------------TRVIVIIGQPGVGKTRYVNSQCV-------YWKPRGPWWDGYTNQ--
-ECVVFDDF-YGWVTFD--ELLRVCDRYP---LKVPVKGS-FVEFVAKFIYITSNEPPENWYDKEN------IRGKIEAF
FRRIDEYLVI-
>Circo_AIF76254
RRFCFTWNNYTEDDIWT---VQTFITT-QKELAPDTNTPHLQGFCNLVKPMRFSA----IKELLS-HR----IHIEKANG
-SDIQ----------NQAYCSKAGS--------------RGQRTDLNAVLDDIRGGETS-LARLATAHPCTYIRYFRGIE
NYLKLVHPILPRDFP------------------TVVNYFYGPPGSGKSKRALEEAQ-----EIYYKPRGLWWDGYRQQ--
-DCVIIDDF-YGWIKYD--EMLKIMDRYP---YKVQIKGG-FEEFTTKFIWITSNVDTDKLYKFEG------YN--PEAF
NRRLTTHVRF-
>Circo_AIF76261
CRWCFTINNWTEEEYGQ---ICSFT---EDKEVGREGTPHLQGFVNFKKKLRRDT----LKKLPGFTR----AHVEPAKG
-SDME----------NQRYCRKQGL--------------QGKSSALQEALKTLENR-G-DLAAVATAYPEVYVRHGRGLR
DYVNTSGLVPRRQSK------------------TVVTVVIGPPGVGKTKYVNDCIS-------YWKPRGPWWDGYQQQ--
-ECVVLDDF-YGWVPFD--ELLRVMDRYP---LKVPVKGA-YCEFNSKKLFITSNKPPEEWYNEEN------ICGTLQAM
FRRFNEVFRM-
>Circo_AIF76265
YRWCFTINNWTSEEYGL---IESTL---RSKEVGEKGTPHLQGFANFKKKLRLST----LKKLPGFTR----AHVERAKG
-TDLQ----------NQKYCRKGNS--------------QGKKPELTVAVRKLESN-G-DLVKIAKEHPEVYIRHGRGLR
DYVNTAGLVGQRAWK------------------TYVVVIVGEPGVGKTRYVNSECV-------YWKPRGPWWDGYAGQ--
-EAVIFDDF-YGWIMFD--ELLRVCDRYP---LKVPVKGA-FVEFVAKKVYFTSNKPPEEWYDKEN------IRGNIEAF
FRRINEYLVI-
>Circo_AIF76266
RRFCFTWNNYTEPDYEK---CKTFIVE-LKEVAPNTGTNHLQGFCSLKKPMRFST----IKKRLD-NR----IHIEKANG
-SDDD----------NEKYCSKAGK--------------QGARNDIQAVISAIQNGEVTTPKMVAAVYGSVYIKYHRGIE
KYLSLLRPVPERNFK------------------TELRIYWGPPGSGKSRRALEEAN-----SVYYKPRGLWWDGYEQQ--
-DSVIIDDF-YGWIKYD--ELLKICDRYP---YKVQVKGS-FQEFTSKYIFITSNTDIYSWYKFPG------YE--TAAI
ERRAEIHEYI-
>Circo_AIF76280
KRWCFTINNPTPAEERH---LREIPVN--REVGAQ-GTPHIQGFVNFVKKKTLNQ----VKLFVG-PR----AHVEKARG
-SDEQ----------NRDYCSKDGR--------------PGARHDLASAVSTALST-G-DLGQVAQSHPETYVKYHRGLA
ELIKASNKLPRRAWK------------------TEVHVHVGPPGCGKSKWASEFAT-------YWKPRNKWWDGYASH--
-EVVILDDF-YGWLPFD--ELLRICDRYP---LEVEVKGG-LVPFLARTVIITSNKMPQEWYSSDA------VP-HAEAL
YRRITTLISW-
>Circo_AKE49355
RRFCFTWNNYTEEAYKK---CEDFINT-YEEIAPNTGTIHLQGFCNLHKPIRFNK----IKKHLD-NS----IHLEKANG
-SDED----------NQKYCSKSGS--------------QGHRSDLQAVVETIAQSNS-TLQDIATKHPTAYIRYHKGIR
EYLRMVKPIQERQHK------------------TLVYYFWGPPGSGKSRRALEEA------KIYYKPRGLWWDGYNQQ--
-PNVIIDDF-YGWIKYD--EMLKIMDRYP---YKVQVKGG-FEEFTSERIWITSNIDTDTLYKFIN------YT--NTAF
DRRITLKCHM-
>Circo_AKO84203
RRWCFTINNPTPEEEDA---VKNLAP---REVGEN-GTPHLQGFVNLKKTTRMGA----LKSRLG-GR----GHFEPARG
-DDCS----------NQDYCSKGGK--------------QGKRNDLHDAVSTLKET-K-SLAAVAAAHPETYVKFSRGLR
ELLLISEMATPRKWK------------------TEVNVLVGPPGCGKSRYCLETAA-------YWKPRGKWWDGYDGH--
-SDVILDDF-YGWLPFD--DMLRLCDRYP---LRVETKGG-TMNFIGRRIFITSNKLPHEWYN-DE------IG-NKAAL
YRRLTSVTVW-
>Circo_AMH87650
RRYVFTLNNYSDDDVAR---LQQLEA---KEVAPTSGTKHLQGFINFGRAVRFNS----ARKLVG-GD----GVTEASGG
-PNGD----------CFDYLPHGNS--------------QGRRTDLQRVADLAQSGTT--AHTIAREFPVEFIKYGRGIT
NLLRTINPIPSRSKP------------------TIVVVLVGAPGVGKSRFANEVGS-----GTYYKPRGEWWDGYCQQ--

-RSVIIDDF-YGWIKYD--ELLKICDRYP---HKVPVKGG-YEEFTSEYVFITSNSNVDQWYKFDN------YS--PAAI
HRRLSVYVGL-
>Circo_AMH87652
RRYVFTLNNYGPEDESR---LANLDG---REVAPTTGTKHLQGYINFGRAIRFNT----AREMVG-GS----GVTEAFGP
-PNGV----------CFDYLPHGNS--------------QGRRTDLQRVADLAQAGTT--AHTIAEQFPVEFIKYGRGIT
NLLRVINPVKSRTKP------------------TNVIVLVGTPGVGKSRFANEVGS-----ATYYKPRGEWWDGYYQQ--
-RSVIIDDY-YGWLKYD--ELLKICDRYP---HKVPVKGG-YEEFTSEYIFITTNSLIDLWYKFDK------YT--PKAI
LRRLAVYVTL-
>Circo_KJ641742
KRWCFTINNPSPAEERH---LREIPVN--RERGAQ-GTPHIQGFVNFVKKKTLNQ----VKLFVG-PR----AHVEKARG
-SDEQ----------NRDYCSKEGR--------------PGARHDLASAVSTALST-G-DLGQVAQSHPETYVKYHRGLA
ELIKASNKLPRRAWK------------------TEVHVHVGPPGCGKSKWASEFAT-------YWKPRNKWWDGYASH--
-EVVILDDF-YGWLPFD--ELLRICDRYP---LEVEVKGG-LVPFLARTVIITSNKMPQEWYSSDA------VP-HAEAL
YRRITTLISW-
>Circo_KT732825
KDYCFTVNNYDDDGVRL---LSGLVQGGIYECG-ESGTKHLQGFIQFTRKVRFST----VQASLP-KG----THIEKRRG
-TPEE----------ASEYCKKEGK--------------SGNRSDLEAVAKEVARGAS--DFQIAETFPAVFVRYYRGLR
ELRCVLNGNVQRSWK------------------TRCHVYYGYPGSGKSLQAEPIG------------------------
----------YGWIVFD--ELLKICDRYP---YRVPIKGG-YRNFVARDIFITSNSGPDSWYEREP------IK--REAM
SRRFEYVIEF-
>Circo_KU230452
KRWCFTLNNPTDEEVQK---IKDMN---PSKERGEQGTPHLQGFLHLKCKKRLNQ----LKEFLA--R----AHWEKARG
-SDED----------NEAYCSKEGK--------------GNR-SDLSGAVAAVKAG-R-AMSEIAREFSEVYVKYGRGLR
DLALLIGQ-KPRDFK------------------TDVILITGPSGVGKSRWAF-EYK-------FYKMKGEWWDGYANE--
-DVVVIDDF-YGWLPFC--ELLRLTDRYP---HKVPVKGS-YVEFNSKTIIITSNTHPETWYNEEK------CY--LQAL
FRRINKWMFW-
>Circo_NP_573442
KRWCFTLNNYTAEEEAK---VRALL---PGKERGEQGTPHLQGFLHFKKKQRLSA----LKKLLA--R----AHWEKARG
-SDHD----------NEEYCSKENQ--------------GNR-SDLAGAVAAVKAG-R-RMVDIAREFSEIYVKYGRGLR
DLALMIGQ-KPRDFK------------------TEVVVITGPSGVGKSRLAS-EMK-------FYKMKGDWWDGYSNE--
-DIVIMDDF-YGWLPFC--EMLRLMDRYP---HKVPVKGS-YVEFTSKKIVITSNTHPESWYCPDK------CY--LPAL
FRRINKWMYW-
>Circo_YP_764455
KRWCFTLNNYTDEEVSA---VKAWN---ASREKGENGTPHLQGYIHLKKKARLST----LKKLLS--R----AHWEKARG
-SDSD----------NEAYCTKDGE--------------GNR-SDLSGAVAAVKAG-S-RMVDIAREFSEVYVKYGRGLR
ELALLIGQ-KPRDFK------------------TEFIVVTGPSGVGKSRYAN-EYK-------FYKMKGDWWDGYSNE--
-DVVVIDDF-YGWIPFC--ELLRLTDRYP---HKVPVKGS-YVEFTSKVIIVTSNTHPDSWYNEEK------CY--LPAL
FRRINKWLTW-
>Circo_YP_803546
RRWCFTLNNYTPEEEET---ARNLIHD-ADKEVGESGTPHLQGFMHFKQKQRLTA----LKKLFP--R----AHFEKARG
-SDQQ----------NADYCGKDGD--------------NNP-SDLAGAVAAVKRG-S-QMSEIAREFSEVYVKYGRGLR
DLRLLIGC-PPRDFK------------------TEVIVLIGPPGCGKSKLAN-EMK-------FYKMKGDWWDGYDNQ--
-DIVIIDDF-YGWLPYC--ECLRLCDRYP---HRVPVKGA-YVEFTSKKIVFTSNRHVDGWWKG-E------IE--KSAF
YRRINVYKFY-
>Circo_YP_004152331
RRFVFTWNNYNADDIEK---VVKFIKE-KEEIAPTTNTPHLQGFCNLKTPMRFKS----IKTHLH-TN----IHIEKAKG
-TDLQ----------NQEYCKKGNT--------------QGKRTDLETLVSAINSGTN-TQQKIAKEFPICYIKYHRGIN
EYLKLIHPIEPRKEK------------------TWVYYYWGPPGTGKSRRALEEAS-----QIYYKPRGLWWDGYHQQ--
-ENVIIDDF-YGWIKYD--ELLKITDRYP---YKVQIKGG-FEEFTSKRIWFTSNVDTDKLYHFID------YN--NAAF

ERRITCKIHI-
>Circo_YP_004376332
KRWCFTLNNPTAEERRH---IQEIITA-DNEVG-DSGTPHLQGFLNMKTKRRLGT----MKKWFN-AR----AHYEAAKG
-TDLQ----------NDEYCTKGGK--------------ERCRNDLQKAIDVVRSS-G-SMRAVAEACPATFIRYGRGLR
DYANVMQYRKPRDFK------------------TEVNVYVGDPGCGKSRKASEL-------CVYYKPRGMWWDGYDGQ--
-ENVIVDDF-YGWMPCD--ELLRVFDRYP---CKVPVKGA-YVEFVSTNIYVTSNKHVWQWYKFEG------FD--PAAV
MRRVNVYLVY-
>Circo_YP_007974237
KRWVFTLNNPSPKEIDH---ILGQDMN--KEMGEG-KTPHLQGFANFSKKKTFNQ----VKKIFG-SR----CHIEKAKG
-TDKD----------NQQYCTKEGS--------------GGQRTDLASAVSTVLES-G-SLRAVAESHPVQFVRYHRGLS
ELLKVAGKAKARDWK------------------TSVHVIIGPPGCGKSKWACNFAT-------FWKPRNKWWDGYCGQ--
-EVVILDDF-YGWLPYD--EMLRLCDRYP---LTVETKGG-TVPFLARTILITSNKVPQEWYSSES------VP-TAEAL
FRRITTLQLW-
>Circo_YP_008130363
RRFCWTLNNYEEDELAS---LQKDLAE-LREVCPTTGTKHLQGFCNLQRPKRFNS----VRRIFG-GR----AHIEKAKG
-TDLD----------NKTYCSKSGT--------------QGARTDLQEVVSCIEGGEK-NLKRLAIQFPVAYIKYFKGIE
QYIRIAHGSGERDFK------------------TECFFYWGATGLGKSRAAREESK-----ATYYKPRGEWWDGYCGQ--
-PNVVIDDF-YGWIKYD--ELLKICDRYP---YRVPVKGG-YENFCSKRIWITSNYPLDTIYRFLN------YD--KGPL
ERRIDVIKHF-
>Circo_YP_009000900
RRVCFTLNNYTEEDLKF---IEEHFKS-VKKVG-ENGTPHSQGFINLKKKTHFNS----VKKLL--PR----AHIEKAKG
-NDQH----------NKDYCSKQEG--------------QGKRNDLADALATVANG-G-DIKALEDSHSLVYAKYKRGIL
ACIDDFGWKKPRDWK------------------TEVHVLWGIPGCGKSRYAREQA-------PYYKPRGEWWDGYTGQ--
-EDVILDDF-YGWLKFD--ELLKICDRYP---YRVPVKGS-FVQFTAKRIYITSNVAASEWYHFQG------YD--PTAL
YRRMTSYLTW-
>Circo_YP_009021843
RRFCFTLNNFTEEEYEK---VTRFIQD-YNETAPTTGTIHLPGFCNLTKGMRFNN----IKSKLA-TR----IHLEKANG
-TDEQ----------NQIYCRKSGG--------------QGKRTDLVSLVEGIQNGQI-RLSDIAKDHPIAFIKYHRGIR
EYLQLTKPVQPRMFK------------------TWVYYYWGPTGSGKSSRALKEAM-----EIYYKPRGLWWDGYHQQ--
-DNVIIDDF-YGWIKYD--EMLKIMDRYP---YKVQVKGG-FEEFTSRRIWITSNVDTDQLYKFIG------YV--SDAF
DRRITNKVYI-
>Circo_YP_009021870
RRFCFTWHDYDCEDVAK---TESFINT-HKEVCPDTRRIHLQGFCSLAKPKRFKW----IKEQLS-NR----IHIEKAMG
-SDKE----------NQQYCSKSGEG-------------SGQRTDIQSLLETIQGGEH-DIRRIAEKHPACYIRYYRGIR
SYLNLVAPVSPRNFK------------------TEVRYYWGPPGSGKSRRSLEESS-----GVYYKPRGEWWDGYMQQ--
-TSVIIDDF-YGWIKYD--ELLKICDRYP---HKVPIKGG-FEEFTSKYIFITSNVDVCDLYKFNG------YT--TAAI
DRRITIKENI-
>Circo_YP_009021891
KRYCFTINNYLPEDEAA---VKEFLTE-AKEVGES-GTPHLQGFCNLKKKMRFEP----FKRAIG-GR----AHIEQSRG
-TDVD----------NKRYCSKGGA--------------QGKRSDLKEAVTLLNNG-G-TMTDVARAHPETFIRYGRGLR
DYVIQAGLTKPRAWK------------------TEVHVIVGPPGVGKSRHVQETAL-------YWKPRGKWWDGYTGQ--
-SHVVLDDF-YGWLPYD--DLLRLCDRYP---LRVETKGG-TVEFVAKVIWITSNKQVKDWYDYEE------LKVDARAL
YRRLTTYQVM-
>Circo_YP_009047065
RRFCWTLNNYTEEDVDV---LQKDLTE-LRETCPSTGTKHLQGFCNLQRPKRFNS----IRQIFG-GR----AHIERAKG
-TDLE----------NKEYCSKGGA--------------QGTRTDLEKVVSVIEGGER-SLKNLAIQFPIAYIKYYKGIE
QYIRIAHGNSERDFK------------------TEVFYFWGPTGTGKSRKAREESL-----ATYYKPRGEWWDGYTGQ--
-PCVVIDDF-YGWIKYD--ELLKICDRYP---YRVPVKGG-YENFVSKYIWITSERCLEEVYRFIG------YD--CSSL
RRRLNKEIFF-

>Circo_YP_009091696
KRYVFTLNNYTTEEYAR---IDNVGAD-GKEVG-ENGTPHLQGFINLKVKKRFSQ----IKEMLG-SR----CHIEKARG
-TDLE----------NRVYCSKEGG--------------QGKRSDLDAAAETLRTSLG-DLRSVAELYPSQFIRYGRGLR
DYASVLGLVKPRDFK------------------ANVTVITGPPGCGKSRYAADH-------APYYKPRGDWWDGYHTN--
-ATVILDDF-YGWIKLD--EMLRICDRYP---HQVPVKGG-YVQFLARDIFITSNKPVEEW--FPN------CD--CSAL
FRRINVYLTW-
>Circo_YP_009110680
RRFVFTWNNYTSEDYDK---CCEFIKS-NKEIAPTTGCPHLQGFCNLHKPMRFGA----IKKHLH-NS----IHIEKANG
-SDED----------NKKYCSKSGK--------------QGERTDLQSCISDIQSG-A-TIKKIAEDHPAVFIRYHRGIR
EYIRTVMPIKERDFK------------------TEVYYYWGPPGSGKSKRALEEAK-----QIYYKPRGLWWDGYQQH--
-DCVIIDDF-YGWIKYD--EMLKIMDRYP---YKVQIKGG-FEEFTSKYIWITSNVDTDDLYKFIG------YK--TDAL
ERRITNKEYM-
>Circo_YP_009116910
RKWVFTVNNYTDDEWNE---LKRWCMV-NKEVGPQCGTPHIQGYLSANKPHRRSG----MVEVCR--R----
ARWEAAKG
-NDTQ----------NDDYCRKAATG-------------AGQRTDLLRVAAAVDSGVS--LREIASENPSEFIKFHRGIL
AYRNLVRPAVPRDFK------------------TELHVFVGPPGSGKSRRAQELAS-----SVYRKVRGPWWNGYEQQ--
-QSVIIDDF-YGWVPFD--ELLRVADRYQ---HQVEIKGG-FEEFNSKIIIITSNRPIKDWYKFDS------YD--PEAL
YRRCSVYQHI-
>Circo_YP_009126896
RSYSFTLNNYTDDEVFA---LTSMCEPINKEIAPTTGTPHLQGCIIFTSPRSFKA----LKKVVPFNR----ANYKPTIS
-EPG-----------SARYCKKDG---K-----------QGKRSDIENVRDILTSGGN------IRDVVSTATSIQSVRM
AEIHLKYFEKKRNWK------------------PTVRWYYGATGTGKSKTAYEECD-----EYVAMSTGKWFEGYDAH--
-THVIIDDMRKDFMKFH--ELLRMLDRYA---FMVECKGG-SRQFLATDIYITSCYAPDEMFESR---------EDVNQL
IRRLDEIRKF-
>Circo_YP_009134739
KRWVFTLNNPTEQEVES---VKSLP---PSKEKGEQGTPHLQGFLHLKKKVRLNQ----MKQLIP--R----AHFEIARG
-SDED----------NEQYCSKEGK--------------GNR-SDLAGAVAAVKAG-R-PMTEVAREFSEAYVKWGRGLK
DLALMIGQ-KPRDFK------------------TEVIVLTGPSGVGKSRWAN-EQK-------YYKMKGDWWDGYSNE--
-DIVVIDDF-YGWIPFC--ELLRLCDRYP---HKVPVKGS-YVEFTSKKIIITSNTHPNHWYNEEK------CY--MQAL
FRRINKWLFW-
>Circo_YP_009170674
RRWCFTINNYGAPDLEC---VNEFR---CEKEKGAKGTPHLQGFIHFTGNWRFNR----VRNLLG-GR----AHIEKARG
-NDDQ----------NKAYCSKEEF--------------QGKRNDLGRVASALESG-A-TLSEVARASPEVFIRYGRGLR
DYMNVRGLVKPRDFK------------------TEVIVLVGEPGSGKSKYANELPK-------YWKPRGQWWDGYNGE--
-DIVVLDDF-YGWVPYD--ELLRIGDRYP---LKVQVKGA-FVEFTSKMLVITSNKRPEEWYDKEK------IA-DQSAM
WRRFDKMYYC-
>Circo_YP_009237526
RRVVFTLNNYTNEILTS---ICNYAES-NKEVAPTTGTPHLQGFLHFKNPKTHKT----LGKVL--PG----GHFLHAKG
-SDED----------SQVYCSKEDH--------------QGKRSDLDDAILTLESG-G-DLKRVAMEHAGAYVRYHRGFA
AYKSLVCATAPRDFK------------------TKLVVLFGPPGTGKTRAAYELAG------PYPKPRGEWWDGYCGN--
-NGVIIDDF-YGWLKFD--ELLKISDRYP---YRVPIKGG-YENFCSKIIIITSNIDISKWYKFDG------YD--PAAL
YRRCTKYLRC-
>CRESS_AXH77830
KSWCFTINNPTGNDDWE---LEALKT-----ETGEQGTRHYQGYVVMNGQRTLRV----MKTLL---TR---AHLEARRG
-THQQ----------AADYCKKDGQMSAA----------DKNKCMWRNMIEWAEKGEL---DKIKEEYPSFYLRY---LE
KFRSLSRPTLSILPE------------------LENEWWWGATGTGKSRKLWA---------HYAKQLNKWWDGYDGE--
-DVVAIEEWAP-KNECTASLLKIWADRYP---FPAEVKGGKLNRIRPRKLIVLSNYSPEQCFG---------QEEDLGPI
RRRFRVIHFE-

>CRESSV1_FJ959078
ERFTARTFCVTVNNR-----DEVEQVQESRELAPETGRVHIQAYVELYMAAGIVM----LKRLFNCP-T---MHVEKRRG
-TQDQ----------ARNYCMKETRDGKT--P----GNGQGKRNDLTDAVEALEAGGV---EAVVSKHPNTYVRYHRGIH
ALYQAKL-EAIASRQQ--R-------------NVKTAVFYGEPGTGKT-HCAFELA-----RNAGRNQSVWFNGYQNQ--
-KILVVDEMNGDWIGWQ--LLLRMTDKYP---LQCQTKGG-MVWAMWEIVIFTSNAHWEDWYPYYAG-----G-
MDKAAL
KRRIHKVVKF-
>CRESSV1_KF133822
----LRNVCFTLYNADP--EDVASKLSELEEKCPESDRLHLQGYLECTKPVRFGT----LKDFLGS--T---VHLERRRG
-NRQQ----------ARDYCRKEET-K------------QGQRTDLDAIREMVVAGAT--EESIADEYFGTWARNHRAIA
RYKFLKSKPRD-------F-------------KPRVIIRWGVAGSGKTRGVYDTHE-----PR-PNGGTVWFDGYDPH--
-EVVLLDDFYG-WLPWS--MLLQMLDRYP---MSVPKKGSTCNFR-AKFIYITSNADPETWYD-YSK-----PGIEFEAL
KRRVDETHHF-
>CRESSV1_KF246569
----MWALTVKNWDLFD-LEEIGNYQDGQKHTGEKTGYQHCHLNLELEKRQYMTW----IKRVLFNIPD---
MHCEKRQG
-TREQ----------CDTYLAKDGEFE------------RGHRSDLDEIHDMIKEGKD--LFDVYESHFASTVRYSSGLE
KYIVLHDSKRARNSET--T-------------APQVIVYVGPAGSGKSWHCFNDED-----YPIQMYEKVYFDGYNRE--
-KTIWFDEFNGRSMPFG--KFCQLADRFP---GIYETKGGSVLISGLKKILISTISYPATWWG-SNR-----FNLDPDQL
YRRLTKCYYL-
>CRESSV1_KJ206566
----YRKVFVTSFN----TSAMEQDLVDCMEHAPTTGRIHYHLYIEFYSQKTMNT----IKKILKD-AT---ANIQPARG
-TPQE----------AVDYIKKDGATN------------QGHRSDLDDVFQRLQVGDN--ILDIIEGHPGTCIRYIKGIL
AVKGLVDQRRQRQEAR--Q-------------MPTVLVYIGKSGAGKSHACSQDPD-----YPVQGPSKVYFDGYTGE--
-STIWFDEFGGSVLPFH--VFLRLADKYE---TRVETKGGSVCITGLQKILISTTTPPKLWWE-SRK-----FNEDPYQL
WRRLTRVYYI-
>CRESSV1_KM573766
----YSNWILTTWDPYL-KENWWEMDNHEHCHEEQR--EHWHIYFELNHRKNMAS----IK-EWLNDLT---VHCEVRRG
-TGQQ----------AIKYCQKGFDFQ-------------GSRSDLVNCKQLLEEGVS--MIELAQAHFGDFVRYYRGLY
QYADLLSREKQRTAGL--S-------------EVEVTVFIGPAGSGKTYNCSKKAE-----YMQQQNGKCYFDGYEKQ--
-KCIWFDEFTGSTMQFN--HWCRLADKYG---VRVETKGGSVQISGLKRIIISTIIPPGEWWN-SQS-----FRDDPEQL
WRRITEIYYC-
>CRESSV1_KM874309
KLLMRRNVVGTLTNERP--HEITS-----YEICPSSKRRHLQFYCEFVGQQRLGA----VRQLFGP--T---VHVEPRRG
-TVDE----------ARTYCSKEDTPK------------SGKRSDLADIKEELDAGSS--LKSISQQHFGQFLRYRKSFE
AYIVLNQDPRT-------W-------------EMENSILWGEPGTGKTKLAYDLKE-----MR-NQNGNVWFDGYHGQ--
-EILLIDDYYG-WIPLA--FLLQLLDRYP---MNVQTKGGSVPFT-SKKIIITSNKSPECWYN-WSK-----FGKNFGAF
ERRINQVFHY-
>CRESSV1_KM874347
RNWCFTLDG-YTDNI-----ADMVSD-EEEEICPESSRRHLQGYVEYTVSRRLAA----VKLSLGSE-S---VHLEGRRG
-TASE----------AAEYCEKEDGE-------------QGARSDLATVASAVISGKR--VEEVALEFPTTFIRYNRGIE
KLTNVVR-RKEQMRNL--R-------------MLKVVVLWGDAGTGKT-RTAFELS-----QDG----QLWWDGYEGQ--
-KHLIIDDFYG-WIKWG--LFLRILDIYP---LQLPVKGG-FTIASWDVVFITSNCMPRNWYERG----------MPAEL
ARRLTHIVEF-
>CRESSV1_KP153497
KSWCFTIHSRGEGDC-----DWLHPI--GMERCPETGKLHLQGALQLDKQQRLSF----MKKLHKT------AHWEVMKG
-NWTQ----------SIEYCSKSEKM--------------GQRTDLETIGKMVKENKT--NLELVDTLGAGVSKFQKHIS
FTYSEK----DSDRQA--T-------------GVKVIVLYGPTGTGKT-YAAVNSN-----SQK--ATKLWFDGYENQ--
-KVLVIDDFDG-SVEFR--YLLRMIDVYK---FPAEVKGG-MVWGVWDTVIITSNVHPASWYTDT-----------SPL

KRRIAEIRLC-
>CRESSV1_KT149404
R-WVYTLFAAAEDDY-----SNLDDF-ERLECCPTTSKVHLQGYVEFEKPQRMAA----LKK-LNS--T---VHLETAKG
-TREH----------CVRYCTKDETT-------------QGRRNDLLETTLAIVNGELS-RDDVFDTRPDLICKYARGIN
ELLTYRA-KKERQGDE-----------------LHTEVLWGDAGVGKT-RYAYGRC-----KGG----ALWWDGYDGQ--
-SILVIDDFYG-WVEHS--VLLRILDRYP---FKIDIKGS-STYANWKEVYITSNRHPSTWYTRAPW-------TEDKAL
QRRLGAIYEC-
>CRESSV1_KT862256
----VWTMTVKNWDLFD-EERIQNQTEGSKHTGQQTGYQHCHFNLELEKKKTMAW----IKRELFNRED---IHCEPRRG
-SREQ----------CDSYLNKDGEFK------------PGRRTDLDDIHDMIKEGAS--LYDCYEEHFGTVVRCERGLR
DYIALRDTILATKKKY--P-------------APEVIVYVGPSGSGKSWHCSEDPD-----YSIQMDSKIYFDGYNNQ--
-KTLWFDEFSGKTMPFT--KFCQIADRYP---GRYETKGGSVLIYGLKKILISTVEYPALWWG-SDR-----YNKDPEQL
FRRITKCYYL-
>CRESSV1_KU043411
----MRCATVTSFN----IDYIKRDVINNLEVCPETGKLHYQMYMEFEEKVTVKQ----IKRLLRD-NG---AHVEPRYG
-TAKE----------AIEYCKKDGNQ-------------QGERTDLADVYESLKVGRS--LLDIIEEHPGTYIRYFRGIE
RVQDLFRRKQQKLEER--V-------------QPTVLVYIGKSGTGKSHHCYHDPD-----YPVQQAGKVYFDGYDGE--
-STIWFDEFGGSVLPFG--VFLRLCDKWE---TRVETKGASVCITNLRKILISTTTYPKNWWD-SRK-----YQEDPKQL
WRRLTHVYYI-
>CRESSV1_KU043424
----FRFACITAWN----MDAFDPQRMHDRETCPRTGRHHLQCYFEAPNPKTVEQ----WQDVVCDKPR---AHVECRHN
-EGDR----------AADYCKKDGDG-------------QGARMDLADCKTMIDEGKQ--MIDLYEKHFGTCVRCHRGLM
LYKDLVDRKRRKEAEP--E-------------PKEVVVYVGASGSGKSHHCWHDPD-----YPLLAENKVWFDGYEGE--
-EVLWIDEFRGSVFPFG--LFLQVTDKWG---ARVEVKGGSVETF-FKKILISTTVPPGEWYK-CPN-----FLSNPQQL
WRRLTKVYWL-
>CRESSV1_KX388513
AEWT-TYG---------------DPTTMNLEYTQNQDNFHWQGYVQFHKSLRFTQ----VKKYLKSY-T---AHVEQANG
-TLEE----------NIQYCSKSASE-------------SGERTDIERLYVDVKSGKS--MACIIDHHTNTWFKYHGAVD
KYLEQK-----VACQK--R-------------TCTVTVWVDQGQTGKTTRALYDIN-----ERTHEEGRLWWDGYDGQ--
-PTLVIDDCVH-LIKFD--YWKSLVDGHP---MRIQIKNG-WKNALWNRVIINSNRHPNNWWPDNSE-----ANLSMPYF
KGRIALINHV-
>CRESSV1_KX388515
RHWTFTIGGEEAQTITG--IEDWDATTMNLEYTQNEDNFHWQGYVQFHKSLRFTQ----VKKYLKS--T---
AHVEQAKG
-TLEE----------NIQYCSKSASE-------------SGERTDIERLYVDVKSGKP--MASIIDHHTNTWFKYHGAVD
KYLEQK-----TACQK--R-------------NCKVTVWVDQGQTGKTTRALYDID-----ERTHEEGRLWWDGYDGQ--
-PTLVIDDCVH-LIKFD--YWKSLVDGHP---MRIQIKNG-WKNALWNRVIINSNRHPNNWWPDNTE-----ANLNMPYF
KGRIALINHV-
>CRESSV2_FJ959082
RNWCFTYNNYDSSPQK--------CMK--YEVG-ESGTPHLQGFVIFKN-AVAKP----SQYFKPAYH------FEKARG
-TPQQ----------ALEYCQKDGNKTV---A----DGGEANKRRYEEAFSAAKEGR------MDDIPADIYIRHYSTLK
KIRFDHAPPAQNNDVL----------------NNY--WVYGPSGTGKSKSVREF--------LYVKNQNKWFDGYEGE--
-DFVLIDDVHP-NWSGK--TLKIWSDHYP---FSPETKGGHIKMIRPEGIIVTSNYTIEEMYEAE---------EDRQPI
RRRFKVIKEY-
>CRESSV2_JF755415
RGWCFTINNPTE-DDSL---VA---LE--KEVGE-EGTPHLQGYCYFKNPKSLAQ----LKVLLPRAH------LEAQKG
-SHEQ----------AIEYCKKEGDMTQ---K----RKGEIGKEFWDEQLSLAKKGR------LEEIDSK----------
----------------------------------GHWYYGSTGTGKSRKARE---------FYLKGCNKWWDNYQGQ--
-EVAIIEDFDKVHSVLG--HLKIWADRYS---FPAEVKGS-QINIRPKKIIVTSNYHPNEIWT---------DLATLDPI

LRRFNIIKFS-
>CRESSV2_JX185415
KNWCFTSFNVDPPK------FDPIT----RETCPTTKKSHLQGFICLKERKRLAG----VKKLLGSVH------LEASRG
-TVDQ----------NIQYCSKDGESSG---P----AT-----SPFALAIAAATDGRL---DDVKSTHPGIYLRYKKTLE
SLQKFRSDDLDGSC---------------------GVWICGPPRIGKDYAVRV----------YCKALNKWWDGYLGE--
-PNVLISDVEPDHFKIG--YLKIWSDRYP---FIAEIKGS-SMKIRPEKIFVTSNFRLSDCCNG----------EILGAL
QSRFTIYDMF-
>CRESSV2_JX904107
RHFAWTMNNPGP--DTE---AAV--IA--REVGE-SGTPHLQGMTSFEHARSLSA----IIKLLQKAHP---GTHVEICR
-DAYK----------SMVYCQKDGDKKP---A----DGGGMERDRWKRAYEMAAEGN------LEDIDADIKLRFYGTLK
KIKEDHQVTPTSLPT-----------------L-DFHWYQGSSGSGKSKFAHD---------YYLKSPNKWWDQYEPG--
-QTVIIDEWDPNHKVLA--SLKKWADHHP---FAAEIKGG-TRMLRPPKLIITSNYTIKECFP---------QENDHLPL
LRRFTVKQFG-
>CRESSV2_JX904185
RNYVFTRNNYED---TT---FDT--LE--KEIAPTTGTPHLQGTVCFASPRSFEA----VR-KLMGCH------IEVCA-
-DLQK----------SITYCKEDGDMTQ---K----EKGESNAQRWRDIRLAAEEGR------MDDIPDDVRFHHIR-TI
EHHRDLASKKRKIAT-----------------EEQHLWYYGASGTGKSRKARE---------AYLKMCNKWWDGYDDE--
-DVVIIEDFDKKHDVLV--HMKIWADRYP---FLSEYKGG-ARKIRPQKIIVTSNYHPEEIWS---------EESDLGPI
LRRFKLHKFS-
>CRESSV2_JX904344
RNWVGTLNNWTV--DEY---VQL--HS--KEVAPGTGTPHLQMCFFFKNKKSLRA----LQKINGRAY------WEHMRG
-TQEQ----------AADYCKKDGDTP----K----QKGETQKIRFERAWELAKEGN------LDDIDSDIKLRFYGTLK
RIKHDHMLQEILTDT-----------------ESQMEWYCGPSGTGKSRKARE---------AYLKMCNKWWDGYEGE--
-DIVLIEDFDKKHDVLV--HLKIWADRYP---FLAECKGS-SMKIRPAKIIVTSNYHPTEIWS---------DVSDIEPI
LRRFHVTKFG-
>CRESSV2_JX904420
RNFTFTLNNYTQEHLTT---LEILVEQ--KEVG-ESGTPHLQGFICFTTTKSMKQ----CIKAIPGAH------VEFMKG
-TIEQ----------NVAYCSKDNDMTQ---K----KKGEAEKERWEEALAAAKEGR------FDDIPADIQFRYDRNIK
RIYAENKPKPQTLNKL----------------TNE--WYCGPSGTGKSKQARD---------AYVKLNNKWWDGYADE--
-ETVIIDDFDKYDIALS--GLKRWSDHYP---FPAEFKGG-VKVIRPKRIIITSNYTPEEIWEEE---------ATLGPI
RRRFHITQFK-
>CRESSV2_JX904562
RSFSFTWNNYTE--ADI---ARL--KG--KEVGE-SGTPHLQGMINFKSPRSFAS----VMKELEGAH-------VEKTI
-SSYD----------SMVYCKKDGDVSQ---K----AKGDMEKARWKRTRELAAAGK------IEEVDDDIYVRFYGTLK
RIKEDHQQVPPAQAE-----------------L-NFHWFYGASGTGKSRAAYA---------LYIKNSNKWWDGYVDQ--
-PCVLIEEWDPNLAMMA--SMKKWADHHP---FSGEIKGG-TKMLRPPKIIVCSNYTIQECFP---------NEQDWKPL
ERRFKVRKFG-
>CRESSV2_KC248416
RNYVFTLNNYTPVHEIT---LNS------REVAPTTNTPHLQGYICFPNAKTISA----VRRILAGCH------VEVARG
-SHAQ----------CRTYCIKDGDADP---R----EIGNAEADRWEDAWEKAKAGA------IEEIPADIRIRSYSVLR
RIGRDYQPNLALLP------------------ATCGYWIKGESGAGKSHSCFT---------LYPKGPSKWWCGYQNE--
-EVVLLDDVDPSHGLIG--GLKRWADKYP---FIGESKGG-SFKIRPKKFIVTSQYSIEDCFQ---------DVETRVAL
NRRFRVINKL-
>CRESSV2_KF738877
RGWVFTINNYNEWDFVN---ISKLEK-----ERGEEGTPHLQGFAYFKQRISFNG----IRDIL---TR---AHVEIQRG
-FNAQ----------AIDYCKKEGERGAG----------QKDK--WKDVLQLARQGKV---QEIEERYPAIFLRY---FQ
KLCGFYRPEHSIILEN----------------FTNEWWWGPTGTGKFKKLND---------PYEKSLDPWWDNYQRE--
-EIVAIEEFEP-RCKIN-SFLKRWADRYP---FRCEVKGAFLSKLRPLKIIVISNYQLDECFP---------NSKDLDPL
KRRFKEIHFP-

>CRESSV2_KJ547648
RNFVFTWNNYSDASKTY---LST------EEVAPTTGTRHLQGFIAFTNAKTIQQ----ARSKLPGCH------VETMNG
-SIAQ----------SEDYCSKAGTISN---D----NNGRAEKLRWQRARDFAKEGK------LDEIDADIFIRCYSTLK
RIKSDYATKPQPID------------------PVC-IWIYGPTGTGKSHAVET---------CYKKCMDKWFDGYAHE--
-DAIYLEDIDKYQVKWG--GLKRLADRWP---MQASIKGA-MAYIRPKFVLVTSNYRIDEIWT---------DPQTLEPL
QRRFTEIEKL-
>CRESSV2_KM573767
KRWCYTLNNYTPEEEQN---IQN--IE--KEVGEN-GTPHLQGFICFVNRKVFNA----VKKLFKTER----IHLEVSRG
-TIEE----------ASEYCKKDGNEE---SH----RKGEATKRKWEEARENAKQGK------FEDIPADMWIKYRRSWI
AEYTDNLN-KDITEIKDID-------------KDHFYWIYGPTGTGKSHLARQLAQ-----APYLKGLNKWWSGYENQ--
-KVVIIEEWNPEASKLG--SLKQWCDKWP---FTAETKGGHFVGIRPERIIITSNWNIDECFN---------DSHESEPL
HRRIREIYKK-
>CRESSV2_KM573776
KRWCFTINNYTEEDTSR---CER--ID--KEIGEEEHTPHLQGFIVFKNRKTFNV----VKRIIG-EN----AHIEIARG
-TVKE----------ASEYCKKEGQPE---QN----ERGQATKRKWEETLKAAKEGR------FDDIAPDLYIRYRSSLK
AIYQEEVN-KNTKEITDFD-------------KGHFYWIYGPTGTGKSHLARTMAA-----SPYLKGLNKWWSGYKMQ--
-KAVIIEEANPETCKLA--PFKQWCDKWP---FTAETKGGSFEGIRPQYIFITSNYSINECFP---------DPNDSEPM
KRRCHEFFKE-
>CRESSV2_KM598396
KLYCFTSFAVDEPS------YDPTA----RETCPDSGRTHLQGFVAFKNRQYFTA----CKKYFGTAH------VEACKG
-TFAE----------NQEYCKKDGDEIK---S----GG-----DVFNDVLRKAEAGSI---QEIKDLYPGLYIRYKKTLE
SIKRFNAEQLEESC---------------------GIWLTGPPRSGKDYAVSI----------YSKMLNKWFDGYEGE--
-ECVHLSDMDKNHV-MG--SLKIWCDRYP---FRAEIKGG-TMVIRPKYIVVTSNYKLEDIFDG----------SMLSAL
QARFMVMCY--
>CRESSV2_KM821755
RNYVFTVNNYDDEDEHQ---WMEV-----KEIGE-SGTPHLQGYICFTQLKSLNQ----LKGFFPSAH------FETKRG
-THQQ----------AADYCKKDGDVDDC--G----SAGAAMDELMGHTIECIRNGDY---KGIPNATHFI--KAYRVLK
EQQQD-----RTL-----D-------------TLTHQWRWGKAGCGKSKPARD---------AYLKMCNKWWDGYTGQ--
-EVVIIEDFDPDHKCLV--HLKIWADRYA---FPAEVKGG-KIDIRPKTIIITSNYHPRDIFD---------REPDLEAI
MRRFNVTHFM-
>CRESSV2_KM821764
NRWCYTLNNPEPIPWNE---TTQ------KEIAPGTGTPHHQGFICFKNQKYLTQ----VKELNDKAH------WEIAKG
-TNQQ----------ASDYCKKEGDEDP---Q----RLGKANEVKWRLINDLAKAGNL---AEIDIKYPKVWNTSYRNLK
SIKVDHMKPLADLD------------------EPCGVWLYGASGAGKTMKART---------AYLKLPNKWWDGYQGE--
-ANVIIDDVDPNHAYMG--YLKIWADRYS---HLAEVKGS-TIVVRPRKLVITSQYRIKDIWAS--------EPATMSAL
LRRFKEVYAF-
>CRESSV2_KP153360
RRIVFTKNNWTEEDYRA---LMEEP-F--KEIGE-KGTPHLQGYAEFTKKAKYGA----LAKKY-KMH------CEVSRG
-TQDE----------AIKYCMKDGD-TKK--L----DG-SAEKNRWEQARVSAKEGRL---DDVP-ADIYI--RCYRTLK
EIAKDNMPKPESL-----T-------------ELKNLWIYGPAGSGKTRLADA---------SYSKNCNKWWDGYQNE--
-PGVIINDVGKEHSVLG--HFKLWGEHRP---FIAETKGG-AIHIRPQRVIITSQYSLTQIWE---------DEETRDAL
ARRYKVLHDI-
>CRESSV2_KP153364
RNFTFTQNNYGD---TE---LDG--VQ--KEVGESD-LLQLQGVICFNEKIRESA----VRKKLPGCH------IEIAK-
-VLPA----------AIEYCKKDGDMTP---K----EKGALEKRKWDEIRVACEEGR------FEDLPDDIRYKNLR-LN
KMHRCEALRSRELES-----------------EAQHLWYWGEAGTGKSRKARE---------AYLKMCNKWWCGYTEE--
-ETVLIEDFDKKHDVLG--HLKIWGDRYP---FLAELKGD-TMKIRPKQIIVTSNYHPSAIWF---------DEETLLPI
LRRFKCVEFK-
>CRESSV2_KP153369

RGWCFTCNNYSESDYNG---FLAAE-S--KEISS-TGTPHLQGYIYFRTEKSLRT----LRKLSDRSH------WEPAKG
-DSDS----------NFCYCSKGENATGK--R----KA-ANGKENYAETVELAKKGKL---DEID-PGHLL--RFYGSIK
SLQKDNLVRPSDS-----E-------------ELTGLWIYGPSGCGKSKYVR----------DYYKLANKWWDGYRQE--
-ETVLIEDLGTEHDKLG--HLKLWSDRYA---FSGETKGG-MLSCRPKRIVVTSQYSIEEIFL---------DEKTQEAL
NRRFTKLRLF-
>CRESSV2_KP153377
RAWCYTLNNYTE--EER---DSL--RS--YERGA-ADTPHLQGYVQFAHQKTLSA----VKKLLPRAH------LEERRG
-TIDQ----------AVEYCKKDGDMSQ---K----EKGKEEKNRWKRILEKADEGDE---EWLRENEPNVAFKHMATFR
SHKK-PRVGTLQYEE-----------------T-PHEWWVGPTGTGKSRKAHE---------HYAKEKNKWWCGYTGQ--
-ETVIIEEADPKTMELA--ALKVWADRYP---FPGEIKGGRIEGIRPLRVIVISNYTIEECFA---------NQNDVEPL
RRRFKEVKFG-
>CRESSV2_KP153404
RAFIFTWNNPTADTEAA---LES------RETAPTTGTRHLQGYIRFTDGKSLRS----ARRLLNGAH------VEVAR-
-TIRQ----------AIEYCHKEGDVDD---A----ARGDMEKARWEIAWTKAKTAD------LEEIDADIRVRCYSALT
RIQKDYMVPPLPLP------------------APCGLWIHGLSGVGKTFAVYQ---------LYSKNASKWWDGYQNQ--
-DHILFDDMDPDVGKAG--RFKIWADERP---FIADIKGG-SISIRPKIFIVTSQYTIDECFG---------EIQTRMAL
SRRFRIIEKL-
>CRESSV2_KP153447
RGWCFTLNNYTPEHEAL---LAA------REVG-ANGTPHLQGFVYFPNAKTFNG----AKAVLPGCH------LEAAMG
-SVAQ----------NVEYCSKDGDLSA---A----EKGASEAQRWKDARLAAVSGD------IADVPDDIYIRYYRTLK
EISKDHMARPDGLD------------------GVCGLWLYGAAGTGKSRYAQE---------HYMKMTNKWWDGYQGQ--
-DVVVMDDMDPDHACLR--HLKRWADRYP---FIGETKGG-AISIRPKKFVVTSQYSIEDMFKNRDGT----DVETVAAI
RRRFEVKRFG-
>CRESSV2_KP153468
KRWCFTLNNYSAEEQLA---FKAST-V--IEKGE-SGTPHLQGFVVFMTAKRLSA----LKKINPRAH------YEQAKG
-TSLQ----------ASTYCQKEGVLEPT--A----VG-NASNESYRKAMDLAKLGDF---SAIEQEWPGLWLRHRNAFL
SAPRDFGIRPKDL-----D-------------YLPGIWIWGPAGVGKSRLARE-----------EKRLNKWFDGYSAQ--
-KAVLIEDIDQSHRYIS--HLKIWTDRYS---FPAEIKGG-AIQCRPEHVLITSQYHIHDIWD---------DVQTREAI
NRRCFVIYID-
>CRESSV2_KP153483
KNWCMTLNNYDDSEVAR---FESHMRP--FERGE-NDTPHLQCFFSLKAKKRMSC----LKKIFPRAH------FEVKSA
STMEQ----------ASDYCKKEENDNST--A----RG-KAISDNYEETVELAKKGDI---EAINPEHV---LKYYPTIK
RIAHDNKKMPSDLLWE--E-------------HPPNIWIYGPTGTGKSYRARA---------FYSKMAQKWWDKYDGE--
-EGVLIEDMDILHNYMG--PMKIWADKYA---FPVEVKTS-GDRIRPKVIVVTSNYTIEQIWP---------DRSTHGPI
SRRFKVIHVL-
>CRESSV2_KP153485
RNFVFTWNNYTEDSEQF---LRSRVDE--KEIAPSTGTPHLQGFCSFENKKSVQQ----VRVILVGCH------VETMLG
-SISQ----------NEDYCSKAGEVTN---D----NKGRAEQLRWQRARESAKLGQ------FDDIDADIFIRCYSTLK
RIRTDYTAKPPPED------------------VTC-YWIYGPTGTGKSHSVET---------CYKKNMDKWFDGYQGE--
-ETVYLEDIDKYQVKWG--GLKRLADRWP---LLVNTKGS-MQYIRPKRIIVTSNYNLDEIWS---------DSGTLDPL
LRRFTVILKE-
>CRESSV2_KT149394
RHVVFTINNWTEEIWLE---VTQFD-L--KEIGE-SGTPHLQGYGCFHKKQRYAT----LARKW-KGH------FEVSRG
-TPQQ----------ASDYCKKGGDESQS--A----KG-EATKKLYEDAFSLAQKGEI---LSIP-EPLRT--RFYATYK
KVAKDYMPKPESL-----S-------------ELKNLWICGEAGVGKTVLADL---------AYSKNCNKWWDGYQNE--
-PGVIINDLGIEHKVLG--HLKLWGEHRG---FIAETKGG-ALHIRPERIIITSQYEIGQVFE---------DWETRDAI
RRRYKQHKAL-
>CRESSV2_KT149398
RRIVFTYNNYPPEYNAW---LDS------REVAPGTGTPHLQGYAQYVNPKSITA----FRRLFMGCH------VEPARG

-TGSQ----------SRTYCSKDGSTDE--------NPGNREKQRWEDARSLAKEGK------FDQIPADIYIRYIGNLH
RIYREILPPLEPLP------------------ATCGRWLLGRTGSGKSKGVRS---------VYPKPLNKWWDGYDDH--
-THVLLDDVDHNQSSIG--NLKIWSDHYP---FIAEKKGG-SRLIRPELIIVTSQYSIRELFN---------DNELVLAL
ERRFQVINVN-
>CRESSV2_KT149412
RNYCFTYNNYPN--TEL---VDN------HEVAPTTGTRHLQGYIAFHSTKTLAA----ARRLLPGCH------LSAMAG
-SISQ----------NDAYCSKSGTASN---D----DKGRAEKLRWQRARDLAKAGN------LDEIDADIYIRCYSTLK
SISKDHMIKPEPVD------------------VKC-FWIHGATGTGKSYCVET---------CYKKSMDKWFDGYDNQ--
-DVVYLEDFDVYQIKWG--GMKRLADRWP---MQASIKGS-MKYIRPKIVIVTSNYTPNEIWM---------DPQTVEPL
LRRFKVIEKV-
>CRESSV2_KT732816
RGWCYTVNNWTENDEEL---VAK--LS--REIGE-EGTIHLQGYCYFKNFKSLAQ----FKALLPRAH------LETQKG
-TPQE----------ASDYCKKDGDMSQ---K----RKGDLGREYWEEQLSLAKKGK------VEECDPKLQITHFNALN
AISARYAPMPPDNED-----------------I-DNQWFYGPTGTGKSRKARG---------CYLKMCNKWWDGYLGE--
-ETVLIEDFDKKHEVLG--HLKIWADRYA---FPAEVKGS-KVNLRPKKIIVTSNFHPNEIWS---------DLSTLDPI
LRRFNVIKFS-
>CRESSV2_KT732819
RNFCFTLNNYTESDTEA---VK--AID--FEVG-EKGTPHHQGYIRFENARSLGA----TIKQLPKPH------VEVAKG
-NASQ----------NIAYCTKSGVIDS---E----TCGRNEKRRWTDARAAAKEGR------FDDIPDDIYMRHVHAIK
RIRMEDGPKPTDLEPR----------------DTYGLWIYGPPGTGKSHFVRTN--------HYIKGANKWWTGYCGQ--
-KYVVIDELSPASGPLS--QMKKWADRWS---FEAETKGG-NSIIRPYMIIVTSNYSIEEVFSR----------VDAQAI
KRRFTEIKFQ-
>CRESSV2_KT732823
KYWCFTAFASDFEKERV---FSNSD----EELCPLSGRKHIQGFAAFASRKRLTA----CKKLFPSAH------FERMRG
-SARE----------AIEYCKKDGKCVR---S----STGSAKVDRIKDFIELARAGNV---DLCEQRHPNQFLRYFSQLK
NLRKFDIERLSE-----------------------ALWLRGPSGIGKDASRSF----------FKKSQLKWWDGYNNE--
-NIILISDFDESCK-MV--HLKIWADVYP---FNAEVKGS-TIKIRPKYIIVTSNFRMDMLFLG----------TALDAL
KRRFMTFEF--
>CRESSV2_KU043397
KRFCFTFNNYTEEEEKK---LKE--LE--KHEEGEEGTKHLQGFVVWKTARRPGG----LKKINA--K----IHWENTHG
-TSKQ----------ASDYCKKDGKDKDFEHR----KKGAANKVLWDNIYKLAKEGK------FDEIPRNIYIRHMNNLH
KIHDMTMKDRSMDEFTNKD-------------KDHFLWLWGGTGTGKSTNARRIAK-----KPYLKGLNKWWNGYKCQ--
-RVTIIEEANPKACELA--HFKQWADKWS---FPAEIKCSSFDSIRPEYIIVTSNYSIRECFP---------AEQDYEPL
ERRFTEIELV-
>CRESSV2_KU043406
RRYCFTLNNYTRDEEEA---IKQ--IN--HEHSSGQGTPHLQGAITFNKRTYLAT----LKKLIP--R----AHFEIMQG
-NPQQ----------SKDYCTKETSSK--PQH----QAGEANEAKWESAYQAAKEGR------FDDIPRSLWIKHMRSFK
QIFEDAKQDTDMTEYTDQD-------------KHHFLWIWGPTGTGKSHTAHRIAK-----EPYIKDLNKWWNGFTKQ--
-RVVIIEEADPKRCELA--SFKKWADKWA---FTAEAKGTVFPSCRPEYIIVTSNYSIAECFP---------EDADRLPL
ERRFTEQYLP-
>CRESSV3_HM228875
VNWCFTLNNYTNEDVNK---LKQVK----FEVGDKKQTPHIQGFIQFEKKVRLSV----WKKINK--K----IHAEIMKG
-TIEQ----------AINYCKKSGKM--------------GERRDLKEAKKKC-AEVG------LRAITDCTYNLQVIRN
CQIMLEYHEKERDFK------------------PEVIWIYGESGAGKTKYISEKCA-----EYWKDAT-KWWNGYDRH--
-EITVMDDFRASNMKMN--ELLKLIDRYP---HRVEIKGG-FRQMLSKKIYISSIMHPKDVYNLP--------EEPVKQL
LRRIDTIIKI-
>CRESSV3_JN857329
RCCVFTLNNYTEDDMIQ---LNEWD----KEIGKTNKTPHLQGYIEFNNRKYFST----LKAFNP--N----IHWEKRLG
-SQLQ----------AINYCKKEGVQ--------------GKRNDISYYKELA-LKEG------MKKVVEE-GNFQEIRT

CELYLKYKEEERNFK------------------PEVIWIHGESGSGKS---LKASE-----EYWKDKS-QWWDGYDKH--
-NTIIMDDFRSSDMKFD--YLLRLLDRYP---MRVQVKGS-SRQMLSKKIIITSIYSPEEIYSYNN------NREPINQL
LRRIDKTMKL-
>CRESSV3_JX185418
IGWCFTLNNYTEIDEQV---LQALD----HEVG-EGGTPHLQGYLELHKKKRFKQ----VKEMLG-ER----YHIEMRRG
-TAAE----------VDAYCRKQNGQ--------------GLRSDLDRVRLLA-AESG------MRAVAAT-ANYQQIKT
AEIYLTYNEEPRSWK------------------PTVYWIYGASGTGKTRKAYEICG-----TYVKVGTHKWWDGYDGH--
-ENVIIDDFRPMGIPFV--DLLGILDRYE---FRVEVKGK-MRQMLAKTIVITTIYPPEVLY-VNL------KDEPLQQL
LRRIDHFINF-
>CRESSV3_JX904075
RSWCMTINNPTNADKAS---LCGLEV--REFNDPDK-TPHFQCYIRWKNAKTFSR----MKKLFP--R----AHLKVARG
-TDKD----------NKKYCSKEEE--------------QGSRNDLKGIIDMVQQNPS------MDYIID-VSSLQAIRT
AEKLLVYREPRRSWK------------------TEIYWFHGSTGTGKSRRAYELFP------YTAMDTGQWWEGYDGD--
-EVVIIDDMRRDFLKFH--QLLKLFDRYP---YRVEVKGG-SRQFLAKTIVVTSCYSPEDMFDTRD-------QEDIQQL
LRRITEIKSF-
>CRESSV3_JX904076
RGWFLTINNPTEDKLKS---LRYVHK--SEETCPTTGTPHYHVYFRLKDAVTFSK----IKKDIP--R----ANIEPAKG
-NDQQ----------CKDYCSKQNAG-------------QGKRTDMSKVKDLISEDPR------MSSIIE-VGSLQSIRT
AEKLLIYKEPKRNWK------------------TEVYWFCGATGTGKSKLAFELHP------YVAMDTGEWWEGYDAH--
-EEVIIDDMRRDFLKFH--QLLKLFDRYP---YRVQFKGG-SRQFLARTIIVTSCYSPEEMFETR---------EDIQQL
LRRLTKIKYF-
>CRESSV3_JX904139
RAWCLTINNYTEEELEC---IVSYSS--NE-TCPTSGTKHLQCYIRMTNTKTFTK----MKKDFP--R----AHIEMAGG
-NDLQ----------NKAYCSKESK--------------QGKRSDLEKISNMILESPR------MNDMID-LSNLQAIRT
AEKLLMYKEPQRNWK------------------PTVMWFCGRSGTGKSKMAAELLP------YWAMDTGKWWEGYDGH--
-EHVIIDDMRADFLPYN--HLLKLLDRYP---YRLEVKGG-SRQFLAKTIIITSPYSPDQMFGIMH--------EDNKQL
LRRIDKIKYF-
>CRESSV3_JX904407
RSWCFTYYPTNEEDLLW---FKNLTT--TRELCPTTGKLHFQGYISYNNAKTFQQ----TKKWFQLDK----IHIAPAKG
-NDFQ----------NQVYCSKEHK--------------QGKRSDIVRAIDIITQTNS------VSAVLE-VNNYQAVRH
CELWLKYKEPCRPVQS-----------------INVIWIHGSSGSGKTRKVYDDNS------GFTPTSHKWWEGYDGH--
-QVVLIDDIRRDFCKFH--ELLKLLDIYP---FRVETKGG-SRQVQFKTIYITAPYSPIEMWEGRC-------DEDLLQL
TRRITQTIDI-
>CRESSV3_JX904581
-DYCFTDFVLDET---F---LQGLP---YRETCPTTGKKHLQGYIYFKNAKTFSA----VRKLLQPR------SVRACKG
-SAEQ----------NATYCSKENK--------------QGERNDIHDIMENIQTGNT------MRDIVS-ATSFQSIRI
AEIQMKYFEPIRQWK------------------THVSWFYGKSGTGKSKLAYEMCE------YMCMETNKWWEGYDAH--
-EDVIIDDYRRDFCKFK--VLLHLLDQYP---MRVEAKGG-SRQFRAKRIIITTPKSPQETWEGRT-------DEDLYQL
TRRIDIIKEF-
>CRESSV3_KF738883
KRWCFTLNNYTEEEYKE---IEKIEW---REVGEEG-TPHLQGYIELKKKSSLNQ----IKKVNQ--K----IHWEQLRG
-KPFQ----------AADYCKKENMSGG------------GTTEKIGAIIEKA-KSGG------MREVLKP-PKLATVRL
IEKYLSYCEEERKEK------------------PYVHWNLG----NITTYTGRMTP-----N----------DGYDKN--
-ETIVIDDFRASIMRFT--YLLRILDRYP---MRVEVKGG-YRQLNSKNIVITSIIHPESCYHME--------NEPIRQL
MRRIDKITET-
>CRESSV3_KJ641718
RCICFTLNNWTDEEYQQ---LVNWDQ---KEIGKTNNTPHLQGYIEFKKRISLSK----LKKFNP--K----IHWEPRKG
-TQSQ----------AITYCKKENKQ--------------GERTDLVKARTIV-QEHN------MRTLLQLPVNIQQIRM
CEKYLTYCEEQRSWK------------------PKVIWIWGPSGSGKSRLAHAMTE-----NYLKDES-QWWDGYDKH--

-ETIIIDDFRGKQMNFT--YLLKLLDRYA---MKLQVKGG-YRECLAKKIIITSIFSPDRSYAFLN------EEEPMKQL
YRRIDTFINM-
>CRESSV3_KJ641722
VNWCFTLNNYTDDDVKA---LKLVK----FEIGEEKKTPHIQGFIQFEKKVRLTA----WKKINK--K----IHAEIMKG
-NVDQ----------AIAYCKKGGKE--------------RQRTDLREAKKKC-AAEG------MRAITGEEYNNQVIRS
CEIMLKYHEKVRDFK------------------PEVVWVYGNSGSGKTKYVKEKCA-----AYWKDST-KWWDGYDRH--
-ETVVMDDFRASNMKMN--ELLKLIDRYP---HRVECKGS-FRQMLSKKMYITSIMHPKEVYNLP--------EEPVQQL
LRRIDKIVKV-
>CRESSV3_KJ641729
RNFVFTLNNYTEDEYNF---IKNFEQ---KETGAQG-TPHLQGYMELKKQVRFNT----VKSVFP--R----MHIEPRRG
-TQKQ----------AVDYCKKEKM--G------------GIRNDVASVIEFK-NSS-------FKSFFESGADIQAFNL
LGKIKTYMEEKRDYK------------------TFVIWCYGPSGSGKSRWAYEISR-----KYFKDDT-KWWDGYDNN--
-SVTILDDFRASNMKFN--TLLKLLDRYP---YLIEIKGG-YRQFVSSIIIITCIQKHEDTYKILE------NDEPLNQL
TRRINKIMSF-
>CRESSV3_KM598404
--HNFVFTSFKTEDFER---VLGLD----KETCPETKREHLQGYCELDKKKTMAG----IKKLFSDDS----LHIEARKG
-TQKE----------AIIYCQKEGKQ--------------GSRGDLDGARVMA-VESG------MRAVSS--MNMQQIRV
AEKFLSYNEEVRDWK------------------PEVLWFWGDSGSGKSREARFISH-----GYVKNTGGKWWDGYDGH--
-EVVIIDDFRDSWWALT--YFLACTDRYE---FQVEVKGG-FRQLRAKMIIITSLFHPKYMYRAQA------KGDTAYQI
LRRIDSVVHF-
>CRESSV3_KM598406
RAWCFTLNNFTPEEL------AEIEN---KDEIGEKGTRHLQGYLYWKNPRKFTF----IKKIIP--R----AHIEPAKG
-NPQQ----------NITYCSK--DPKQ------------GKRTDLETMREILKEPEP------MRKITE-SLNYQATRS
AEKWLCYNERKRDWK------------------PEVTWYTGPSGTGKTRAAKEELP------WKSG-TNKWWDGYDGH--
-KEVIIDDFRGSSIAFT--DLLRLLDRYP---MQVECKGG-SRQFLAEKISITSIYHPEKVYKNLE-------EEPIEQL
LRRIDKIVNL-
>CRESSV3_KM874300
RAYCMTIHNWSEETVNQ---LKNLK---YNETCPTTDRKHLQVYAYFKNALSFKS----IKKQFPTA------HIEASKG
-TPNH----------NKTYCSKEDE--------------QGKRSDIEVCREIVQSGAG------MREVVN-CQSVQGMRI
AESYLKYHEKPRKWK------------------PTVQWFYGESRSGKTREAYEILG------YTCLNTGKWFEGYDAH--
-ENVLIDDIRKSFMPYD--EFIKLIDRYA---FRRRMLRK-FPPVFSKKDNYHLLLSPRRCLEGRY-------------
----
>CRESSV3_KM874304
-NWCFTLHNYTDDDIEI---FKNIKC---KEICPTTKRQHLQGYIQFDNQRTITA----VKKFLGLPK----IHLEIANG
-SSDD----------NKAYCSKDG---Q-----------QGRRTDITDIKDMISNGAT------MRDIIP-ATSVQSVRM
AEIHLKYSEQKRTWK------------------PKVQWFYGPTGTGKTRTAYEIL------EYTTLDTGQWWEGYDAH--
-ENVTIDDMRGDFMKYH--VLLKLLDRYA---YIVECKGG-SRQFLARHIIITSAFHPKDVFHTR---------EDIAQL
MRRIDCVKEF-
>CRESSV3_KM874317
RSYSFTLNNYTDDEVFA---LTSMCE-INKEIAPTTGTPHLQGCIIFTSPRSFKA----LKKVVPFNR----ANYKPTIS
-EPG-----------SARYCKKDG---K-----------QGKRSDIENVRDILTSGGN------IRDVVS-ATSIQSVRM
AEIHLKYFEKKRNWK------------------PTVRWYYGATGTGKSKTAYEECD-----EYVAMSTGKWFEGYDAH--
-THVIIDDMRKDFMKFH--ELLRMLDRYA---FMVECKGG-SRQFLATDIYITSCYAPDEMFESR---------EDVNQL
IRRLDEIRKF-
>CRESSV3_KM972726
QFVCWTLNNYSEDEYQS---LIEFSE---REVGETG-TPHLQGYGELKKRKKFAA----LKNLFP--R----VHWEQRRA
-SRDA----------AANYCKKEGKN-A------------SEKSSKEAITRVK-SGQT------MRAILDP-PNLSGIRM
CQIWLSYNEPKRNFK------------------SEVFWYYGASGTGKTKLASEQAG-----YWHDG-T-KWFDGYDAH--
-EHVLLDDYRGGNMKFN--FLLKFLDRYP---LRLEVKGG-YRQLLAKKIWVTSIKHPKEIYSFSE------MDEPTEQL

LRRITTIKKM-
>CRESSV3_KP153408
RDWCFTLNNYTEEEYDV---IHSLSL----KEVGESGTNHLQGYIYFVNAKSMSA----VKKMIS-KR----CHLESAKG
-SPLQ----------AATYCKKDNN-VI-----------QGKRTDLDEIRDILKQTNK------MSDVVM-AKSYQSVKM
AEQILKYHEKPRMEK------------------PYVEWYYGPTGTGKSKKAYEVLS------YTCLSTGKWFDGYDAH--
-KNVLIDDMRKDFMKFH--ELLRLLDRYA---MRVECKGG-TRQFVASHIIITSCYHPKDMFETR---------EDIQQL
LRRIDKIENF-
>CRESSV3_KP153422
RTKAVHFVSFETSNETV---ERLASD----EHCPTTGRHHLQCYLYTKNKITLST----IHKAL--KK----CKLFIANG
-TAEQ----------NQTYCSKEK--SG-----------QGKRTDLDTTRANLLAGGT------LRDVVL-ATSYQSVKM
AEQILKYHEKKRDWK------------------PIVKWFWGATGTGKSLTAYDELD-----KYTAMSTGKWWDGYDAH--
-SCVIIDDMRRDFCKFH--ELLRYLDRYP---TIVETKGG-TRQFLAKQIIITSCYAPEDMYETR---------EDIQQL
LRRIDEVREF-
>CRESSV3_KT149403
RAWCFTLNNPTDRDKRA---LIALAN--TLEHAPTTGTPHYQGYMVFHDGKSFSA----LHKAMP--R----AHFEPARG
-TAQQ----------NYDYCTKAPQ-------------QGERVDISQVRELLRSGVT------YRELVD-DVNYQVLRI
AQEWLRVHEPARDFK------------------PEVRWFYGEPGAGKTRAALEWLE-----SFTVETPAKYWDGYDGH--
-NCVLFDDLRADQCGFV--RMLRLLDRYA---MRVEVKGG-SKQLRARYIAITAPHRPEEIYRTSS--------ENIVQL
TRRISSVTHV-
>CRESSV3_KT149409
RDWCFTVNNYTYDDYKV---CQELSV---MKEVAETGTKHLQCYVVYFENAKSFSK----MKKLLP--Q----AHIEKAGG
-TPQD----------ASNYCKKGEQPKNE--------QPSGTRNDLKEIREMLAQGKG------MRDVIE-ATSYQSMKS
AELILKYKERKRNWK------------------PKVIWLWGRSGIGKTRAVYEECP------RKSNNSGKWWDGYDAH--
-ENVLIDDVKDTSEEYS--MLLEILDRYD---VRVQTKGA-TREFLAKTIYLTSLEDPRIMYRRFP-------EE-GYEL
YRRIDEIRHV-
>CRESSV4_AHH31482
NNYTEKDVT---SITKLTFSNCV-FAKVGKEVGD-SGTPHLQGFIHLRKRLRLAC----VKKLVGDR-A----HCEVARG
TDKDNE-----------SYCGKDA--DITELQ----E--WKAYCRHSKVIQELSAAFVKNKNIADQAAQMSGKPLRQWQK
ELKDAPNDRE------------------------IMWYCD-SVNTGKTWFSKYLVALSA------D--------KYAYN-
GERVVVFDLSRS-----DHFNYEVIESIK-NGLMFSPKYTSCTKMPI--PHVIVFANW---MPDE-----------SKL
ADRWNIQS---
>CRESSV4_KX388505
HNYTDENVE---YLK--PADKAD-FIIFGFELTK-EGIPHLQGYVEFGTALVRNT----VKARLDPS-S----NVSNAQK
TREANI-----------NYCSKIESKQDISEI----EAFPEYAIKYRFAIKDMIETAKAKQVRADFEEQYESAELRPWQS
KLVDEPNDRK------------------------IIWIWE-NQNIGKSWLSCYLVAKTK------D--------AHAYN-
GEGIVVFDFSRT-----ERLNYQILESIK-NKVLFSPKYNSCSKYKS--PHVICLANW---PPNR-----------ATM
QDRWDVRH---
>CRESSV4_YP_009021888
NNYTEDEFN---SIKNFCERVAK-FAVMGREIGS-GGVPHLQGFISLQNKVRLTG----IKAIVGQR-A----HVEPAKG
TDQKNL-----------EYCTKSCGVSISELL----DTLAPAYIVHKRAIEECASDIAGAQQFQAERARYATTRWKPFQF
NILKIPHPRA------------------------VHWFWD-AENTGKTFIAKYLVLLSA------D--------KYAYK-
GQRIVIFDFSRS-----DHINYEVMESVK-NGIVFSPKYESGMKVRT--PHMICFANE---RPDT-----------SNM
MDRWEIHE---
>CRESSV4_YP_009163936
NNYTDADCE---ALKKLTTDTCS-RAIVGKEKGE-NGTPHLQGFVSLKTRKRLSA----MKTFLSPR-Y----HFEQAKG
TDEQNT-----------EYCSKEG--DKEALL----EKMLGAYMKYKRSITEMVSEVKSEQETKRLKQDLDNATLRPWQR
ELKEYPHDRH------------------------IIWYID-GQNNGKSWFAKYCVAETA------D--------THAYN-
GERVVIFDLSRT-----GHFNYGALESIK-NGCVFSSKYDSRQKMPI--PHVIVFANW---GPDR-----------SKL
EDRWVTKT---

>CRESSV4_YP_009237559
NNWTLADYD---KVLFTTKFEPV-FLICGKERGE-LLTDHLQGFVHLKSKQRLTF----LKANLSER-A----HFECAKG
SDEDNR-----------DYCSKED--EREPFL----EEFAVPVLRHYASFEKLAASVRRLNALEQLQSEYKEVRLKVWQQ
AIINTPHPRT-----------------------IHWYWE-ATGTGKTWMSKYLVACSN------D--------KYAYA-
GEKVVIFDFTRS-----EHINYEIIESIK-NGCYFNCKYESGMRIPT--PHVFCFSNS---PPDL-----------SKM
ADRWHISQ---
>CRESSV5_JX904231
NNYTDDEEQ---CIGFSENDH-LLYMIVGREKGD-SGTPHLQGYLSLNRRFTLSQ----IKGWLG-SRV----HLEVARG
SPQQNR-----------DYCT--KEGTMHEIA----REWPGLFARYSDRLRTIAEASLPPIDLLGDSYELR-----PWQA
ELASSPDPRE-----------------------VMFFVD-SDGKGKSYFCQWLLSKRD------D--------AYAIDE
TKKIFVFDIPRT-----EYLQYSVLEMLK-DRMVFSAKYQSATKIQS--PHVIVFSNE---YPDM-----------SKM
DDRYNIKE---
>CRESSV5_KJ547646
NNPTQDERN---VLALGD-QPTTQYLIYD-EVGA-SGTPHLQGHVSFVQRYRFNQ----VKNWVSPR-A----HLELVR-
LLRRHI-----------EYCK--KDGHSPELR----EKHPNVMARYPHFANSIIRDLFPQSAPPDLPLRA-------WQQ
RVVELPDPRK-----------------------VYFIVD-RQNAGKTSLAKLLHRTVA------D--------AYLYKI
TTKILILDVPRS-----ELLQYSFIEMVK-DGLLMSTKYESVMKTP---PHILVMMNA---DPDH-----------TAL
TDRYHYII---
>CRESSV5_KJ547650
NNYTDEDIH---KLKALLLQPLVSSCIYQQESAT-PGTPHLQGFISFKTKQSFKF----TKNLVSDR-A----HVEVAKG
TPQQNR-----------IYCSKAKDRKINDIL----ENHASVAARYPRYVREYIDLYVNPPEVPDHPLYL-------WQE
TLTKISDDRQ-----------------------IIFVVD-EVNQGKTWFAKKYCRAKA------D--------AYALNT
DLRVLFLNVTRQ-----EHLQYSFLEAVK-DGSVWSPKYESRTKHQI--PHIVVMMNQ---DPDF-----------QLL
KDRYHTIY---
>CRESSV5_KJ641738
NNYSDDDIE---KLSLVPTVCS--YLIYGKETCP-TTTPHLQGYLHFVKRQTLSG----AKALLGNQSY----HLEASKG
TPEQAA-----------AYCK--KEGSDRDLL----DKAPHLWARNRPALLKMAQLLCPPHRLVEGDPRE-------WQE
RLSSLPNDRT-----------------------IYFVVD-PPNNGKTWFQKWYLSKRD------D--------AYSVDD
TKSVFMINVPRG-----EFLQYSILEMIK-DRVVHSPKYESTTKFKK--THVIVFSNE---YPLL-----------SKM
QDRPVFIN---
>CRESSV5_KM874354
NNYVQADID---RLAFGETDDCT-YLVFGKEVGE-SGTPHLQGFAIFPRKLRLRA----VKSHIGNG-A----HLECARG
SPLQAS-----------DYCK--KDGSRALIA----ATWPDLYVRYHTKLFELVGVLAPKPILQEG--DPN-----AWQT
TLIEAPDDRK-----------------------IYFYVD-ETGSGKSWLTRYLMTSRD------D--------AHAIDV
TKKVFLFNVPRT-----EFLQYSILESLK-DRMVMSPKYNSMMKVSV--PHVVVFSNE---EPDR-----------TKL
ADRFAVTH---
>CRESSV5_KP153451
NNYSDADVE---RLKC---KDGIEVHVFK-EVGD-SGTPHLQGFIKFQSRKRAMF----VKQLIGGN-A----HVEYAK-
FPDAAI-----------EYCK--KDQYDSELR----EEFPKLYCRAARFMKEYRDRCIEPPRL--HPLYE-------WQS
KLNSEPDDRS-----------------------ITFIID-ELNSGKSWYATYYKWNVA------D--------AYELNE
RVRVVFMDCARAG----EYLQYDFLEEIK-DGMVFSPKYESRTKYSN--CHVVVLMNE---QPDM-----------TKL
QDRYVIVR---
>CRESSV5_KR528545
NNPTDADAS---AIALGDGPLTR-YLVVGREIGE-SGTRHLQGFVIFHQVQSRAA----VSGYIAR--A----HLEPARA
SSVQAS-----------DYCK--KDGTAHECA----VEQPAAYLKYP-RLISLFQARAPPPDGREG--ERR-----PWQH
ELEDEENDRR-----------------------VTFIVD-QVGAGKTWFQQWFLSKRD------D--------AHTIDE
TKEVFFFAVPRG-----EFLRYEILEMLK-DRMVFSPKYASRMKFVV--PHVIVFSNE---LPDM-----------NKM
LDRYYIKE---
>CRESSV5_KR528547

NNPTGDEIS---KELFLSTPG-VIYHVFGKETGD-SGTFHLQGYVIFSQRKRLGQ----LREIFP--RG----HFEVSRG
SPHQAS-----------DYCK--KEGLERDLI----LEFPNLVG----ASYNISHDLHPQKRVANGSGKP----------
---TPPRQPA-----------------------IEFIVD-PENSGKTWFCQYMITQRD------D--------TFAIDE
TRTIFLFDVPRG-----EFLQYNVLEQIK-DRLIFSPKYSSTLKISN--VHCVVFSNE---EPDV-----------SQL
SDRLRVVR---
>CRESSV5_KR528551
NNPTQQESQ---TLRAIEQTS---YAVIGREVGE-SGTPHLQCFFIFNNRLRLRQ----VKAVPGLQRA----HLEPARG
TSAQAA-----------NYCK--KDGTERDIL----DHHPSILR-YPHFIEVCHRQYGRRPTLVEGQLRN-------WQL
ELSNMPDDRK-----------------------ICFVVD-EENKGKSWLTAYWYSNRD------D--------TYAIDV
SKRVFVFDINRG-----EYFQYSVVESLK-NRMIMSNKYKSVTKIHK--VHVIVFCNE---EPDR-----------TAM
RDRYQMKR---
>CRESSV5_KR528553
NNYSGADEQ---LLELSRSTDVV-TLIYGREVAPGTGTNHLQGMIIFATRKRLRQ----IRNYPPFQRA----HLEVMRS
TPARAR-----------EYCI--KDGTQEEVA----QLQPIAMLRYR-NFMDLARLRAPQPQLIRNGVARE-----GWQM
DLEHAAPDRH-----------------------ILFYVD-PAGNGKTWFQQYLLTKFA------D--------AYAVDE
SKDIFFFNVPRG-----EFFQYRLAESLK-DRLVFSTKYASGMKIKV--PHVVVFCNE---APDM-----------TKL
ADRIIIHD---
>CRESSV5_KR528554
NNWTAAEQQ---ALISSDNFD---YLCFGRERGD-NNTPHLQGYVILKTKLRLNN----VKALPGFRRC----HLEVSRG
TPQQAA-----------DYCK--KDGDDHEIA----EEYPSLWGRYRSACESFRQLFGKPIEVVDPTFEPR-----VWQQ
RIIDIPDPRK-----------------------VYFVVD-ENNTGKSYLSAYLISKRD------D--------AHAINP
RRSIFLFDVPRG-----EYLQYTIFEQLK-NRTVFSPKYNSITKIKL--PHVIIFSNE---SPDR-----------NKM
HDRYHVTH---
>CRESSV5_KR528556
NNYTPAELT---AIVSAGNFD---YLCFGRERGN-NNTPHLQGYLILKEKKRFSY----VRQLAGLERA----HWESPRS
TPKQAS-----------DYCK--KDGTDREVA----EEFPSLWGRYRSACISFLDLFSPHPTLVQGTLRP-------WQE
DLNTRPNDRD-----------------------VMFVVD-ENNSGKSWFIRYLMTERD------D--------AHAIDP
AKKIFFFDVPRG-----EFMQYAVLEQLK-NRLVFSPKYESRMKVHI--PHVVVFCNE---EPDR-----------TKL
RDRFRVTH---
>CRESSV5_KR528561
NNPTVQDRE---RLDL--ADSCN-YLVYGNEIGS-SGTPHLQGFVIFPKTKRFNA----AKIAIGNT-A----HVECARG
SSVQAA-----------TYCK--KDGSKKELV----LAFPGFYARYRKACFEYAEALTPPPILTQS--EPR-----GWQT
RVDGIANDRT-----------------------IHFVVD-PENAGKTWFCSYALTKRD------D--------AYAIST
EKSIFLMDVPRN-----TFLQYSVLEMLK-DRMIFSPKYESSFKIYV--PHVIVFSNE---QPDT-----------SAL
ADRINIIN---
>CRESSV5_KR528562
NNPTPAEEE---SLFHGTPPCDFKYLVFGRETGE-SGTPHLQGYFELVKKLRIHQ----IKGTLGFERS----HLEVARG
TSLQAS-----------DYCK--KENTIKDVW----DVFPTLAARYNRAVMECIDLFGKKPVLVEGDLRL-------WQL
RLDGIADDRR-----------------------VIFVID-PENKGKSWLVSYWLSTRD------D--------AYAVDI
STCLFVFDIPRG-----QYIQYGIFEQLK-NRVVFSNKYSSQTKINT--PHVFVFANE---LPDM-----------NAL
ADRYKVIN---
>CRESSV5_KT945163
NNWTEPVFQ---QSLLGDEANVK-FGIIGKEVGE-QGTPHLQGFLILHRQQRLSW----VRRHFPDG-C----HWSIARS
DSETNR-----------TYCK--KDGSSPEIA----KTHPKMYLRYP-RSVRLAKRRCALFPVQEG--DLN-----DWQR
ELEEKPDDRT-----------------------VLFYVD-PDGKGKTWFVRRYLTLIK------D--------AYAVDT
NARVFFVNVARS-----EFLPYRFLEMLK-DRMVGSSKYESEMKIHN--VHVVVFSNE---YPDE-----------TKM
ADRYDINT---
>CRESSV6_AJD07486
QAKSFFLTYPQCSA-SKQDLKNFLDTKGRERHED--GNFHLHALVTYEKKINVKR-----TTFFDLNG--FHPNIQAAKN

LPALK------------NYISK---EDIEEE------------NYFEICRKRKVSIISRVPFMYANQAFLKIL-KDASIG
EY-------VIGTITSTVLQNL------QLPTDMTSLWVKGPSGIGKTTWALT-VSNAL-----FVRHLDTLREFRNG-Y
HKTIIFDDMSFQHLPRT--AQIELVDRYH--PQQIHIRYAVVNIPPNIPKIFL-SNDSIFY-------DEAIFRRLTLVN
LETDQ------
>CRESSV6_KM510189
SAKRYLLTYAQCPE-DPDDIFRQLDRKRAELHAD--GNPHIHIAVEFAKKLNSVD-----TKYFDYES--YHPNISPAQS
WPKCI------------NYCRGKNKTLTDKE------------LWLQWLFE------HNVQPTLGLEVWRSLHAPPAFET
RE-------IAATFDQRLLYLQ------LPDDFTKSVVIIGPSGSGKTVWAFNRMFEGL-----SVGDIDDLKLFRPE-Q
HSCIVFDEVRFGRFKLE--TQIALCDTAR--SRTIRCRHGNGFIAANTPRVFTCTGTLPFQ-------DYQIERRIHIIN
LYDLWPPTF--
>CRESSV6_KM598390
AARNVFLTYSHQEG-NMQELFQRLQEIKPEQHGD--GTDHFHAVLCFKRKLDTVN-----PRLFDFNG--WHPKIESPRS
VAASI------------TYVKK----DGDRA------------EWEEYCIG------EHISFAYCESIYNRTHPRQRIYE
GE-------NDGELSLALERFS------WGFPESQSLILVGPSGCGKTLWAKR-NAPCL-----FISHIDRLTEFDER-V
HKSIIFDDMTFNHYPLQ--AQIHIVDQYD--DRDIHVRYKTAFIPKKTPKVFT-CNEEPFR-------HETIKRRSKTYN
II---------
>CRESSV6_KM874358
QCRRLFLTYPHCTA-SIQEVYDLIHSKKEEPHSD--GTPHIHAAVMFAKKVNICN-----ARLFDIND--HHCNMESIKN
WPATL------------NYCKKG--DNWESN------------DFWEYCR------LKKVPFAYASNAMRK---KKSITD
PY-------IIREDLGAYTITE------DSPATLASTVLVGPSGIGKTSWAIR-ESESL-----LVTHMDALRKL-NG-S
HKSIIFDDMSFTHIPRE--AQIHLVDRFQ--PRQVHVRYGTADIPAGIQKIFT-ANQYPFA-------DPAIDRRVHNKN
LY---------
>CRESSV6_KP005454
SAKKFLLTYSQCCE-DPEDIFSRIDALRKELHQD--GNPHIHIALEFETKLNTTN-----CRYFDYKN--FHPNVTVADS
WPKCI------------NYCRGKNKTLIEQR------------AWTQWCYE------HDISPLWKSDVWRQLRTPVGRDE
DA-------RPTTIDTRFAFMV------LPEHFKQSVVLLGPSNCGKTTWAVNHMVEIL-----IVNEIDDLRRLDAT--
HKGIVFDEIRFTKWPLH--SQIALVDNAL--GRSIHCRNINAWIPKGTPKLFTCTEKFPFY-------NYQVYRRLNIIN
LYELWVREL--
>CRESSV6_KP153501
NGKQFFLTYPQCQH-SAAELAAFLTSVATEKHED--GTPHLHACIQYVETLRG-G-----VRLLDFNG--HHPNKQDPRK
FEACK------------QYCRK----DGNKA------------LWFDWCIS------KRITHGYAEWYWNSTR--EHDIN
ET-------VAGQMCESLAGYA------FNRDQHRVIVLKGESGCGKTTWAKT-HAPAL-----FVSHIDSLKRFQPG-F
HKTIIFDDVDFNHYPRT--GQIHIVDWEN--PRAIHVRYGTVEIPAGTFKIFT-CNFDPLT-------DDAIRRRVKVVN
VN---------
>CRESSV6_KT149395
NAKNFSLTYSNVEQWDKESLLRHLESLGDEQHQD--GSTHFHAWVQFPRKRDIRA-----SNFFDWQG--
CHPNVQATNN
LRAWK------------TYICK----DGDKH------------EWVNYCIG------KKIGFQYMEYIWKDCH-MDRDIE
DP-------PEAVMCAALEQFI------YPSLDRSPLVLVGDTGCGKTTWAIK-NAPIC-----FVSHMDQLKHFDPQ-Y
HKSIVFDDMDFKHLPRE--SQIHILDMTT--PRAIHRRYGVTVIPAGTKKIFT-ANSNPFE-------DPAIIRR-RTLR
IVGL-------
>CRESSV6_KT732829
NATRFLLTYPQSTFDVQLVWDFFNSLAIKEHHQD--GSEHVHVAIEFERAVNTTR-----VSIFDFGG--RHPNIQSARN
WAACV------------NYCRKEGCTAEDIR------------EWFEYCIA------EGIGYAYANAIWNQIHSVRPYFE
N--------FSGIVSGPTLSHL------RWDPEWHTIFLCGPSGLGKSSWALA-NAPFL-----FVTDIDDLGFFDPE-V
HKALVFDEIRCTKWPLT--SQIKLATWDT--PVSIRIRYKIAHLPAHVPKIFTSCDWFPMG-------DEQIRRRIKAFN
LYDGRSTES--
>CRESSV6Wastewater_AUM61624
NSQQLFLTYSQCPI-NLELILNHLKSLVEEKHQD--GNLHIHCYLLLKSKLNLKN-----PRKFDYQE--YHPKVEGCRS

YKNVI------------KYVTKDGNYIVE--------------EGMKILA------KTARDLCIHGNTIRRNLESLVPKR
R--------KIVFALSDFRIDF------EWDRTK-TLILWGPTNTGKSSLAKALLPDSL-----FVSHIDRLRDYNSK-S
YTGIIFDDMSFKHIPRD--GQLHLVDNYD--DRDIHCRYAPAFIPAGTPKIITSNNPPSE-IL---LLDPAIARRVQIVN
I------EQVW
>CRESSV6Wastewater_AUM61713
NSQQFFLTYPQCPL-DPKVVLDHILIEEPEQHKD--GNMHIHAYLLFAKKLNVRN-----EKKFDIVT--YHPNMQGVRS
WKNVV------------KYVTKDGNYLVS-------------EALSVLS------KTVRDLVIHGNAIQKNLRSLAVKR
K--------APEYSIEDFRVDF------VWDRRR-TLVMWGPTDTGKTSLAKALLPTSL-----FIRHIDRLKEYDEE-E
FEGLILDDMSFKHWPRE--AQIHLVDIEN--DSHINVKHGMAVIPAGTPRIITTNNMPAE-IL---LDDGAIARRIQIVH
I------ESVK
>CRESSV6Wastewater_AUM61719
NTQTLFLTYPKCKM-TPEQALEQLQLIVKEQHMD--GTMHLHAYLRLDHKMNIKD-----ERKFDLEL--
YHPNMQGARS
PKAVI------------KYVCKDGKYVFE--------------EGMKVLS------KTFRDLAIHGEAIERNMRARKKQK
T--------EPKFSLDLFKVSF------EWDKTK-TLVLWGPTNTGKTSLAKALLPNAL-----FIRHIDALKEYKTG-Q
YTGLIFDDMSFKHWPRE--AQIHLLDTDN--VSQINVKHSIAIIPEGTPRIITTNMMPAE-ILALNIEECALSRRVQLEW
I------ERVY
>CRESSV6Wastewater_AUM61738
NTRTLFLTYPKCNL-TVELLLEKLEAP-TEEHQD--GTPHLHCYLKLSRKVNIKD-----PSKLDLKE--YHGNYQGCRS
PKCVL------------KYVTKGGKYIGA--------------EAVNLLAP-----KGARDLLLYGDLIQKSMAKLKKAK
SP-------QINFKLEDFSLDW------TWDVTK-TLVLTGKTNLGKTTLSHCLIQEAL-----LIRHMDRVKEYDSN-L
WGGIIFDDMSFKHLHRE--AQIALTDTAF--DTDIHVRYSVATLPRETPRIITTNLDPYL-ILD---YDEAIRRRCQLVE
V------SN--
>CRESSV6Wastewater_AUM62043
NSQQLFLTYSQCPI-DLELILNHLKSLVEEKHQD--GNLHIHCYLLLKSKLNLKN-----PRKFDYQE--YHPKIEGCRS
YKNVI------------KYVTKDGNYIVE--------------EGMKILA------KTARDLCIHGNTIRRNLESLVPKR
R--------KIVFPLSDFRIDF------EWDRTK-TLILWGPTNTGKSSLAKALLPDSL-----FVSHIDRLRDYNSK-N
YTGIIFDDMSFKHIPRD--GQLHLVDNYD--DRDIHCRYAPAFIPAGTPKIITSNNPPSE-IL---LLDPAIARRVQIVN
I------EQVW
>GasCSVlike_YP_007517186
LTLN----NPTPEECHHLLREVFKRGSE---VG-KKGTPHLQGFVHLKNAKTLTG----LKKFLGS------RYHVKQAN
GTDYE----------NWQYIGL----ETPTE--------EGDPDAWDSILEMIEGG--FNNRDIVRKWPSIAIRCQSAID
RYRVEYEWGECRAWRD-----------------VEVEYIGGPTGTGKTRGVLYHAD-------GNGK---PFDKYDGE--
-G-TIVFEEFRSQ--YTCRDMLNWIDGHP---LLLPARYA-DRMAKFTKVIILSNWRFAEQYRTVAED----SPETYKAW
LRRVGTI----
>GasCSVlike_YP_009126903
LTLN----NPTDAEIASLLQETLKRGLE---VG-AEGTPHLQGFVHLKNAKTGTA----LKKMLGS------RWHWGKAN
GTDFE----------NWAYIME----DKPAE--------EGEPDAWDSILMMIEAG--EDDRAIVRKWPSIAIRCQSAIA
KYRASFEWAECRAWRE-----------------VEVEYISGPTGCGKTREALYLPD-------GNGK---PFDMYDGE--
-G-TIVFEEFRSQ--YTCRDMLNWVDGHP---LMLPARYA-DRMAKFTKVIILSNWRFEEQYRTVQAD----SPETYKAW
LRRVSTI----
>Gemini_AAB87607
NAKNIFATYPRCSLPKEEALELLRQIPTAELHEDGE--PHLHVLLQFEGKLQITN-----PRLFDLVSGNFHPNIQGAKS
SSDVK------------SYIEKD----GDTI----------PTKALNIVKELAPKDFVLHYHNIKSNLDRIFSKPLEPYS
CPFRLNSFNNVPQIMKDWASIN-VVDAAARPDRPISIVIEGESRTGKTMWARSLGAHNY-----LCGHLDLSPKVYSN-N
AWYNVIDDVDPHYL--K--HFKEFMGAQH--
DWQSNTKYGKPIQIKGGIPTIFLCNPGPNSSYKSFLDEDKNKDLKQWAL
KNAIF------
>Gemini_AAF75542

NAKNYFLTYPQCSLTKEEALSQISALSTPELHEDGS--PHLHVLIQFEGKFKCQN-----NRFFDLTSPSFHPNIQGAKS
STDVK------------AYMEKD----GDVL----------KAAALNILKEKAPKDFVLQFHNLNSNLDRIFTPPIEKTF
LLFYLLLSTKFQKNLKNGLLAN-VVSAVGGPLRPMSIVIEGDSRTGKTMWARSLGPHNY-----LCGHLDLSPKVYNN-
D
AWYNVIDDVDPHYL--K--HFKEFMGAQR--
DWQSNTKYGKPVQIKGGIPTIFLCNPGPNSSYKEFLDEEKNKALKNWSL
KNAIF------
>Gemini_AAK73446
RNANTFLTYPKCPENPEIACQMIWELVVREAHKDGS--LHLHALLQTEKPVRISD-----SRFFDING--FHPNIQSAKS
VNRVR------------DYILKEP---LAMF----------KEEYLSMIQKELPYDWSTKLQYFEYSANKLFPEIQEEFT
NPHPQSSDLLCNESINDWLQPN-IFQSSDERSRKQSLYIVGPTRTGKSTWARSLGVHNY-----WQNNVDWSS--YNE-D
AIYNIVDDIPFKFC--P--CWKQLVGCQR--DFIVNPKYGKKKKVKKSKPTIILANSD-----EDWMKEMTPGQLEYFEA
NCVI-------
>Gemini_AAL96826
QARNIFLTYPKCDIPKNEALQMLQALSWSEEHSDGS--PHLHCLIQLSGKPNIKN-----ERFFDLTHPRFHPNIHAAKD
ANAVK------------NYITKD----GDCC----------VAEALEIIRAGDPRAFIVSYHNIKANLDRLFKKAPEQWV
PPFPLSSFTNVPDEMQEWADGY-FGGPAARPVRPVSLIVEGDSRTGKTMWARALGPHNY-----LSGHLDFNHRVYSN-
N
VAYNVIDDVSPHYLKLK--HWKELLGAQK--
DWQSNCKYGKPVQIKGGIPSIVLCNPGEGSSYKDFLDKAENASLKNWTI
KNVIF------
>Gemini_AAN76737
SSKNYFLTYPRCSISKEETLSQLLNIETPELHEDGQ--PHLHVLIQFEGKFVCTN-----QRLFDLVSPTFHPNIQGVKS
SSDVK------------AYIDKD----GVTI----------IDAAMTVLKEEQPKDFVVQYHNIRSNLEKIFTPPTEPWV
PPFQLSTFNNVPLIMSDWVNEN-ISDSAARPLRPISIIVEGPSRTGKTIWARSLGPHNY-----LCGHIDLNPRIYSN-H
SWYNVIDDVDPHYL--K--HFKEFMGAQR--
DWQSNCKYGKPIQIKGGIPTIFLCNPGPHSSYKEYLSEEKNKSLNDWAQ
KNAIY------
>Gemini_AAP73446
SARNIFLTYPKCSLSKEEALEQLCRIECPEAHQDGT--MHLHALVQFKGKAQFRN-----
ARHFDLTHPHFHGNVQGAKS
SSDVK------------SYITKN----GDYI----------VQGALNIIKELLPHNYVFQYHNLRCNLERIFAPPIAVYT
SYYKPADFSQVPSVMTDWAENN-VVDPAARPRRPISIVVEGATRTGKTLWARSLGVHNY-----MCGHLDLSPKIFSN-
D
AWYNVIDDVDPHYL--K--HFKEFMGAQM--
DWQSNIKYGKPTQIKGGIPTIFLCNPGPRSSYKEFLDEEQNESLKEWAY
KNAVF------
>Gemini_AAX39336
QAKNVFLTYPKCSISKEHLLPFIQTLSLPELHQNGE--PHLHALIQFEGKITLTN-----NRLFDCVHPSFHPNIQGAKS
SSDVK------------SYLDKD----GDTV----------KSEALNVIRELVPKDFVLQFHNLNSNLDRIFQEPPAPYV
SPFLCSSFDQVPVEIEEWVADN-VIDSAARPWRPNSIVIEGDSRTGKTIWARSLGPHNY-----LCGHLDLSPKVYNN-A
AWYNVIDDVDPHYL--K--HFKEFIGAQR--
DWQSNTKYGKPVQIKGGIPTIFLCNPGPTSSYKEFLDEEKQEALKAWAL
KNAIF------
>Gemini_ABD35287
NRKNFFLTYSQCPISKEEALQQLINTQTPELHEDGN--PHLHALVQFEGKFCLTN-----PRFFDLQSPNYHCFITDAKS
SSDVK------------AYIEKD----GDIL----------KEQALDVVRELAPKDYVLQFHNLNANLERIFMPPPQEYI
SPFNCSSFDQVPEEIEEWAADN-VMSAAARPWRPISIVIEGDSRTGKTMWARSLGPHNY-----LCGHIDLSPRVYSN-E
AWYNIIDDVDPHYV--K--HFKEFMGAQR--

DWQSNTKYGKPVQIKGGIPTIFLCNPGPTSSYKEFLDEEKNIGLKNWAL
KNATF------
>Gemini_ABD67440
NAKNYFLTYPKCPLTKEDALEQLLALSTPELHEDGQ--PHLHVLLQFEGKQQTKN-----
QRFFDLHSRCYHPNIQAAKS
CSDVK------------TYMEKD----GDIL----------KLEALLILKEKAPKDFILQFHNLNCNLSRIFTEPTQAYE
SPFTLESFDKVPSYISSWAERN-VRDPAARPDRPISIVIEGDSRTGKTMWARAIGPHNY-----LCGHLDLNDKTYSN-E
AWYNVIDDVDPHYL--K--HFKEFMGAQR--
DWQSNVKYGKPTKIKGGIPTIFLCNPGPKSSYKSTWMKRTMLRLNSQRM
RNSRIKYY---
>Gemini_ABG90906
SAKNVFLTYPKCSLSKETALELLKIVNCPEKHQDGS--LHLHALIQFKGKAQFKN-----PRHFDLQHPNFHPNFQGAKS
SSDVK------------SYIEKD----GDYI----------AEAALQIIREKLPRDFIFQYHNLRSNLDRIFSPPPSVYS
SPFLSSSFNAVPDIISDWAAEN-VMDSAARPDRPISIVIEGPSRIGKTVWARSLGPHNY-----LCGHLDLSPKVYSN-S
AWYNVIDDVNPQYL--K--HFKEFMGAQK--
DWQSNCKYGKPVQIKGGIPTIFLCNPGEGSSFKLWLDKPEQGALKNWAT
ANAIF------
>Gemini_ACB44970
NAKNYFLTYPKCSLTKEEALSQLQNLETPELHEDGS--PHIHVLIQFEGKFQCKN-----NRFFDLVAPSFHPNIQGAKS
ASDVK------------TYIDKD----GDVL----------KEDALKVLKELAPKDYVLQFHNLMTNLDRIFPSRIEVYR
SPFNVSSFDRVPPELVDWVSSN-LRCSAARPFRPIGLVLEGDSRTGKTMWARSLGPHNY-----LCGHLDLNPRVYSN-D
ALYNVIDDVDPHYL--K--HFKEFMGAQR--
DWQSNTKYGKPIMIKGGIPTIFLCNKGPQSSYKEFLDEEKNAALKQWAL
KNAVF------
>Gemini_ACI06063
YSKNYFVTYPKCSLTKEEALSQLLNLQTPELHEDGT--PHLHVLIQFEGKFKCQN-----MRFFDLVSPNFHPNIQGAKS
SSDVK------------SYIDKD----GDIL----------RSEASQFGNELDRKDFVLQYHNANANQDRIGQDTPVPYP
FPFSSSQSNQGTEEREESAKEN-VVDAAARPLGPQSISTEGDSRTGKTMWCMSLGPHNY-----LCGHLDLSPKVYSN-
D
AWYNVIDGVDPHFL--K--HFKEFMGAQR--
DWQSNTKYGKPVQIKGGIPTIFLCNPGPISSYKEFLEEEKNTALKNWAV
KNAIF------
>Gemini_ACO88014
QGRAFFLTYSQCPREPKDVGEFLTSHSTLEKHQDGN--NHLHAIVCTSERRDIRD-----PRIFDFG--EFHPKIETCRS
VSKSL------------KYIQKEA---GSFY----------IEEALQLVKDNEPRTFWLQHHNLVTNARRIWSEVRADFV
PKYSESSFS-VPRVLSDWVANN--LRADPLPDRPLSLIIEGDSRTGKTAWARSLGSHNY-----LSGHLDLNGAVFDN-E
ASYNVIDDVNPKYL--K--HWKEFLGAQK--DWQSNLKYGKPVLVKGGKPAIVLCNS--
DQSYKSFLDCEENQHLRSWTS
KNAVF------
>Gemini_ACV60535
NAKNYFLTYPHCSLTKKEALSQLKNLETPEFHEDGT--PHLHVLIQFEGKFQCKN-----QRFFDLISPSFHPNIQAAKS
STDVK------------SYMDKD----GDVL----------KASALNILREKAPKDYVLQFHNLNNNLDRILPS-MEVYV
SPFSSSSFDRVPEELEEWAAEN-VVSAVAGPLRPIIIVIEGDSRTGKTMWARSLGPHNY-----LGGHLDLEPKVYNN-D
AWYNVIDDVDPHYL--K--PFKEFMGGQR--
DWQSNTKYGKPVQIKGGNPTIFLCNPGPNSSYKDYLDEDKNSALKYWAL
KNAIF------
>Gemini_ACV83312
NAKNFFLTYPHCSLTKYEALSQLQALQTPELHEDGE--PHLHVLIQFEGKFKCHN-----QRFFDLVSPTFHPNIQGAKS
SSDVK------------TYMEKD----GDFI----------PSTALNILKEEQPRDYVLHLDKIRTHVQRLFAKAPRPWV

SPFQLSSFTNVPDEMQEWADKY-FGGAAARPERPISIIIEGDSRTGKTMWARALGPHNY-----LSGHLDFNSRVYSN-E
VDYNVIDDVTPXYLKLK--HWKELVGAQR--
DWQSNCKYGKPVQIKGGIPSIVLCNPGEGASYKDFLDKHENVSLKTWTL
HNAKF------
>Gemini_ACY79450
QAKNYFLTYPHCSLSKEEVLDQLKKIQTPELHEDGQ--PHIHVLLQFEGKFVCTN-----QRLFDLVSPTFHPNIQGAKS
SSDVK------------SYVDKD----GDTL----------KEAALQIIREKLPEKFIFQYHNLCSNLDRIFSPPPSVYS
SPFSLSSFNNVPDIISDWAAEN-VMDSAARPDRPISIVIEGPSRIGKTVWARSLGPHNY-----LCGHLDLSPKVYSN-S
AWYDVIDDVNPQYL--K--HFKEFMGAQK--
DWQSNCKYGKPVQIKGGIPTIFLCNPGEGSSFKLWLDKPEQEALKNWAV
KNAIF------
>Gemini_ADN84041
NAKNFFLTYPKCSISKEEALGQLLSIPTPELHENGE--PHLHVLIQFEGKYQCTN-----NRFFDLVSTTFHPNIQGAKS
SSDVK------------SYIDKD----GDTL----------AEQALQIIKEHQPQHFVLQYHNLVSNITKIFHKPPEPWV
PPFQLSTFNNVPAIMSEWVNVN-ISDAAARPLRPISIIVEGPSRTGKTIWARSMGPHNY-----LCGHIDLNPKIYSN-D
AWYNVIDDVDPHYL--K--HFKEFMGAQR--
DWQSNCKYGKPIQIKGGIPTIFLCNPGPHSSYKEFLGEEKNTSLHDWAK
KNAIF------
>Gemini_ADW24243
NAKNFFLTYPRCSLSKEAALEQLQNIQTNEFHENGE--PHLHVLLQFEGKFQCRN-----ERFFGLVSETFHPNIQGAKS
SSDVK------------KYMEKD----GDVI----------TTSALNILKEKASRDFIIHLHNIRANLNFLFAPPPTVYE
TPFSIESFNNVPETLTSWAAEN-VVCPAARPFRPISIVVEGESRTGKTMWARSLGRHNY-----LCGHLDLSAKVYSN-D
AWYNVIDDVDPHYL--K--HFKEFMGAQK--
DWQSNVKYGKPTQIKGGIPTIFLCNAGPRSSYKMFLDEENNASLKEWAL
KNAIF------
>Gemini_AEE99005
MPKNYFLTYPNCSLSKEEALSQLKNLATPEFHENGE--PHLHVLIQFEGKYQCKN-----QRFFDLSSPTFHPNIQAAKS
ATDVK------------TYVEKD----GDFI----------KSEALNILKEKAPKDYILQFHNLSSNLDRIFSPPLQVYV
SPFLLLLLIKS-RELKKGSPEK-PVSSAARPWRPISIVIEGDMQTGKTMWARSLGPHNY-----LCGHLDLSPKVYSN-E
AWYNVIDDVDPHYL--K--HFKEFMGAQR--
DWQSNTKYGKPIQIKGGIPTIFLCNPGPTSPFRGNLGRRKKYILEKLGS
QE---------
>Gemini_AER09339
NARNYFLTYPKCSLTKEEALSQLQALATPELHENGE--PHLHVLLQFEGKFQCKN-----NRFFDLVSPSFHPNVQGAKS
SSDVK------------SYVDKD----GDTL----------AAQALQILREEQPRDFVLHLDKVQAHVQKIFAKAPEPWV
PPFPLSSFTNVPDEMQSWADEY-FASAEARPNRPISLIVEGDSRTGKTMWARALGPHNY-----LSGHLDFNSRVYSN-E
AAYNVIDDVAPHYLKLK--HWKELVGAQR--
DWQSNCKYGKPVQIKGGIPSIVLCNPGEGSSYKDFLDKEENSALRSWTL
HNARF------
>Gemini_AEY63664
QAKNIFLTYPRCSLSKEELLSFLVGLSLPELHQNGE--PHLHVLLQFDGKITITD-----NRLFDHVHPSFHPNIQSAKS
STDVK------------SYLDKD----GDTV----------KGEAMSVIKELAPKDFVLHYHNIKSNLDRIFEEPVAPFV
CPFPISSFTLLPPELVEWASTN-VCSSAARPWRPKSIVVEGESRTGKTMWARSLGPHNY-----LCGHLDLSPKIYSN-D
AWYNVIDDVDPHYL--K--HFKEFMGAQR--
DWQSNTKYGKPIQIKGGIPTIFLCNPGPSSSYKEFLEEEKNYALKEWAD
KNAEF------
>Gemini_AF003952
RNANTFLTYPKCPENPEIACQMIWELVVREAHKDGS--LHLHALLQPEKPIRISD-----SRFFDING--FHPNIQSAKS
VNRVR------------DYILKEP---LAVF----------KEEYLSMIQKELPFDWSTKLQYFEYSANKLFPEIQEEFT

NPHPPSSDLLCNESINDWLQPN-IFQSSDERSRKQSLYIVGPTRTGKSTWARSLGVHNY-----WQNNVDWSS--YNE-D
AIYNIVDDIPFKFC--P--CWKQLVGCQR--DFIVNPKYGKKKKVKKSKPTIILANSD-----EDWMKEMTPGQLEYFEA
NCVI-------
>Gemini_AFA26437
YAKNYFLTYPQCSLTKEEALSQFQNLSTPELHEDGS--PHLHVLIQFEGKYKCQN-----NRFFDLVSPTFHPNIQGAKS
SSDVK------------SYMEKD----GDTL----------KSEALRVIKEIAPKDYVLQFHNLNANLDRIFTPPVEVYV
SPFCSSSFDQVPDELEEWAAEN-VVGSAARPLRPISIVIEGDSRTGKTMWARSLGPHNY-----LCRHLDLSPKVYNN-D
AWYNVIDDVDPHYL--K--HFKEFMGAQR--
DWQSNTKYGKPVQIKGGIPTIFLCNPGPNSSYKEFLDDEKHSALKNWAL
KNATF------
>Gemini_AFB81519
YSKNYFITYPKCSLTKEEALSQLLNIQTPELHEDGT--PHLHVLIQFEGKFKCQN-----MRFFDLVSPSFHPNIQGAKS
SSDVK------------SYIEKD----GDIL----------KSEALRVIKELAPKDFVLQFHNLNENLERIFQGPPAPYG
SLFSSSFFEQIQKKLEGGVAEN-VVSAVGRPIRPISLVVEGDSRTGKTMWARSLGPHNY-----LGGHLDLSPRVYSN-D
AWFNLFDDADPHYL--K--HFKEFMGAQK--
DWQSNTKYGKPVQIKGGIPTIFLCNPGPNSSYKEFLDEEKNSALKNWAL
KNAIF------
>Gemini_AFB83419
QAILYVITYPQCSLTKEEALSQIQAINTPELHEDGS--PHLPVLIRFEGKFVCTN-----NKFFDLVSPNFHPNIQGAKS
SSDVK------------AYINKD----GDAF----------KSEALRVLKELAPKIFGLQFHNLNANLTRIFREAPAPYI
FPFSPSSFDQVPEELGIWAIDN-VVDAAARPLRPRSIVIEGDSRTGKTMWARSLGPHNY-----LCGHLDLSPKIYSN-D
AWYNVIDDVDPHFL--K--HFKEFMGAQR--
DWQSNTKYGKPVQIKGGIPTIFLCNPGTNSSYKEFLNEEKNTALKNWAL
KNAIF------
>Gemini_AFD54490
QSKNYFLTYPKCSISKEEALAQLLALDTPELHGNGE--PHLHALLQFEGKFTCTN-----CRFFDLRHPSCHGKYESCKS
SSDAK------------SYIEKD----GDYV----------VEEALQIIKEEQPQHFFLQHHNVVANAYRIFQKAPEPWT
PPFPLSSFINVPEEMKAWADDY-FGSAAARPERPISIIIEGDSRTGKTMWARALGSHNY-----LSGHLDFNSKVYSN-D
VQYNVIDDIAPQYLKLK--HWKELIGAQK--
DWQSNCKYGKPVQIKGGIPCIVLCNPGEGASYKSFLDKEENASLRQWTI
HNAQF------
>Gemini_AFF58888
SAKNYFLTYPHCSVTKDETLSQLRTINTQELHEDGS--PHLHVLIQFEGKFVCTN-----NRLFDLVSPTFHPNIQGAKS
SSDVK------------SYIDKD----GDTL----------KQDALRIIRELAPKDFILQFHNLSSNLDRIFAPEVPVYT
SPFLSSSFNRVPEELEVWVSEN-VKSAAARPLRPKSIVIEGDSRTGKTMWARSLGPHNY-----LCGHLDLSPKVYSN-D
AWYNVIDDVDPHYL--K--HFKEFMGAQR--
DWQSNTKYGKPVQIKGGIPTIFLCNPGPGASYIEFLNEEKQTALKNWAL
KNAEF------
>Gemini_AFH68197
NAKNYFITYPKCSLTKEDALSQLQNLETPEFHENGE--PHLHVLIQFEGKYQCKN-----NRFFDLVSPTFHPNIQGAKS
SSDVK------------SYIAKD----GDIL----------KSEALRVIKELAPKDFVLQFHNLNANLDRIFQEPPAPYV
SPFSSSSFDQVPEELEEWACEN-VVDAAARPHRPQSIVIEGDSRTGKTMWARSLGPHNY-----LCGHLDLSPKVYSN-D
AWYNVIDDVDPHFL--K--HFKEFMRAQR--
NWQSKAMYWKSVQINGRMLPIYLSNPGPNSSYKRILDDGKNPALKIWTV
QNAIF------
>Gemini_AFM38721
ASRNIFLTYPQCSLDKNHVLELLQNLPWTEIHSDGN--PHLHILIQLSGKSNIKD-----ERFFDISHPSFHPNIQAAKD
TNAVK------------NYITKE----GDYC----------MAAAIAIIKAGDPVRWITQGHNIRSNLQFDFAKQKEPWT
PKFPLSSFNNVPEEMQEWADDY-FGDSAARPLRPRSIIIEGDSRTGKTQWARTLGSHNY-----ISGHLDFNSKTYSD-D

VEYNVIDDVAPNYLKMK--HWKELIGAQT--
DWQSNCKYGKPREIKGGLPAIVLCNPGEGTSYKDFLDKEENAPLKRWTL
QNATF------
>Gemini_AFN80601
RARNLFLTYSKCNLTAVFLLEYISSLLKKEAHKDGS--HHLHCIIQCSKYVRTTS-----AKFFDVGE--FHPNVQNPRM
PKKAL------------AYCKKSP---ISDA----------KEDYLSMVKKSFPFDWATRLQQFQYSAESLFPSTPPPYV
DPFGMP-SQDEHPVIGAWLRDE-LYTD-SPAERRRSLYICGPIRTGKTSWARSLGSHNY-----WQHSVDFLH--VVQ-N
AKYNVIDDIPFKFV--P--CWKGLVGSQK--DITVNPKYGKKRLLSNGIPCIILVNED-----EDWLQQMQPNQAEWFNA
NAVV-------
>Gemini_AFN80669
RTKHVFLTYARCPISPEEAGQKIADRLKNEFHADGE--PHLHAFVQLEANFRTTS-----PKYFDLGE--FHPNIQAARQ
PASTL------------KYCMKHP---ESSW----------RDEYLSMVRKSFPFEWAVRLQQFQYSANALFPDPPQTYS
APYASR-DMSDHPVIGEWLQQE-LYTVRSPGVRRRSLYICGPIRTGKTTWARSLGTHHY-----WQHSVNFLE--EWNCQ
AQFNIIDDIPFKFV--P--CWKGLVGSQY--DLTVNPKYGKKKRIPNGIPCIILVNED-----EDWLTSMSTQQVDWFHG
NAVV-------
>Gemini_AFV91331
RAQNIFLTYPRCDLDPKDAGEIIQSKMQSELHSDGE--YHLHGLLQLSRQFSSNN-----PRIFDIGA--HHPNIQSAIS
PKSVR------------DYILKNP---ITQF----------KESYLSMVRKSFPFEWATKLSQFEYSASKLFPEVTPEYK
SPFPTE-SLICNENIQDWVDNT-LYQ-PNRTSRGLSLYICGPTRTGKTSWARSLGVHNY-----WQNNIDFSV--YND-N
ATYNVIDDIPFKFC--P--CWKALAGSQS--DFTVNPKYGKKKRIKGGIPCIILVNED-----EDWLTCMSSSQKTYFES
NVVI-------
>Gemini_AGF41094
QAKNYFITYPKCSLTKEEALSQIQNIQTPELHEDGS--PHLHVLIQFEGKFVCTN-----NRFFDLVSPTFHPNIQGAKS
SSDVK------------SYIDKD----GDTM----------AEAALAIIREELPKDFIFQYHNIKNNLDRIFAPPLEVFI
SPFPSSSFDQVPEELEEWAAEN-VVSAAARPWRPKSIVIEGESRTGKTMWARSLGPHNY-----LCGHLDLSPKVYSN-D
AWYNVIDDVDPHYL--K--HFKEFMGAQR--
DWQSNTKYGKPIQIKGGIPTIFLCNPGPNSSYKEYLDEEKNAALKNWTL
KNAEF------
>Gemini_AGG08895
QARNYFLTYPHCSLTKEEALSQLKNIETPELHEDGS--PHLHVLVQFEGKFVCTN-----NRFFDLVSPTFHPNIQGXKS
SSDVK------------SYMEKD----GDTI----------KEAALSIIREKLPKDYIFQFHNLNSNLDRIFTPPLEVYI
SPFSSSSFDQVPEELECWVSEN-VMDAAARPWRPNSIVIEGDSRTGKTMWARSLGPHNY-----LCGHLDLSPKVYSN-
E
AWYNVIDDVDPHFL--K--HFKEFMGAQR--
DWQSNTKYGKPTQIKGGIPTIFLCNPGPTSSYKEYLDEEKNSALKAWAL
KNATF------
>Gemini_AGH29892
SSKNYFVTYPHCSLTKEEALAQLKLLNTPELHENGE--PHLHVLIQFEGKYQCTN-----NRFFDLVSPSFHPNIQGAKS
SSDVK------------SYIDKD----GDTI----------KEEAMMIIKEKLPEKFLFQYHNLSSNLDRIFKKAPDPWV
PPFPLSSFTNVPDEMQEWADEY-FGGAAARPVRPMSIIVEGDSRTGKTMWARALGSHNY-----LSGHLDFNSRVYSN-
D
VEYNVIDDVTPHYLKLK--HWKELIGAQK--
DWQSNCKYGKPVQIKGGIPSIVLCNPGEGASYKDFLEKEENASLKSWTL
YNAKF------
>Gemini_AGJ03640
NAKNIFLTYPKCPIPKEQMLEILQSINCPEKHQDGS--LHVHALIQFKGKSKFRN-----PRHFDVTSPIFHPNIQGAKS
ASDVK------------AYIDKD----GDVL----------KEDALKVLKELAPKDYVLQFHNLISNLDRIFQPRSEVYV
SPFSISSFDRVPPELVDWAGVN-VVCAAARPFRPISIVIEGDSRTGKTMWARCLGPHNY-----LCGHLDLSPKVFSN-D
AWYNVIDDVDPHYL--K--HFKEFMGAQR--

DWQSNTKYGKPVMIKGGIPTIFLCNKGPNSSYKEYLDEEKNAALKQWAI
KNAVF------
>Gemini_AGK24653
HAKNIFLTYPRCSLSKDEALELLLGVTTPELHEDGQ--PHLHVLLQFEGKLQITN-----PRLFDLVSRTFHPNVQGAKS
SSDVK------------SYIDKD----GDTV----------PTKALQLLKEQAPKDYVLQFHNIKGNLERIFMKVSTPWT
CPYDPRSFNNVPDVMLDWVSVN-VKDPAARPNRPISIVVEGDSRTGKTMWARSLGVHNY-----
LCGHLDLSPKVYSN-N
AWYNVIDDVDPHYL--K--HFKEFMGAQH--
DWQSNTKYGKPIQIKGGIPTIFLCNPGPSSSYKSYLDEERNSSLKQWAL
KNAIF------
>Gemini_AGV02071
NAKNYFLTYPKCSLTKEEALSQFLNLETPELMKMGL--LHLHVLIQFEGKFQCRN-----NRFFDLTSPSFHPNIQGAKS
SSDVK------------AYMEKD----GDIL----------KAQALNILKEKAPRDFLLQFHNLNSNLDRFFTPPVEVFK
SRYLSSSFDQVPEELEEWAAEN-VMDAAARPERPLSLVLEGESRTGKTQWARSLGPHNY-----LCGHLDLSPKEYSN-
D
AWFNIIDDVDPHYL--K--HFKEFMGAQR--
DWQSNTKYGKPVQIKGGIPTIFLCNPGPNASYKEFLDEDKNSALKSWAL
KNATF------
>Gemini_AGV02076
QSKNIFVTYPQCDIPKDEVLQMLQNLQWREEHSDGN--PHLHCLVQLSGKPNIKD-----
VRFFDITHPRFHPNVQPAKN
SDAVK------------NYITKG----GDFC----------ATEALDIIKDGDPKTFIVQYHNIRSNLDRIFKKAPEPWI
PPYQLSSFVNVPDDMQEWADSF-FGDHAARPERPISIIIEGDSRTGKTMWARALGPHNY-----LSGHLDFNSRVYSN-N
AEYNVIDDINPQYLKMK--HWKELIGAQK--
DWQSNCKYGKPVQIKGGIPSIVLCNPGEGSSYKDFLDKEENVALKNWTL
HNAKF------
>Gemini_AHA82274
NAKKYFLTDPKCSLTKGEALCQLQTRETQELHVDGF--RHIQVVIQFEGKFHCKN-----NRCLELVSPSFHPDIQGAKS
SSDVK------------SYMEKD----GDVL----------KSQALNILREKSPTYFVLQFHNLNTNLDRIFQPPSEVYV
SPFSISSFDRVPADLVDWVTSN-VVCAAARPFRPISIVIEGDSRTGKTMWARCLGPHNY-----LCGHFDLRPKVYSN-E
AWYNVIDDVDPHYL--K--HFKEIMGAQR--
DWQSNTKYGKPVQIKGGNPTTFLCNPGPNSSYKEYLDEEKNSALKTWAL
KNATF------
>Gemini_AHL29198
NAKNYFLTYPQCSLTKEEALSQLQNLATPELHDDGS--PHLHVLLQFEGKYQCKN-----PRFFDLVSPTFHPNIQGAKS
SSDVK------------SYIDKD----GDIV----------KSEALNVIRELAPKDYVLQFHNLNANLDRIFAPPLEVFV
CPFLSSSFDQVPEELECWAADN-VRDAAARPWRPISIVVEGASRTGKTMWARSLGPHNY-----LCGHLDLSPKVYSN-
D
AWYNVIDDVDPHYL--K--HFKEFMGAQR--
DWQSNTKYGKPIQIKGGIPTIFLCNPGPTSSYKEYLDEEKNSALRDWAL
KNAEF------
>Gemini_AHM88370
KSANAFLTYPRCLLTPFEAGQHLWEVARPESHQDGT--PHLHVLLQTIRPMSTRD-----PGFFDIQG--YHPNIQASRS
PNKTR------------EYILKSP---ITVY----------KAEYLSEVQKAFPFEWATKLQQFEYSAERLFPTLPSPFV
PPHPPSEDLNCYETIRSWKDEN-IFQGDSRTSRPRSLYIVGPTRTGKTTWARSIDNHNY-----WQNGVDFLK--YRK-S
AKYNVLDDIPFKFC--P--CWKQLVGGQK--DYTVNPKYARRMEVPGGIPSIILVNYD-----EDWLKVMTPAQLEYFYD
NCVV-------
>Gemini_AHM88378
RNANTFLTYSRCNLDPEAVGLHLWELIGHEAHADGE--WHLHALAQSVKPVHTTN-----QGFFDIEG--FHPNIQSAKS

ANKVR------------EYILKNP---VCQW----------REEYLSMLQKALPYEWATKLQYLEYSASKLFPDTVEEYT
NPHPPTTLLREPTTIDNWVQSN-LFQNNTG-TRKLSLYILGPTRTGKSTWARSLGRHNY-----WQNNVDWSC--YDE-D
SVYNVIDDIPFKFC--P--CWKQLIGCQK--DYVVNPKYGKKRRVKTSIPSIILANED-----EDWLKVMSPGQLDYFHQ
NCVV-------
>Gemini_AHM88382
RNANTYLTYPKCPLEPEAIGLTLWSLIAPEAHQDGT--WHCHALAQSVKPVTTRN-----SRFFDIED--HHPNIQSAKS
VDKVR------------AYILKDP---IALW----------KQEYLSRLQNELPYEWATKLQYFEYSANKLFPDIPEPYI
HPHPQTEELHCKETIDDWLKPN-IFQ-QLPSDRKQSLYIVGPTRTGKSTWARSLGRHNY-----WQNNVDWTS--YDE-E
AMYNIVDDIPFKYC--P--CWKQLIGCQK--EYIVNPKYGKRKKVSRSIPTIVLANED-----EDWLKDMTPAQREYFEA
NCVI-------
>Gemini_AHX57826
NAKNYFLTYPQCSLTKEDVLSQIQNLQTPELHENGE--PHLHVLIQFEGKYNCTN-----NRFFDLVSPNFHPNIQGAKS
SSDVK------------SYLDKD----GDVI----------MEESLQIIKEQQPAHYYLQYHNLVANATRIFRKPPEQWI
PPFPLSSFNNVPEVLQEWADNY-FGDPAARPLRSKSIIIEGDSRCGKTMWARALGKHNY-----LAGHLDFNAKCYSD-
D
VDYNVIDDVSPSYLKLK--HWKDLIGAQT--
QWQTNCKYGKPIMIKGGVPSIVLCNPGEGSSYKDFLEKEENASLKRWTL
YNAEF------
>Gemini_AIT39773
DASEIFLTYPRCPVDPMDVGNYLWTMLLTERHADGS--FHLHAFVQLGYNLITAN-----PHHFDYKQ--
YHPNVQPVRS
SKSVR------------DYCLKNP---ISQY----------RASYLGMIKTTFPFEWATKLQAFEYSASKLFPEVEPEYE
TPNWAV-SLRCPEAIADWANHN-VFQ-PNDGTRRKSLYICGPTRTGKTTWARSLGTHNY-----YNNNIDFTN--YRD-R
AFYNVIDDIPFKFC--P--CWKSLVGCQK--DYIVNPKYGKKKKIAGGIPSIIIVNED-----DDWLPKMTAAQRSYFEA
NCEV-------
>Gemini_AIY31184
YAKNYFLTYPNCSLCQEEALSQLKNLETPELHENGE--RHLHVLIQFEGKFQCKN-----QRFFDLVSPNFHPNIQAGKS
STDVK------------TYVDQD----GDLI----------KSEVLNILKENAPNDYILQFHYLSSNLDRIFSPHLEVYI
SPFLSSSFNQVPVELDEWVAEN-VVSTAARPWTPISIVIEGDSRTAKTMWARSLGPLNY-----LCGHLDLSPKVYRN-D
VWYNVIEDVDPHYL--K--HFMVFMGAQR--
DWQSNRKYGKPIQIKGGIPSIFLCNPRPTSTYREYLDEEKNISLKNWAL
KNAIF------
>Gemini_AJM13604
NAKNYFLTYPQCSLSKEETLSLLLAKQIPELHQNGE--PHLHVLLQFEGKYQCTN-----QRFFDLVSPNFHPNIQGAKS
SSDVK------------TYIEKG----GDFI----------KEAAMRLIKERDPKSYFLQYHNISANAEKIFAPKRVPYE
SPYTPASFTNVPEELKLWVAEN-VMGPAARPQRPKSIVIEGSSRTGKTMWARQLGPHNY-----LCGHLDLSNKVYSN-
N
AWYNVIDDVDPHYL--K--HFKEFMGAQH--
NWQSNIKYGKPVQIKGGIPTIFLCNPGPQSSYKEFLDEPKNAGLKQWAL
KNATF------
>Gemini_AKS48121
NAKNYFLTYPRCPLSKEEALSQLLALQTPELHDDGT--PHLHALLQFEGKFQTRN-----QRFFDLVSQTYHPNIQAAKS
ASDVK------------QYVEKD----GDFI----------KSEALTILKEKAPKDYVLQFHNINCNLDRLFAPRVPVYS
SIYSIASFNNVPEGLRAWAATN-VKDTAARPDRPISVVIEGDSRTGKTMWARALGRHNY-----LCGHLDLSAKVYSN-
D
AWYNVIDDVDPHYL--K--HFKEFMGAQK--
DWQSNVKYGKPTHIKRGIPTIFLCNPGPRSSYKEYLDEANNASLKVWAL
KNAEF------
>Gemini_ALF37659

KAKNIFLTYPQCSVTKEDALEQLININTPELHENGE--PHLHALIQFEGKVQIRN-----PRYFDMQHRSFHCNIQGAKS
SSDVK------------SYVSKD----GDHI----------ALEALQIIREKLPEKYIFQYHNLKPNLEAIFLPPPDLYQ
PPFPLSSFTRVPEIIQEWADSY-FGDPAARPYRYNSIIIEGDSRTGKTMWARCLGPHNY-----ITGHLDFSLKTYSD-N
VLYNVFDDVDPNYLKMK--HWKHLIGAQR--
EWQTNLKYGKPRVIKGGIPSIILCNPGEGSSYQDFLNKSENEALRSWTL
QNSVF------
>Gemini_ALR86823
AAKNFFLTYPHCFLSPTEALEQLKQLQIPEHHDDKERTPHLHALLLLEGKFHGQN-----
PRFFDLVSPNYHCNIQGAKS
SSDVK------------TSSQKE----GEYS----------KQEALRVIKELAPKDYVLQYHNLNANLAAIFAPPPEIYQ
PPYTHNQFI-LPQDIQAWVDSN-FTEPATPPERPQSIIIEGPSRTGKTLWARSLGTHNY-----ITGHLDFSARVYHD-E
VEYNVIDDVDPHYLKMK--HWKHLIGAQK--
EWQTNLKYGKPRIIKGGVPSIILCNPGDGASYKDFLDKPENETLKSWTI
QNSAF------
>Gemini_ALV85583
NAKNYFLTYPRCSISKEEALSQLLALETPELHEDGA--PHLHALLQFEGKFQCTN-----CRFFDLRHPQCHGEYKSCKS
SSDAK------------SYIEKG----GDYI----------KDEAMQIIKEKLPEKFLFQYHNLSSNLDRIFSKPPEPWA
SPFPLSSFTNVPDEMKDWVAQY-IGDAAARPVRPVSLIIEGDSRTGKTMWARALGPHNY-----LSGHLDFNSRVYSN-E
VEFNVIDDVSPHYLKLK--HWKELIGAQR--
DWQSNCKYGKPVQIKGGVPSIVLCNPGEGASYKDFLDKAENSALKAWTL
HNATF------
>Gemini_AMK07575
NAKNIFLTYPQCSLSKQIVLEILQNISCPEKHQDGS--LHIHALIQFKGKAKFRN-----ERHFDISHPHFHPNFQGARS
SSDVK------------AYIEKD----GDYV----------KEDALNIIKEKLPKDFIFQYHNLSANLDKIFKPQQQQYV
SPFNVTTFNNVPNELSQWVYQN-VVDAAARPWRPMSIVIEGVSRTGKTLWARSLGKHNY-----
LCGHLDLSPKVYSN-D
GWYNVIDDVDPHYL--K--HFKEFMGAQR--
DWQSNTKYGKPIQIKGGIPTIFLCNPGPSSSYKEYLDEEKNSALKEWAL
KNATF------
>Gemini_AMP46444
NAKNYFLTYPNCPLDKNEALSQLQAKQTPELHESGE--PHLHALVQFQGKYNCTN-----
QRFFDLESPTYHPNIQRAKS
SSDVK------------SYIDKD----GDTT----------AEGAIKIIKERQPWTFILQRHNIVSNLEREFIKPLEPYV
SPFHISTFTNVPTIIQDWASQN-VKSPAARPLRPQSIVIEGDSRTGKTVWARSLGPHNY-----LCGHLDLNNKVYSN-D
AWYNIIDDVDPHYL--K--HFKEFMGAQQ--
NWQSNTKYGKPIQIKGGIPTIFLCNPGPTSSYKEYLDEAKNASLKLWAI
KNATF------
>Gemini_AMW86999
KAKNYFITYRVLSCSKEEALSQLQNLNTPEFHQNGK--PHLHVLIQFEGKFNCTN-----YRLFDLVSPTFHPNIQGARS
SSDVK------------SYVAKD----GDTI----------KEEAMKIIKEKLPEKFLFQYHNLSSNLDRIFSKAPEPWT
PPFPLSSFTAVPDEMQQWADEY-FGGAAARPERPVSIIVEGDSRTGKTMWARSLGPHNY-----LSGHLDFNARVFSN-E
VEYNVIDDVAPQYLKLK--HWKELLGAQK--
DWQSNCKYGKPVQIKGGIPSIVLCNPGEGASYKDFLDKEENSALRNWTN
KNAKF------
>Gemini_APP87725
NAKNIFLTYPQCHISKESALEQLKAFHYPESHQDGQ--PHLHALLQFKGKFQTTN-----QRFFDLVSPSFHPNIQGAKS
SSDVK------------SYIEKD----GDVI----------KDQALQILKEKAPKDYILHYHHLVGNLGRIFKTPPKEYT
PPFSLDSFNNVPDELWDWVWESGLGNAPAGPLRPKSLVLEGDSRTGKTLWARALGKHNY-----
LSGHLDLNDKVFSL-D

ADYNIIDDVDPHYL--K--HFKEFMGAQV--
GWQSNTKYGKPVQVEKTMPSIFLCNPGPNSSYKEFLDEEKNAALKNWAL
KNAIF------
>Gemini_BAF02752
QSKNYFLTYPKCSLTKEEALEQLKSLSTPELHEDGS--PHLHVLVQFEGKYVCTN-----NRFFDLVSPAFHPNIQGAKS
SSDVK------------AYMDKD----GDSI----------KSDALQLIKELAPKDYVLQYHNLSVNFDKIFTTPVDTFV
SPYPSSSFDQVPEELRQWAGEN-VKDAAARPWRPISIVIEGESRTGKTMWARSLGPHNY-----LCGHLDLSPKVYNN-
D
SWYNVIDDVDPHYL--K--HFKEFMGAQR--
NWQSNTKYGKPIQIKGGIPTIFLCNPGPASSYKEYLAEDKNTALRSWAL
KNATF------
>Gemini_CAJ85998
NAKNYFLTYPDCSLAKEETLEKIKALDTPELHQNGN--PHLHVLIQFEGKYQCKN-----QRFFDLVSPTFHPNIQGAKS
SSDVK------------SYINKH----GDTL----------KSEALNVIRELAPKHFVLQFHNLNANLDRIFAPPLEVFI
SPFSSSSFDQVPKELEEWTAEN-VVSAAARPWRPKSIVIEGESRTGKTMWARCLGPHNY-----LCGHLDLSPKVYSN-
D
AWYNVIDDVDPHYL--K--HFKEFMGAQR--
DWQSNTKYGKPIQIKGGIPTIFLCNPGPNSSYKEYLDEDRNAALKAWTL
KNAEF------
>Gemini_CAM91896
NAKNYFITYPQCSITKEEALAQIKSFSYPELHQDGL--PHLHVLIQFEGKFQCTN-----QRFFDLVSQTFHPNIQGAKS
SSDVK------------AYIEKG----GEFL----------KEEALQIIKEKDPKSFFLQYHNLSANADKIFMTPPNPYV
SNFLTSSFDQVPEELEIWVSEN-VMDAAARPWRPMSIVLEGDSRTGKTMWARSLGPHNY-----LCGHLDLSPKVYSN-
D
AWYNVIDDVDPHYL--K--HFKEFMGAQR--
DWQSNTKYGKPIQIKGGIPTIFLCNPGPTSSYKEYLEEDKNAPLKAWAT
KNATF------
>Gemini_CBA18089
NAKNYFLTYPDCSIAKETALEQLINLETPEFHENGK--PHLHALVQFEGKFQCTN-----CRFFDLKHPNAHANIQGAKS
SSDVK------------SYIKKD----GDYI----------KEEAMQIIKEKLPEKFLFQYHNLSTNLDKLFKKAPEPWV
PPFQLSTFTNVPHEMQEWADDY-FGVVAARPDRPISLIVEGDSRTGKTMWARALGPHNY-----LSGHLDFNSRVFSN-E
VEYNVIDDVSPHYLKLK--HWKELIGAQR--
DWQSNCKYGKPVQIKGGIPSIVLCNPGEGASYKEFLDKQENAALRSWTL
HNAKF------
>Gemini_CBH28932
QAKNIFLTYPQCSLTKEEALSQLQAIHLPELHEDGQ--PHLHILLQLEGKIQVTN-----NRLFDLVSPNFHPNIQGAKS
SSDVK------------SYIDKD----GDTV----------KQTALRIIREKLPEKYLFQFHNLNSNLDRIFSKAPEPWV
PPFPISSFINVPEEMQEWADDY-FGSSAARPLRPMSLIVEGDSRTGKTMWARALGPHNY-----LSGHMDFNSRVFSN-E
VEYNVIDDVTPQYLKMK--HWKELIGAQR--
DWQSNCKYGKPVQIKGGIPSIVLCNPGEEVSYKEFLDKEENAALKSWTL
HNAKF------
>Gemini_CBJ17676
NAKNIFLTYPKCPIPKEQMLEILQSINCPEKHQDGS--LHIHALIQFKGKAKFRN-----PRHFDVTHPNFHPNFQGAKS
SSDVK------------SYIEKD----SDYI----------ADAALAIIREKLPKDFIFQYHNLKCNLDRIFTPPVEAYV
SPFSSSSFDQVPQELEEWAAEN-VVSAAARPLRPISIVIEGDSRTGKTMWARSLGPHNY-----LCGHLDLSPKVYNN-D
AWYNVIDDVDPHYL--K--HFKEFMGAQR--
DWQSNTKYGKPVQIKGGIPTIFLCNPGPNSSYKEFLDEEKNAALKNWAL
KNATF------
>Gemini_CDW92215

NAKHYFLTFPKCSLTKEEALEQLLKLDTPELHENGE--PHLHVLLQFEGKFNCQN-----QRFFDLVSPTFHPNVQGAKS
SSDVE------------SYLDKD----GDIL----------KQEALNVLRELAPRDYTLQFHNLNSNLDRIFQAPPEVYI
SSFSCSSFTQVPDELEEWVADN-VRDSAARPWRPKSIVLEGDSRTGKTVWARSLGPHNY-----LCGHLDLSPKVYSN-
S
AWYNVIDDVDPHYL--K--HFKELMGAQR--
DWQSNTKYGKPVQIKGGIPSIFLCNPGQTSSYKEFLDEEKNKALKAWAV
KNATF------
>Gemini_CRI68211
QCKNIFLTYPQCDIPKDEALEMLRSLKWSEEHSDGF--PHLHCLIQLSGKCNIKD-----ARFFDITHPRFHPNVQAAKD
SNAVK------------NYVTKD----GDYY----------AEEALDIIKEDDPKPFIVSYHNIKANIERIFTTPPKPWT
PPYPLSSFNNVPEDMQQWVAEY-FGSSAARPDRPISIVMEGDSRSGKTMWARALGPHNY-----LSGHLDFNYKVYSN-
D
VEYNVIDDLTPQYLKLK--HWKELIGAQR--
DWQSNCKYGKPVQIKGGIPSTVLCNPGEGSRYIAFVNKEENASWIAWTQ
KNVHF------
>Gemini_DQ458791
QSKYVFLTYPKCSSQRDDLFQFLWEKLTPELHQDGT--THYHALLQLDKKPCIRD-----PSFFDFEG--NHPNIQPARN
SKQVL------------DYISKD------------------KEEYLDMIKEEFPHEWATKLQWLEYSANKLFPPQPEPYV
SPFTES-DLRCHEDLHSWRETH-LYHDNRNGIRHPSLYICGPTRTGKTTWARSLGRHNY-----WNGTIDFTN--YDE-H
ATYNIIDDIPFKFV--P--LWKQLIGCQS--DFTVNPKYGKKKKIKGGIPSIILCNPD-----EDWMLSMTSQQKDYFKD
NCVT-------
>Gemini_FJ665283
NAKNYFLTYPQCSITKESAIEQLQNLQTPEIHENGE--PHLHALIQFEGKFQCTN-----CRVFDLKHPTSHANIQSAKS
SSDVK------------SYIEKD----GDYI----------KEEAMQIIKEKLPEKFLFQYHNLSSNLDRIFTKA--PWS
PPFHLSSFTNVPREMQEWADDY-FGGAAARPERPISIIVEGDSRTGKTMWARALGTHNY-----LSGHLDFNSKVFSN-H
AEYNVIDDIAPHYLKLK--HWKELMGAQK--
DWQSNCKYGKPVQIKGGIPSIVLCNPGEGASYKCFLDKEENAALKHWTI
HNAKF------
>Gemini_FJ665634
QGRAFFLTYSQCPREPKDVGEFLTSHSTLEKHQDGN--NHLHAIVCTSERRDIRD-----PRIFDFG--EFHPKIETCRS
VSKSL------------KYIQKEA---GSFY----------IEEALQLVKDNEPRTFWLQHHNLVTNARRIWSEVRAEFV
PKYSENSFS-VPRVLSDWVTNN--LRADPLPDRPLSLIIEGDSRTGKTAWARSLGRHNY-----LSGHLDLNGAVFDN-E
ASYNVIDDVNPKYL--K--HWKEFLGAQK--DWQSNLKYGKPVLVKGGKPAIVLCNS--
DQSYKSFLDCEENHQLRSWTS
KNALF------
>Gemini_FM877473
QAKNVFLTYPKCSIPKEHLLSFIQTLSLPELHQNGE--PHLHALIQFEGKITITN-----NRLFDCVHPSFHPNIQGAKS
SSDVK------------SYLDKD----GDTV----------KSEALNVIRELVPKDFVLQFHNLNSNLDRIFQEPPAPYV
SPFPCSSFDQVPDEIEEWVADN-VRDSAARPWRPNSIVIEGDSRTGKTIWARSLGPHNY-----LCGHLDLSPKVFNN-D
AWYNVIDDVDPHYL--K--HFKEFMGSQR--
DWQSNTKYGKPVQIKGGIPTIFLCNPGPTSSYKEFLDEEKQAALKTWAL
KNAIF------
>Gemini_JX094280
QGKSIFLTYPKCPLTPLFVIDYLYQLLKNENHQDGE--PHLHCLVQLDKRFNTTS-----QRYFDISDPNYHPNCQVPRR
DADVA------------DYIAKG------------------KSEFLGRVQIEQPYVWATQLRNLEYAANSKWPEQPSVYI
PKWTVF--NNVPEPIREWADTN-LFT-------------VSPTKTGKTAWARSMGLHNY-----FCGGVDFSV--WNN-F
ATYTVIDDIPFQFL--P--CKKELLGCQK--DFTVNEKYRKKCRIP----TIVLCNPD-----QSYKAALMGSEMYEWSL
SNVI-------
>Gemini_KC108902

HAKNIFLTYPHCHLSKEDALLQLQTIECPELHENGE--PHLHVLIQFEGKIQLYN-----PRHFDLRDRGCHPNIQGAKS
SSDVK------------SYIEKD----GDYT----------AEAALAVIREKLPKDYIFQFHNLKPNLAAIFNPPPVGYI
PKYNHTQFV-LTDDILDWLESN-FFEESISPDRPKSIIIEGPSRTGKTLWARSLGSHNY-----ITGHLDFSTRIYND-D
VTYNVIDDVDPQYLKMK--HWKHLIGAQK--
EWQTNLKYGKPRIIKGGIPAIILCNPGEGASYQTFLDKPENEALKSWSL
QNSIF------
>Gemini_KT214373
QGKSIFLTYPQCPLIPMFLIDYLYQLLKNEEHQSRE--PHLHCLVQRDKKIDIKD-----PRFFDIKDPNYHPNTQIPRR
DADVA------------DYISKG------------------KSEFLSRCRTEQPYTYATQLRNLEYMASREWPEPPTVYQ
PRWTHF--PSVPESIKQWADEN-IFTDQKP-DRPLTLIIEGPSKTGKTAWARSLGRHNY-----FCGGVDFSF--WDN-H
ASYNVIDDIPFQFL--P--CKKELLGSQR--DFTVNEKYRKKTRVPGGIPTIVLCNPD-----QSYKTALSSSDMYEWE-
----
>Gemini_NP_040557
QAKNIFLTYPQCSLSKEEALEQIQRIQLSELHEDGQ--PHLHVLLQLEGKVQITN-----IRLFDLVSPTFHPNIQRAKS
SSDVK------------SYVDKD----GDTI----------LDQALQILKEEQPKDYFLQHHNLLNNAQKIFQRPPDPWT
PLFPLSSFTNVPEEMQEWADAY-FGDAAARPLRYNSIIVEGDSRTGKTMWARSLGAHNY-----ITGHLDFSPRTYYD-E
VEYNVIDDVDPTYLKMK--HWKHLIGAQK--
EWQTNLKYGKPRVIKGGIPCIILCNPGPESSYQQFLEKPENEALKSWTL
HNSTF------
>Gemini_NP_050017
NAKNYFLTYPHCSLTKEEALSQIQAIETPELHEDGT--PHLHILIQFEGKFQCKN-----PRFFDLTSPTFHPNIQGAKS
STDVK------------EYIAKN----GDVL----------KASALAILKEEAPKDYILQFHNLNSNLDKIFSLRIEM-Y
ISPFLVSFDQVQRNLNEWVAAN-VVKPAR-PLRPISIVVEGDSRTGKTMWARSLGPHNY-----LCGHLDLSPKVYNN-D
PWYNVIDDVDPHYL--K--HFKEFMGAQR--
DWQSNTKYGKPVQIKGGIPTIFLCNPGPHSSYKEFLEEEKNTALKNWAL
KNAIF------
>Gemini_NP_066185
QARNIFLTYPQCTIPKDEMLQLLQNLNWTERHADGT--PHLHCLIQLSGKSNIKD-----CRFFDITHPQFHPNVQSAKD
TNAVK------------NYITKE----GDYC----------VREALDIIKAGDPKTFVTQHHNVKANLEHIFAKPPEPWN
PPYPLSSFTNVPEEMQEWADDY-FGRPVADSHKAKSIIIEGNSRTGKTCWARALGTHNY-----LCGHLDFNMRVYSN-
E
VEYNVIDDVAPTYLKMK--HWKELIGAQR--
DWQSNCKYGKPVQIRGGVPTIILCNPGEGSSFRDFLDKPENDGLRQWTL
YNAIF------
>Gemini_NP_620741
KAKNYFLTYPKCDLTKENALSQITNLQTPELHENGE--PHLHILIQFEGKYNCTN-----QRFFDLVSPTFHPNIQGAKS
SSDVK------------SYIDKD----GDVL----------KSQALDVIKELAPRDYVLHFHNINSNLDKVFQVPPAPYV
SPFLSSSFDQVPDELEHWVSEN-VMDAAARPWRPVSIVIEGDSRTGKTTWARSLGPHNY-----LCGHLDLSQKVYSN-
N
AWYNVIDDVDPHYL--K--HFKEFMGAQR--
DWQSNTKYGKPIQIKGGIPTIFLCNPGPQSSFKEYLDEEKNQALKNWAT
KNAIF------
>Gemini_NP_671468
NAKNFFLTYPQCSLSKEETLSQIKALNTPELHENGQ--PHIHVLIQFEGKYQCTN-----YRFFDLVSPSFHPNVQGAKS
SSDVK------------SYIDKD----GDTL----------AEEALKIIREEQPAHFFLQHHNLVVNASKIFQKPPEPWV
PTFQLSSFTNVPAEMQDWADQY-FGSAAARSERYMSIIVEGDSRTGKTMWARALGPHNY-----LSGHLDFNSQVYSN-
E
VEYNVIDDINPNYLKLK--HWKELIGAQK--
DWQSNCKYGKPVQIKGGIPSIVLCNPGEGSSYKEFLDKEENRALHNWTI

KNALF------
>Gemini_P0C647
RNANTFLTYSKCPLEPEFIGEHLFRLTREETHTDGT--WHCHALLQCIKPCTTRD-----ERYFDIDR--YHGNIQSAKS
TDKVR------------EYILKDP---KDKW----------REEYLSLVQSSLPYDWATKLNYFEYSASRLFPDIAEPYT
NPHPTTEDLHCNETIEDWLKPN-IYQQNAPGERKRSLYICGPTRTGKTSWARSLGRHNY-----WQNNIDWSS--YDE-E
AQYNVVDDIPFKFC--P--CRKRLVGCQK--DYIVNPKYGKRRKVSKSIPTIILANED-----EDWLKDMTPAHVEYFEA
NCDQ-------
>Gemini_Q80GM6
RNVNTFLTYSRCPLEPEAVGLHIWSLIAHETHEDGG--YHIHVLAQSAKPVYTTD-----SGFFDIDG--FHPNIQSAKS
ANKVR------------AYAMKNP---VTYW----------KQEYLSMIQKALPYEWATKLQYFEYSANKLFPDIQEIYT
SPFPQSTALLDPTAINTWLENN-LYQQNSNSNRKLSLYILGPTRTGKSSWARSLGRHNY-----WQNNVDWSS--YDE-D
AEYNIIDDIPFKYC--P--CWKQLIGCQK--DYIVNPKYGKRKKVSKSIPTIVLANED-----EDWLRDMTPAQQDYFNA
NCET-------
>Gemini_YP_115511
TASSFFLTWPRCPINKESALEQIKTLSLPEKHQDGS--PHLHALVQFQKKYRCTN-----CRLFDLSNPNYHCNIQTARS
SSDAK------------SYIEKD----GVFC----------KDEALTIIKELLPKDYVLQYHNLNANLERIFAPPTTVYM
PPFQTTTFNNVPEALTDWVTTN-VADSAARPFRPISIIIEGPSRTGKTLWARSLGPHNY-----LCGHLDLSPKVYSN-N
AWYNVIDDVDPHFL--K--HMKEFMGAQR--
DWQSNCKYGKPIQIKGGIPTIFLCNPGPQSSYKEFFEEEKNKAINDWAK
KNSIY------
>Gemini_YP_619883
QAKNIFLTYPLCSLTKDEALEQLQSIQLTELHENGK--PHLHALVQLEGKVQITN-----ERQFDLVSPTFHPNIQGAKS
SSDVK------------SYIDKD----GDTL----------VQEALQILKEEQPRDFVKDFHNLKGNLEKIFTKAPEPWV
PPFSLSSFNNVPEELQSWADDY-FGGCAARTLRPISIIIEGDSRTGKTMWARSLGIHNY-----LSGHLDFNSRVYSN-D
VMYNVIDDVPPHYLKMK--HWKELIGAQT--
DWQTNCKYGKPIQIKGGIPSIVLCNPGEGASYKYYLDKQENSHLKAWTH
HNAEF------
>Gemini_YP_764516
KAKNYFLTFPKCSLSKEDALGQLKDLQTPELHENGE--PHLHVLIQFEGKYNCTN-----QRFFDLVSPSFHPNIQGAKS
SSDVK------------SYIDKD----GDTL----------ADAAMAIIREKLPKDYIFQYHNLKSNLDRIFQAPPEVYV
SPFSSSSFNNVPEELEEWAAEN-VVGAAARPWRPQSIVVEGDSRTGKTMWARSLGPHNY-----LCGHIDLSPRVYSN-
E
AWYNVIDDVDPHYL--K--HFKEFMGAQR--
DWQSNTKYGKPIQIKGGIPTIFLCNPGPTASYKEYLEEDKNAALKAWAI
KNATF------
>Gemini_YP_001040016
SAKNIFLTYPKCSLSKEIALELLKAIKCPEKHQDGS--LHLHALIQFKGKAQFKN-----PRHFDLQHPTFHPNFQGAKS
SSDVK------------SYIEKD----GDYI----------KDAALQIIREKLPEKFIFQYHNLVSNLDRIFQEPPAPYI
SPFLCSSFNQVPEELEVWAAEN-VMGAAARPWRPISIVIEGDSRTGKTMWARSLGPHNY-----LCGHLDLSPKVYSN-
D
AWYNVIDDVDPHYL--K--HFKEFMGAQR--
DWQSNTKYGKPIQIKGGIPTIFLCNPGPTSSYKEYLDEEKNTPLKDWAF
KNATF------
>Gemini_YP_001285764
SARNYFLTYPHCSLTKEETLSQLSNLVCPELHEDGS--PHLHVLIQFEGKYVCTN-----KRFFDLVSPTFHPNIQGAKS
SSDVK------------SYIDKD----GDTL----------VNAAKQLVKEKQPWTYLLQRHNIVANIEKEFEKPPEPFV
CPFPTTAFDNVPTMMKVWAQEF-VTNSAARPLRPKGIVIEGESRTGKTLWARSLGPHNY-----LCGHLDLNPNVFSN-
D
AWYNVIDDVDPHYL--K--HYKEFMGSQT--

NWQSNRKYGKPVQIKGGIPTIFLCNPGPTSSYTEFLEEEKNAKLKRWSN
YNAIF------
>Gemini_YP_001333687
QGVSFFLTWPKCPVTKESALDQIQALTLPEKHQDGS--PHLHALVQFQKKFICTN-----CRLFDLSHPQFHCHIETARS
SSDAK------------SYIEKD----GVFC----------KDEALNIIKELVPKDYVLQFHNLNQNLERIFAPPVNVFE
PPFPLSSFNNVPAVINQWVNDN-IMDAAARPFRPISIIIEGPSRTGKTLWARSLGRHNY-----LCGHLDLSPKVYSN-E
AWYNVIDDVDPHYL--K--HMKEFMGAQR--
DWQSNCKYGKPIQINGGIPTIFLCNPGPTSSYKEFFEEEKNKAINDWAK
KNVIY------
>Gemini_YP_002224032
SAKNIFLTYPKCPLSKETVLDLLRNISCPEKHEDGS--LHIHALIQFKGKARFRN-----ARHFDLIHPHFHPNIQGAKS
SSDVK------------SYIEKD----GDFI----------AEAALAIS-VKMPRDYIFQYHNLKSNLERIFTPLRVDYV
SPYPLCSFDRVPEELEIWASEN-IVSPAARPFRPISIVLEGDSRIGKTMWARSLGPHNY-----LCGHLDLSPRVYSN-D
VWYNVIDDVDPHYL--K--HFKEFMGAQR--
DWQSNTKYGKPVQVKGGIPTIFLCNPGPHSSYKEYLDEDRNAALKNWAV
KNVVF------
>Gemini_YP_002941855
NCRNFFLTYPKCSLSKEEALSQLLALETPELHEDGQ--PHLHVLIQFEGKYNCQN-----NRFFDLVSTTFHPNIQGAKS
SSDVK------------TYVEKD----GDFI----------VMEALNILREEQPKDFVLQHHNIRSNLERIFQKAPEPFV
PPFPLSSFTLVPEEMQEWADSY-FGDAAARPERAISIIIEGDSRTGKTMWARSLGVHNY-----LSGHLDFNSRVYSN-D
VEYNVIDDISPHYLKMK--HWKELIGAQR--
DWQSNCKYGKPVQIKGGVPSIVLCNPGEGSSYKDFLDKEENASLRNWTL
RNAQF------
>Gemini_YP_003288768
RNANTFLTYSKCSLDPEILGLSLWSKLAPEAHQDGT--WHCHALAQSVRPVTTSD-----PRFFDVNE--YHPNIQSAKS
VDRVR------------EYILKDP---LCQW----------KHEYLSMLQKELPYEWATKLQYFEYSANKLFPEIAEPYT
NPHPPTQDLHCYERIEEWLNFN-VYQVQQEAGRARSLYIVGPTRTGKSTWARSLGRHNY-----WQNNVDWSS--YDE-
E
AVLNVIDDIPFKYC--P--CWKQLVGCQK--NYVVNPKYGKKKKVKRSIPAVILANED-----EDWLRDMTPAQRDYMEA
NCEV-------
>Gemini_YP_003622552
KAKNIFLTYPRCSLTKEEALSQLQSIQCPELHENGE--PHLHVLIQFEGKCQITN-----ERHFDLTSPRFHPNIQGAKS
SSDVK------------TYIDKD----GDTI----------AEAALQIIKEQLPRDYVFQYHNVISNLNKIFTPPTTIYK
SPFKVEQFNNVPEVLSQWASDN-VKASAARPMRPISIVLEGESRTGKTMWARSLGRHNY-----LCGHLDLSAKVYSN-
D
AWYNVIDDVDPHYL--K--HMKEFMGSQR--
DWQSNVKYGKPTQIKGGIPTIFLCNPGPRSSYKEYMDEESNAALKEWAL
KNAIF------
>Gemini_YP_003778178
HAKNIFLTYPHCHLTKEDALLQLQTIQCPERHDNGE--PHLHVLIQFEGKIQLYN-----PRHFDLRDGGCHPNIQGAKS
SSDVK------------SYIEKD----GDYI----------AEAALAVIREKLPKDYIFQFHNLKPNLAAIFNPPPVGYV
PKYNHTQFV-LTDDILDWLESN-FFEESNSPDRPKSIIIEGPSRTGKTLWARSLGSHNY-----ITGHLDFSTKVYND-D
VSYNVIDDVDPHYLKMK--HWKHLIGAQK--
EWQTNLKYGKPRIIKGGIPSIILCNPGDGASYQNFLDKPENEALKSWSL
QNSVF------
>Gemini_YP_003828907
AAKNIFLTYPQCDIPKDEALKMLQELAWTEEHSDGS--PHLHCLIQLSGKSNIKN-----ARFFDLTHPRFHPNIQAAKD
SNAVK------------NYITKE----GDYC----------AEEALAIIKAGDPKTFVVNYHNVKANIERLFQKDEDPWV
PPFQLSSFNNVPQEMKDWVDEN-VTARAAGPVRPRSIIIEGDSRTGKTMWARAIGPHNY-----LSGHLDFNARVYSN-

N
VMYNVIDDVGPQYLKLK--HWKELIGAQR--
DWQSNCKYGKPVKIKGGIPSIVLCNPGDGASYKAFLDKEENASLRAWTL
KNANF------
>Gemini_YP_003915159
RSQHVFLTYPRCPIPPKDAGSFLKKLCKRELHQDGE--PHLHAFLQFDKVFRTTS-----AKYFDFFE--FHPNIQAARN
PEKTL------------EYCQKNP---ADFY----------RDEYLSMIRKAFPFDWAIRLQQFEYSAKALFPEAPIQYQ
PQFVSN-DMSDHPVIGEWLDTE-FFTQEGPHHRRRSLYICGPTRTGKTSWARSLGTHHY-----WQHSVDFLT--EWNKN
AIYNVIDDIPFKFV--P--CWKGLVGSQF--DITVNPKYGKKKTIPNGIPSIILANED-----EDWLQTMSPQQADWFHG
NCVV-------
>Gemini_YP_003966137
SSKNIFLTYPQCDIPKDEVLQLLQNLPWSERHADGT--PHLHCLIQLSGKSNIKD-----CRFFDITHPRYHPNVQAAKD
ANAVH------------NYITKE----GDYC----------VKEALEIIKAGDPKTFVIQHHNVLANLERIFKKPQETWT
PPFPLSSFNNVPDEMQEWADNY-FGPIESRPIRPKSLIIEGDSRTGKTCWARSLGKHNY-----LSGHLDFNPRVYSN-E
VEYNVIDDIAPSYLKLK--HWKELIGAQH--
DWQSNCKYGKPVQIKGGIPSIILCNPGEGSSFKDFLDKDENESLKRWTL
YNAVF------
>Gemini_YP_004089627
RSRNIFLTYSKCHLDPVFMQEHLSSLLRREEHQDGS--YHLHCLVQCKKYVRTKS-----AKFFDVEE--FHPNVQNARM
PHKVL------------AYIKKNP---LCFV----------KEDYLSMVRNTFPFDWATRLQQFQYSAESLFPSVPTPYM
DPFGMP-AQDEHPVIGAWLQAE-LFSR-RPDERRRSLYICGPTRTGKTSWARSLGAHNY-----WQHSVDFLN--LVA-N
ATYNVIDDIPFKFV--P--CWKGLVGCQF--DITVNPKYGKRRMLKNGVPSIILVNED-----EDWLKQMQPSQVGWFET
NCII-------
>Gemini_YP_004958233
NAKNYFLTYPQCSISKEEALQKLISLRTPELHQDGE--PHLHVLIQFEGKYQCTN-----QRFFDLESPTYHPNIQGAKS
SSDVK------------SYIEKG----GDFI----------KDSALQVIKEKDPKNFFLQYHNISANANHIFAPKITPYV
SPYDPNSFDNVPEAMKEWASKN-VMGPAARPDRPLSIVIEGPSRSGKTKWARALGPHNY-----
MCGHIDLSLKVYNN-N
AWYNVIDDVDPHYL--K--HFKEFMGAQH--
NWQSNVKYSKPVQIKGGIPTIFLCNPGPQSSYKEYLEEPKNAQLKIWAE
QNASF------
>Gemini_YP_006273070
RSNNCFLTFPHCNSCPYGMVQHFWDLISTELHQDGT--PHLHALLQTRKQISTND-----PHFFDFDG--HHPHIQAAKN
PTLCR------------DYILKGP---ITFS----------KSDYLSKVRRNFPYDWATKLYNFEYSASKLFPEQQPEYS
NPHGQSVDLYCYETIQDWIDSN-LFQVHYCYMLNTPTYIVGPTRTGKSTWARSLGRHNY-----WQNNVDFTV--YDP-
E
AAYNIIDDIPFKFC--P--CWKQLVAAQR--DFTVNPKYGKKKLIKGGIPSIILVNSD-----EDWLKTMTPEQQEYFEA
NSII-------
>Gemini_YP_006590064
NAKNYFLTYPQCSLSKEAALEQIISLQTPELHDDGQ--PHLHVLLQFEGKFCCTN-----NRLFDLVSPTFHPNIQGAKS
SSDVK------------SYVDKD----GDTI----------LERALQILKEEQPKDFVLHHHNIRSNLERIFAKAPEPWA
PPFHLSSFTLVPQEMQDWVNDY-FGGAAARPERPISIIIEGDSRTGKTMWARALGSHNY-----LSGHLDFNSRVYSN-D
VQYNVIDDIAPQYLKLK--HWKELIGAQK--
DWQSNCKYGKPVQIKGGVPCIILCNPGEGSSYKSFLDKEENAGLKGWTL
HNAKF------
>Gemini_YP_006666523
RSRNVFLTYSKCHLEPAYMLERLSRLLKKEEHKDGS--YHLHCLVQCRKYIRTKS-----AKFFDVEE--FHPNVQNARV
PHKVL------------AYIKKGP---VCFV----------REDYLGMVKKEFPFDWATRLQQFEYSAQALFPCLPPPYV
DPFGMP-SQAEHQVLGAWLREE-LYSQDSPAERRRSLYICGPTRTGKTSWARSLGCHNY-----WQHSVDFLH--VIP-T

ARYNVIDDIPFKFV--P--CWKGLVGAQR--DITVNPKYGKKRLLPNGIPSIILVNED-----EDWPQYMQPSQAAWFED
NCVV-------
>Gemini_YP_006666527
RGKNIFLTYSRCEIDPALITDALWDKFSSELHQDSG--FHVHCLVQLTDQYRSRD-----SSFADLGG--NHPNIQTVRS
ATKVK------------EYILKEP---VSQS----------RDDYLGMVRKSFPFEWATRLAQFEYSASKLFPDITPQYQ
SQYQTT-DLTCHENLLDWYQEN-LQCYIDGAGRRKSLYICGPTRTGKKSWARVLGRHNY-----YNMQVDWAT--YDQ-
E
AQYNVIDDIPFKFC--P--HWKALIGCQK--DFTVNPKYGKKKLIKGGIPTIILVNED-----EDWLADMTPGQVSYFEA
NVQI-------
>Gemini_YP_006666531
RARSAFLTYPKCTLEPRDVVEHLYSKFRKEHHSDGD--YHLHCLFQLDKAFSTND-----SSTFNILD--YHPNIQTAKS
PTNVR------------DYCLKNP---VSKA----------RASYLSMVRKAFPFDWATKLQQFEYSASKLFPDVIPEYT
SPFPTE-NLMCNERITDWLDNT-LYQSAHPRTRKSGLYICGPNRTGKTSWARSLGKHNY-----WQMNLDFAN--YNN-E
AQYNVIDDIPFKFC--P--YWKALVGSQH--EYTVNPKYGKKKLIKGGIPSIILVNED-----DDWMRAMNDGQRSYFEG
NMSI-------
>Gemini_YP_006666535
RAKNIFLTYSKCLLDPQEALRDITHKLRKELHQDGT--FHLHCFVQCKKHVRTTR-----ARFFDLEE--YHPNVQNARM
PHKVL------------AYCKKSP---VSYA----------KEEYLSMVRKTFPFDWATRLQNFEYSAERLFPSTPPPYV
SPFNMP-SQEEHPVLGAWLRAE-LYTQGNPAERRKSLYICGPTRTGKTSWARSLGKHNY-----WQHSVDFLN--IIP-D
AEYNVIDDIPFKFV--P--CWKGLVGAQR--DITVNPKYGKKRLLSNGVPCIILANED-----EDWLQQMQPGQADWFNA
NCEV-------
>Gemini_YP_006905839
NCKNLFLTYPQCSLSKEETLSQLTNLSLPELHQNGE--PHLHVLVQFEGKVRLTN-----CRLFDLVSPTFHPNIQGAKS
SSDVK------------AYLEKD----GDVL----------KSEALRLIKELAPKDYVLQFHNLNSNLERIFTPPVEIYR
SPFMSSSFDQVPDELEQWVADN-VKAAAARPWRPKSIVIEGDSRTGKTMWARSLGPHNY-----LCGHLDISPKVYSN-
D
AWYNIIDDVDPHYL--K--HFKEFMGAQR--
DWQSNTKYGKPIQIKGGIPTIFLCNPGPTSSYKEFLEEEKNSALKRWTL
QNATF------
>Gemini_YP_007250561
SAKNIFLTYPRCSLTKEEALFQLQNISLPELHEDGE--PHLHVLIQLEGKVQIYN-----QKLFDLSSTSFHPNIQGAKS
SSDVK------------SYMEKD----GEFL----------AESALQILKEEQPAHYFLHYHNLVNNANRIFQKAPEPWV
PPFQLSSFNAVPDEMQEWADDY-FGGAAARPERPISIIVEGDSRTGKTMWARSLGPHNY-----LSGHLDFNSRVYSN-E
VEYNVIDDVAPHYLKLK--HWKELIGAQK--
DWQSNCKYGKPVQIKGGIPSIVLCNPGDGGSYKDFLDKEENASLKQWTL
KNAKF------
>Gemini_YP_008411025
NAKNYFLTYPRCSLTKEETIEQLRNLQYPELHENGE--PHLHVLLQFEGKYNCTS-----DRFFDLSHPSFHPNIQRAKS
SSDVK------------AYIGKD----GDTL----------KQEALNIIKELMPKDYVLQYHNLHSNFDRIFTSAIPPYK
SPFLSSSFDQVPDELEEWVSEN-VLPATARPERPISIVVEGDSRTGKTLWARSLGPHNY-----LCGHLDLSPRVFSN-D
AWYNVIDDVDPHYL--K--HFKEFMGAQR--
DWQSNTKYGKPIQVRGGIPTIFLCNPGPTSSYTEFLDEEKNKSLKAWAI
KNARF------
>Gemini_YP_009021763
QSRNIFLTYPRCNLAPELIGSFLLSLLSPELHKDGT--PHIHALAQTDKRVHTYS-----PGFFDVQG--FHPNIQSARS
PQTVL------------SYILKSP---TGTF----------KQEFLNGVKKAFPYDFCARLQNWEYAANKLFDT-PAVYQ
PPFPDS-YFHCHENIHDWVRDN-IYEITP-EARPLSLYICGPTRTGKSTWARSLGRHNY-----WQNNVDFTS--YDV-E
AKYNVIDDIPFKYC--P--CWKALIGGQK--DFTVNPKYGKKKLIKGGIPSIVIVNDD-----EDWMRAMTASQRSYFER
NCVV-------

>Gemini_YP_009026388
KAQNIFLTYPRCDISVDVAARTLLTLCHREHHADGS--NHLHILLQTDKTMYTRN-----PHYFDICG--HHPNIQPAKS
PDNVR------------AYILKDP---ITSF----------KDDYLTRVRNTFPFDWATRLQQFEYSASKLFPEPVREYV
NPFPPSEDLFCREIIDRWVDMD-ITDAFDAAQRRRSLYIVGPTRTGKSTWARSLGRHNY-----WQHMVDFTA--YDT-L
AKYNILDDVPFKFC--P--NWKQLVGCQR--DFIVNPKYAKRKEIPGGIPCIILQNPD-----DDWLPVLSPSQMDYFVN
NCDV-------
>Gemini_YP_009129272
QAKNYFLTYPRCSLSKEEALEQILGLNTPELHEDGE--PHLHVLIQFEGKYTCTN-----QRFFDLVSPTFHPNVQGAKS
ASDVK------------TYIEKG----GEFL----------ASASLQIIKEKDPKTFYTQYHNLSANAAKIFMAPPEPFR
CIYLSSSFNNVPEELDDWVSVN-IVDPAARPRRPKSIFIEGETRTGKTEWARSLGPHNY-----LCGHLDLSPKVFSN-D
ALYNVIDDVDPHYL--K--HFKEFCGSQR--
DWQSNTKYGKPLLVKGGIPTIFLCNPGPTSSYKEWLDEEKNSNLKNWAL
GNAVF------
>Gemini_YP_009226627
QAKNIFLTFPHCTLSKDEALEQLQNLACPELHENGE--PHLHVLIQFEGKVQICN-----PRHFDLRHPSFHPNIQGAKS
SSDVK------------SYIEKD----GDYL----------AEAALAIIREKLPHDFLFKYHNLKPNLAAIFAPPPAVYT
PPFTHTQFN-IPEEIRQWVNNN-FIEEASSPERPQSIIIEGPSRTGKTLWARSLGTHNY-----ITGHLDFSARVYHD-D
VEYNVIDDVDPHYLKMK--HWKHLIGAQK--
EWQTNLKYGKPRIIKGGVPSIILCNPGDGASYKDFLDKSENEALKSWTI
QNSAF------
>Genomo_AGS12486
NFRYVLVTYAQCDL-DPWRVVERFS---SEHHEDGG--LHLHVFADFGRKFRSRK-----ADILDVDG--RHPNLAPIRT
PAKAY------------DYAIKDG----DVV----------RDEFWALVHELDPKAAACSFNALSKYADWRFAEKPPVYE
HDGRI-EFVPGDADGRDDWVSQSGIGL--DPGRVKSLVLFGGTRTGKTTWARSLGKHLY-----
CIGLVSGAECSKGADA
EYAVFDDIRGFGFFH----GYKEWLGAQP--HVSIKQLYREPYYMKWGKPSIWICNTDPRLDAYGP-----ATPDWEWME
GNCHF------
>Genomo_AIF34843
HSRYALLTYSQCDL-SPATVGEFFT---NENHANGG--IHLHCFVDFIRKRRFRA-----PRCFDIEG--RHPNVEPSGT
PERGW------------DYVIKDG----DVV----------RESFWDLVHELDPKSAACSFTQLQKYCDWKFAPSPPVYG
SPDGI-TFVGGETDGRDDWLLQSGIG---NRPRCMSICVYGESRTGKTLWARSLGSHIY-----CVGLVSGDECMKAETV
DYAVFDDVRGIKFFP----SFKEWLGCQA--WVTVKCLYREPKLVKWGKPSIWLSNTDPRDHME--------NSDIDWMN
KNCIF------
>Genomo_AJD07464
NRRYVLLTYSQAEF-NYWAIVDMLS---SELDADGG--THFHVFVDFGRLFSTRK-----TNVFDVDG--HHPNILPVKT
PGEAF------------DYAAKDG----DIV----------GEEFLHFLDQLAPRDLMRGFIQFRSYADWKWAVAPERYV
NPPGV-MFDTGHAEQLSEWLSQANLGSG-VAHSRKSLMLWGPTQYGKTTWARSLGNHIF-----
FGSQFSGKLALDGQDA
EYAVFDDWKGMKALP----GYKDWFGCQW--QISVRKLHHDAKLITWGRPIIWLCNKDPRLMHVAT-----
DDVDWEWMD
DNVIF------
>Genomo_AMH87666
CARYGLFTYSQCDL-NHWAVLDLFS---GELHEDGG--THLHVFADFGRRFRSRS-----SKIFDCEG--RHPNVSASGK
PDEGW------------DYAIKDG----DVV----------RDEFWRLVHELDPKSLVIHFPAISKYCDWKFSPRQVEYV
HPINI-DFVGGEVDGRDHWLAQAGIGL--GTRGMKSLCVYGKSRTGKTLWARSLGRHIY-----
CIGLVSGAECARAEEV
DYAVFDDIRGIKFFH----AYKEWMGAQA--VVSVKLLYRDPKLVRWGKPSIWLSNKDPRCDMS--------QEDVEWLE
DNCIF------
>Genomo_AMH87678

SARYVLLTYPQCTL-DGWAVSDHLS---AENHSDGG--THLHAFVDFGRKKQSRR-----SDFFDVGG--HHPNIAPSGR
PERGY------------DYAIKDG----DVV----------REQFFDLLRQLDPKTLVTRWTELNKYADAAYAPTPEPYV
GSDGK-QFELGMVPELARWGEQL-VA---NDPN-LVL--YGPSRLGKTVWARSLGPHVY-----SMGIVSGKLLLRDPEA
KYAVFDDMRGIGYFH----SWKEWLGAQS--VVTVKELYRDPVQLVWGRPCIWLANRDPRLELGAYFTA-
RFQSDWAWLE
TNCYF------
>Genomo_AMH87693
NRRYVLFTYSQADF-DYWAVVDLLG---DEVHADGG--IHFHVFTDFGRLFSTRK-----VRVFDVGG--KHPNIKPIRT
PAQAY------------DYAIKDG----DVV----------SEEFLHFCDQLAPRDFIRGFTNFRAYSNWKFNPGIPEYD
QPDGA-TYDTSAAPGIDEWLSQ-----------RKSLVLFGPYGCGKTLWARSLARHIY-----FGSSWSGELAFAGDTA
EYAIFDDWAGLKCLP----KYKDWFGAQW--HISVRRLHHDAKLVEWGRPIIWLCNRDPRILSHER-----DDIDWEWME
TACHF------
>Genomo_AMH87702
NAQHFLLTYAHVEELDPFRIVDVLG---AEFYNTGG--FHFHVFCSFERNFRSRK-----ADVFDVDG--YHPNIEPSKN
AAGGF------------DYATKDG----DIV----------AEEFWRLCEELDPKSMVCNFPALSKFAEWRFRPIPVPYA
SPDGV--FDTSGYPIIEEWR-SSVFDE--YSTYLTSLVLFGPTRLGKTTWARSLGSHIY-----FGGLFSAGEAMRCPEA
EYAIMDDIAGIKFFP----RFKDWLGCQA--QFQLKVLYKEPALYDWGKPCIWCYNVDPRAGMS--------VEDIEWLE
GNCVF------
>Genomo_AMH87708
NVRRALLTYSQCTL-DPFAVSDHFS---NERHADGG--LHLHVFVDFGRQFSSRK-----TDVFDVGG--HHPNIAKCRT
PWKAY------------DYAIKDG----DVV----------PEEFWALCHDLAPRDLARSFPSLQKYCDWRYRPVTRPYC
TPDGY-EFDTSRAPELNEWLLQANLG---LGWRPKSLVLYGDTRLGKTVWARSHGPHIY-----
FCGLYSGAEAMKHDGA
DYAVFDDIQGIKFFH----GFKNWLGAQA--EFQVKILYKDPVIIQWGKPSIWLSNSDPRLDLS--------PSDATWLE
GNCIF------
>Genomo_AMH87733
NSRYKLLTYAQCDL-DPFDIVDLLS---GELHSDGG--IHLHVFVDFGRKFRSRS-----VGIFDVGG--RHPNVVASGT
PEEGY------------DYAIKDG----DVV----------REQFFELVKSLDPKTFVTRLQDLQRFADWKFRSDPEPYI
TPNNI-HFPDDGTDGRAEWVHSS-------------LCLYGESRLGKTLWARSLGHHVY-----FCGLFSGKELLNNLDA
DYAVFDDMQGISFFH----GWKNWFGCQL--NFQVKQMYRDPVNVTWGKSCIWGSNDDPRDSMKQ--------
VDVDWIN
KNCIF------
>Genomo_AUM61807
YSKTFLLTYSQCDLPKDAVKEYLNSISELGQEHHQDGNLHIHAWIKFKTKYQSRN-----PRCFDING--FHPNIMTARS
KKGSI------------AYVSKEDTDPLQ------------SKEFMSLVLQNHARDYANNYDRLISMSKNHYKPEPVQYT
PKYKEFRNLPMDMLTINXNDTD----------RPRTLIIHGESRLGKTQWARSLGKHIY------SRGCIIYDDFIRSPT
AEYAVFDDLWDWNY-----FLKDFIGAQQ--TVTITGKYRKPVQLNWSRRSIFLTNEFEQWSQEQQ----------YLQA
NSTIITL----
>Genomo_KJ547626
AAKQFFLTYPRCDLDLQLLLDGLTT---AERHGDEG--LHVHAIIVCAERIDTSN-----PRFFDVAG--FHPNIQTVRN
LRQAY------------TYLDKE-----PVQ----------AVEFMNLMKKYHPRDYILSFTRLLDFAEVHFTEPPPLYE
TP--------------EGY----------SFYRPKSLVLIGPSRTGKTTWARSLGRHVY-----WNSLVNLDVWS--PLA
DYIIFDDVD-IDFLP----GYKCWLGAQK--EFTVTDKYKKKRKIMWGKPCIWLCIKDP--LLSS--------KVYRWIS
MNCVF------
>Genomo_KJ547627
-------TYPQCSLERTELRDSLIQ---REQHSDGG--LHLHAYLHFGRRRRFTS-----ADAFDVDG--FHPNIQKPRS
ARNVI------------AYCSKE-----DKD----------E--FLEEVRTHFPRDYVLSLERLLFFCEWRFGRDETEYT
GRSRT---EFREPDTLTNWVNTNL--------LQVCIWTYGPSSLIWTQWARSISTHVY-----CQGMFNLDEWN--DKA
KYVIFDDID-IKYFP----HWKSILGCQR--DIQLTDKYRKKRRLRNGLPCVWLCNMDPRGALS---------RTQCWIE

SNCDV------
>Genomo_KJ547634
RAKAFIVTFPQVSEDVQQRFDHEGASLLDERHQDGG--VHYHMYIGFDEVVHINR-----ANLFDYFG--
AHGNIKSVRT
PRTVY------------DYCGKD-----GDV----------KDTFLSLCRQLAPKDWLLSNSRILEYANTYYPEIPSPYE
GPPIA---EM------ERYPELERWLE--QAERVKSLILYGPSRTGKTLFARSLGEHAY-----FNLQFNMDGFS--DGV
KYAVFDDIQGFEFWH----SYKGWLGAQK--EFVITDKYRKKRTIKWGKPTI-MCLNPN--FLKG--------VDYEWLQ
LNCVI------
>Genomo_KJ547638
HCRYALITYAQCDL-AGPTVGEFFE---SENHADGG--VHLHCFVDFGRKRRFRR-----PRVFDIEG--RHPNIEPSGT
PEKGW------------DYAVKDD----DIV----------RESFWDLVHELDPKSAACSFTQLQKYCDWKFAPIPPVYE
SPGGI-SFTGGDVDGRDDWLSQSGIG---SGPRCMSICVYGESRTGKTLWARSLGPHIY-----CVGLVSGDECMKANDA
EYAVFDDIRGIKFFP----SFKEWLGCQA--WVSVKCLYREPKLVKWGKPSIWLSNTDPRDYME--------NSDIDWMN
KNCIF------
>Genomo_KJ938716
NGIRFFLTYARAAI-SIDDVADHLH---QEDHPDGG--IHYHVVLCFATRYQG-A-----LDSFDVLG--HHPNWTPIRN
ATNRR------------HYIRKGI-----RA----------GEEFLALCRLHQPKDWILRNEALTKYAASYYQEAREKKS
LPPG------TLSSFLPRWMRYVYSGSTPKPDRPKTLLLVGPTRLGKTHWAESLGRHSY-----MCGMWRSDSFN--DRD
DYLILDDFDFDFFHG----MRKAIWGAQP--EFTTVDKWRKG-VAKWGKPLIWLCQKNPFVALKNG-
RPVMAEEERAWYR
ANCVE------
>Genomo_KM598389
DGSHVFLTYPQCSLSKERVRDFLLV---EELHGDGQ--PHIHAYAAWDSRRRLVG-----AGCFDVDG--HHPNIQKPRS
AKAVA------------EYCGKH-----DTE----------AKTFLARVEEHYPRDLCLSLDRLLQFCEWRWGTERIGYS
GRTRE---LFLEPDQLREWVRLSLEVL--MPERPISLLLVGASKLGKTEWARSLGPHMY-----FCGQFNLDDWN--DEA
KYIVLDDFN-IKFFP----QWKSFFGAQK--QFVLTDKYRKKRTVKWGRPLIWLCNADPRGALSG--------AELEWLQ
INAMM------
>Genomo_KM821748
NAQRYFLTYSQAAL-DIDDLANFLH---AENHQDDG--IHYHVVLCFDERFQR-A-----LDVFDLDN--HHPNIAIIKN
ASNRR------------HYIRKGA----DRP----------EEEFLILVRVNQPTEWVLRNDSIVKYAKTHYKAAREEKV
YPPES----WVVPPALDDWVAEVFSDVTPRPDRPKTLLLVGPTRLGKSVWAKSLGRYSY-----MCGMWHSDEFD--ESA
QYLILDDFEFDFFHG----MRKAIWGAQE--VFTTTDKWRKG-VARWGKPTIWICQHNPFTAVKTG-
LAVMDETERDWYK
QNCVE------
>Genomo_KP153522
DARHLELTYSRCNATVEDITEFLETTVSSAREYHQDGVPHYHILVKFNKRRTFRN-----SREYDFNG--HHPRIKPVRN
VHDYF------------RYISDPSKPSRDTT----------KDEYFTLLKLHKPRDYCLNLQRLEYTASRLFTAIT-PYV
PEYEYDTFN-VPEELHEWATVG-------LQPRPKSLIICGPSRIGKTEWARSIGLHTY-----WYGQCNIDS--WND-K
MEYLIMDDFSPTKY-LP--LWKGYFGAQK--ELNVTQKYRGIYTKLFRVPVLWLCNQIPKDWEQEWLNMNAD--------
----
>Genomo_KT253577
SARYVLLTYAQSDL-SEWDILDHIS---SEDHADGG--THLHVFVDFGRKKQSRR-----SDFFDVGG--HHPNITPSGR
PECGY------------DYAIKDG----DVV----------REEFFDLLRELDPKTLVTRWSELCRYADHAYESKPEPYV
GPSGV-EFELGMVPELVRWRGDT-LA---DDSE-LVLCYFGASRLGKTLWARSLGPHVY-----TMGILSGHVLLRDPEV
NYAVFDDMRGIGMFP----SFKEWLGAQA--VVSVKKLYRDPVQVPWGRPCIWLSNADPRDQIKSGLSD-
RVENDIAWLE
DNCIF------
>Genomo_KT598248
NAKYFLITYSQSGL-DEWAVNDHFS---SEDHADRG--THLHAFVAFERKFRSRR-----PDIFDVGG--FHPNIAPSGN

PAGGY-----------DYATKHG----DVV----------GGELRDLVRELAPKEAILRWREIEGYIGNEFAEERPAYV
HPIGF-EFELGMVPELVEWRRNT---LD-ADVRYKSLVIFGPSQTGKSTWARSLGNHFF-----ARGKFNGREFVKVQSV
DYYVLDDMEGLRFFP----GWKHFLGMQT--
WFNVRQFHRDPPMVKGGKPCIWLCNLDPRDEMYANLRHNQVDNDVAWLE
ANCIF------
>Genomo_KT862241
QARYVLLTYAQCDL-DAWAVNDHLA---FEHHADGG--THLHAFCDFSRKFRSRR-----PDVFDVGG--FHPNIEASGR
PEGGY-----------DYAIKDG----DVV----------EGDFWECVARLDPRSLCTNYNSLRAYANWKYRPTPVQYV
HPAGV-EFELGMVPELAVWREIA-LG---ANRRAKSLVIYGDTRLGKTLWARSLGPHIY-----TIGQMSGEVILRDPDA
DYVVFDDMRGLDFFH----GWKEWFGCQT--VVTVKKLYKDPMQMPWGKPVIWLANRDPREELRDSITC-
HIEGDIKWLD
GNCIF------
>Genomo_QCH00638
PRDGIKESQ----LVRLLQTLDLHEAYLGREIG-DGGYRHYQCAIDCAGDLRAFV----DNNQLGWHV-------EDCVS
WNDAI------------NYSRKTDNYRYV--------GDSIEEREYQRIAGRPINIVGT-----------------RID
KHIREQNDRQ-----------------------ISVCVDTVGGTGKTTHGYLNVRRTA------ETPVRIMDYIAIHYK
NENVIWIDLPRSRKVDI--NLAECLEDIK-DGLIASAKYQGTIRLIR-GVKVLVTTNHWIDKTAYK-----------MLS
TDRWDVFT---
>Genomo_YP_003104796
HAKYVLLTYAQCEL-DAFRVMDKLS---LEHHEDGG--THLHCFAEFGRKFRSRK-----ADVFDVDG--HHPNITKSGT
PEKGY------------DYAIKDG----DVI----------RDEFWELVHELDPKAAATSFSQLQRYCDWKYQYHAPEYE
SPAGA-HFIGAELDGRNRWLEQSGIG---SSERVKSLVLYGPSQTGKTSWARSLGKHIY-----CVGLVSGTECLKAPDV
EYAVFDDIRGIKFFH----SFKEWLGCQP--HLSVKELYREPKVIEWGKPSIWCSNADPRNELL--------QVDIDWME
MNCTF------
>Genomo_YP_009021043
NARYALITYAQCAL-DGFRVMDHFS---GEIHQDGG--VHLHCFIDFGRKFRSRR-----TDIFDVDG--RHPNIEPSGT
PWRGY------------DYAIKDG----DVI----------RDHFWELVHHLDPKAAACNYGQLAKYADWRFASVPPVYE
SPGGI-SFIGGDVDGRDAWCEQSHIR---SGPRCMSLVLYGDSRTGKTLWARSLGTHLY-----TVGMVSGEELKKADDV
KYAIFDDIRGIKFFP----AFKEWLGAQA--YVTVKELYREPKLVKWGKPSIWISNDDPRLGMD--------ASDVSWLE
HNCYF------
>Genomo_YP_009109725
DAKYCLLTYPQIET-EAYEFPELAS---EELHADGG--YTSHCFLDFGRKFSSRD-----TRIFDIQG--HHPNIERVRT
PRTAY-----------NYTIKDN----DVV----------RDEFFDLCKSLQPRSLACSFLSLTRYADWRYRPVPTPYQ
HPDDW-SFNLESHSVLLDWVDESLR-----GGQDRSLVMYGETRLGKTVWARSLGPHLY-----FCGLYSYKEASRAHEA
EYAIFDDLQGIKFFH----GFKNWLGAQQ--EFQIKGLYRDPELLKWGKPSIWCSNTDPRQDLD--------YSDRCWLE
GNCVF------
>Genomo_YP_009109727
NSRYVLLTYAQCDL-DPWSVSNHLS---AEIHTTGG--IHLHCFADFNRKFRSRS-----ARIFDVDG--RHPNVVPSGT
PEKGY------------DYAIKDG----DVR----------TTEFLELHDEMDRSGLIARFNNVRAYADWRFRPEPVVYA
SPDGV-DFRSGSTDGRDDWLVQSRIG---DEPIFCLLILYGPSLTGKTTWARSLGDHIF-----IQGVLSGKEVLNSESA
RYAVLDDIRGLKFFP----AWKDWLGGQR--WISVKQMYRDPILLKWGRPCIWCANRDPRADIRRSIDK-
DMEDDMDWIN
ANCIF------
>Genomo_YP_009109729
NARYFLVTYPQSGL-DEWAVNDHFG---SENHAVRG--THLHVFCDFGRKFRSRR-----ADIFDVGG--FHPNIERSRN
PRKGA-----------LYACKDG----DIV----------QREFFELAEDICPWDLITKFGSMHAYAKWKFPELAEPYE
NPAGF-TLADGAFPDLVSWRTG----AL-EHSRIKSLVLIGDALTGKTTWARMLGNHLY-----MKERYNAKQASLADGV
DYGVIDDISGIKYFP----HWKSWFGGQP--HIQVKILYKDERLVKWGKPLIWIANRDPRDQLRDMVSR-
DCNNDVYWME

GNAIF------
>Genomo_YP_009109733
DGRYFLLTYAQCTL-DAWTVNDHLA---FELHADGG--THLHAFCDFSRRFRSRR-----SDVFDVGG--RHPNLVPSGK
PEGGY------------DYAIKDG----DVV----------EGEFWKCVARLDPRALCTNYNSLRAYANWRYRPAPVPYE
HPAGI-EFELGMVPELAVWREIA-LG---ADR---ILVIFGDTRLGKTLWARSIGPHIY-----TIGQMSGEVILRDPDA
DYAVFDDMRGLEFFH----GWKEWFGCQS--VVTVKKLYRDPVQMPWGKPVIWLSNRDPRDELRDSITN-
HIEGDIKWLD
GNCIF------
>Genomo_YP_009115514
HARYALLTYAQCDL-CPFTIVDLLS---TEHHQDGG--IHLHVFVDFGRKYRSRR-----ADTFDVGG--FHPNISQSGT
PEKGY------------DYACKDG----DVV----------RESFWDLVHDLDPKSAVTCFTQLQKYCDWKYRYCPPAYE
SPAGA-RFRNDTSDGRGDWLLQSGIG---G-ARVKSLVLYGPSQTGKTTWARSLGAHIY-----QVGLLSGSECMKAPDV
EYAVFDDIRGMKFFP----SFKEWLGCQP--HVCVKELYREPRVIEWGKPAIWCSNADPRDDMS--------YCDVQWME
ANCTF------
>Genomo_YP_009115515
HCRYVLLTYAQCGL-SADAVGERMA---SEDHADGG--VHLHVFVDFGIKRRFRR-----ANIFDVDG--CHPNISPSGT
PEKGY------------DYAIKDG----DVV----------RESFWSLVHELDPKSAACNFTSLQKYVDWKFAAVPPVYE
SPAGI-SFRGGDVDGRDQWLLQSGIG---LGPICMSICVYGESRTGKTLWARSLGPHIY-----CVGLVSGDECMKAQDA
EYAVFDDIRGIKFFP----SFKEWLGCQA--WVTVKCLYREPKLVKWGKPSIWLSNTDPRDHME--------NSDIDWMN
KNCIF------
>Genomo_YP_009115519
HCRYALLTYAQCDL-SPTAVGEFFD---NENHADGG--VHLHCFVDFGRKRRFRR-----ARVFDIEN--RHPNVEPSGT
PEKGW------------DYAVKDG----DIC----------REAFWDLVHELDPKSAACCFTQLQKYCDWKFAPRTPEYE
SPGGL-EFIGGGLDGRDDWLLQSGIGG--RARRCMSICVYGESRTGKTLWARSLGPHIY-----CVGLVSGDECMKASTA
DYAVFDDIRGIKFFP----SFKEWLGCQA--WVSVKCLYREPKLVKWGKPSIWLSNTDPRDYME--------NSDIDWMN
KNCIF------
>Genomo_YP_009164036
GCRYALITYAQCRPRIPWAIVGLFS---TESHADGG--IHFHALVDFGRQFGSRK-----ADVFDVGG--RHPNIQKSGT
PEKGY------------DYGIKDG----DVV----------AGEFWRLCHELAP-EHVCRFPSYARATSCADRSLARSLA
ARHGV-TRNVCNADKC----------------RRKSLVVVGESELDKTVWARSLGNHLF-----FSGLYSGAEAMRYADA
EYAVFDGMQGIGSFH----GYKNWLGAQM--NFQVKVLYPDPKMITWGKPCIWLSNEEPRN--E--------SVDQDWLK
ENCIF------
>Genomo_YP_009181999
NAKYFLITYSQSGL-DEWAVNDHFS---SEDHADRG--THLHAFVAFERKFRSRR-----PDIFDVGG--FHPNIAPSGN
PAGGY------------DYATKHG----DVV----------GGELRDLVRELAPKEAILRWREIEGYIGNEFAEERPAYV
HPIGF-EFELGMVPELNLG--------L-ADVRYKSLVIFGPSQTGKSTWARSLGNHFF-----ARGKFNGREFVKVQSV
DYYVLDDMEGLRFFP----GWKHFLGMQT--
WFNVRQFHRDPPMVKGGKPCIWLCNLDPRDEMYANLRHNQVDNDVAWLE
ANCIF------
>Genomo_YP_009252353
NSRYVLLTYAQCDL-DPWAVNDLLS---TERHEDGG--IHLHAFVDFNRKFRTRR-----SDIFDVDG--HHPNISQSGT
PEKGY------------DYAIKDG----DVV----------REQFWELCHELDPKAAATSFTQLSKYADWRFAPDPPVYE
HPIGI-SFTDGDLDGRREWLDQAGIG----GGRCKSLCLFGRSRTGKTLWARSLGQHIY-----CVGLVSGDECMKAPDV
DYAIFDDIRGMKFFP----SFKEWLGAQA--WVTVKRLYREPALVQWGKPSIWLANSDPRNDMS--------QDDVQWME
DNCIF------
>Genomo_YP_009252356
NAKYVLLTYAQCDL-DGFAVMDRIS---EETHADGG--THLHVFCDFGRKFRSRK-----TDVFDVLG--YHPNIEPSGT
PEKGY------------DYAIKDG----DVI----------RDEFWELVHLLDPKSAACSFGQLQKYCDWKFAVAPPTYT
SPTGV-EFDDGSIDGRLDWLQQSGVG---SGPRCMSLCLYGESRTGKTLWARSLGAHIY-----

CVGLVSGDECMKADDA
DYAIFDDIRGIKFFP----SFKEWLGCQA--WVTVKCLYREPKLIKWGKPSIWISNTDPRDNME--------TSDVHWMN
KNCIF------
>Genomo_YP_009252359
SARYVLLTYSQCTL-DEWDVLDHIS---SEDHADGG--THLHVFVDFGHKKQSRR-----SGFFDVGG--KHPNVVPSGR
AGEGW------------DYAVKDG----NVV----------REEFLELVRALAPKEFILRHKELLEYADRYYAERIEPYV
GPDGI-EFELGMVPELAGWGREL-VE---ADSEAKSLVLYGPSRLGKTLWSRSLGSHVY-----IMGMLSGAVLLRDPDA
QYAVFDDLRGIAMFP----SFKEWLGAQA--VVTVKKLYRDPVQVSWGKPCIWLANSDPRDQLKADITE-
RIYEDIAWLE
DNCVF------
>Genomo_YP_009252362
NSKYVLLTYAQCDL-DEWAVSDHLS---SELHTTGG--VHLHVFVDFGRKFRSRR-----VDIFDVEG--RHPNVVPSGT
PEKGY------------DYAIKDG----EVV----------QDEFLDIYGEVDTRGLIKNFANVRSYAKWRYAGTLPKYE
SPYSIGEFRRGS-DGRDQWLAQSGIR---SGRPKS-LVLYGPSRTGKTSWARSLGTHVY-----FGGAFSGGDALACDDV
KYAVFDDMRGIPFFH----GWKDWLGAQQ--EFMVKALYHDPKLFKWGRPSIWCANRDPREEMEN--HM-
FCRGDIDWLN
ANCIF------
>Genomo_YP_009252365
SARYVLLTYPQSEL-SEWAVLDHIS---GEDHADGG--THLHVFADFGRKKQSRR-----GDYFDVGG--KHPNVVPSGR
PEGGW------------DYATKDG----NVV----------REEFLDLVRQLDPKSFVLKHQEIVRYADIFFAEDREPYV
GPDGI-RFELGMVPQLDEWRRES-LG---DNPKSRSLVLYGPSRLGKTIWARSLGPHVY-----IMGLLSGAVLLRDPGA
SYAVFDDMRGLPMFP----SFKEWFGSQS--LVTVKKMYRDPVQMRWGKPCIWLANSDPRDQLKADITD-
RIYEDIAWLE
ANCVF------
>Genomo_YP_009252368
GARYALITYAQCAL-DGFRVMDHFS---GEVHADGG--IHLHCFIDFGRKFRSRR-----TDIFDVDG--RHPNIAPSGT
PWRGY------------DYAIKDG----DVI----------REHFWELVHHLDPKAAACNYGQLAKYADWRFAAKPPVYE
SPGGI-EFVGGDVDGRDAWCDQSGIR---SGPRCMSLVLYGDSRVGKTLWARSLGAHVY-----
TVGMVSGGELKKVDTV
KYAVFDDIRGIKFFP----AFKEWLGAQA--YVTIKELYREPALVRWGRPSVWISNDDPRLVME--------ASDVSWLE
ANCIF------
>Genomo_YP_009351871
AAKSVFLTYPKCHLDKEVFVKAIED------HKDGT--PHYHVIGEWSVKKNIKN-----PRHFDIQK--YHPNIGRTRN
RLAAW------------RYVHKTK----GRS----------RNTFISRFRQEAPRDLIVHYSNVKAYADDEFKVEAPEYC
TPEMS---GYWDLPEELKEWVEENLTH--KSARPKSLCLYGETRLYKTIWARSLGPHSY-----MSGCWNARLLD--DDK
QYVVIDDVPLKDMFK----HFKQMLGCQN--NFSVTDKYVKKLHFKWGLPCIYLANQDPREYSDG--------THRRWLN
GNCVF------
>NanoAlpha_AAA51422
NYSSAAERE---N-FLLLKEEDVHYAVVGDEVAPATGQKHLQGYLSLKKRIRLGG----LKKKYGSR-A----HWEIARG
TDEENS-----------KYCSK-ETLILP-DR----MKIEQPEIFHRYQSVNKLKKFKEEFVHPCLD--------RPWQI
QLTEAPDDRS------------------------IIWVYG-PYNEGKSTYAKSLIKKKE------NL------FSYVDEG
SDKHIVFDIPRC-----DYLNYDVIEALK-DRVIESTKYKPIKIVG--KIHVIVMANF---MPDF------------CKI
EDRIKIIY---
>NanoAlpha_AAA51426
NYSSAAERE---D-FLLLKEEDVHYSVVGDEVAPATGQKHLQGYLSLKKSIRLGG----LKKKYGSR-A----HWEIAKG
SDEQNR-----------RYCSK-ETLVLP-EE----LKMDDPSKYRRCLAVESIKDARINSEWVHEL--------KEWQN
KLIQHPDDRS------------------------IIWVYG-PNGEGKSTFARYLLKPTS------DM------HIITMD-
PDNHWIIDIPRS-----DYLNYGVIEQIK-NRVLINTKYEPCVIRGQ-NVHVIVMANV---LPDY------------CKI
EDRIKIIN---

>NanoAlpha_ACB86656
HYSDATERG---K-FLTLKEEDVHYAVVGDETAPNTGRKHLQGYLSLKKRFRISG----IKKKYSSR-A----HWEKARG
SDYDNK-----------AYCSK-EALILP-ER----MKIEQPEIFHRYASVKKMKEFKERYVVYPILD--------RPWQV
QLTELPDDRT------------------------IIWVFG-PKNEGKSTYAKSLIQKKE------NL------FAYVDEG
STKNVVFDLPRT-----EFINYDVIEALK-DRVIESTKYKPVKYLN--TVHVLVMANF---LPDM------------CKI
EDRIKIVA---
>NanoAlpha_AIF34798
NNYAAADL----------PLCKDRYVVWQHEKV-DTD--HIQGYIELSKPQRISA----MIKWLPG--A----HFEERRG
TPDQAR-----------KYCMEEDTRVEADRR----EVYNAHSDIAAKYPRYVETMLRFAKEDAILVFEPR----QGFQT
DLLDMADSRS------------------------IHWVYD-RVNNGKTYFAKYLVDKSV------DA------YAYGGE-
--SIVIFDYVRD-----EYVGYGVIEQLK-NGIAMSTKYESITKRNIP--HVIVLANF---KPQE------------GKF
SDRIKMIN---
>NanoAlpha_AKO71308
NN-PTLPV----------MRDEIKYMVYQVERG-QEGTRHVQGYVEMKRRSSLKQ----MRSFFPG--A----HLEKRKG
SQEEAR-----------SYCMKEDTRIEP-LE----YLYDCPNTFDRSKDTLYRVQSEMNKTKAMNSWRTS----FSAWT
SEVENPCHRR------------------------IIWVYG-PNGEGKTTYAKHLMKTSL------DC------RLYNYE-
--DIVIFDIPRC-----DYLNYGLLEEFK-NGIIQSGKYEPVLKIYV---EVIVMANF---LPKE------------GIF
EDRIKLVS---
>NanoAlpha_ALK03646
FNYV----------LLFSLPPWANYIVYQEEICPDTSRKHIQGFINLKRPQRFSF----LKKNLPDG-A----HIESCKG
SASSNR-----------DYCTKDASRFAP-EE----LRLADPKLYRRCLATKVNFEFSSVVLPVFDR---------PWQL
LVEKIIDDRT------------------------IIWVYG-SQNEGKTTWAKKKVQDGE------NK------YQYVEH-
-LGNCIFDIPRQ-----DNLQYTVLEEIK-DRLIRSSKYEPIDVND--NVHVVVLSNF---LPQLDVYDSRGNVVKRQLL
RDRLCIVN---
>NanoAlpha_HE654123
NN-PLPLS----------LHESMKYLVYQTEQG-DSGNIHFQGYIEMKKRTSLAG----MKKLIPG--A----HFEKKKG
TQGQAR-----------AYAMKEDTRVEP-IE----YIEECCDTYDKSASTLREYRGELKKKQAIASWELQ----RKPWM
DEVDARDGRR------------------------IIWVYG-PLGEGKTSYAKHLVKTTA------DA------FAWDHQ-
--ELVLFDFPRS-----EYVNYGAIEQLK-NGIIQSGKYQSVIKYYV---EVIVFANF---TPRS------------GMF
DDRIVFVY---
>NanoAlpha_HM163578
FSYV----------LLFSLPEWAEYLVFQEEQCSSTGRKHIQGFVTLKRSQRLSF----LKNKLGDG-V----HLEIAKG
SASSNR-----------DYCTKDDTRSSP-EE----MRLSDPKLYRRCLATRVNKEFSGLVLPVLDR---------PWQL
LAEKVPDDRT------------------------IIWVYG-SQNEGKTTWAKSKIQAGE------NK------YSYADH-
-LGHAVFDLPRQ-----DVLQYTVLEEIK-DRLIRSSKYEPIDFND--RVHVVVLSNF---LPQLDLYDSRGNLVKKQML
RDRVVIIN---
>NanoAlpha_JF957636
NN-PTLPV----------MRDEIKYMVYQVERG-QEGTRHVQGYVEMKRRSSLKQ----MRVFFPG--A----HLEKRKG
SQEEAR-----------SYCMKEDTRIEP-LE----YLYDCPNTFDRSKDTLYRVQAEMNKTRAMNSWRTS----FSAWT
SEVENPCHRR------------------------IIWVYG-PNGEGKTTYAKHLMKTSL------DC------RLYNYE-
--DIVIFDIPRC-----DYLNYGLLEEFK-NGIIQSGKYEPVLKIYV---EVIVMANF---LPKE------------GIF
EDRIKLVS---
>NanoAlpha_JX458742
NFSGDPP------ELAWLARDEIKYACWQHEKG---THDHMQGYLQLKKPSRLTA----VRKLFGNR-P----HLEVQKA
RAEDAR-----------DYCMKDESRVAP-ER----MAEENPSVYRRVLAAQSVERFRSDPSLLPDP---L----RDWQI
CLLELPDNRS------------------------IIWIYG-PAAEGKTMMAKELFRRAD------NK------YQYAVE-
VESHVVFDIPRD-----DYIQYSLIEELK-NGMIVSNKYEPIRVVSGREVHVVVMCNF---MPDY------------SKI
PDRIVIVY---
>NanoAlpha_KC978991

NFQGALS---------LSFDERVQYAVWQHERG---THDHIQGVIQLKKKARFST----VKEIIGGN-P----HVEKMKG
TIEEAS-----------AYVQKEETRVAP-EE----MKLKDPDVALRCN-AKRLKEDYCSCFSSFKL--------RPWQI
ELHRVPDDRS-----------------------IIWVYG-PDGEGKSTFAKELIKYTQ------DL------YMYAQD-
PERNIAFDVPRC-----EMMNYQAMEMMK-NRCFASTKYRSIDLCK--NVFLVVFANV---EPDP-----------TKI
GDRIVIIN---
>NanoAlpha_KC979052
NFKG---------EIILSLDTRVQYALWQHEYVS---HHHLQGFIQMKAQSTLGQ----MKALIPG--A----HFEVMKA
DSDQAR-----------SYAMKEDSRLEP-EE----MKVEDPSLYRRCLSRKMTEEQRSSSTWDYDL--------RPWQD
SILEAPNYRD-----------------------VLWVYG-PNGEGKSTFARHLLKDTQ------DM------HLITAE-
PKNNWVFDIPRV-----DYINYGVIEQVK-NRVMVNTKYEPVIMRN-NPVHVIVFANC---LPDV-----------TKL
EDRIKMIY---
>NanoAlpha_KF471057
FFSATAP------DLVLFENTHVSYACWQEEESPTTRRRHLQGYLQLKGKRSLAQ----VKALFGDN-P----HLEKQRA
KTDEAC-----------DYCMKEETRVSP-VR----MAEENPSLFRRVKAKIAEEEFQKTAREIQILN--L----KSWQS
RLQTLPDDRT-----------------------IFWVYG-PTGEGKSTFARDLYRSAD------NS------YQYIGQ-
LGNNIVFDIPRD-----DYLQYSLIEMFK-DRLIVSNKYEPLMAPN--CIHVVVMSNF---MPDF-----------EKI
QDRVHVIP---
>NanoAlpha_NP_619759
NFSG---------DASLSFNERVQYACWQHERVS---HDHLQGYIQMKKRSTLKM----MKELLPG--A----HLEVSKG
TPEEAS-----------DYAMKEETRVAP-ED----MELEDPAKARRCRAKIDKEKFIAEFKVEDDE--------QEWKK
ILEKEASPRS-----------------------ILWVYG-PQGEGKTSKAKELITRKD------DA------YSYVED-
PTRHVVFDIPRD-----EYCNYSLIEMLK-DRIIISNKYEPITNCY-N-IHVIVMANF---LPDV-----------TKI
EDRIKIIY---
>NanoAlpha_NP_619760
NYKTALERE---TFILFSRDE-LNYFVCGDEIAPTTGQKHLQGYVSMKKLIRLGG----LKKKFGSI-A----HWEIAKG
DDFQNR-----------DYCTK-ETLIAP-EE----MKLRDPDTALRCK-AKKLREEYCSEVSVFSL--------RPWQI
ELHRAPDDRT-----------------------IIWAYG-PDGEGKSTFAKELIKYTQ------DL------YMYAQD-
PERNIAFDVPRC-----EMMNYQAMEMMK-NRVFASTKYRPVDLCK--KVHLIVFANV---APDP-----------TKL
EDRIVIIN---
>NanoAlpha_U16735
NFEG---------EIILPFNESVQYACWQHERVG---HDHLQGFIQFKSRNTLRQ----AKYIFNGN-P----HLEIAR-
DVEKAQ-----------LYAMKEDSRVAG-EE----MKIADPSLYRRCLSRKMAEEQRCSSEWNYDL--------RPWQE
EVMHLPDYRT-----------------------IIWVYG-PANEGKSTFARHLLKDTQ------DM------HLVTAE-
PKNNWVFDIPRV-----EYVNYGVIEQVK-NRVMVNTKYEPCVMRN-HPVHVIVFANV---LPDL-----------GKL
EDRIKLIR---
>NanoAlpha_YP_003104737
NN-PLPIF----------LHESMKYLVYQTEQG-ESGNIHFQGYIEMKKRTSLAG----MKRLIPG--A----HFEKRRG
TQGEAR-----------AYAMKEESRIEP-IE----YIEECCNTYDKSASTLREFRGELKKKKAIATWELQ----RKPWM
DEVDARDGRR-----------------------IIWVYG-PQGEGKTSYAKHLVKTTA------DA------FAWDHQ-
--ELVLFDFPRS-----EYVNYGVIEQLK-NGIIQSGKYQSVIKYYV---EVIVFANF---TPRS-----------GMF
DDRIVFVY---
>NanoAlpha_YP_008169853
FSYV----------LLFNLPDWANYLVFQEEESPSTGRRHIQGYVNLKSPQSFSF----LKKKLGDG-V----HLEQARG
SASCNR-----------DYCRKTDSRVSP-EE----LRLSDPKLYRRCLATRVNTEFAGLVLPVLDR---------PWQL
LVEKVPDDRT-----------------------IIWVYG-SQNEGKTTWAKSKVQAGE------NK------YSYAEH-
-LGHAVFDIPRQ-----DVLQYTVLEEIK-DRLIRSSKYEPIDFND--QVHVVVLSNF---LPQLDSHDSRGNLIKKQML
RDRVVIVN---
>NanoAlpha_YP_009058890
NFQGALL---------LSFDERVQYAVWQHERG---THDHIQGVIQLKKKARFST----VKEIIGGN-P----HVEKMKG

TIEEAS-----------AYVQKEETRVAP-EE----MQLKDPDTALRCN-AKRLKEDFMKEKTKLQL--------RPWQK
ELHDLPDDRT-----------------------IIWVYG-PDGEGKSMFAKELIKYTQ------DL------YMYAQD-
PERNIAFDVPRC-----EMMNYQAMEMMK-NRCFASTKYRSVDLCK--NVHLVVFANV---AYDP-----------TKI
EDRIVIIN---
>NanoAlpha_YP_009246456
FNYV----------LLFTLPEWANYLIYQEEECPTTKKRHIQGYVNLKRNQRFAF----LKKKLPDG-T----HIEACKG
SSSSNR-----------DYCRKDDTRTDP-EE----LRLADPKLYRRCLATKVNTEFGGLVLPVPDR---------PWQL
VAQKVPDDRT-----------------------IIWVYG-SENEGKTTWAKTKIQDGE------NK------YQYAEH-
-LGHCIFDIPRQ-----DNLQYTVLEEIK-DRLIRSSKYEPIDFND--KVHVVVLSNF---LPCLDSYNNRGELVKKPLL
RDRVFLIN---
>pCRESS1_CDF01935
CVFNNPAEHT---GEPQEVCERLKEWVN-FCKS-KSGLLHVHMVLEDTKSMRFTA----IKSSYCQG-----MHFEPTKG
NKK-Q----------ADDYINKEIKGKQ------------GKRTDLDCISDLITDG-LKPSEILEENPRYYTKEN---II
KKMYFRKRYAETEFTRD----------------VKVFWHYGSSGSGKSYSRKQVVE------KTFG---GAFDNYEGQ--
-K-VLWIDDYRG--EFRFQELLRYLDVYK---AELPARYN-NVKALWNEVHITSVLTPQLCYSEACN------LDRIEQL
LRRITCL----
>pCRESS1_CUO23215
AVFPNPEQHE---GSPEDIIEKLKQWIG-YCIS-EKGLPHVHMVLEDTGSCRFTK----VKKAYPT------AHLEPTKG
NKK-Q----------VLAYIKKNIEGNK------------NTNDTLATIEMLIEEG-MTPNQIMAEDIRLRREEN---LI
RKCYFAKRYKETPPIRT----------------VKVVWHCGDSGCGKSYSYIDLCE------KDY----AGFDGYCGE--
-P-YLFMDELKQD-SLPFELLLTITQGYR---SQIHCRYS-NCITLWNEVHITSIFSPEDIYAGVVSR---EGKDTIKQL
LRRITKY----
>pCRESS1_CUO57637
AVLPYPEKK----GSPEEILEQMKQWIG-YCIS-KEGMPHVHMVLEGSVSMRFSA----VRKCYGK------AHLEPTRG
SRK-M----------VLQYIHKNIEGNK------------NQNAILDRIEELIEDG-KTPNEIMGEDIRLRKEET---LV
RKAFFAKRYRETPPYRK----------------VTVVWHLGESGSGKTFSYTKLCE------SEY----AAFDLYSGE--
-K-ILFLDEIRST-SLPYETLLTLIGPYR---TQIHCRYA-NAFALWEEVHICSILAPEDLYKGMVKS---EERDCIQQL
LRRINKY----
>pCRESS1_CVH76026
TSNNPHIHYRGDDDYRTACEAFVMWRRGRAFERGEQGTLHLHGLLCSKSDMGKST----LIEKFPQTD------FRETRG
SVDDC-----------LDYLHKRGWGDYLCGG------VGGGGKTFERIDAMLDQGMTPNDIFALGTKYAYYGQEIQRRY
NALMADKARSRD--------------------ALRCVYHTGDSGSGKSKLLEQQGRSVY-----YTADYDPFDSYNGE--
-DVLFLDELRSYSFETP--QLLSIMESYR---HEVPARYSNRLAVYSEVHLSSIFPPEKIVPPNEPLKQLLRRIDEVVYH
ATAGRYVT---
>pCRESS1_WP_000032131
CTLNNIDKLFTFNYSPEEMVEHLIYWVDGYEIG-DNGNHHSHLILEAKNQTRFSA----IKKLYPT------IHVELTRG
TRE-Q----------VIAYLNK--ANQQ------------GKRKDLDIIQELLEEG-LSPEEIMRQNLSYRKFSK---MI
KEHFYQLQVANAPLVKK----------------MKVYWHLGGSGTGKSVRLKEIFG------IDYGG----LDNYMGE--
-S-ILFMDEFKG--DIDYQAFLKILDVYP---NQVHARYS-NVYALWDKVHISSIFSPYQIYKMLVSP---DKNDPITQL
YRRIHFI----
>pCRESS1_WP_003030931
CVLNNVDKLFFFPLSPEEIVDFLMEWIARYEIG-DNGVHHCHMVLEDKKAFRFSA----LQKLYPT------IHAEITRG
TKE-Q----------IIAYLEK--ARNQ------------GHRSDFDYIQKQIENG-ATPEEIMMGNLEYRKYSK---MI
REHFFQHRLAQTPDIKD----------------MKVYWHVGESGSGKSVNLKKEFG------RDFDG----LDLYCAE--
-P-ILFMDEFKG---MSYKEFLKVTDVYP---VQLHARYT-NTIALWNEIHITSIFTPKEAYGLMVPE---SEIDSYKQL
QRRLTNV----
>pCRESS1_WP_003102166
CTLNNIDKLFSFTYSDEEMVEHLIYWVDGYEIG-DSGNHHSHLILEAKNQARFSA----IKKLYPG------IHVELTRG
TRE-E----------VIAYLNK--ANQQ------------GKRSDLDTIQELLEAG-LTPESIMRQNLSYRKFSK---MI

KEHYYQMQVENAPLVKD----------------IKVYWHLGSSGTGKSILLKQDHG------KDYGG----LDNYTGE--
-P-ILFMDEFKG--DIDYQAFLKLLDVYP---NQVHARYS-NVYALWDAVHISSIFTPNQLYEMLVPE---EKNDPIKQL
FRRIHFI----
>pCRESS1_WP_026524352
LTLNNPQKG-----YTHDFIIDTIHTFR-DEIG-ESGNYHTHIYILLGKKKRWSS----VQRAFLH------SHIEKVMG
SPQ-E----------CRAYIRKTIPTFK------------NDRE-MLQIEDMINQG-MRPEQIMQQSVVFRQFET---II
RKSFFAKRLKETPPLRQ----------------IKIVWHLGASGSGKSFSYTQLCE------QDYSNCTALFDGYEAQ--
-K-VVFLDEVKTD-SFKYGYLLQILQGYK---TQIHARYC-NIQSLWTEIHATSIYAPDEIYDEMVAV---PTIDSKTQL
LRRITDY----
>pCRESS1_WP_026669310
LTLNNPQKG-----YTHEFIIDTIHKFK-DEIG-KSGNYHSHLYILLGKKKRWSS----VKRAFPH------SYIVKVMG
SPQ-E----------CRAYIRKTIPTFK------------NDRE-MLQIEDMINQG-MRPEQIMEQSVVFRQFET---II
RKSFFAKRLKETPPLRQ----------------IKIVWHLGASGSGKSFSYTQLCG------QDYSNCTALFDGYEGQ--
-R-VIFLDEVKTD-SFKYGLLLQIFQGYK---GQIHSRYN-NVYSLWTEIHATSIFCPDELYDEMVPL---SSVDSKTQL
LRRITDY----
>pCRESS1_WP_029176105
CTLNHIDKLFSFSYSEEDMVNHLIYWVNTYEIG-DNGVHHSHLILEAKNQTRFSA----IKKLYPA------IHVELTRG
SRE-Q----------VIAYLNK--ANQQ------------GKRTDLETIQELLETG-LSPEQIMRQNLSYRKFSK---MI
KEHYYQIQVENAPLTKD----------------MKVYWHMGGPGSGKSVQLKKTFG------TDYGG----LDNYTGE--
-P-ILFMDEFKG--DIDYQTFLKLLDVYP---NQVHARYS-NIYALWDTVHISSIFTPYQIYTMLVPD---EKYDPIQQL
YRRIHYI----
>pCRESS1_WP_029690610
CVLNNVDKLFFFPLSHEEIVDFLMEWIARYEIG-DNGVHHCHMILEDKQAFRFSA----LQKLYPT------IHAEITRG
SKE-Q----------VIAYLEK--SGNQ------------GFRSDLDYIQKQLDNG-ATPEEVMMQNLGFRKYSK---MI
KEHFFQKKIKETPDVKD----------------IKVVWHWGESGSGKSTTLKETCG------RDHEG----LDFYNAE--
-P-VLFMDEFKG---MPYKDFLIVTDVYP---TQLHSRYT-NTFALWMEIHIASIYTPKHAYNLMVPE---GEIDSYQQL
KRRLSEI----
>pCRESS1_WP_029694263
CVLNNVDKLFFFPFSPKRMIEILIDWMEDYEIG-EDGVHHCHMILEGKQAIRFSA----LQKLYPT------IHAELTRG
TKE-E----------VFAYLNK--ASKQ------------GKRTDLEIIEQLLEEG-QSPEEIMCRNIGYRRFSK---LI
KEHYYQMRLRMSPRFKE---------------NMDVIYHVGASRSGKSIRLMDKYG------VDYQG----LDNYMGE--
-K-ILFMEEFKG--ELSYQEFLRVTDSYV---QQFHARFT-NIYALWEKVHISSIFPPKELYKLMVSN---SATDTMEQM
MLRISKV----
>pCRESS1_WP_047207334
CVLNNVDKLFFFPLSPEEIVDFLMEWIARYEIG-ENGTHHCHMILEDKKAFRFSA----LQKLYPT------IHAEITRG
SKE-E----------VIAYLEK--AGNQ------------GFRSDLHFIQHQIENG-ATPEEIMRGNLEFRKYSK---MI
REHFFQHRLSQTPDTKE----------------LKVVWHVGDSGSGKSVQLKQIYG------RDHDG----LDLYCAE--
-P-ILFLDEFKG---MAYKEFLKVTDVYP---AQLHARYT-NTLALWNEIHIASIFTPKHAYNLMVPE---GEIDPYEQL
QRRLTKV----
>pCRESS1_WP_053982727
GTIANMEKAG---ENPEQLAEFISEWSA-VCVS-AKGLYHAHVVLYGNLTTLGNV----AKILFDS-------HIEPQLG
GKK-E----------LKSYLLKNIQDTK------------GKRSDLEDIEELLEQG-FTPREIM-ENFPYRKYEK---MI
KSAFIDKRIQETPLLKE----------------KKCIWIVGESGTGKSYYYYQLCQ------EDF-----EFYIEQGA--
-PPILFMDEFKG--DMRFAQLLVMLDKFR---AQVHCRYS-NCFCLWSTVVITSVFPPDEVYAGMVDD---ADRDKIDQL
IRRLDVI----
>pCRESS1_WP_062004798
CVLNNVDKLFYFTYTPEEIVDYLMEWIGRYEIG-DKGTHHCHMVLEDKQSFRFST----LQTLFPT------IHAEITRG
TKE-E----------VLAYFEK--ANRQ------------GQRSDLDYIQQQLEEG-ATPEEIMLDHLEYRAYSK---MI
REHYYQLRLRDTPDHKE----------------LKVYWHTGDSGSGKSVRLKQEFG------RDYQG----LDGYQGE--

-G-ILFMEEYKG--EMNYAEFLKVTDRYP---HQMHARYS-NVFALWEEIHITSIFSPKQVVYNIMVPE---ETADSVDQM
MRRINKV----
>pCRESS2_BAK32345
ITIN----NPHDSITHEAILDTIEEMNG-DEIG-ESGTYHTHIYLVSNNAIRFST----MKNKFPT------AHFEIARG
TST-D----------NRDYIFK---EIPLER--------QGARNDLADLYDMIKQG--MDNFDILEST-PENMMKLDKIE
RTRQVIKESEYRNTFR----------------KLTVTYISGDTETGKTRYVMESNG-------DYK----PFDGYQGQ--
-P-VICFEEFRSS--LPIASMLNYLDGYP---LELPCRYS-NKIACYTSVYIVSNISLEKQYEYEQEN----ETETWNAF
LRRIHHE----
>pCRESS2_CBL15233
LTIN----NPDDELDHNSVKNTLHLFSP-DEI-ATTGTKHMHIFIYSKSPIRFST----LKNRFPV------AHIDKANG
SVM-E----------NRDYLRK---NVPKPV--------DENSPDMSALIEEIENG--LDTYEIIKLH-PKYAFRIKEID
TLRQTVLSNMFREKKR----------------QVTVYYIYGKSGTGKTRGIYQKHR-------AYRSTGINFDSYHGQ--
-S-VLVFEEFVSQ--IPIEDMLNYLDIYP---LMLPARFN-DKVACYDTVYITSNISLGEQYSEVQHY----KPETWKAF
LRRINFL----
>pCRESS2_CCX75435
LTIN----APVEGYTHEKIVNIIRSFKT-DEQG-E--RYHTHIFIVFSSRVRFSM----VKRYFPE------AHIEKCRG
NVS-E----------NVSYIKK---IQPSDS--------KGKRSDLSELYRMIQDN--MTNAEILAVN-QDYIMQIDRLD
KVRTTILMERFKETVRL---------------DLEVIYIFGKTGTGKTRRVLEENG-------DYN----PFDSYTAQ--
-Q-AICFDEFRSS--LKLKEMLLYCDIYP---IELPSRYS-NKFACYNKVYIVSNWELEKQYSELQRE----DKESWQAF
LRRIHKV----
>pCRESS2_CCY69022
LTIN----NPVEGYTHEKIKETLIMFTT-DEIG-GQGTYHTHIYVVFSSRVRWSK----VKKNFDE------AHIEIAKG
SAQ-S----------NVEYIKK---EIPTQ---------RGKKADMEELYEMIKNG--YSNAEILAIN-NDYILNIDKLD
KVRTMLLTEKYKNERRL---------------DLKVIYIYGATGTGKTRGVLDEHG-------DYL----PFDGYGTQ--
-E-VIAFDEFRSG--IKISDMLNYCDIYP---IELPARYS-NKFACYSRVYIISNWSLEMQYSEVQKN----SPESWQAF
LRRIHEV----
>pCRESS2_CCZ45692
MVIN----NPAEGLGHAAITEIVLKFHP-DEI-ATTGTFHTHIFFCTRSPVRFST----IKKRFPT------AHIERAYG
TPR-E----------NKEYISK---ELPADS--------EDKAPEMFQLMQELRSG--KSTMEVLEEH-PNLAFRIRDIE
LLRQTILAEKYSAENR----------------KLEVTYLYGASGVGKTWGIFEQHD-------NYRARGISFDGYNGQ--
-D-VLVFEEFNSQ--VPIEDMLNYLDIYP---LHLPARYN-DRVACYTKVYLTSNLPLEKQYRAEQWD----RPETWRAF
LRRIHNV----
>pCRESS2_CDB27189
LTFN----NPVQGFTHAVIKTTLASFPG-DEIG-EQGTPHTHLYLYSPNAILFST----LQQRFMG------AHMEAAKG
SHR-E----------NRDYIRK---PLPAEQ--------NKRESISSEILELVQSG--ASNAEILLQY-PSAMNRLQHIE
TARQTLLEERYRNQWR----------------NLEVTYLWGPTGVGKTRSVMELYS-------NYD----PFDDYRGQ--
-N-VILFDEFRSS--LPVADMLKYLDGYP---LMLPCRYS-NKVACYTKVFLISNIPLSAQYPNVQLS----EPETYRAF
CRRINQG----
>pCRESS2_CDC44519
LTIN----NPVEGFTHDKIKDLIYLMKP-DEIG-EEGTYHTHIFICGRSGIRFST----LKRAFES------AHIEMAKG
TSL-Q----------NKEYVSK---DMPIER--------QGKRNDLDDLYSMIKEG--MSDYDILEQG-SDYMLNLDMIS
KTRQILVQEKFKNTFR----------------KLDIVYIWGETGTGKTRSVMEGHG-------DYL----PFDNYAGQ--
-D-VILFDEFRSS--LALTDMLKYLDGYP---LELPCRYA-NRYACYTKVYLISNIPLSEQYPNVKRD----EYGSWLAF
LRRIQTV----
>pCRESS2_CDE72464
LTIN----NPLEGCSHEEIKKAMESFST-DEIGLEEHTPHTHVFIYTTNGTMFNT----VKNIFPI------AHIEHCKG
TYA-Q----------NRDYIRK---TMPTER--------QGGRNDLADLLDMITAG--LDTSTILEQY-PNYMLQLDKIE
RTRQIMLESKYKNVFR----------------EMEVQYIFGAPGSGKTRGVMEKYG-------DYK----PFDGYHGQ--
-D-VIIFEEFRSS--LKIGDMLNYLDGYP---LSLPCRFS-NKQACYTKVFIISNIPLTAQYNDLQSE----QTATWKAF

LRRLNGV----
>pCRESS2_CUP05665
LTIN----NPVEGFTHEKLNSILASMAS-DEIG-ENQTYHTHVFLCGRSGIRFST----LKKQFEG------AHFEMAKG
TAE-Q----------NMQYVSK---EMPIER--------QGKRNDLDDLYGMIKDG--LTNYEIMEQM-PEALLNLDKIE
MTRQTIIQEKYKNQWR---------------DVQVEYIYGDTGSGKTRSIMEQYG-------DYL----PFDGYKNQ--
-D-VVIFEEFRSS--IRFTEMLTLIEGYP---VELPCRYA-NKYACYTKVYIITNVPLSKQYPAVQLD----ESVSWLAF
LRRIHKV----
>pCRESS2_EES75484
LTIN----SPEKGYTHEVIYQVASNFKT-DEQG-SN--FHTHVLLVFKSRVRWST----VQDKFPH------AHIEEGKG
DIN-Q----------ILQYMRK---DRPVDT--------KEKVSEFSELYDLVYDE--VPTGEIIKFN-PKYIRYIDKIA
PMRIEIMNEKYRGKRRL---------------DLKVIYVFGLSGTGKTRMILDRHG-------DYF----PFDSYNMQ--
-Q-VLCLEEFRDS--LTITQCLNLLDIYT---VELPARYA-NKLGIYKTVYMVSNWEIGKQFKSVQQE----HPETYHAF
RRRFHYL----
>pCRESS2_KJZ87129
ITIN----NPVDGYTHENLKNILNNFKN-DEIG-ENKTYHTHIFLACSGAVRFST----VKKRFEG------AHFEMANG
TSK-Q----------NREYVFK---DCPIER--------QGQRNDLIDLYDSIKAG--LSNYDIIEDN-PNFMFDVDRIE
RARQMVRDEQYKNTFR----------------ELEVTYIYGKTGCGKTRGVMEQYG-------DYN----PFDSYKGQ--
-D-VIIFEEFRSS--LKIQDMLNYLDGYP---LELPCRYA-NKIACFTKVYIITNIALEHQYDSIQKE----FNETWNAF
LRRIHKI----
>pCRESS2_SCH17786
ITIN----NPADGFTHERIRQELESMKS-DEVG-ENGTYHTHLYLQGKGAVRFST----IKKHFEG------AHFEMAKG
TAM-Q----------NREYVSK---EMPIER--------QGARNDIADLHAMIKQG--LSNYDIMEQV-PEAMLMLDKIE
QARQTIVQESYKTKWR----------------NMSCYYIYGDTGTGKTRSIMEQYG-------DYS----PFDNYRGQ--
-D-VVIFEEFRSS--FRVSDMLNYLDGYP---LELPCRYA-NKYACYTKVYIISNIPLSEQY-RNQPQ----E--TFEAF
LRRLNGV----
>pCRESS2_SCH60086
FTINNPSKKTDTFDTDEKVINFIMQYEE-RERGHNENTEHIQGFIQFKNRKRGTT----LQNMFPP------QHGEFANG
TAQ-Q----------ASDYCKK---ELRVTK--------GGKQLTNEDILQRIKEG--ADDIRILEEFP-QLWNQIDRLQ
KVRDLYVFDKWRNVFR----------------DVQVTYICGQSGTGKTRSVMEQYG-------DYK----PFDSYHGQ--
-D-VIVFEEFRNS--LPIDNMLNYLDGYP---LELPARYM-NRIACFTKVYIISNWNFEEQYTAIQHK----YYETWNAF
VRRIDKI----
>pCRESS2_WP_007865724
CVFN----NTDKNCSHQAIKEKLSNWEN-DEIA-KT--PHTHLFVQFKNPVYFSS----IKKTFPS------AHIEEAQG
TAE-E----------NRAYIRK---TMPQTG--------QGRRSDLANLYQMIKDG--YSNVEILEIN-PDNLLNLQHID
KARLEILSSRYKAERRM---------------NLLVTYVSGATGYGKSRYILDNHG-------DYK----PFDTYSGE--
-D-VIVFEEFRSD--LPIGNMLNYLDVYP---LQLPARYN-NRQACYNFVYIVSNWKLDDQYHNIRLE----QKETWNAL
IRRIHKV----
>pCRESS2_WP_009246639
VTIN----NPLKGFNHLKIKKTLIEFAT-DEIG-KQGTPHTHIYVCFKSRVRFST----VQKYFPT------AHIEKPHA
SVQ-S----------NIDYICK---TVPLQ---------KGTRPDMEELYQMIDAG--YSNAEILAIN-NDYILDIDKLD
KVRTMLLIEKYKGKRRI---------------NLKVIYISGATGTGKTRGVLDEHG-------DYQ----PFDGYSCQ--
-P-VLAFDEFRSS--LKLSDMLNYCDIYP---IDLPARYA-NRFACYETVYIISNWELEQQYKEVQED----NPESWRAF
LRRIHEV----
>pCRESS2_WP_009301216
LTIN----NPKDGLTREIITDRMNSLFP-DEVS-QSGTPHTHIFIYRKSPIRFST----IRSKFPT------CHCEKAHG
SVL-E----------NKEYVSK---EIPNEK--------QEKNPLNYEVIKDLEDG--KVIGEIVSDR-PELIFKVKQIE
ALKEALLIKNANKFRS-----------------LSVIYCFGESGVGKTRMVYECHE-------NYRHKEMSYDVYHGE--
-K-VLLLDNFQNS--LCIDDLIALLDIFP---MYLPARFY-DRYSVYEFVYLLSVLPLENQYKDIQKH----YPLKWNAL
INKISKI----

>pCRESS2_WP_013270924
LTFN----NPEKNCSHQAIKEQLLNWEN-DEIA-KS--AHTHLFIQFKNPVYFSS----VKKSFPT------AHIEEAQG
SAE-E----------NRAYLRK---TMPQTG--------QGRRSDLANLYQMIKDG--YSNVEILEIN-PDNLLNLQHID
KARLEILSNRYKAERRT---------------NLVVTYVSGATGYGKSRNILDNHG-------DYK----PFDTYSGE--
-D-VLVFEEFRSD--LPIGNMLNYLDIYP---LQLPARYN-NRQACYNFVYIVSNWILEDQYHNIRLE----QPETYKAL
MRRIHKV----
>pCRESS2_WP_013271491
ITCN----NPLDGFTSEKIKEIMQRKTY--ETG-ESGTHHFHLYTKFCHAQITDT----ISKTFGN------AHIEVIRN
SSSID----------NRDYIRKECPIDGKEN--------QGHRNDIDLMISLVQDG--ASNMEVVQAV-SSMALRIPAIE
QYRQAYWEEKGR-GYR----------------HMDIWYIYGKTRTGKTSYVYQSHH-------DYKNG--IWDKYDTT--
-R-VLLLDEYRSA--LPFSLILALCDGQP---LTLNCRYA-NRVCLHETVYIVSNISLLEQYPNIQRE----EPESWNAF
LARINHV----
>pCRESS2_WP_013978550
FTFN----NPAEGDTHESVAGRFSELSL-DEIGAETGTYHTHGFIYSPSPIRFTR----LKKLFPF------AHIEKANG
TCK-E----------NRDYVAK---ELPKER--------EPKEDRKERLYAMVEAG--LTTEEIIELD-KSFIFQANTID
GLIQRRLASRHKGVNR----------------SVAVIYIWGETGTGKTRSIQERHS-------SYRG-AVSFDAYKGE--
-P-VLVFEEFNSQ--IAIEEMLNYLDVYP---LMLPARYS-DKVACFTQVYITSNIPLEKQYPEVQRT----RPATWRAF
LRRIGKI----
>pCRESS2_WP_018597672
LTIN----NAVDGFTHEHIIELANTFKS-DEVG-ETQTHHTHVYLAFRSAVRFTS----LQKKFMG------AHFEVAKG
TSQ-Q----------NRDYVFK---EMPVER--------QGKRNDLEDLYDMIKQG--MDNYQILEEC-PQYMLNVDKIE
RCRQIVREEKYKNTWR----------------DLHVTYIYGETGSGKTRTVMEKYG-------DYD----PFDSYKGQ--
-D-VIAFEEFRSS--LRVRDMLNYLDGYP---VELPCRYA-NKVACFTQIYIITNIPLNEQYTDLQRA----QMETWQAF
LRRIHEV----
>pCRESS2_WP_020072285
LTIN----NPGK-WSHEKICAVLGKMQ--DEQGLQEQTPHTHVFLVANSAIRFST----VKGYFPT------AHLDPACG
TSE-E----------NRAYVQK---VLPTEN--------PGQRTDWDIALAMLEDG--HSAMDVIRVQ-THLMRYRSTLE
QIRQELIAEQFRDTFR----------------ILETTYIYGTTGLGKTRFVMERYG-------GYQG---CFDKYQSE--
-D-VIVFDEFSSS--LKIQDMNNFLDGYP---LMLPCRYA-NRVACYTRAYIISNIPLEYQYANVRLD----TPAVWNAF
IRRIHKV----
>pCRESS2_WP_021629801
LVIN----NPSEGLDHSAIKEILQRFSP-DET-ASTGTYHTHLFFYAPSPVRFAT----IKNRFPV------AHIEKAYG
TVQ-E----------NRAYIRK---EAPPER--------AEKHPEMFRLVQNIRDG--MTTTEIIDDN-PAMAFRVRDID
LLRQTLTAEKYAVENR----------------PLEVSYLYGASGAGKTRSIYEAHD-------NYRAKGISFDGYHGQ--
-E-VLVFEEFSGQ--VPIEDMLNYLDIYP---LSLPARYN-DKTACYTTVYITSNLPLEKQYRGEQWD----RPETWRAF
LRRIHNI----
>pCRESS2_WP_021882760
LTIN----NPDEKFSESEIENILNTFKF-REI-GENGTPHIHLFIYAKSRIRFST----IKKRFPT------AHIDKAYG
SVV-D----------NIAYITK---EAPSAL--------EEHSPELSQILDDIVSG--MSTSEIITEY-PQHIFRVNAID
TVRQTFLADKYRERMR----------------SVCVTYIHGASGVGKTRGIYKHFP-------SY-SNGVKFDSYCGQ--
-D-VLVFEEFASQ--IPIEEMLNYLDVYP---LMLPARYT-DKVACYTKVIITSNLPLNKQYVNEQIE----KQKTYNAF
LRRINYV----
>pCRESS2_WP_023977019
LTIN----NPVDGFTHEVLKEKLKEFKN-DEIG-ENKTYHTHVFIACSGAVRFST----MQNRFKG------AHFEMARG
TCK-Q----------NREYVFK---DCPVER--------QGQRNDLIDLYDMIKGG--MTNFDIIEDN-PSYMLEIDRIE
KVRQTVRDEQFKNTFR----------------ELEVTYIFGSTGSGKTRGVMEYFG-------DYD----PFDSYKGQ--
-D-VVVFEEFRDS--LKISDMLNLLDGYP---LELPCRYA-NKIACYTKVYIITNLDLNDQFKGVQVK----HPETWKAF
LRRIHKV----
>pCRESS2_WP_024346025

MTIN----NPADGYTHEYIQKTLSTFKS-DEVG-EQGTPHTHVFACFSSAVRFSM----IKKHFPT------AHIESAKG
SIA-Q----------NLDYIKK---ERPPEN--------LGKDKDLETLYHMIDEG--LSNAEIIRIN-QDYIMQIDKLD
KIRTTHLQDKFKGERRL---------------DLEVTYMFGATGTGKSRGILDEYG-------DYD----PFDHYSCE--
-P-VLVFEEFRSG--LPLSDMLNYLDIYP---ITLKARYS-NKFACYTKIFVVTNWELEKQYAERQIT----DRASWRAF
LRRIHKV----
>pCRESS2_WP_036328238
LTIN---YKEDPTNNDE-LLDYIKDIKS-LEQGE-KGTKHHQIYISFEHAKSFET----IKKYFPK------AHIEAMKG
TPE-Q----------ASEYCTK---ELPIK----------GKRTDLEDIYKMIASG--FSDMQIRETYPSQYIRYNHKFK
EIRQEILEEQFNTLFR----------------KIDVVYLVDLPGTGKTRYIMEKYG-------NYK----PFDTYKGE--
-D-VIVFEEFRSK--LPIENMLNYLDGYP---TRLPARYG-DKVACYTKVYIVSNWEYTEQYKNIREL----YPTTMQAL
DRRINFV----
>pCRESS2_WP_037404274
LTIN----NPIENLKHNTIKEYLSNLDN-DEIG-QEKTYHTHVYIHLKNAIRFST----LKAMFTT------AHIEKANG
TAI-Q----------NKEYILK---IIPEES--------QGKRTDLIEIFEMAKDG--IKTVDILSEY-PSALLYIDKIE
KVRTEIQREKFKNTFR----------------KLDVTYIYGKTGAGKTRYVMDSFG-------DYK----MFDSYSLQ--
-D-VIIFEEFRSS--VHVKDMLSYLDGYP---IDLPCRYS-NKVACFTKVFIISNIDLLAQYTDIQRK----EVETYRAF
LRRINKI----
>pCRESS2_WP_038278663
LTIN----NPTDCFSHDKIKEYLVTFKS-DEIG-EQGTPHTHIFICYRSPVRFSM----IKKHFPT------AHIEAVAG
SIE-Q----------NIDYLKK---DRPPEN--------LGKDKDLEALYHMVDEG--LSNAEIIRLN-NDYIMQIDKLD
KIRTTYLQDKFKGERRL---------------DLSVEYTFGSTGAGKSRGILDEFG-------DYD----PFDHYSCE--
-P-VLVFEEFRSS--LPLSDMLNYLDIYP---ITLKARYA-NKYACFSRIFIVTNWELEKQYAERQIT----DKESFQAL
LRRIHKV----
>pCRESS2_WP_038350939
ITIN----NPLEGFSHDYIKAQLEKFKS-DEVG-EQETFHTHIYMACSNAVRFST----VKNRFEG------AHFEMAQG
TSQ-Q----------NRDYVFK---ELPIER--------QGQRNDMADLYDMIKQG--YSDFEIMEES-PAFLMNIDKIE
KARQIITSEKYKNTFR----------------ELEVTYIYGKTGSGKTRSVMEKYG-------DYQ----PFDNYHSQ--
-D-VVIFEEFRSS--LKIQDMLNYLDGYP---LELPCRYA-NKYACYTKVYIITNIPFEEQYDNIQHV----SPETFNAF
KRRIHKI----
>pCRESS2_WP_044928503
LTIN----NPLEGYTHDAIKKNVCTLKS-DEVG---KTHHTHVYILFQSPVRFST----IKRLFPE------AHIEKAYG
SSI-Q----------NRDYIFK---EMPTER--------QGERNDLTALYELISEG--KSNYDILEEQ-PEFITQIERMD
KVRQIIQEESYKDIFR----------------NLEVDYLYGDTGSGKTRSIMEKFG-------NYK----PFDQYKGQ--
-D-VIMFEEFQSS--IHINQMLIYLDGYP---VTLPCRYS-DKVACYTKAYILSNIDLKEQYPDIQTY----SPETWKAF
LRRIHKV----
>pCRESS2_WP_044942941
LVIN----NPLEGLDHSTIKEILQRFSP-DET-ASTGTYHTHLFFYAPSPVRFST----IKNRFPT------AHIEKAYG
SVQ-D----------NRAYIRK---EIPPEQ--------AEKHPEMFRLVQNIRDG--MTTTEIIDDN-PAMAFRVRDID
LLRQVLTAEKYAVENR----------------PLEVSYLYGASGAGKTRSIYETHD-------NYRSKGISFEGYHGQ--
-E-VLVFEEFSGQ--IPIEDMLNYLDIYP---LSLPARYN-DKTACYTKVYITTNLPLEKQYRDEQWD----RPETWRAF
LRRIHTV----
>pCRESS2_WP_051546484
LTIE----NPKTGYTPHKLKSMIKKMKS-YET-GECGTEHVHIYLSLKNSIRFST----IQNHFPS------AHIEKSEG
NHD-E----------CIAYIQK---KRPEVK--------RRRKKKMRSVLDLIRTG--KSNLEIVQIY-PSFISKMKALD
EIRQEFLKEKYGHTNR----------------HVECTYIYGDTRTGKTSDIIKKYG-------SYAP----FDGYRGQ--
-D-VLVFDEFRAENAMPFSNMLTYMEGYP---LELPARYG-NKVACYTKVIVISNDPLKNQYTSVDRS-----SQSWMAF
LARFMSV----
>pCRESS2_WP_051600858
LTIN----FNGAPLTEDALIELIQMNTF-FEK-GEQGTLHVHIYIHSENPRRFST----MKMTFPR------AHIEKALG

SPA-E----------IRDYIKK---KIPTPG--------ASRSNNKNKLLEDITAG--KSTAEIIKDS-PDYIFKINCIN
TAREELLNTDHQNSFR----------------DVTVYYVYGATGTGKTYSIYQCYD-------DYPRNKVRFDAYMGQ--
-K-VLVLEEFRSE--IPISSMLNYLDRYP---LKLPARYY-DRQACFTTVIITSNIPLEEQYLAIQDV----QPETWRAL
IRRINYV----
>pCRESS2_WP_051639324
LTIN----NPQDGITHDSIKQSLQPYTD--ETG-AHGTYHYHVYIYFKNAIHFSS----LKKLFPT------ANIQQAMG
NSL-Q----------NRSYLLKSAHECPTEI--------RGKRNDLERMYELIKEG--YSNSEIIEACGKTAILHIEKLN
KLRHSYLIDYYKGTRRL---------------NLKVHYISGKTGLGKSRDILDEYG-------DYQ----PFDSYQNE--
-N-VLVFEEFRSL--IRLSDMLNYLDIYP---CVLPARYS-PKIACYDTVFIVSNWEFESQYYELQQD---PQITTYEAF
KRRINGY----
>pCRESS2_WP_052011064
MTIN----NPKEGFSHNEIHEILKNFKT-DECG-T--CYHTHLFLVFSSRVRVST----IHRNFEG------AHIEIARG
SIS-D----------NINYLKK---TRPPDS--------QGKRTDMSELYQYINDG--LSNAEILALN-QDYILNVDKLD
KVRNILLTNRFKEEVRL---------------DLQVIYISGATGTGKTRGVFEKDG-------DYA----PFDAYCCQ--
-E-TIVFDEFRNS--LKLSEMLNYLDIYP---VDLPSRYN-NKVACYRKVYIISNWKLEQQYSYEQIN----DRESYLAF
LRRIHKV----
>pCRESS2_WP_053167095
LTIN----NPTKMITHESIQEILATFKS-DEVA-S--QHHKHVFLAFSSPVRFSQ----IKKYFPS------AHIDKSRG
TAE-N----------NRHYVFK---EIPNER--------SGNRTDLAELYELIQDG--FSNAEIIEQN-PDNILFLQHID
RTRKAILEEKFKSNWR----------------NLDVTYIFGPTGTGKSRHVMEKYG-------DYL----PFDSYRQQ--
-D-VIVFEEFTSS--LKIQDMLNYLDGYP---LELPSRYS-NKQATFTKVYILSNSPLRKQYETVQIE----KIEVWRAF
LRRIKKV----
>pCRESS2_WP_066546553
ITCN----NPEEGLSSDRIKEIMQRKTF--ETG-EQGTDHFHLYVKFVNPQSTRV----LSKAFGN------AHVEIIRS
SSSSQ----------NRDYIRKECPDESEEI--------QGRRTDIERMISLVQDG--ASNVEIVQAV-PSMALKISALD
QYRQAFYEEQGK-QYR----------------DVTTIYIYGRTRTGKTSSVYANHD-------DYKTG--VWDQYDTT--
-R-VLLLDEYRSS--LKISELLAICDGQP---HTLRCRYS-NRVCLHDTVYIISNISLLQQHKEIQRD----EPESWEAL
LARIKIV----
>pCRESS2_WP_066550639
LTWN----NPQDGETHDKLKEILMKWGDSDEIG-ETGTPHTHMFVCYQNAVRFSS----IKDSYPS------AHIEVAKG
SPE-S----------NRAYIRK---FIPKEG--------QGRRNDLNQLYEQIKAG--YTNAELLEND-PDNMLRLSYID
RTRNELLIEKYKGTRRL---------------DLQCIYVFGETECGKTKTILDEHG-------DYK----PFDHYAME--
-D-VLVFDEFRSD--LPIGAMLDYMDIYP---LQLPARYN-NKTACYHYVYLVSNWKLEDQYHDEQLE----HKSSWAAF
LRRIKKV----
>pCRESS3_AKO38848
FLTVPAEGEGVSRDE------LEQALEPEQGKG-TNQYRHWQLILVHPEPVRFST----LRRKLPT------AHLEPVR-
DLR-A----------SLAYVQKGPG--------------QGHRSDLDTLRSRILDGQETADELILSDTGA--WRHSRLVG
DLVSARDRSRQEK--LR---------------DVQVRVVFGDTGTGKTSAALSGL-------QHWAT----FDGYDGQ--
-D-SLVLDEFAG--QPPLTELLTWLDVFP---VTLNARYR-ARQAAFVRVVLCSNAPPWTWYPWAPKA---Q----RAAL
ARRLHLV----
>pCRESS3_KFI81686
MLTVPAEDHTQDEVR-----TLFERISTHETGA-TTGYEHFQCFLQMKSPMRFST----LKNHLTG------AHIEPRHG
SVE-D----------CVAYCTKKDR--------------QGRRSDLIAFREQILDG-VPVQQVLLDDTEAKAAHCTKWLN
AFSEACARQEYGN-KLR---------------DVSVHYLYGAPGVGKTRYVYDR--------YDYAP----FDEYDRH--
-R-VLVLDDYDS--QLPWEQLLSYLDRYP---VTLPARYH-NHQACFDTVWIISNLPLTAQYPDITGA---R----RLAL
LRRITDC----
>pCRESS3_KFI87454
MLTVSAE-QNEITEA-----DLVDAFEDREEGG-HTGYRHYQLFMQATSRIRLST----VRARLEK------NYIEPRRY
SVA-S----------CVAYVSKHED--------------QGKRTDLEELRDAVVKG-ASVNEILN-DPSLKAARFMPWLE

KMVGARQAARFSQ-EDR---------------EVTVHYLWGKPGLGKTRSVLDG---------NYEP----FDDYSGQ--
-S-TLVLDEFAG--QLPFQLLLNVLDRYP---CKLPCRFH-DTWAGWTTVWIISNKPLERQYQDVEPQ---V----RAAL
DRRITTN----
>pCRESS3_NP_613078
LLTIRRELPDERTVDDVVN-ALQGIFDAEKGEG---GYRHYQIFAQGK-RQRFST----LKKKLTA------AHVEPRKG
SVS-E----------AVGYCSKHEKESH-----------QGERSDLARL-KARAAG-ETVSQILLSEDGELAARYLGWLR
ATCDAAQAAKYRTKVRD---------------DLEVNFLYGETGVGKTSHVYES--------EDYAA----FDKYEGE--
-G-ILLLDEFTG--QFPMPLMLKLLDKWP---MQLPARYS-NRWAAFSRIWVVSNLPPNNLYSYAPES---Q----RRAF
FRRFA------
>pCRESS3_WP_002529618
MLTLPEEYYSRDIVE--------DKLRSESGK--ESGYRHFQIYVENKNAIKFET----LRSKFPR------GHYEPRRE
SKS-Q----------CLKYCTKDRL-PR-----------TKRDIS-AEISEKMKEN-VPASTLIQDPRY-AQFLKYIEAL
ETIR---LKNLGLEDRD---------------ALEVHYLYGPSRVGKTYKILHGLN------YNFKP----WDNYEGQ--
-S-VLLLDEFAG--QISFEFLLQVLDKYQ---LELNARYR-NKWACWTQVWIVSNLPMESLYRRVSPE---Q----WRAL
CMRFTSY----
>pCRESS3_WP_016667133
MLTIKAD-FTREEVE--------EKLKKECGE--ESGYLHWQVLIENKTAIKFET----LKNKFRT------AHCEVAKN
-LF-A----------CRKYVSK----PE-----------TKEGDAVSDLRHAIFED-KSADELILSDGRYRPYVAYAKEL
ERIR--DASKFGSP-RT---------------SVNVRYLYGAPGVGKTWGVYDEFG------YTYIP----WDEYQSQ--
-R-VLLLDEFDG--QIEFELLLKVLDIYP---LTLPCRYQ-NKYAAWDTVIMVSNNPLESLYRRVSAS---K----WAAL
LRRINVY----
>pCRESS3_WP_021975256
MLTISAEKHTRQDVE-----ELLDILG-QEEGG-KSDYPHFQAFLQLQTPVHMGT----LKNKFKK------AHIEMRKG
TVQ-D----------CVDYCSKKDQ--------------QGSRSDLAELRRQIMDG-ASVSEVLLNDDACQAARYTRYLS
ELATARDRVKYGR-QLR---------------DITVHYLWGDPGVGKTKYIYDN--------NDYRP----WDEYEGQ--
-S-ILVLDEFDS--QFSWDQLLVFLDRYP---VMLPARYN-NHVACFTTVWIISNEPLSKQYPERTGE---K----RNAL
LRRISTN----
>pCRESS3_WP_022856850
MFTLKIENDSTLTTLLG---MLGSWTHERGSKS-GYDHFQG--FLRCKSSNRFST----VKNHFIK------VHIEARKG
SPR-Q----------AYDYCTKLDLAT-------------GKRNDIEDAR-ELISG-LTPRKIMLADSEGRFAHLTTYIE
TYYQARLSNEYATK-ER---------------DVLPIYLYGETGSGKSRWVADN--------LDYTP----YDGYTDQ--
-K-ILVFDEFHS--QRPIEEMLRLLDPYP---VELPARYH-NKQACYRLVIVISNFPLATQYET--AE---Y-PQ-RAAF
QRRFSNS----
>pCRESS3_WP_023022037
MATISAEKH---REE------IEEALEE---KG-EEGYRHWQLLIDGNSPIRFST----LKNKLPT------AHLEPRRG
PIQ-Q----------AIEYATKGDE--------------RGRRKDVDIVREAVEKG-LSVDEIFLQVPEA--ARMTSFVE
RLVAARERAQNSA--PR---------------EVEVMWLYGPPGTGKTSLAVD---------IDYSP----FDSYAGE--
-K-TLILDEFDG--SMPLSLVLNILDVWP---MSLPARYA-NKAAAYTQAVMVSNESPWGYYLWEPAS---R----RQGL
ARRIDTI----
>pCRESS3_WP_025221073
CVTLEASS---ITLEDYVD-HVREFFDAELGEH-TTGIPHIQAFFQGK-PKRFST----VVRFLES------PYVDKRRG
TVK-Q----------AVSYAQKRES--------------QGKREDLLDLRKMVDAG-LTVDEILLEDVEGKAARYVGWLD
R-LVAARDAKKMSELQR---------------DLHCTFIWGKTGVGKTRYALEQG-------RDYRP----WDMVDDT--
-D-VIVCDEYNG--QLDLTEFLTILEGYG---ASMRARYR-NRWPNYSQVYVLSNTALNEMYSYEPSE---R----RRAL
FRRFDRI----
>pCRESS3_WP_033495900
MVTIASKYVCDWSVILD---ELNDMTAYAEEGT-GKRGMRHGHFIVYTPRMRMGT----FADHFPT------AHIDPVER
TPK-V----------VERYVTKYDTVDESEGF----PAGQGKRTDLKDAVKKVLAG-AIPDMLMRDDP--KLIAYDRYLD
RAYEIAMK-EKGRK-MR---------------DVHTLYVYGETGVGKSAWLYGKRHNVYR-LTDYAP----WDNYRGE--

-S-VVIVEDFDG--RMRLDDVLRWTDRYP---VELTARYA-NKHALYTTVIFTSNRPMSGWYGY--DE---FEPK-QGPI
NRRVSTVCASL
>pCRESS3_WP_036342632
MLTIPASKFDKKEVE--------NRLKKEKGKT-DTEYLHWQVFVHGTAAIRFDT----LRNKFDQ------VHLEPRCG
TIL-D----------CINYVTKDGN-AR-----------TKSRISNEEIYQEISGK-TAGQIINDHPELGMQFLK-IKAL
ENIKEEQFRGLQTEDRE---------------NIEVNYLWGPPGAGKSWHVLNEAG------YGYQV----WDNYQGQ--
-R-VLILEDFTG--QIGIEELLQVTDIYA---TELDARYS-NHYAGWEVVWIISNLRLDDLLKKYPKE---L----RPAL
VSRITNV----
>pCRESS3_WP_043170238
MLTIRAEGHTEDDVK-----ALFEKIGVREIGG-KTEYEHFQCFLQVKTPMRFST----LKNHLTD------AHIEPRRK
TVE-D----------CVNYCTKKDK--------------QGQRSDLIGFREQILGG-MSVQEVLLGDTEAKAAHCTRWLG
ELEAAYVRKEHGG-KLR---------------DLDVHYLYGAPGVGKTRYVYDK--------YNYKP----FDEYNRH--
-K-VLVLDEYDS--QLPWEQLLCYLDRYP---VTLPARYH-NHQACFTTVWIISNLPLSAQYPDIVGE---R----RFAL
IRRLTDC----
>pCRESS3_WP_052119337
MLRISKIEDYEDIEWSDICSAFHDAYGSLEKGT-KTGYLHFQVLLITKYEKKGQA----IIDAIAK------GGTEKLRK
NIY-A----------GVRYTSKGEQT-------------QGARNDLNELRRAIDDH-MTVDDILRDPDSIKSARYVSWLD
RLQRANSVTPHEATEQR---------------DVKAHYLYGSPRIGKTRLIYDN--------IDYQP----FDSYVGQ--
-K-VLVLDEYDS--QFPITSINNFLDRYG---CELPARYH-NSWANWDEVWVISNLPINSQYSDDNTD---K----KNAF
IARFTDI----
>pCRESS3_WP_052825216
MLTIPAGRHTEDDVA-----ELLERICSREKGE-ETDYEHFQCFLQIPSPMRWST----LKNHLAQ------VHIEVREH
SVE-S----------CVNYCSKQDQ--------------QGKRTDLSDLREKILNG-ASVEDVLLEDTESKSARYVKWLS
ELAAARDKKKYGR-QMR---------------NVEVHYLWGAPGVGKTSYVYER--------YDYQP----FDEYDRQ--
-P-VLVLDEYDS--QFDWEKLLCYLDRYP---LMLPARYH-NRQACYTTVWIISNKSLDEQYPLVQGE---R----RLAL
TRRLSDV----
>pCRESS3_WP_055838650
MLTLPES-EDQAEIE--------KRLGKEAAP--TTGYLHWQLYLETKSALRFST----LRKLFPK------GHYKPARD
TRI-Q----------CVRYCTR--T-LK-----------QGKRTDLDSYSEQILEG-KSADEVIHDDPRAVIYASHLWQL
ELIR---DRETWGKKFR---------------ELEVHYIHGGTRTGKTSALFETYG------YNWKP----FDGYRGQ--
-D-ILLLDEYNT--SVPMVDLLKLLEGYP---LEVSARYS-DKIAKFTTVFIVSNLELSEQHQTIQHE---HPKQ-WAAL
GARLTSV----
>pCRESS4_CBL40434
MQYEYNPTEDLHF-NRVIMKALAHK------TDGTPKAPHWHVYIYCNPAKSLDD----ISKWFGVPT----NMIE-LKV
GSFLDC---------AEYFTHEK--QPRDEKR----AKYDIQFRDLQR--VDVLNFGKTLKQCKIDDP-VLYVKEMQILK
KCRLEY--------LYTQP-VP---------KSRMNIYVTGQGGVGKGHTCKALARAFT-----IGAKNATFEGYDGQ--
-PVIIWDDRRDYELGRE--NVFNVFDTIP--QNLRQNIKYGSVKLLNAINIVNSVQPYTEFLDGLEYKA-
KDKSEDKQQS
YRRFPFC----
>pCRESS4_CDA18875
VTDVKHPTEELIN-KKIDTVLKSHASTY---TDGDIKPAHFHVVMRFARAQELDS----LSEWFGIDK----NFFE-KKK
GSFFDS---------VLYLTHQSD-KECEINK----EKYNICIKDKLR--LDVLYNGMSIRQVKKNYP-MEYNDDMEGLQ
KRRGDY--------LKDAP-LP---------PYRISFYISGSGGAGKGLFSEALARACY-----VGSDSVCFENYDGQ--
-PVLIWDDCRHNKLDRG--TIFNVFDTKP--KRISQKKKYSQTNLINPINIVNSVEPINGFLDGLQYYD-KSEAENKAQS
YRRFPIC----
>pCRESS4_CEI31812
TQNERYLTRVLMS-ERIGKAVGKKGMQY---TVGHRKADHFHIAVRRKSFSTLGQ----IARAFGVPP----NAVEI-KP
-AFMDL---------IEYLTHEHP-NQHKLAR----AAKAGSLKKRDAVREAVMLGEMTLKQVREDER-AIYIQDLDKLQ
KLRQDF--------MLHQP-AP---------RHRTNYYIGSPAGTGKTQLAKLFARMHV-----ATDARVPLQNYKGQ--

-PVIIWDDYSVPALSRE--GVWQVFDDHP--SASDVNIKYGSVRLVHAVNIIAKTTPYAEYLDGLEYTD-ASEAEDRNQS
WGRFPVV----
>pCRESS4_CRY93789
MQYEKHPTEKLID-EVIK-VALAHKSVY---SLGETKPKHWHIVCRCQAAVEVST----IARWFKIPE----NFVD-VPK
GAFLDC---------VEYLTHERE-EQRAEKR----LKYDTDPKTAMW--YDVMFNGLTLKQALERDR-WAYMEMLEKLK
KARLDY--------ISRMN-PP---------DTRINYYVEGKGGVGKGLISRAIARSFE-----VGAKGAPFEGYDGQ--
-PVIIWNDRRAYELGRG--NVFNVFDSHP--TKQRQNIKYGSINLCNEVNIVNSVQPYAEFLDGLEYED-KNGVEDKGQS
YRRFPFM----
>pCRESS4_CRY97508
LQYEKNPTADLHF-TENILKCVAHKSVV---TEGQPKGVHWHIVLETAGLMPVST----IARWLGIPE----SMVE-IPK
GAFIDC---------VEYLRHSDI-RQMVLRK----TKYPLSQADFLK--NEVLYNGMRLAEVQERYP-SIYMKEQTVFD
RLRMKY--------LVERAPLP---------ASRINFYIEGLTGYGKDTMARSIARGFE-----IGGKKVTFDSYDGQ--
-PVIIWSEFRAEALGYE--EVLGAIDIIP--KNNRHHKKFGAVKLINSVNIVTSTEPYAEFLKGLE------SDPDPSQA
NRRFPLI----
>pCRESS4_GAC78794
MQYRKNPSVVMLT-QQIDQGIKTLGGRR---LVEGVKGLHAHMVLWIAPRPSIRT----ISDAFAIPS----AVVRTPKK
GAFYDL---------CEYLPHESR-GSHMATR----HNAATDGAKLSKLYQAVGSGSLTLRQVREREP-AIYFAKIAHLQ
KCRDDY--------LLRAP-LP---------PFRTNYYIGGPARTGKSLYAETLARQYM-----VGRPGVAFQSYDGQ--
-PILIWDDYRPLAIQRD--SIWPVLDIDP--KRVQVNKKFGAVSLLNSVNIITGIQSYVEFLDGLEYTD-KKEVEDKDQA
YGRVPLV----
>pCRESS4_WP_000186194
IQYENNPTESLNFDENILNALAYFSHVI---TEGKPKGTHYHVVMELTNPASISA----IAKRFGVPA----QYVE-VIE
GAVLDC---------IAYLTHEDA-KQVDNQK----AREALTKRADARVIIEKISQGMTLSQVYEFDN-VMAVENKNLFK
TARQEY--------LKNAP-VP---------PVRTNYYVYGEGGTGKSLSAKVLARSFV-----VGDGAVPFDGYDGQ--
-PIIIWDDWRALHFDRS--LVWKLFAINP--ERISVNVKYGSTSLINAVNIVTCVDPYLKFMEELEYTD-KRKKEDSRQA
FRRFPFF----
>pCRESS4_WP_000818357
VQYEFNPTEDLHFNEVIKNAILNKETKY---VEGDVRPPHWHVLLKFKNQIEFST----IAKLFNVPE----NLVE-KKT
GAFFDY---------LYYMTHEDD-KQVDIRE----DRRAK-KLSKVEVFLDKLTSGMTMKQVFERDS-VVFAENATLFR
RARRSY--------LKYAP-TP---------LVRTNYHISGAGGTGKTLIAKSMARAFV-----VGDGRVAFDEYDGQ--
-PIIIWDDFRAKAFERG--TMWKIFAIHP--DKVSVHVKNGETTLINTVNIITSVEPFTEFVNGLAFKD-N-KSEDVGQA
YRRFPIF----
>pCRESS4_WP_005464724
MQYREHPTEVMLT-QQIDEGLAALGDRR---LVEGIKGLHAHMVLWVAPRPTIRT----VSDAFSIPS----ARVKPPKK
GAFFDL---------AEYLPHESR-GKHMAMR----RNAAER-AKLSKLFQAVGKGSLTLKQVRDQEP-AIYFAKLAHFQ
KLRGDF--------LAYQD-AP---------ESVMNFYVFGEGGTGKDLLAKALARAFK-----VGGENVSWEGYDGE--
-PVVIWEDTRVGTASRG--MLFRILEPDE--K-PIVNIKGSKTQLLNRVNIVTGPEGYEEFLRGLEYES-MQQAENLGQG
FRRFPVI----
>pCRESS4_WP_006681830
TQNLSHPTQTLIT-TQVGKALAKKGVVY---TSGSPKAPHVHVVIQRSSFASIAQ----VARAFGVPP----QCV---EP
-AFLDL---------VEYLTHENP-KQHKIAR----QEKGLLQRRRKEAALKVAGGEWSLDHVRKHDL-ELWSAPMSHLK
GLRADY--------LASLA-PP---------LEVVNFYVFGPGGVGKDLLAHALARSFT-----VGGSNVSFEDYDGE--
-EVIIWSDFRASACDRG--LLFRVLDAS---EKVIVNVKGSHTQLVNRVNIVTGPDDYKTFLNGLEYAT-NRTSENKDQA
YRRFPLI----
>pCRESS4_WP_007889993
MQYCKHPTEVLIT-EQIK-SLFDRRTDF---DEGDKKPPHFHVVFRTDRNTDLET----VADWLGIPV----QYVDGARD
GTFVDL---------LRYLTHESE-KEADIRE----ARYSP--KDYYR--HKVAYEGMSISEVIAENE-DAYLKDMTFLD

KCRSKY--------LAAFAKMP---------DLRINIYLDGAGGIGKNTASKAIAHVFE-----AGGANTSFEGYDGE--
-PVIIWNDCRSTRFERN--ELFDILDPHP--TDARHNIKFGSVRLTNPINIINGIEPYNKFLDGLAYVD-KRSGEDSSQA
YRRFPII----
>pCRESS4_WP_017824301
TTDCVHPTEVLLT-VTIEKRLSKSSVCF---TEGEHKPDHFHVVMQLKNQASVGQ----VARGYKLHP----GCVRK-KE
GTFLDC---------IEYLTHEHE-KQRVAAR----KQGFHAAKK-MKIRLAVMNGQMTLKQVREDEP-GVYVQDLEKLQ
KLQQDF--------RLHQP-AP---------RHRTNYFIGGMAGTGKTQLAKLFARTYV-----ATDPRVPLQNYKGQ--
-PVIIWDDYNALALGRS--GVWQVFDDHP--SATDVNIKYGATRLVHTVNIITKTTPYAEFLDGLEYTD-
KSKAEDRNQS
WGRFPVV----
>pCRESS4_WP_021639163
MQYEKHPTDELLT-EKIKSVVAKYKSVY---T-GTKKPKHFHIALKMKNCIELDT----VAKWFGILP----NYVE-IPK
GAFLDC---------VQYMTHERT-EQRDEMK----AKWELSDRQVMG--QRIMLEGLTLRQVKAEDP-LLYADNLEFFR
KMRGVY--------LSDLE-PP---------KTRINYYLCGDAGAGKGVMSKAVARAFE-----VGADNALFEGYDGQ--
-PVLIWHDRRAGELGRS--NVYNVFDTHP--SKQRQNIKFSSVGLINRVNIVNSVQPYVEFLETLAKDD-NDGAE-
KSQA
YRRFPLI----
>pCRESS4_WP_043534193
MQYHQHPTEVIFT-QQLDEGLAALADRR---LVEGLKGLHVHIVLWFKPRPTVRT----VSDALTVPS----PRVRVPNA
GAFYDL---------CEYLCHETR-GAHMATR----HDAAEG-AKLSKLFQAVGQGTLTLKQVRDQEP-AIYFAKLAHFQ
KLRGDY--------LAYQD-AP---------ESVMNFYVFGEGGTGKDLLAKALARAFK-----VGGDNVSWEGYDAE--
-PVVIWEDMRVGTASRG--MLFRILEADE--K-PIVNIKNSKTQLLNRVNIVTGPQDYEEFLRGLEYES-
MQEAENLGQG
FRRFPVI----
>pCRESS4_WP_044572803
VQDEKHPTAILLT-RRIEKVLAKRPNQY---TDGEYKVPHFHIAEKRKNEASVGQ----VARAYDVAP----QYVRV-KP
-AFLDL---------VDYQTHGLE-RQRVAKR----INSPRKQTPIDKLAMRIQEDGLTLRLAKEEDP-LSFNRAPGRME
KSRATY--------LRHLP-PP---------SSRINFYFEGEGGVGKDLLAKALARTFS-----VGGENVGLDGYDGQ--
-PVIIFEEARAGSMGRK--ELFAFMNPFP--EKQSLNVKYGATQPVNTITIFTGPDDYDTFLDGLEFID-KSKAENKPQA
RRRIPII----
>pCRESS4_WP_052038917
TQNLSHPTQTLIT-TQVGKALAKKGVVY---TSGAPKAPHVHVVIQRSSFASIAQ----VARAFGVPP----QCV---EP
-AFLDL---------VEYLTHENP-KQHKIAR----QEKGLLQRRRKEAALKVAGGEWSLDHVRKHDL-ELWSAPMSHLK
GLRADY--------LASLA-PP---------LEVVNFYVFGPGGVGKDLLAHALARSFT-----VGGSNVSFEDYDGE--
-EVIIWSDFRASACDRG--LLFRVLDAS---EKVIVNVKGSHTQLVNRVNIVTGPDDYKTFLNGLEYAT-NRTSENKDQA
YRRFPLI----
>pCRESS4_WP_067940518
MQYREHPTEVMLT-QQIDEGLAALGDRR---LVEGIKGLHAHMVLWVAPRPTIRT----VSDAFSIPS----ARVKPPKK
GAFFDL---------AEYLPHESR-GRHMAMR----RTAAES-AKLSKLFQAVDKGSLTLKQVRDQEP-AIYFAKLAHFQ
KLRGDF--------LSHQD-AP---------ESVMNFYVFGEGGTDKDLLAKALARAFK-----VGGENVSWEGYDGE--
-PVVIWEDMRVGTASRG--MLFRILEPDE--K-PVVNIKGSKTQLLNRVNIVTGPEGYEEFLRGLEYES-
MQHPENLGQG
FRRFPVI----
>pCRESS5_WP_014735272
SYTYQNNT-EDAFREKIIRLVKALCTH--IDQNGELKSLHAHIYFEFKNSRFYSS---LFKTLEISRD----KNLQVVKD
K-----------AKVCRYLTHRNEKDSKRADK----KKSEEEIDCYLEISYQICEQGLLLNQAFEEFDQQLWNRNKKQFE
INRQEY----IDKEFVRMSKGN---------RNHIGLYIQGSGGTGKTTLARFLAEEQ-----HAP-STDFGSGYDGQ--
-RTIVINEFDASGISFR--ELFQILEPDA--VTQLSSRFKDAHI-INDLTIMTNSDDFIEWADAWKKKE-YHQLMRRIPF
FVKLNG-----

>pCRESS5_WP_017368666
NYDYEHD--TKEQWKEKITQLIASNVS--LLTDGLPKPLHVHILVGFENGKTQTA---VMKIFNVSRP----KNCQHTNT
I-----------AGSARYLTHRTEQA----------EKVNNIDEFVAELSEKIYLGKLYPTNIFKEFDDRLYKKYRKEFE
KDFQEY----LQHKGEEYKIKG---------RDLATIYIWGVSEGGKSHIAKRMGLLH-----LIPASGDPSGLYQGE--
-EVSIFNEISGKEFNNK--EFLGLADPRN--YSPINSRGKDKHW-LASYLFLTSTDSRETFIKNLSFEQ-RQRHEIARRV
PYEIRCV----
>pCRESS5_WP_018166163
SYTYQNDT-EEAFREKIIRLVKTLCTH--LDENGELKSLHAHIYFEFKNSRFYSS---LFKTLEISRE----KNLQVVKD
K-----------AKVCRYLTHRNEKDSKKADN----RKSEEETDFCLEISYQISECGLLLKQAFEEFDQQLWNRYKKQFE
INRQEY----IDKEFIRMSKGS---------RNHIGLYIEGSGGTGKTTLARFLAEEQ-----HAP-STDFGSGYDGQ--
-RTIVINEFDASGISFR--ELFQFLEPDA--VTQLSSRFKDAHI-INDLTIMTNSDDFIEWADAWKKKE-YHQLMRRIPF
FVKLNG-----
>pCRESS5_WP_024382134
SYAYQNDT-EEAFREKIIRLVKALCTH--IDQNGELKSLHAHIYFEFKNSRFYSS---LFKTLEISRD----KNLQVVKD
K-----------AKVCRYLTHRNEKDSKRADK----RKSEEEIDFCLEISYQISECGLLLNPAFEEFDQQLWNRYKKQFE
INRQEY----IDKEFIRMSKGN---------RNHIGLYLEGSGGTGKTTLARFLAEEQ-----HAP-STDLGSGYDGQ--
-RTIVINEFDASGISYR--ELFQILEPDA--VTQLSSRFKDAHI-INDLTIMTNSDDFIEWADAWKKKE-YHQLMRRIPF
FVKLNG-----
>pCRESS5_WP_024389873
SYAYQNDT-EEAFREKIIRLVKALCTH--IDQNGELKSLHAHIYFEFKNSRFYSS---LFKTLEISRD----KNLQVVKD
K-----------AKVCRYLTHRNEKDSKRADK----RKSEEEIDFCLEISYQISECGLLLNPAFEEFDQQLWNRYKKQFE
INRQEY----IDKEFIRMSKGN---------RNHIGLYLEGSGGTGKTTLARFLAEEQ-----HAP-STDLGSGYDGQ--
-RTIVINEFDASGISYR--ELFQILEPDA--VTQLSSRFKDAHI-INDLTIMTNSDDFIEWADAWKKKE-YHQLMRRIPF
FVKLNG-----
>pCRESS5_WP_024390948
NYKFEHDDTEDDWKNEIKKQFEAVSDP--IDTDGEKKALHVHFVARFENAIYYDT----TIEKFKCEP----RNFEKGRS
E-----------TSALLYLTHTTSEAE---------GSNK--DEDVARIIDELSEGLMTIDDVKQAFDTMTWMKNKRYFK
EAVAEY----YQNKYYDWLEKG---------RTFQLVYIQGSSGIGKTSFAREIGKEH-----NAPNDTDFLSGYENE--
-AVTVFDDLRPNTFGYT--EFLNLFEKER--VSKYSSRFNDKAW-FAEVAVITKSTSINDWTSKLELKS-ASKPNVLYQP
RRRFSLI----
>pCRESS5_WP_024393234
NYDFEHSDSKDDWKEKIKKELFEID----IKESNELKALHCHFVIRFNNPRSYSN----ILELTKCEE----RNFERSTN
E-----------GAILRYLTHTTPEAI---------GKKEKVVDFVNDLAYRLSVGEFKPINAIAEFGQSIYRKEKKKFQ
EDYADF----LETKKKDLLLNG---------KELSTIYIDGFSEVGKSTFAQDLANAY-----LAAKKKDWISKYKDE--
-YITIFNDVDPYDFNFT--YFLGTFETKI--LVDVGSRYKDKTW-FSEYAIITKSTDIHEFVNKIELRE-DNHFNIRYQV
QRRFSLI----
>pCRESS5_WP_024399566
SYAYQNDT-EEAFREKIIRLVKMLCTH--LDENGELKSLHAHIYFEFKNSRFYSS---LFKTLEISRE----KNLQIVKD
K-----------ARVCRYLTHRNEKDSKRADK----RKSEEEIDFCLEISYQISECGLLLNPAFEEFDQQLWNRYKKQFE
INRQEY----IDKEFIRMSKGN---------RNHIGLYIEGSGGTGKTTLARFLAEEQ-----HAP-STDLGSGYDGQ--
-RTIVINEFDASGISFR--ELFQILEPDA--VTQLSSRFKDAHI-INDLTIMTNSDDFINWTDSWKKKE-YHQLMRRIPF
FIKLNS-----
>pCRESS5_WP_024408358
SYEYENNT-EDDFKQSVEKRIKELCQN--INEDGTPKYLHVHFVIIFKNAHSYQS---VYNALKISRQ----ENLEVVRS
S-----------IKACRYLTHRNERNIKNHST----EKSEEVDEYCIELSYLISSDGLLPQEAFEQFTQKAWNTNKRTFE
ENRLEY----IQQEFEHMSRGE---------RNHNGIYIQGDGNSGKSFLARLIAEQA-----HVP-SIDLGSGYKGQ--
-KTMIINEFDASGMAYR--ELFQILEPDS--ANQLSSRFKDAYI-INDMTIITNSESYWDWVDDWKKKE-
WHQLMRRIRY
VIKMDQN----

>pCRESS5_WP_029171254
SYEYENKT-EDDFKKSVEKRIKEYCQH--INDEGTLKPLHAHFYIEFKNARPFSP---VYKALKISRE----ENLEFVRS
S-----------IKACRYLTHRNERNIKNHSK----EKSDEVDDYCLELCYQISSEGLLPEEAFEQFTQKAWNTNKRQFE
ENRQEY----IQQEFERMSRGE---------RNHNGIYIHGLGNSGKSFLARLIAEQA-----HTP-SIDLGSGYKGQ--
-KTIIINEFDGSGMAYR--ELFQILEPNS--ANQLSSRFKDAYI-INDLTIMTNSETYWDWVDGWKKRE-YHQLMRRIRY
VVKMDQN----
>pCRESS5_WP_029176301
NYKFEHDDTEKDWREKVSQELENV-----IDTDGEKKALHVHFVARFANPMDYEP----TREKFGCEP----RNFEKGRS
E-----------SSALLYLTHTTPESA---------GSLK--DDDVASIIDELAEGTMLLTDVKQRFDTMTWMKNKRYFK
EAVAEY----YQDKYLEWLEKG---------RTFSLIYLEGPSAIGKTKFANKIARRH-----NAPNDTDFLNGYEQE--
-VVTVFDDLNPKTFGYT--EFLNLFEKER--VAKYSSRFNDKAW-FAEVAIITKSTSIDSWTTSLELKT-DKTANILYQP
RRRFSLI----
>pCRESS5_WP_033583888
SYEYENKK-ECDFKKSIDKRIKALCKH--KKEDGTPKCLHVHFVIIFKHAHSYQS---VYKSLQISRE----ENLEFVRS
V-----------IKACRYLTHRNERNIKNHNK----EQSDEVDEYCLDLSYQISSDGLLPLEAFEQFTQKAWNQNKRQFE
ENRLEY----IQKEFERMSRGE---------RNHNGIYIQGSGNSGKSFLARLIAEQA-----HIP-SIDLGSGYKGQ--
-KTMIINEFDASGMSYR--ELFQILEPNS--ANQLSSRFKDAYI-INDLTIMTNSESYFDWVDAWKKRE-YHQLMRRIRY
ILKMDYN----
>pCRESS5_WP_038978316
NYEYDHEA-TEHEWRKQMVDMFTAIQD--VLPNGDKKGLHVHFVIKFKNPRVIRS---IMKTFGISRT----ENISKVKS
V-----------KGSLSYLLHITKQAK---------DDKDIIVEEVLSQVTSLREKGEMAD--YKKFPSDIYYNYTTKRE
YAEREY----FADKLRYRNKHG---------RWLRNLYITGKGGTGKTTLANKLGYAH-----VGAAKSDPMGTYKNQ--
-KVTILNEMQGSLFDYR--EIMNVFDDHQ--QAPVSSRTKDINW-TADYLIMTSSKSFERFRNETELPT-GQYKDVAFQF
TRRFSNY----
>pCRESS5_WP_039694423
SYEYENNT-EDDFKKSVEKRIKEYCQN--TNNDGTLKTLHAHFYIDFKNARPFST---VFNALKISRQ----ENLEFVRS
S-----------IKACRYLTHRNERNIKNHSK----EQSEEVDDYCLELSYQISSEGLLPLEAFEQFTQKAWNQNKRQFE
ENRQEY----IQKEFERMSRGE---------RNHNSIYIQGDGNSGKSFLARLIAEQA-----HTP-SIDLGSGYKGQ--
-KTMIINEFDASGMAYR--ELFQILEPDS--ANQLSSRFKDAYI-INDLTIMTNSEKYWDWVDDWTKKE-
YHQLMRRIRY
IIKMNHN----
>pCRESS5_WP_044771983
SYDYENST-EVDFKQSVEKRIKALCSN--QKDDGTPKPLHAHFYIDFKNPRAYSA---VFKALSISRQ----ENLEAVRS
S-----------IKACRYLTHRNERNIKHHSK----EKSDEVDDYCLELSFQISSEGLLPLEAFEQFTQKAWNQNKRQFE
ENRQEY----IQKEFERMSRGE---------RNHNSIYIQGDGNSGKSFLARLIAEQA-----HTP-SIDLGSGYKGQ--
-KTMIINEFDASGMVYR--ELFQILEPNS--ANQLSSRFKDAYT-INDLTIITNSENYWDWVDDWKKKE-YHQLMRRIRY
VIKMHQN----
>pCRESS5_WP_046467524
SYDYDTVA-TPDEWMDKIIAQFKKEGA--YLPDGTLKSLHVHIVIHFKNPRTVGA---VYKAFGVSRF----ENISKAKS
I-----------KGALKYLLHITPQAKNQEEA----EEKRVVIARCKSLLTDIATKGTAPGEWWEDFESDVYVNYKKKFQ
EMEKEY----FYRLTQKKKREG---------RDLRNIFVSGEGNSLKSSVAKEIALRH-----IASPPSDFVSLYKNE--
-KVSILNEMVSDAFNPR--EFMNVFDDFQ--IGSVSSRFKDINW-LADKTVMTTSDTFSEFRSNTE-------KDVFYQF
SRRVEHY----
>pCRESS5_WP_049478725
SYEYENKT-EKDFRESVIQRIHDYCKN--LKDDGSSKPLHAHFYIDFKHAHTYSS---VLKALSISRK----QNLEFVRS
S-----------IKACRYLTHRNERNIKNHSK----EKADEIDDYCLELSYLISEEGLLTTEAFEQFTQKAWNSNKRQFE
ENRQEF----IQKEFIRMSKGE---------RNHTGIYIQAEGGTGKSYLARLLAEEA-----HTP-SIDLGSGYKGE--
-KTIVINELDASGMTFR--ELFQILEPDS--ATQLSSRFKDAYI-INDLTIITNSDTYWDWCDSWKNKE-YHQLMRRIRF
VIKMDKN----

>pCRESS5_WP_049481849
SYEYENKK-ECDFKKSIDKRIKALCKH--KKEDGTPKCLHVHFVIIFKHAHSYQS---VYKSLQISRE----ENLEFVRS
V-----------IKACRYLTHRNERNIKNHNK----EQSDEVDEYCLDLSYQISSDGLLPLEAFEQFTQKAWNQNKRQFE
ENRLEY----IQKEFERMSRGE---------RNHNGIYIQGSGNSGKSFLARLIAEQA-----HVP-SIDLGSGYKGQ--
-KTMIINEFDASGMSYR--ELFQILEPNS--ANQLSSRFKDAYI-INDLTIMTNSESYFDWVEAWKKRE-YHQLMRRIRY
ILKMDYN----
>pCRESS5_WP_049535277
SFEYENET-EEDFRESVIQRIKDYCKN--LKDDGSSKPLHAHFYIDFKHAHTYSS---VYKALSISRE----QNLEFVRS
S-----------IKACRYLTHRNERNIKNHSK----EKADEIDEYCLELSYQISEEGLLVMEAFEQFSQKAWNSNKRQFE
ENRQEF----IHKEFIRMSKGE---------RNHKGIYIQAEGGTGKSYLARLLSEEA-----HTP-SIDLGSGYKGE--
-KTIVINELDASGMTFR--ELFQVLEPDS--ATQLSSRFKDAYI-INDLTIITNSDSYWDWCDSWKNKE-YHQLMRRIRF
VIKMEKN----
>pCRESS5_WP_050238550
NYEYEHTETESEWKQRILEELFSIS----IKEHNELKGLHCHFVIRFDNPRSYDS----ILELTSCQE----RNFQRSTN
Q-----------GAILRYLTHTTPEAL---------GRKEKVTDFVADLAYRLSIGEFKPHTAIAEFGQSIFRKEKKKFQ
EDYNDF----LDSKKRDMLLNG---------KELSTIYIEGPSEVGKSVFAQDLANSY-----LASKHNDWISKYKDE--
-FVTIFNDLDANLFSFT--DFLGTFEQKI--LVDVSSRYKDKTW-FSEYAIITKSSDIDEFVNKLEIRE-DNHQNIRYQV
QRRINLI----
>pCRESS5_WP_053863690
SYTYQNKT-EDAFREKIIKLVKALCTH--IDQNGELKSLHAHIYFEFKNSRFYSS---LFKTLEISRD----KNLQVVKD
K-----------AKVCRYLTHRNEKDSKRADK----KKSEEEVDCCLEISYQICDQGLLLNQAFEEFDQQLWNRNKKQFE
INRQEY----IDKEFAHMSKGN---------RNHIGLYIQGSGGTGKTTLARFLAEEQ-----HAP-STDLGSGYDGQ--
-RTIVINEFDASGISFR--ELFQILEPDA--VTQLSSRFKDAHI-INDLTIMTNSDDFIEWADAWKKKE-YHQLMRRIPF
FVKLNG-----
>pCRESS5_WP_058211405
NYDYEHS--TAEEWKNKIRQLIAFNT---ILEDGLPKSLHVHILKNFKEAKTQTA---VMKMFNVSRE----ANCTNARS
I-----------TSSARYLTHRTSQAS---------DRTIEVDEIVMGLSINIGNGKLYWLKAIDKFDIKLWNKYSRIFE
KNFKEY----IQHKAEDYKLKG---------RNLTTFFIWGDSEVGKTWLAKCMCLLH-----MVPASGDIAGLYDGE--
-KASLWNEVSGLELSNK--EFLDRFDPKT--YSPSNSRGKDKHC-LSDYFFLTSTDDLETVVNNLEFEI-KRRHEINRRL
PIEIKCI----
>pCRESS5_WP_061417941
SYEYENET-EEDFRDSVIQRIKDYCKN--LKDDGSSKPLHAHFYIDFKHAHTYSS---VYKALSISRE----QNLEFVRS
S-----------IKTCRYLTHRNERNIKNHSK----EKADEIDEYCLELSYQISEEGLLIMEAFEQFTQKAWNSNKRQFE
ENRQEF----IQKEFIRMSKGE---------RNHTGIYIQAEGGTGKSYLARLLAEEA-----HTP-SIDLGSGYKGE--
-KTIVINELDASGMTFR--ELFQVLEPDS--ATQLSSRFKDAYI-INDLTIITNSDTYWDWCDSWKNIE-YHQLMRRIRF
VIKMEKN----
>pCRESS5_WP_061863770
SFEYENET-EKDFRESVIQRIKDYCKN--LKDDGSSKPLHAHFYIDFKHAHTYSS---VFKALSISRE----QNLEFVRS
S-----------IKTCRYLTHRNERNIKNHSK----EKSDEIDEYCLELSYQISEEGLLITEVFEQFTQKAWNSNKRQFE
ENRQEY----IQKEFIRMSKGE---------RNHSGIYIQAEGGTGKSYLARLLAEEA-----HTP-SIDLGSGYKGE--
-KTIIINELDASGMTFR--ELFQVLEPDS--ATQLSSRFKDAYI-INDLTIITNSDTYWDWCDSWKNKE-YHQLMRRIRF
VIKMEKN----
>pCRESS5_WP_061866456
NKQYEHQESDTDWKEKVKSETFYLATE--LTSDGERKGLHCHMILEFRNPVTITSLEKFKFEAGKFQS----
RNVEASKS
S-----------SGSYRYLTHTTDKAI---------RPEK--DEALQEAFFKVRTGEIFDEEVRERFTTELVIKNKKFID
NSRQMY----QKEVFEDMQNNG---------RNLKTFFISGSSGLGKSRFAKDLARRY-----TAPTAKDFISEYKAQ--
-DVTIFDDVDAKSFGFQ--EFLNIFDKDN--ITKISSRYTNKAW-VSHYAIITKASKIRNWIERVEYEK-DKE----VQV
SRRFDLW----

>pCRESS5_WP_067193806
SYDYENST-EDDFRKSVEQRIKELCQH--LKDDGTPKPLHAHFYIEFKNPRVYST---VYKALQISRQ----ENLEFVRS
T----------IKACRYLTHRNERNVKNHSV----EKSDEVSDYCLELSYKISEDGLLPSEAFEQFTQKAWNSNKRQFE
DNRQEY----IQKEFIRMSKGE---------RHHNGIFIFAEGGTGKSFLSRLLAEEA-----HTP-SIDLGSGYQGQ--
-KTIIINELDATGMAYR--ELFQILEPDS--ANQLSSRFKDAYI-INDLTIMTNSESYWSWCNSWNKKE-YHQLMRRIRF
VIKMDTN----
>pCRESS6_ABJ73998
VQQLEDKHWTAENCRPLLEEVVTRLSST--ISVDEPKANHVHFLFKFDKGASLQK----LALAMGIES----QYLEKLKS
GGYDN---------CQAYLVHAKD--MKSWIR----GRAKQENLSVDWIIAEVLAGQLTKNQILLTDEYKVYGQHKRKIN
EALDTAGERKSYKTVAELEAGK---------FKKTVIFVKADSGIGKTALSKKLIGLC------VTASTNAFDEYNGQ--
-DILFLDDIRGDSLTVS--DWLKLLDPYM--ISPISARYHNKLG-SAKVIIITSTKHPFKFFENAK----GIVGEDLGQF
IRRIDYL----
>pCRESS6_ADX23728
VQQLEKDFWFFEDLIPLLEEIEKRLVSK--LVIEELKASHVHILIKFEKGTTLSQ----LAYILGIEA----QYIEKAKS
GGYDN---------LLAYLVHSKD--KQIWFK----GRATKENLSLDYVISEILEGHITKSQVLLTNDYKVYALNQRKIE
DAFAAYQEKKGFVTIQSLENRE---------FRKTIIFITGKTASGKTSLAKEIIKSC------ITASTNSFDNYNGQ--
-EILFMDDVRGYGLTAT--DWLKLLDPYN--ISPISARYKNKLG-YAKVIIIASSVEPSLFFHSAK----NYHYEDPSQF
IRRLDAL----
>pCRESS6_AEU41945
TQQFKADMWAVVDTARIMKRVAERLYKS--FAIVP-KELHMHAVIELPSKRDLSF----ISTAIGIRP----EQIEVPR-
GGREN---------MLAYLVHAKD--VDTWNY----RKATKASVKADWLVKEVQAGRVSKETIMLTDDAEVYADNMRAIN
DAFQYYAERQGFKTLEALRNNE---------FEMSVYYIQGAPRMGKTFFAKKFVNDY------ETADTQPMDDYAGE--
-EIVFMDDLRASAMTAS--GWLKLLDPLT--TAPMSARYKNKQK-ATRVIVITSYLDPFTFFSYVKG-V-GGSNEALEQF
IGRLSLI----
>pCRESS6_BAM66968
EQYFDSEFWKSENKDKIFQEIYDRLKF---RKNGQLIDPHVHVYVELPTKRSLEL----VADRIGISQ----HFIDANGR
NFSFN---------QKAYLIHAQQ--KDDFLK----RSATKENESLDLIFDKVLFGQITYDEIMEDDSYRLYANNEQRFI
SAFNSFSQYKARKTLKALRNCE---------FKLSVVYIQGNSGIGKSHLAQEIVEKY------SASSSNPFDEYKGE--
-EIILLDDLRPESMERA--DWLKVLDPMN--RSRISARYRNKVI-AARVIVLTNTETAESFFKNIKN-------EDLDQY
VRRINLN----
>pCRESS6_CGE81062
VQQLKKDYWFPKNCLPLLEKIVEKLEQE--TSVLEKKEEHVHVLLKFENGASLNK----IALATKVKS----QYFEKLKS
GGYDN---------CLAYLVHAKD--METWIR----GRATRDKLSVDWLIEQILTGKINKRTIMLSNEYTIYGQHKRKIN
EALDTAGEHKSYRTVAELEAGK---------FKKTILYITAESGAGKTQFAKRLIKLC------VTASTNAFDEYNGQ--
-EILFLDDIKGDSLSIS--DWLKLLDPYM--ISPISARYHNKMG-SAKIIIITNTKEPISFFEHSK----GSVGEDLGQF
VRRIDYL----
>pCRESS6_CMU27730
TQQFSNGFWELD-IEKILEEIVRRVSTS--I-VTKPAKSHIHALLKFDKGATLST----LSKKIGLAE----QHLEKAKS
GGYDN---------LLAYLIHAKD--KLSWLK----GKAKRQYEDIDLLIDNILNESITKNEILLEQKRTLYAVHKARIN
DTFRTVGEIKGTRTKYELDNEE---------FKKTILFIHGSTGLGKSKFAKELTKDV------VTAATNIFDEVNGE--
-EILFLDDVRGDSLTAS--DWLKLLDPFN--ISPISARYQNKMG-AAKVIIITSSKYPLDFFYDTK----GNDREDLSQY
VRRIECL----
>pCRESS6_EOB33201
EQQLKPDFWKKENKIEIFKEIYERVR----ISYTKLVEPHIHGYIEFSNKKDLNV----LALSLGILP----QYIESSGR
GGKIN---------SKAYLIHAKD--REDFEK----YAATEKDESLDLTLSKIQLGELTYNDVMEDDSAFLFGNNQQKFR
EGFNFYGERQTFLRLKSLERKE---------YQMTVIYIQGDSDIGKSELAKNIALQY------SASSSNPFDNYLGE--
-DILLLDDLREDTMRAS--DWLKLLDPLN--SARMSARYQNKLV-VPRLIIMPVYMSPKLFFGRIKA-------EDLNQF
LRRINFL----
>pCRESS6_KEQ49321

VQQLENEHWTAENIRPLLETVTDKLNKE--INVIEKKAEHIHFLFKFEKGASLNR----IALAVGVEP----QYLEKLKS
GGYDN---------CLAYLVHAKD--MATWVK----GRATKESLSVDWLIEKILVGEVTKRNIMLTNEYAIYGQHKRKIN
EALDTAGERKSYQTIDDLESGE---------FKKTILFIKAESGVGKTLYSKKLITLC------ITASTNAFDEYNGQ--
-EILFLDDIRGDSLNVS--DWLKLLDPYT--ISPVSARYHNKMG-AAKLIIITSTKEPLSLFKQSK----GNTGEDLGQF
VRRIDYL----
>pCRESS6_KST89836
TQYQQADFWKIRNQAKIFNEIYNRLKL---KNAGELKHPHIHGYIEFEKLKTIAS----VSACLGIEP----QYVEVPRK
GGRLN---------CLAYLVHAKN--LEDFKK----YNATKKEIGLDLALQEVQQGKLKLREIMRDENALLYANHMNQFN
DSFNFYGLRNAMLRLDELEQGK---------YNLTVLYIQGAPGIGKSFLAREVAKKF------SASSSNPFDDYYGE--
-DILILDDLRQESLKVS--DWLKLFDPLN--TARMSARYRNKMI-VPRLVIVANYQSVEQFFGSFNKN------EDINQF
KRRINFT----
>pCRESS6_KXT86702
VQQLEEEHWTPENCRPLLEMIIEKLEEK--VSTIETKVEHVHVLLKFEKGASLNK----LALAIQVEP----QYLEKLKS
GGYDN---------CLAYLCHCKD--METWTK----GRATKEALSVDWLIEKILSGEVTKSNILLTNEYAIYGQHKRKIN
EALDTAGERKSYQTIADLEAGN---------FKKTILFITAESGVGKTRYSKKLITLC------VTASTNAFDEYNGQ--
-EILFLDDIKGDSLTVS--DWLKLLDPHM--ISPISARYHNKMG-SAKIIIITNTKEPISFFEQAK----GNMGEDLGQF
VRRIDYL----
>pCRESS6_WP_000044268
TQQFDIDYWETD-IKKILAEIIERVNFT--E-LTKPVEPHIHALLKFSKGATLPE----LAAHIGIEP----QYLEKAKS
GGYDN---------LLAYIIHAKD--QKSWLQ----GRAKQKQEDIDLLLDDILNERITKQELLLNPSHLLYVVHKTRIN
EAFRAIGEIKGTRTKQDLENGL---------FKKTILFIYGKSGLGKTRLAKELVSLV------LTAGTNIFDEVNGE--
-EILLLDDVRGDSLTAS--DWLKILDPYS--ISPISARYQNRIG-SAKVIIITSTKHPLEFFYHTK----GNDREDLSQY
IRRFDFL----
>pCRESS6_WP_000201649
TQYFDPKYWELN-VEKILEEIVRRVGTS--I-GTKLKKPHIHALLKFEKGATLTD----FAVQIGLKP----EYLEKAKS
GGYDN---------LLAYLIHAKD--HLSWLK----GKAKKQYKDIDLLIDNILNGNITKKEMLLNKDHMLYAVHKSKVN
EVFRTIGEIKGTMTQHELENKK---------FKKTIFFIFGLSGLGKTKFARTLTKSV------LTAGTNMFDEVNGE--
-EILLLDDVRGDSLTAS--DWLKLLDPYN--ISPISARYQNRLG-ASKVIIITSSKHPLTFFYHAK----GNTNEDLSQY
IRRIAHL----
>pCRESS6_WP_000746010
EQQLKPKFWKQENKDKIFRLIFDRVRT---ISYAKLVEPHVHGYIDFPKKFDLSK----VASVLGVER----ERIEPKSK
GTRIN---------ALAYLIHAKD--KEDLEK----YAAKEKDESLDLVLSKVYKGELTYFDIMKDNNYYLMANNRQKFL
EGFDIFGERESVLRLEALQNGE---------YDLTVLYIQGKPGIGKSTLARDIALEY------SASSKNPFDNYSGE--
-EILILDDLREDSLAPA--DWLKLFDPIN--SARMSARYRNKLV-VPRLVIMSAYMSPKQFFGQIQE-------EDINQY
LRRVNYS----
>pCRESS6_WP_000791389
AQQFDSQFWDLS-IKDILTEMVNRANTL--E-LQELKSEHGHFLLKFSEGATLID----LALAIGVEP----QYIEKAKS
GGYDN---------LLAYLIHSKD--FSSWSK----GRTKRNSEQIDSLIANILNGKITKSEILLSSNLQLYALYKTKIN
EAFMTLGEVKATRTKKALENGD---------FKKSIFFITGKSGLGKSVLSRELVGDV------ATAGTNIFDEVNGE--
-EILLLDDVRGDSLTAS--DWLKLLDPYN--ISPISARYHNKMG-ATRVIVITSTKHPLEFFFKTK----GNEIEDLSQF
IRRFDSL----
>pCRESS6_WP_001034312
VQRLEPEYWHLNELTPLLEEVVNRVSIN--E-VTKPEKPHAHILLKFSKGETLIN----LSLQLGIEP----QYIEKAKA
GAFDN---------FLAYLIHAKD--YKSWKK----GRAKSKNTSLDEIYLQILNQQISKQEILSDPEQILYALNKTKIN
EAFMTLGEIKSNATKQALENGE---------FKKTIIFITGKSGLGKSRFAKTFVKEV------VTAGTNIFDEVNGE--
-EILLLDDVRGDSFTAS--DWLKLLDPYN--ISPISARYHNRMG-SARVIIITSTKHPLEFFIHTK----GNEKEDLSQF
IRRTTSL----
>pCRESS6_WP_003024533
VQQLEEEYWTSGNSLPLLQEVVKRLNQT--ISITELKKKHVHFLLKFEKGASLQK----IALSIGVEP----QYLEKLKS

GGFEN---------CLAYLVHAKD--MQVWLK----GRATRENLSVDYLISEILKGNITKNNILLTNEYKVYSLHKRKFL
EAFETFGERKGYQAIADLEAGK---------FKKSVFFIHAESGKGKTRLAKHLIQLC------LTASTNAFDEYNGQ--
-DILFLDDIRGDSLTLS--DWLKLLDPYT--ISPISARYHNKMG-SAKVIIITSTRTPIEFFQLTK----GSINEDSGQF
IRRIDYL----
>pCRESS6_WP_003032217
VQYFSEKYWSFEDIAPLLNEMVKRISEE--E-LTKPIDSHGHLLFKLSEGMPIIK----LSDIIGIEP----QYIEKAKR
GGYDN---------LLSYLIHSKE--FSSWKK----GRVKKLEDEIDNLILDIIQEKIGKSEILLDEKHMIYTTFKTRIN
EAFAVLGEIKGNRTKKAIENGE---------FKKTVLFIHGRSGLGKTRFAKELASKA------TTAGTNIMDDLNGE--
-EILLLDDVRGESLTAS--DWLKLLDNYN--ISFSSARYKNILP-SARIIIVTSTKHPLEFFYKCK----DNEREDLSQF
IRRFDSL----
>pCRESS6_WP_003035134
VQQLEDTYWTPDNCKPLLQVVVDKLNNE--ITVTEKKAEHIHFLFKFEKGASLSK----IALAIGIEP----QYLEKLKS
GGYDN---------CLAYLVHAKD--METWMK----GRATKEALSVDWLIEKILSGEITKSNIMLTDEYAIYGQHKRKIN
EALDTSSERKSYKTISELENGE---------FKKTVIFITADSGIGKTTISKQLIRIC------VTASTNAFDEYNGQ--
-DILFLDDIRGDSLTVS--DWLKLLDPYM--ISPISARYHNKMG-SAKVIIITSTKKPVSFFEASK----GNIGEDLGQF
IRRIDYL----
>pCRESS6_WP_003048523
EQQLKTSFWKQENKDKIFRLIFDRVRT---VSYTKLVEPHVHGYIDFPKQIDLSK----VASALGLER----ERIEPKSK
GTRIN---------ALAYLIHAKD--IEDFEK----YAATEKDESLDLVLSKVQKGELNYLEVMEDDEAFLFANNQQKFR
ESFNFFGERETVLRLKDLKKGN---------YQLTVLYIQGEPGIGKTHLANELILEY------PASSKNPFDNYYGE--
-EILFLDDLREDSLSAS--DWLKLFDPLN--SARMSARYQNKLV-VPRLIVMTAYMSPKQFFGNIKT-------EDLNQY
LRRVNFS----
>pCRESS6_WP_003104234
EQQLHAEYWKAENKTKIFQEIHRRIK----IKYIKPVEPHIHAYIEFASRRDLSV----LASTLGLLP----QYIEPSGQ
GGKVN---------SKAYLIHAKS--RADFSK----RYAVEKDESLDKVFQEIINGNLTEDDIFADEETFLWSYNQTRLD
EAFRAYGKIASKRTLRELENGE---------FKPTIIYVHGSSGIGKTTLALEVIEEY------SAGAKNIFDEYFGE--
-EVILLDDPRSDSLLPA--DWLKLLDPLN--KSYLSARYKNKLV-IGRLIVITNYQSLKSFFGKIQN-------EDLNQY
IRRFNNV----
>pCRESS6_WP_004183001
AQQLEPKYWNLNELTPLLEEVVNRVSVN--E-ITEPENVHVHILLKFSKGATLIS----LALQLGIES----QYIEKAKS
GAFDN---------FLAYLIHAKD--YKSWKK----GRAKSKNTSLDDIYLQILNQQISKQEILSDPKQIIYALNKTKIN
EAFMTLGEIKSNATKLALENGE---------FKKSIIFITGKSGLGKSRFAKTFVKEV------VTAGTNIFDEINGE--
-EILLLDDVRGDSLTAS--DWLKLLDPYN--ISPISARYHNRMG-SARVIIITSTKHPLEFFFHTK----GNEKEDLSQY
IRRIDSL----
>pCRESS6_WP_014571792
TQFLKKEYWAVIFQKQIFMALIPQL--------VTPKPPHIHAIVEFEKKKDINV----VALALGLEP----QYLDTAKR
GAEEN---------LLAYLIHAKD--RKRWKN----QAIVKKDEQLPILLEAIREGALNREQVLENPDLFLYQRHIMEFQ
AAFEGYLLKAEAETKKAIKSGE---------LKLTTLYIYGLSNAGKSRFAETLGETY------TTASTNPFDEYTGE--
-QIVILDDLRAKSMTAE--NWLKILDPER--ISKSAARYHNKII-SSHLIIITAPISPQSFFHQIS------ESEELNQF
MRRLSLT----
>pCRESS6_WP_014623544
VQQLEDEFWEPDDCRPLLELIIKNLESK--VSVIENKTEHIHALLKFEKGASLTK----IALAIGVEP----QFLEKLKS
GGYDN---------CLAYLVHAKD--METWVK----GRATKENLSVDWLIEKILSGEITKSNIMLTDDYAIYGQHKRKIN
EALDTAGERKSYRTVAELESGK---------FKKTVLFITAESGVGKTHYSKQLIALC------VTASTNAFDEYNGQ--
-EILFLDDVKGNSLTVS--DWLKLLDPYM--ISPISARYHNKMG-SAKVIIITNTKEPIRFFEQAK----GNIEEDLGQF
IRRTDYL----
>pCRESS6_WP_015647385
VQQLENEYWKPSNCRPLLEMIVEKLEKK--VSIFENKEDHVHALFKFEKGASLNK----LALAIQVEP----QYLEKLKS
GGYDN---------CLAYLCHSKQ--IETWIK----GRATKENLSVDWLIEKILAGDLTKSNIMLTDEYSIYGQHKRKIN

EALETAGERRSYRAIAELEAGK---------FKKTVLFINAESGVGKTQFSKNFIRLC------VTASTNPFDEYNGQ--
-EILFLDDIKGDSLTVS--DWLKLLDPYM--ISPISARYHNKMG-SAKLIIITNTKEPLSFFEQAK----GNIGEDLGQF
VRRIDCL----
>pCRESS6_WP_017371219
MQYLEKEYWETRNQKEIFALVCEKFRQI--NENGKLEAPHLHWAIHLKERTTLNK----IAKAFQVEP----QYIETGNQ
GAMIG---------RLAYLTHQTE--KMKFKK----LLATRAKYELNHLLQQVQVGKLFIEDILSDEKYFVYANNLAKFK
EAFEAYAQRNSLLTIQDRVAQK---------FEFTSIYIYGKSGSGKSEIAYDILKQY------FGGSKNAVDDYKGE--
-ELLLFDDVRPETFSPA--DWTKILDYKN--KSALSGRFHNRPL-SNRLVLMTNTQSPFEFFK--EN-------EPIEQY
LRRLTYV----
>pCRESS6_WP_017649267
VQQLEEKYWTADNCQSLLEAVVKHLEVT--VTVDETKEDHIHFLFKFAKGASLEK----LALSIGVEP----QYLEKLKS
GGYDN---------CQAYLVHAKD--MESWIR----GRAKQDNLSIDWIISEILAGKLSKNQILLTDDYKVYGQHKRKIN
EAIDTAGERKSYKTISELEAGQ---------FKKTIIFINAESGVGKTAISKKLIGIC------VTASTNAFDEYNGQ--
-DILFLDDIKGDSLTVS--DWLKLLDPYM--ISPISARYHNKMG-SAKVIIITNTKEPMHFFEQAK----GNIGEDLGQF
VRRIDYL----
>pCRESS6_WP_018030886
EQQLKSDYWKKKNRVAIFKEIHRRIQ----KKYLTLVEPHIHGYIEFASRRDLNH----LASVLGLLP----QYIEPSGR
GGKVN---------SKAYLIHAKN--QLDFIK----RSATEKDEELDSVFQAIVNGTLTEDDIFANEETFLWSYNQTKLD
EAFRAYGKIASKRTLRQLESGE---------FQPAVIYIHGSSGIGKTSLALELIEQY------SAGTKNIFDEYFGE--
-EIILLDDPRYDSLLPA--DWLKLLDPLN--KSHLSARYKNKLV-IGRIIIITNYKSLKSFFGKIQH-------EDLNQY
IRRFNNV----
>pCRESS6_WP_018376545
SQQLRTELWKAEAPDELLKEMIEHLSTK--Y-VQVLKSFHVHFLFKFSEGATLTE----IANALNLEI----QYLEKPKS
GAYDN---------LLSYLVHAKD--IHVWEK----GRAKQQKFDVEALIMDIIVGKIKKTEVLSSPDYPIYAFNKSKLN
EAFETYAERKSLKTLKDLELGL---------FQKTIIFFQGASGLGKTTLAKELTHEI------ITAATNPFDDVKGE--
-EIILLDDVRGQALSAS--DWLKLLDPFT--ASPISSRFKNRSG-AVKTIIITSRKPPLEFFFKTK----DSGYEDLSQF
IRRIHHF----
>pCRESS6_WP_018380019
EQQLQSDYWKDENKRAIFQEIYDRVK----VSFSRLVKPHVHGYIEFKTKRDLNI----LALSLGLLP----QYIEPSGK
GGKIN---------SKAYLIHAKS--KEDFAK----QSATEKDESLDLVISKVQKGELSYQEVMEDDEAFLFANNQQKFR
ESFNFFGEREAFLRLKSLERGD---------YQLTVLYIQGEPDVGKSTLAKEIALKY------SASSSNPFDNYYGE--
-EILFLDDLREYNLSAS--DWLKLFDPLN--SARMSARYQNKLV-IPRLVIMPVYKTPKTFFGEVQA-------EDLNQF
LRRINFL----
>pCRESS6_WP_019299400
EQYFDQEFWKEKFKENIFQEIYDRLKF---KKWDKLIEPHIHVYVELPTKRSIER----IADRIGISQ----HFIEPKGK
NFPFN---------EKAYLIHAQQ--EKEFLK----RSATKTDESLDLIFDKVLFGKITYDEIMEDETYRLYANNEQKFI
SAFNSFAQYKARKTLKALKNKE---------FKMSVIYIQGKSGIGKSHLAGEIVEKY------SASASNPFDEYKGE--
-EILLLDDLRPDSLERA--DWLKVLDPMN--KSRISARYRNKAI-ASRVIVLTNTETAEQFFKNIKN-------EDLDQY
IRRINLT----
>pCRESS6_WP_020997784
VQQLEEEYWSVDNCRPLLEIIIQKLEEK--VTIINDKEEHVHALLKFEKGASLKK----IALAIGVEP----QYLERLKS
GGYDN---------CLAYMVHAKDF-IETWVR----GRATKEDLSVDWLIEKILAGEISKSNILLTDEYAIYGQHKRRIN
EALDTVGERKSYQAIIDLETGN---------YKKSALFIMADSGVGKTKFSMELIHHC------LTASRNAFDEYQGQ--
-DILFLDDIRGDSLSVS--DWLKLTDPFM--ISPISARYHNKMG-SAKLIIITSTLLPSVFFSQAV----GNKNEDNGQF
IRRFDYQ----
>pCRESS6_WP_020999261
AQQLRTELWEIDTPEELLREMVKHLSKK--E-VQVLKASHVHFLFKFSEGGTLSD----IANALGLET----QYLEKPKS
GAYDN---------LLAYLVHAKD--IRAWEK----GRAKKLKFDVEALIIEIIEGKIKKERILSSPDYPIYAFNKTKLN
EAFETYAEQKSMKTLKNLELGL---------FQKTIIFIQGSAGLGKTTLAKKLAREI------ITAATNPFDDVKGE--

-EIILLDDIRGQALSSS--DWLKLLDSFT--ASPISSRFRNRSG-AVKTIIITSSKHPLEFFFKTK----DSGYEDLSQF
VRRINYL----
>pCRESS6_WP_022765681
AQELNPELWFD--LQRVTAIIAKRLEDT--QQKVTLKHLHAHWVIEFKKGAKLTD----IALAIGLAS----QYVEKPKK
GAVDN---------MLAYLIHIKY--ESVWKK----GIATLAAEDIDWLEEQILEGKIKKTQIFLTDSFKTYSHYRQRCE
DAFACYTDKKIYTAIEAMKNKE---------FLLTTYFITGASRKGKSRFAEDLAQRC------RTPTSNPLDDYAGE--
-EIIIMDDSRGCTLTAE--AWLTFLDPNF--CNPAGARYHNKPG-IPKVLIITSTKSMLEFFFYTG----GSRSEAMDQF
FARVFSR----
>pCRESS6_WP_024385235
TQQLESEYWDIDDLSLLLEEVVNRVSVN--G-ITVPERNHAHILLKFSKGATLIS----LALQLGIEP----RYIEKAKS
GAFDN---------FLAYLIHAKD--YKSWKE----GRAKSKYTSLDEIYLQILNQQITKQEILSDPKQILYALNKTKIN
EAFMTLGEIKSNATKLALENGE---------FQKTIIFITGKSGLGKSRFAKTFVKEV------VTAGTNIFDEVNGE--
-EILLLDDVRGDSLTAS--DWLKLLDPYN--ISPISARYHNRMG-SARVIILTSTKHPLEFFFDTK----GNEKEDLSQF
IRRTTSL----
>pCRESS6_WP_024400359
VQQLEPEYWDIEELTLLLEEVVNRVSVN--E-ITELASPHVHILLKFTKGSTLTN----FALQLGIEP----QYIEKAKV
GGYDN---------LLAYITHQKD--YKVWQQ----GRVKSKNASLDEIYLQILNQQISKQEILSDPKQILYALNKTKIN
EAFMTLGEIKSNATKQALENGE---------FKKTIIFITGKSGLGKSRFAKTFIKEV------VTAGTNIFDEVNGE--
-EILLLDDVRGDSLTAS--DWLKLLDPYN--ISPISARYHNRMG-SARVIIITSTKHPLEFFFHTK----GNEKEDLSQF
IRRTDTL----
>pCRESS6_WP_025016923
TQYLNPKYW--INKQAIFEEIFARANL---ENKG-LKDAHIHGYLEFSKQKTIAS----ISSVLAIEP----QYVEAPKK
GGRLN---------CLAYLIHAKN--LEDFKK----YNATKKEIGLDLALQEVQQGKLKLREIMRDENALLYANHMNQFN
DSFNFYGLRNAMLRLDELEQGK---------YDLTVLYIQGAPGIGKSFLAREVAQKF------SASSSNPFDDYYGE--
-DILILDDLRQESLKVS--DWLKLFDPLN--TARMSARYRNKMI-VPRLVILANYQSIEQFFGSFNKN------EDINQF
IRRISFS----
>pCRESS6_WP_027972054
VQQLEEEYWEPENCRPLLELIIKKLRET--VTIVKNKEEHVHALLKFEKGASLNK----IAFAVGIEP----QYLERLKS
GGYPN---------CLAYLVHAKD--METWIK----GRATKEDLSIDWLIDKILAGEVTKSNIMLTDSYAIYGQHKRKIN
EALDTAGERRSYQTIAEMEAGK---------FKKTIIFIQAESGAGKTRLSKKFIALC------VTASTNALDEVNGQ--
-EILLLDDLRGSSLTVS--DWLKLLDPYM--ISPISARYHNKIG-SSKVIIITSTKKPIDFFEVAK----DNVGEDLGQF
IRRIDYL----
>pCRESS6_WP_032497992
VQQLEKEYWHSSNCRPLLDLIIEKLEKK--IVTSTNKAEHVHALLKFEKGASLNK----LALAIQVEP----QYLEKLKS
GGYDN---------CLAYLCHIKS--MEIWVR----GRATKELLSVDWLVEKILSGKLTKNNILLTDEFSIYGQHKRRIN
EALETIGERKSIQTIAELEAGK---------FKKTIIFITADSGMGKTQYSKKLITIC------VTASTNAFDEYNGQ--
-EILFLDDIRGESLTVS--DWLKLLDPYM--VSPISARYHNKMG-AAKVIIITTTNDPLSFFRKAK----GSFGEDLGQF
VRRIDYL----
>pCRESS6_WP_032941943
EQQLKAEYWLKENKLLIFEEIYRRLKT---KNDNEFIFPHVHLYGKYTDKRTLAR----IAKVLGIKE----QYIEPKNG
NFEEN---------QLAYLTHAQQ--TKKFEK----QSATKRDESLDLINQQILKGELFLEDILADDDFLLYSNHKLQFK
QAFDSYAERLAFKNLKDLTTGK---------YKLTVMYFQGKSSLGKSYLARTIAQKY------SASSSNPFDDYYGE--
-DIILLDDIRPDSLRKA--GWLKLLDPIN--TSRMSARFTNKQV-VPRLILITNTQLPEQFFNIFKD-------EALDQY
IRRINFC----
>pCRESS6_WP_034704841
VQQLKEEFWTINNIQPLMEELNKRFHNE--RLVIEPKEKHAHILVKFAKGDTLNN----LSVTAGVDP----QYIEKAKS
GGYDN---------LLSYLVHAKD--METWVR----GRATKADLSVDYLVSEILDGKLTKSQVLLTNEYKVYALHKRKIN
DAFDTAGENKSYQTIADLDAGN---------FKKTILFIMAESGAGKTVLSKKIISIC------LTASNNAFDEYNGQ--
-DVLFLDDIRGDSLSVS--DWLKLLDPYT--ISPISARYHNKMG-SAKVIIITSTKTPSEFFSIAK----SNFHEDLGQF

FRRIDLL----
>pCRESS6_WP_036321578
VQQIKPECWGLRAAASFSQYLVVLLEST--QLGEVLKYLHLHLLLKFKTSAAVEK----LAAILGVEV----QYVEDKSR
GQHDN---------GLAYLTHVKY--YPAWRK----GRAHKRKLEFEPFREMVLQGELTRDQIMLTDEFDIYSRHQREID
DALSAYGQRRAYRAAAKLRAGA---------FSTQVVFIHGEAGVGKTRFANAFIQEY------RAATTNPLDDWRGE--
-EIMLLDDLRASAMDAN--DWLLLLDPHN--ASPARARYKNKGE-VARLIVITATIEPVEFFFFAK----GNVDEALDQF
IRRLQSV----
>pCRESS6_WP_039670385
EQQLKGEYWKAKNKQEIFQEIYNRVK----KTFNNLVFPHLHGYIEFSNKRDLSV----LALNLGLYP----QYIEPSGR
GGKIN---------SKAYLIHAKS--LDSFTK----QSAKEQDESLDMIFQEIIKGKLTEDDIFADEKTFLWAYNQQKFD
EAFKAYGKISAKTTLRQLENGE---------FKPTILYIHGQSGIGKTSLAYDLVAEY------NAGSKNIFDEYFGE--
-EIIVLDDPRYDSLLPS--DWLKLLDPLN--KSYLSARYRNKLV-IGRVIVITNYMSLSEFFRQIPK-------EDINQY
LRRFNNV----
>pCRESS6_WP_039677656
VQQLENKYWTADNCRPLLEIIIKKLEDE--ISIIKNKAEHVHILLKFEKGASLNK----IALAIEVEP----QYLEKLKS
GGYDN---------CLAYLVHAKD--MKTWVK----GRATKENLSIDWLIEKILAGEVTKSNIMLTDDYTIYGQHKRKIN
EALDTAGERKSFQAIEDIDSGK---------FKKTIIFLQGESGQGKTKLSKSIINIC------STASTNAFDEYNGQ--
-DVLFLDDMRGDSLTVS--DWLKLLDPYT--ISPISARYHNKMR-AAKLIIITSTKAPLEFFSLAK----GNFGEDLGQF
VRRIDLL----
>pCRESS6_WP_039694464
EQQLKSEFWKQENKNEIFRTIFDRVRS---ISYTKLVEPHVHAYIDFPKQMDLSK----VASALGVER----ERIEPKSR
GTRIN---------ALAYLIHAKD--REDFEK----YSATEKDESLDLILSKVQTGELNYIDVMENDNAFLFANNQQRFR
ESFNFFGERETVLRLKDLKQGN---------YQLTVLYIQGKPGIGKTHLANEIILSY------SASAKNSFDNYYGE--
-EILLLDDLREDSLSPS--DWLKLFDPLN--SARMSARYQNKLV-VPRLIVMTAYMSPKQFFGQIET-------EDLNQY
LRRVHFS----
>pCRESS6_WP_041290927
TQYLDPSYW--QGAGPILAYVVQRLEAE--REVVEPKPDHLHAVIKFAKSAPLDR----LAFGIGVEP----QYVEKPGR
GAFDN---------MLSYLTHVKY--RETWLK----GRAHKVAENFEDMRERVLQGEITRDQIMLTDEFDIYSRHQREID
DALSAYGQRRAYRAAAKLRAGA---------FSTHVVFVHGDAGIGKTRFATDFITSY------RAATGNPLDDWRGE--
-EVLLLDDLRASAMDAN--DWLLLLDPYN--ASPAKARYKNKGE-VARLIVITATIEPVEFFFYAK----GNVDEALDQF
IRRLASV----
>pCRESS6_WP_044671103
EQQLKSKFWKQENKGKIFRLIFDRVRT---ISYTKLVEPHIHGYIDYPKRIDLSK----VASALGVER----ERIEPKSK
GTRIN---------ALAYLIHAKD--KEDFEN----FSATEKEERLDLVLSKVQSGELTYLDVMKDDKAFLFANNQQKFR
ESFNFYGEREAFLRLQALQRGD---------YQLTVLYIQGKPGIGKSTLARDLALEY------SAGSKNPFDNYYGE--
-EILLLDDLRKDSISGT--DWLKLFDPIN--SARMSARYQNKLV-VPRLVILSAYMSPKTFFGQIET-------EDLNQY
LRRINFS----
>pCRESS6_WP_044762265
TQQLEFEFWELDNAKELLHKMVEYLSWS--E-VKVLKTIHVHFLFKLAEGATLPE----IANALGLET----QYLEKPKS
GAYDN---------LLSYLIHAKE--IRTWEK----GRAKKQKNDVEVLIMDIIKGKIKKDEILNNPEYAIYAFNKSKLN
EAFETYAERKSLKTYKDLELGL---------FQKTIIFLQGASGLGKTTLAKELAHKV------ITAATNPFDDVRGE--
-EIILLDDVRGQALSAS--DWLKLLDPFT--ASPISSRFKNRSG-AVKTIIITSSKSALEFFFKTK----DSGYEDLSQF
VRRINHF----
>pCRESS6_WP_044774450
VQQLEEEYWEPENCRPLLELIIKKLRET--VTIIKNKEEHVHALLKFEKGASLKK----IALAISVEP----QYLEKLKS
GGYDN---------CLAYLVHAKD--METWTK----GRAIKEDLSVDWLIEKILAGEISKSNILLTDEYAIYGQHKRRIN
EALDTAGERKSYEAIADLEAGK---------YKKSAIYVLADSGVGKTKFCMELIHRC------LTASRNAFDAYQGE--
-EILFLDDIRGDALSVS--DWLKLNDPFM--ISPISARYHNKMG-SAKLIIITSTLLPSVFFSQAE----GNKNEDNGQF
IRRFDYQ----

>pCRESS6_WP_045759092
VQQLEEKYWGPNDCRPLLEMIIKKLKSK--ISTVEDKAVHVHALLKFEKGASLNK----IALAVEVEP----QYLEKLKS
GGYDN---------CLAYLVHAKD--IETWLK----GRATKENLSVDWLVERILDGEVTKRNIMLTDEYAIYGQHKRKVN
EALETAGERRSYRTIAELEAGK---------FKKTVIFIQASSGVGKTKLSKDLIELC------MTASTNAFDEYSGQ--
-EILFLDDIKGNSFTVS--DWLKLLDPYT--ISPISARYHNKMG-SARVIIITSTKLPVELFYFAN----GNHNEDLGQF
VRRIDLL----
>pCRESS6_WP_047206721
TQYFETKYWELN-IEKILEEIVRRVSTS--I-GTNLKKPHIHALLKFEKGATLTE----LAVKIGLEP----QYLEKAKS
GGYDN---------LLAYLIHAKD--HLSWLK----GKAKKQYKDIDLLIDNILNGTITKKEMLLNKDHMLYAVHKSKVN
EAFRTIGEIKGTMTQNELENKK---------FKKTILFIHGVSGTGKTTLANQIVQNV------LTAGTNMFDEVNGE--
-EILLLDDVRGDSLTAS--DWLKLLDPYN--ISPISARYQNKIG-AAKVIIITSSKHPLTFFYHTK----GNNREDLSQY
IRRIAHL----
>pCRESS6_WP_049476139
VQQLEDTYWTFDDCKPLLQAVVDKLNDE--ITITEKKAEHVHFLFKFDKGASISK----IALAVGVEP----QYLEKLKS
GGYDN---------CLAYLVHAKD--METWMK----GRATRESLSLDWLIEKILSGEITKSNIMLTDEYAIYGQHKRKIN
EAIETSGERKSYKTISELENSE---------FKKTVIFITAESGIGKTALSKQLIRIC------VTASTNAFDEYNAQ--
-DILFLDDIRGDSLTVS--DWLKLLDPYM--ISPISARYHNKMG-AAKVIIITSTKKPISFFESAK----GNIGEDLGQF
IRRIDYL----
>pCRESS6_WP_049499636
VQQLEEKYWMPNDCLPLLKMIVNNLESK--ISTVQDKAVHIHALLKFERGASLSK----IALAIRVEP----QYLEKMKS
GGYDN---------CLAYLVHAKD--IEMWVK----GRATKENFSIDWLIEQVLDGKLTKSNIMLTDEYEIYGQHKRKVN
EALDTAGERRSYRTIAELEAAL---------FKKTVIFIQANSGVGKTKFSKELISAC------VTASTNAFDEYNGQ--
-EILFLDDIKGDSFTVS--DWLKLLDPYT--ISPISARYHNKMG-SARVIIITNTKLPVELFYFAK----NNYNEDLGQF
IRRIDLL----
>pCRESS6_WP_051176704
TQQLRPELWELRRANRLLSLLVLLLEQE--QNTMEPKFPHLHATVKFP-TFKIAR----IAKILGVET----QFVEKPN-
GSHDA---------QLAYLIHAKD--EAEWFL----GRGKKLALSVDALLEQVLSGNITKENVLLSDEFRVYSELSDKFN
RAFVVAGERKAYRAAEAMKNGD---------FHTAIFFLYGAAGSGKTRLANLLVDQY------RAASRHSLDDWQGQ--
-EIILMDDVRASAMSAS--DWLTLLDPYN--PNPASARYQNKLA-VARLVIITASIDPVTFFYYAK----GDVDEALDQF
IRRLMAS----
>pCRESS6_WP_052506726
TQAWLSNHWDDNNLSLVTQKFVEYLSET--REVLEPKHLHGHWVVKFAKGLTLSD----IAVAVGLAP----QFIEKAKP
GGYDN---------MLSYLIHAKD--KSAWEK----GRAKKRDELVEELYEKVINGEVTHDQILLTDEYAVYSRNIDRFE
KGFKAYTDRKILQAVRDLEAGK---------FNLSVLYFQGQAGHGKTATAVQLAQSC------QAAATNPVDDYNGE--
-EILIMDDLRGNAMRAT--DWLKLLDPYN--SSPNSARYKNKRV-VSRYIFITSIQDVYEFFYYTG----ADRSEPLDQF
MRRILAL----
>pCRESS6_WP_053092713
VQQLEDEHWTAKNIRPLLETVTDKLNRE--VNVIEKKAEHIHFLFKFEKGASLNR----IALAIGVEP----QYLEKLKS
GGYDN---------CLAYLVHAKD--MATWVK----GRATKENLSVDWLVEKILAGDITKENIMLTNEYAVYGRHKQKVN
EAIETAGEQKSYQTIAELKEGK---------FKKTILFISAPSSAGKTRFAKELIDIS------LTASTNAFDDYNGQ--
-EILFLDDMRGSSMTAS--DWLKLLDPYM--ISPISARYHNKIG-SAKVIIITSTKKPIPFFEIAK----ENDNEDSGQF
VRRIDYL----
>pCRESS6_WP_054952722
TQYLDPSYW--QGAEAILAYVVQRLEAE--REVVEPKPEHLHAVIKFAKSAPLDR----LAFGIGVEP----QYVEKPGR
GAFDN---------MLSYLTHVKY--RETWQK----GRAHKIAENFEDMRERVLQGEITRDQIMLTDEFDIYSRHQREID
DALSAYGQRRAYRAAAKLRAGE---------FSTHVVFVHGDAGIGKTRFATDFITEY------RAATGNPLDDWRGE--
-EVLLLDDLRASAMDAN--DWLLLLDPYN--ASPAKARYKNKGE-VARLIVITATIEPVEFFYYAM----GNVDEALDQF
IRRLASV----
>pCRESS6_WP_056938517

AQQLQPEFWQPNDARDLLDNVVQRLDKT--ETIIRQKEDHVHFLLKFDKGNTINN----LAMTIGVEP----QYLEKAKS
GGYDN---------LLAYLVHAKD--RETWLR----GRATQNMQSVDYLIAQVLQGKLTKSQIMSDEDYMVYGLNSSKIN
GAFTVIGERKSITAQRDIEASK---------FKKKIIFISGTAGVGKTKFGKLLVRQC------VTASTNPFDEYSGQ--
-EILFLDNVRGETLGFL--DWLKLLDPHN--ISPISARYHNKFG-VAKVIIITSPVPPYQFFNHPK----FNSMEDLGQF
YRRIDFW----
>pCRESS6_WP_058223604
EQQLKAEYWLEQNKALIFEEIYRRLKT---KNDYELVFPHVHLYGKYSDKRTITR----IAKVLGIKV----QYIEPKDK
NFEEN---------QLAYLTHAQQ--AKKFEK----QSATKRDESLDLINQQILKGELFLENILADDDFLLYSNHKLQFK
QAFDSYAERLAFKNLKDLTTGK---------YKLTVMYFQGKSSLGKSYLARTIAQKY------SASSSNPFDDYYGE--
-DIILLDDIRPDSLRKA--DWLKLLDPIN--TSRMSARFTNKQV-VPRLILITNTQLPEQFFNIFKD-------EALDQY
IRRINFC----
>pCRESS6_WP_061343647
TQQLEQEFWDNRNQRRIFEIIATRFLRK--DDTGNFIKPHVHWLLELKNKRDLDE----IAYKFFVHP----QQIEKGSK
GCFLG---------RIGYLTHQAE--SAYFKK----RLAFQQKMDVDYYLQQVQQGILFLDDIFLDLNYNVYANNKQKFR
EAFDAYSELNSFRTNRDKRLGI---------FDFTTIFIYGRSGLGKTTIAMAILDRY------SGSAKNAVDDYKAE--
-ELILFDDLKQDSFLIA--DWLKILDSRN--ESTISGRFHNKPL-SARLIILTTIESPFKYFDFGKD-------EPKEQF
IRRLSYI----
>pCRESS6_WP_067483596
VQQLEPEFWVINNIRPLIEELVKRFEKL--ISVEELKAKHVHILIKFGKGDTLNS----LAVKAGVAP----QYLEKAKS
GGYDN---------LLSYLVHAKD--METWVR----GRATKADLSVDYLVSEILAGKLTKSQVLLTNEYRVYALHKRKIN
DAFDTAGESKSYQTIADLDAGN---------FKKTTLFIMAESGAGKTVLSKKIISIC------LTASNNAFDEYNGQ--
-DIIFLDDIRGDSLSVS--DWLKLLDPYT--ISPISARYHNKMG-SAKAIIITSTKTPSEFFSIAK----GNFHEDLGQF
FRRIDLL----
>pCRESS7_ABC65794
VINAN--KITKSKIENILELKK--K----TYQNGDLKSPHYHIYLRFNYAYDTKH----IAQWFNTQD----NFVSKIKG
R----------FSDALMYMTHAN--SNLNKQF----KMNYARLKEINSQIISGEIKEYNIDERINVDEYYVYSAAIEKAF
KYRVIT----LKR-------IK---------RQMECVFITGQSGSGKSTLAKKIAYIS-------SGSNDILDDYRGE--
-ECIILDDLRSNCLGLS--DLLKMLDNNT--ASSVKSRYKNKVL-ECKLIIITTVKSIDDFFEDIFR-----KDETIIQL
KRRCTYY----
>pCRESS7_ABC65805
VINSD--KINKTKIENILELKK--K----IYQNGDLKKPHWHIYLRFNYAHDTKH----ISQWFNTQD----NFVSKIKG
R----------FSDALMYMIHAN--RIFLREY----KID-SRLEEILSKIQSGEIKECNSTNHISIIENNIYSSAIEEAV
KYRNNT----LKG-------MD---------RQMECVFITGLSGCGKTTLAKKIAYIS-------SGSNDVLDDYRGE--
-ECIILDDLRSNCLGLS--DLLKMLDNNT--SSSVKSRYKNKVL-ECKLIIITTVKSIDDFFEDIFK-----KDESIIQL
KRRCKLH----
>pCRESS7_CCY61699
VQQLE--YMSQEDVEAGLD--H--N----VHEDGTPVAPHWHIMIRFKRPVQTES----LCKWFGIKS----NMIGYILG
T----------FGDAVAYLTHRN--KLSKKKA----SQR---KEEIIELIRSGIVREYNYTEYITALEYDKFKRAIDNAF
TYRRDT----LKS-------LD---------RHMNVIYIYGGSGTGKTTYAKQLAYIS-------SGSNDPLDGYKGQ--
-DCLILDDIRPGDFLLS--DFLKILDNNT--QSTVKSRYKNKLL-ECQYLIVTTSFDIPVFFDLLLDS----EGESVKQM
ERRCTLK----
>pCRESS7_CCZ68460
VSNLESELFDIERMKQVLEEKSK-T----VYTEGELKPKHIHLLLRFNQPQKLKN----IAGWFQIPP----NFVSKIHN
R----------WDSAVLYQIHAN---CMKRN----------SIDSILMDILNGEIPEYQR-SVIPPLFRVHYAREINEAF
RCRVQN----LQE-------TS---------RKMECIYITGSSQAGKTTLAKKIAYIS-------SSGTDFLGEYALE--
-PCVILDDIRPSSINLS--ELLKLLDNNT--VSAVKSRYKNKCLANCKLLIITTVLDIETFYHNVFSE----EDEPMIQF
KRRCGTH----
>pCRESS7_CCZ93342
IQQLE--YMSAEDVGSGLD--H--N----VNDDGSPKAAHWHIYIRFKDSTPTDS----ICKWFGITS----NYIGRIQG

R----------FADALAYATHRN--VRSKEAD----KQR---KAEISDLIISGVIREYNYTDYITIQEYDRFRKSIDNAF
KYRLDK----IKG-------EN---------RDMEVIYIFGDSSCGKTTYAKELAYVS-------SGGEDMLDDYKGQ--
-DCVILDDLRANDINFS--SLLKLLDNHT--QSMVRARYHNKFL-ECKLMIITTSKSMEELFRELPGS----DNEDITQL
RRRCKLY----
>pCRESS7_CDE19587
VTQPEYLKSPLQDILRKYRTIKQWAYILHDKDKDAS--SHYHIYINFQQTVDSKD----VAGWFGIPEQ----FVNKVEG
RKTDML-----------MYLTHSN---------------DSYDFSEVVANFDFKSEIEQAKIIGDFEKYSYIHSLAVSE
QPKCFDRLQKLWKLQCQWLSLN-------SDRNLKVIFVTGKSGTGKTYFARKYMRAYVS-----SSSNDPLDMYMGQ--
-KGIIFDDLRDEAFEFA--DILKLLDNNT---STAMKSRFTNKVLNCKVMIITSFIPIKYWYKSVRYS-----CDGIEQL
YRRINMY----
>pCRESS7_CUN62864
QTQIP--FLPKEKMLSIIAEHTTAG----IKEDGTPKEPHWHIELRLTRGRRLTD----IASWFGLPT----SCIQTSKS
G---------RYEPMLQYLIHEN------SKI----DGDK-RISEIVELIANGTIREFNIDEFVTVREYDKYRSHIKNAL
EYRSII----LEKQ------NT---------RNMEVIYIYGKGGTGKSSFAEAIAKR-------SASERDPLATYKGQ--
-DSFALDDVRGNTFEFQ--DWQGVLDNFQ--DRPGSSRFHDKHFTECKLLFITTTDSAEDFWKEMSAKR-P--
NEDSHQF
FRRIKTV----
>pCRESS7_KXT29039
VIKED--LIKKDFIDKVLK-KK--K----VLEDGSLKNPHYHIVLVLKTPYDVEY----IASWFKTSS----NFVEKIKG
N----------MSDILNYLTHKN--DGSIKKY----RLD-KRLKDLLTKIMNGEIKEYNISKKITVYENNIYATALERAF
KFRTNF----LKG-------VK---------RNMDCIFITGKSGSGKTTYAKYLAYVS-------SGSNDILDDYQSQ--
-ECIILDDLRPECLGLS--DLLKMLDNNT--ASTVKSRYKNKVL-ECNLMIITTTIPIKKFFDLVFYKK--EKKETIVQL
QRRCKVH----
>pCRESS7_ODR34583
VSDTT--HLSLETIQAVTGDKA--K----N-KDGTDATPHYHVFLRFEDTQDTKY----VAKWFGIAE----NFVGKING
K----------WTDALLYLTHEN--ALTSQSL----KAR---EAEIVSLIANGTIKKYNYNEYITPVEYVRLNASIKAAM
NYRADM----LSHN------HN---------RQLEVVYIVGGSGCGKSTYAKRLAYVS-------SGSNDVLDDYGGQ--
-DCLILDDLRPSCLGLS--DLLKLLDNNT--STSVKSRYKNKIL-ECSLIIITTVKEIDEFFSKVFEH----EDEPLKQL
KRRCRTM----
>pCRESS7_SCG87263
VSRLTDDLFDINTIPDIIKKHS-------VYTEGTLKPPHIHLYLHFNSPQHIPQ----IAKWFNIGK----QFLEVCHG
R----------EIDVLAYLVHLTPSSSARRNN----TLHQADIDLICQKIMSGEICEYNKVQMIGPQILFDSGYKIERAF
KIQQET----FEE-------NN---------RDTLAIYIEGPAQVGKTTFAKTIAYIS-------SSSNDLLGDYKQE--
-PVVILDDLRPDSIGIS--DLLKLLDNHT--ASTFKSRYKNKYLN-CRIIIITSTLEISRFFEEVAAG----KAEPIEQL
KRRCRIH----
>pCRESS7_WP_002578150
QNQIE--YTSESSFKNIVNNLYKRG----TYTEGNLKKTHIHGMLRLNNSYKFST----IANWFDVTA----QRIRKIET
S----------YAAACAYLIHRN--NQLTNEN----HMK-KLKKRILEEVEAGTLRGYNFHENYAFSDRVALRSYLNNAI
EIIKTK----LNSN------KE---------RDLEVIYIHGSSGAGKTTYAKMTAATS-------GEDRDPVETYDSH--
-PCMILDELRPSSMKLT--SFLKLVDNNT--ESMAGARYHGKAFIECKLIIITSILPIEEFFKNLQAN----DNETAIQI
KRRCKTM----
>pCRESS7_WP_011161011
VINSN--LINQSKIENILEAKK--N----IYQNGDLKTPHWHIYLRFNNAYDVKH----IAQWFNTEE----NFVSKIKG
R----------FSDALMYMIHAN--RIFLRKY----KID-TRLKDILTKIHSGEIKEYNITNYISIIEHNIYSAAIEKAF
KYRTNT----LKG-------IE---------RNMECVFITGMSGSGKTTLAKKIAYIS-------SGSNDVLDDYQGQ--
-ECIILDDLRSNCLGLT--DLLKMLDNNT--SYSVKSRYKNKVL-ECKLIIITTVKSIDDFFEDIFK-----KDESIIQL
KRRCKLH----
>pCRESS7_WP_019282500
VSNIE--HLDLEYIKEKLESATNGK----TYEKGDLKAPHVHLMMKFKSPQKVHC----IAKWFKVKD----NNINKIKS

K----------WVSALRYLIHAN--HNTHNNK----KKQ--RKEEIVNNISKGEWKALDLTDNITELEFVEFSADINKAL
NYRQTF----LQLNN-----KG---------RDMNVIYISGGSGSGKTTLAREYAYVS-------DGGKNPMDNYMGQ--
-DCIILDDFRPDVMGFS--DLLKLLDNHT--SSMVNARYYNKFMGECKLLIITTIDDLPDFFKKMQDT----KGEPIKQF
ERRCKTK----
>pCRESS7_WP_028509833
TQDCC--GWTPENVHKFIDGWSSVKDYAYKKDDTTPREPHIHLMLRFSCAVHTSN----ILARAKITE----NRIQKMKS
WSAALNYLTHRDEHKWKHVYHQA------KQL----RADKGREKEIVEAIASGEIRLFNLSEHITSYEENLYSKAIKTAF
NRRTRD----LKLK------NE---------RNMEVIFISGESGVGKDTFAREWCKDSYF---TTGNNDSPFDDYMGQ--
-DVIIWSDARDDVYKPA--QIHTMLDNHW--SSTQKARFVDQVLN-CQYFIITSVKPLNEWYKNFYSKE----GEDIKQL
YRRIKTWYDM-
>pCRESS7_YP_001966814
VIKAD--LIKQTEIEKVLESKK--K----KYLNGDYKIPHWHIMLRFHQSQEFKY----IAKWFNTTE----NFVSQIKG
R----------FTDALLYLTHAN--RTFMRKY----KLD-TRLVDILNKINSGEIKEYNITNHITIIENNIYSAAIEKAF
KYRNSK----LRE-------MD---------KKMECVFITGQSGSGKTTLAKQISFIS-------SGSNDVLDGYKGQ--
-ECVILDDLRADCFGVS--DLLKMLDNNT--ASSVKSRYRNKYL-ECNLIIITTTKTLNAFFDTVFNKS---DDEDVKQL
KRRCRIH----
>pCRESS7_YP_003617079
VINQK--LITKTKIETILETKK--K----IYQNGDIKAPHWHIYLRFNYAQDTKH----ISQWFNTQE----NFVSKIKG
R----------FSDALMYMIHSN--RIFNRKY----KMD-ARLKEIIDKIESGEWKRYDLITKINGYENNIYYSAIKKAF
ERRIDF----LEE-------ME---------RKMECVFITGMSSSGKTTLAKKIAYVS-------SGSNDILDNYKGQ--
-ECIILDDLRSYCLGLS--DLIKMLDNNT--ASSVKSRYKNKVL-ECKLIIITTVKSIDDFFDDIFKD----KDESIIQL
KRRCTYH----
>pCRESS7_YP_006961027
VINKT--LITKTKIETILEAKT--K----TYQNGDIKAPHWHIYLRFNYAYDTKH----ISQWFNTQE----NFVSKIKG
R----------FSDALMYMIHAN--RIFNRKY----KMD-ARLKEIIDKIESGEWKRYDLINKINGYENNIYYSAIKKAF
ERRIDF----LEE-------MK---------REMECVFITGMSGSGKTTLAKKIAYVS-------SASNDVLDNYKGQ--
-ECIILDDLRSYCLVLS--DLLKMLDNNT--ASSVKSRYKNKVL-ECKLIIITTVKSIDDFFDDIFN-----KDESITQL
KRRCKFH----
>pCRESS8_ABP89830
AYMFCSTVESVSKNLQEIIKLFQEIDT----------EPHYHIIVRFKNAVWLNS----IINKLSQNQS---NFFEAWKG
KVNNA----------YSYLIHRTEDAESKIQS----NSRKETINVVRNLINKIIAGDISFDEAIKEVDGYTLVKYDREFS
RAKKRRTEIDFENWKENALKNG---------FKREIIWLYGPSGTGKSRLCKHFAKSYT-----TGSSRDPFQNVASQ--
-ETIIIEEIRPGNFNYA--DFLLIIDPFN-ADATASSRFFDKPI-IATTIIINTPFSPFQFYESISKQ--VGKIDTVIQL
IRRITLL----
>pCRESS8_AKG47101
VIMFTQQLKHLD--SQDELIKKVNTD-------NSPITSHIHLVLCFKQRVRITS----IARNLDQK----EQYFEDIET
SRNNA----------FAYLIHNTTQAKQIIFY----SP--------KQVLADFNSGNINKLEALKRIKSPRIPQYVASIN
KIEEINIQLKQKNWIAEHEKSQ---------KPIAVVWVYGFSGTGKTEFAKHIAKKDF-----TGSTRDLFQNIGT---
ASSLIIDEIRPKDIKFN--DLLKITDPFN-YRKFAPARYKDRAI-IADTIIFTSPYSPVRFFSKYKLD--NN--DTFRQL
QRRITLT----
>pCRESS8_CDA26462
QFMYTQDLEHLPKQEQLKEILEKSVNE-----KGEPIRPHFHVILKFKDAKTISR----IAKLFN-DQQ---QYIEVWHN
TINNG----------YSYLIHETTNAREKVNK----PS-KH---DIENFIDDYSNEQLTKEGLQEKIGVLEMAKHKTLLD
HIEDILAYKKHQQFLKDFK--G---------QKCTTYWIWGSSGIGKTKLVREVLEEII-----LGSQRDHFQEYAGQ--
-EFIVINDLRPNDYDYG--QLLTLLDPWE-IDKMAPARYHDRYL-NARSIYITTPYDPLSFYFECNIS--NQVVDSFEQL
KRRIVS-----
>pCRESS8_CDI42894
NFMYTQDVDHLPKKDNLASILEKSVDE-----SKKKIRPHYHVMIRFKDAKTITK----ISKIFG-DKA---QYIEAWHN
TINNG----------YSYLLHETNGSRRKVKK----PS-RQ---AIEDYIEDYSNGILTREALQAQIGVLEMAKHKTLLD

HIDQILDQKKHNEFLEEFK--G---------QKCVTYWLWGESGVGKTRLVREALEKCI-----LGSQRDHFQVYEGQ--
-NHIVINDLRPNDYSYG--QLLMLLDPWE-NDKMAPARYRDKYL-NAKSIFITTPYDPFSFYNGCYIE--
NMVVDSFEQL
KRRILP-----
>pCRESS8_CDI43023
NFMYTQDLDHLPSKDELKDRLEKSIDE-----NGKKVRPHFHVMIHFRDAKTISR----VSKIFN-DHQ---QYIEAWHS
IINNG----------FSYLIHETTNARQKVKK----PS-RQ---AIDNFIDDYSNEEITKEELQDKIGVLEMAKHKTLLD
HIEDILAYKNHQQFLKDFK--G---------QKCKVYWIYGVSGIGKTKLVREILEKCI-----LGSQRDHFQEYKGQ--
-GFVVINDLRPNDYDYG--QLLTLLDPWE-IDKMAPARYHDRYL-NARAIYITTPYDPLSFYFECNIA--NQLVDSFEQL
KRRIIP-----
>pCRESS8_CUR41281
IVMYVQQLSYLP-NSLDALERRLKER-----KNGKAIEKHIHLDLRFKTRMSVKS----IAKMLDDE----TERIESLEQ
SWINA----------LSYLIHRTAKSEKSIVG----A---------NKIVDQFLKEEIDYDTAEMLLSAKVLSKNKKILD
DAQNFLNRKHYKEWVKDKKKTN---------QSIIVVWIWGEAGTGKTSFCKDFMNEYE-----ASGHNDPFQNYAG---
EKGLILDELRPRNITYS--DLLKILDPYD-YDKTAVARYHNKYL-MCDYIFVTSPFSPYSFYKNAQVK--DT-NDSLDQL
HRRISIL----
>pCRESS8_CYW87437
NVMFTQQVSYLTSG--NLTDIIKELDE-----NGQLVKPHIHIVLQFDSARSLNN----IAKLLNQP----VQCLEAWRG
SVNNA----------YSYLVHHTQSARQNVTK----QS-KINSVIINNLLDLLYEGAISKKEIEQKLSGSQYAKAKAKIE
AVYLKQLENRASEWQKEMREKN---------EKSIVIWLFGKAGTGKTRLARHYAKQFI-----TGSIRDPFQNYQL---
EPVIILDELRPHQFDYT--DLLKLFDPYN-EQVMASSRYFDKPI-MANIFVITSPYSPYDFFLELRKS--RHQVDSYQQL
MRRLTLV----
>pCRESS8_CYX46115
NVMFTQQVSYLTSG--NLTDIIEELDE-----NSQLVKPHVHIVLQFDSARSLNN----IAKLFNQP----VQCLEAWRG
SVNNA----------YSYLVHHTKSARQNVTK----QS-KINSVIINNLLDLLYEGAISKQEIESRLSGSQLAKAQSKIE
TIHLKRLEQNAREWQKEMRERN---------EKSTVIWLFGKAGTGKTRLAKHYARQFI-----TGSTRDPFQHYQM---
EPVVILDELRPHQFDYS--DFLKLFDPYN-EQVMASSRYFDKPL-MANTYIVTTPYSPYDFFLELRKS--
RHQVDSYQQL
MRRLTLV----
>pCRESS8_EEJ43069
TFMYTQQLQHLPFDVAAFQSRLEN-TV-----DGHPVTSHIHAVLRYQNARSVDS----VAKQVSDK----AQYIEIWNG
NYANA----------YAYLVHKTDGASS--TG----NR-NQ--LAIKYILDEIVSGKITKEEAYQLLPGSLLSKSVNNIN
SAFQIRQVLEAERWRKDKKASG---------ERIHVIWIFGVAGTGKTRFAIDFFKKFK-----SGSSKDPFQGYSG---
QHGIILDDLRPNGLSYA--DLLRFLDPWN-LEAMAASRYFDKGL-QADYVIITSPLGPVEFYDSLFTD--EMDA--FDQL
TRRLETV----
>pCRESS8_EFO53527
NIMFVQQIEYFQSS--NLQDIIEYIGQ-----DGNLVTPHIHLVLQFESARSLNN----LAKLTKQP----IQCFEQWRG
SVNNA----------YSYLVHHTESNEKNVTK----RY-EINTMIIDNLLDLLYIGDITKSEIEQRLTGSQYAKARQKIE
TVYLKRLETQAELWRQEMIDKN---------EIVTIIWLFGKAGTGKTRLARQYAEQFI-----TGSIRDPFQQYNL---
EHVIILDELRPHQFDYS--DLLKMFDPYN-VKAMASSRYFDKPL-LANIYIITSPYSPYDFFLELTKK--RKHIDSWGQL
MRRLSLV----
>pCRESS8_KRK41125
SFMYTQDLDHLPKPEELKDRLEKSTDK-----DDKEVRPHFHVMLHFKDAKTISR----VAKVFA-DQE---QYIEAWHS
TINNG----------YSYLIHETNNARKNVKK----PS-RK---EIENFIDNYSNEKLTKEELQEKIGVLEMAKHKILLD
HIDDILAYKKHQQFLKDFK--G---------QRCRTYWLYGVSGIGKTKLIREILEEFI-----SGSSKDHFQEYKGQ--
-HFIVINDLRPRDYEYG--QLLTLLDPWE-IDKTAKRRYKDVFI-NVCAYFISTPYSPLNFYNECRID--NRLVDTFSQL
ERRVIA-----
>pCRESS8_KRN00682
QFMYTQDLNHLPKKEDLKTILEKSIDK-----DGKTIRPHFHVVLKFKDAKTISR----IAKLFN-DKQ---EYIEVWRN

TIGNA----------YSYLIHETRNARKKVAK----PS-KK---DIDDLIDDYSNEILTKTELQEKIGVLEMAKHKTLLD
HIEDILAYKKHQQFLQDFK--G---------QKCTTYWIYGESGIGKTKMVREILEQVI-----LGSQRDHFQEYRGE--
-EFIVINDLRPRDYEYG--QLLTLLDPWE-IDKMAPARYHDKYL-NARAVYITTPYSPIAFYQGANIV--NGLIDSFEQL
NRRIIT-----
>pCRESS8_KRN07545
VWMYEQQLEHLP-FNIDALKRRVDSVN----AEGKRVKSHIHLVMTFKERVAANS----LGKVFGDQ----PQQFESAKK
GADNA----------FMYLIHQTDNSKRAQID----P---------RDIIELLGAGEIQEEQARAMMMANTYFKYSRRIS
EVALGANKLKFEKWLKNKIETK---------ESIKVIWIYGGAGTGKTRYAVEFADKFK-----TVTTNDPFEGYNG---
QKILIIDELRPETLKYP--DVLHLLDPMS-YEKKTVARYHNSNI-MADFIFVTTPYDPLTFYEKITKL--DRSVDSFEQL
RRRIGLL----
>pCRESS8_KXA58447
AIMYTQQMRLAILS--DWKKEIDRVNE-----DGETVEPHIHLMMYFKHARSPHS----IAWEINERNGKQIERLEFFK-
HLNNG----------FSYLVHQTKDARKQVER----NQ-SKKGELIREYLDMLYDGLLTLEEIESELTGSQYAKASTRLK
AVAEKRQERLGREFLNRMKYEQ---------KTKQVVYIYGESGLGKTRLAKTYAENFV-----TGSSRDPFQSYQN---
QETIIIDELRPDSFRYD--DLLKILDPYN-FDVFLPSRYIDKAL-TAELIFITSPYSPKELY-DNFQT--SKRIDRFDQL
ERRIQTA----
>pCRESS8_SCH55298
AMMYEQQLCHLP-SVDEMYRRIE-ITD-----AGRPAADHLHVMMEFANPRSVRS----VAKSLGDK----AERLEAWKA
GTENG----------FSYLCHRTDGAESRVSK----A----RSHSIKILLDDLLEGRIDREGLISQLSGSEYARAKRQIE
DVYARRLQVSAAEWRAKMRDEG---------RRVQTIWIFGPAGTGKSSLAKQYAQSFV-----SGSTRDVFQGYAG---
QHTIILDELRPSSIPYA--DLLRVTDPYAIHEVMAPARYADKAI-AADLIIVTTPYNPMEFYCEQVRG--ATDIDGFGQL
ERRLSLV----
>pCRESS8_WP_000093566
AIMYTQQMRLAILS--DWKEEIDRVNE-----DGETVEPHIHLMMYFKHARSPHS----IAWEINERNGKQIERLEFFK-
HPNNG----------FSYLVHQTKDARKQVER----NQ-SKKGELIREYLDMLYDGLLTLEEIESELTGSQYAKASTRLK
AVAEKRQERLGREFLNRMKYEQ---------KTKQVVYIYGESGLGKTRLAKTYAENFV-----TGSSRDPFQSYQN---
QETIIIDELRPDSFRYD--DLLKILDPYN-FDVFLPSRYIDKAL-TAELIFITSPYSPKELY-DNFQT--SKRIDRFDQL
ERRIQTA----
>pCRESS8_WP_002821392
GWMFVEQLKSLNRT-----SQIQNVNA-----QGEPIPAHIHLMMSFKSAVTAIT----LAKHFSTT----PERFERNKF
GIING----------FNYLVHRTQNSEQAIKR----SKKSKSRESVNFILDQFGDGLINKATARIRLEGHILAQNSTKLQ
NLDKERAEIDYMNWRKMMIGNH---------FVKTTIFIFGETGCGKSLLAKKIASQYF-----SGGSNDPFQDYEG---
ERAVILDELRPGIIEYP--DLLRILDPYS-WDTATHSRYHNSKL-QAQLFIITTPYDPYFFYRFTKDL--VRFMDPFEQL
NRRISMT----
>pCRESS8_WP_003549058
QFMYTQDLDHLPKKEDLKTLLEKSIGK-----NGKTIRPHFHVVMKFKDAKTISR----VAKLFN-DKQ---EYIEVWRN
TIGNA----------YSYLIHETSNARKKVAK----LS-KK---DIDDLIDDYSNEILTKTDLQEKIGVLEMAKHKTLLD
HIDDILAYKKHQKFLKDFQ--G---------QKCTTYWIYGESGIGKTKMVREILEQVV-----LGSQRDHFQEYRGE--
-EFIVINDLRPRDYEYG--QLLTLLDPWE-IDKMAPARYHDKYL-NARAIYITTPYSPMAFYNESGIV--NSLIDSFEQL
NRRILS-----
>pCRESS8_WP_003665528
VWMYTQQVEDLPFDSIDALIRRINKK-----GKK--VTPHVHVGFTLTKRTTISR----MSKILNDR----TQQITSVAN
STKNL----------MGYLIHHTREAE-EITS----T---------RDILDEYANENISRDQAESLLKGSDLAHNLRNLD
ALDSYILEEKRRRWVQKMKKAK---------KPIYVVWLSGAAGTGKTTYAKRYAEKFV-----TTSQNDPFQGYRG---
QQVLIIDEIRPETLSYA--DLLQICDPYL-YEKNLTARYRNPSF-QSSIVFLTSVYTPLEMYNAMRVK--RK-IDTFNQL
KRRIGMN----
>pCRESS8_WP_004900270
QFMFVQQLRYLGYDLTALNERIDILTE-----DGELIEPHLHLALRFENPVSLKR----LAESLETE----PQYIAQWKG
AANNL----------YSYLIHRTETAEQAIQG----RK-GKRGTLLNETLDALLEGNITLFDAFEILPGRIAGKHRKKLE

SAYQTRMELNAKVWLENKKALG---------KPIQVFWFYGPAGVGKTRYAKQYLSRFI-----SGSNRDPFQNYSKS--
AHKVILDDIRPNTFSYE--ELLRIFDPWN-MEVSVGSRYSDKNL-QVDIFIITSPLAPDVFVDTLQVF--ELDDN-FDQL
LRRLTTV----
>pCRESS8_WP_006499656
VLMLVQQVQLLPNNPKHFLKSKCEKS-----KNNDLVVPHYHLVMQFDHRVDVAA----VAKIFEQG----IEHFESTKT
AAKNS----------FAYLVHATDNSE-SELS----T---------ADILDGVAEGNITKDQAFDMLRARILVHNKKSVE
TMAEEYQRKHHLEWLKKRNEEG---------KGIPAVWCFGQGGTGKTSYAKHFAEEFV-----TSGSNDPFQGYQG---
EEVLIIDELRPDVLPYS--DLLQLLDPFN-FEKRLKARYFNPFF-SSNFIFVCTVMGPIEFYNSMAIA--HKNIDTFEQL
RRRLAMV----
>pCRESS8_WP_007125042
QFMYVQDLNHLSKESDFKQILNQSKDQ-----EGKIIRPHIHAVLKYENPQKLST----IANLFN-DQA---QYVDVWKG
RIANA----------YSYLLHETEEARTKISK----S--PK---YISSLINQYAEGKIQYQELEDQIGISQLARRKKLID
QIDELKADKEHKEWLKKFE--G---------KPMHTLWLYGEAGVGKTRYAEYLLRKVI-----LGSSRDYFQTYNGE--
-HFIILNDLRPNDFNYS--DLLRILDPYQ-HDKAAPSRYRDKKL-SVEEIIITTPYSPKDFYRTTKID--DRQVDTVDQL
LRRIQP-----
>pCRESS8_WP_008469878
QFMYTQDLDHLPNIKSLQSLIKTEKNE-----DNTPIRKHLHLVLKYKNPQTLHH----ISNLLT-DKD---QYFEIWNG
RINNA----------YSYLIHATNEAKDSISN----S--NN---KVQTYIQRYAEEEISYKQLIDQIGLINIAKNKRLID
TIQNLLISKHHQEWLHNFE--G---------KKMICLWLWGDAGVGKTTYAKKLLSQIV-----LGSSNDYFQNYTDE--
-SFAIINDLRPEDWKYA--DLLRLLDPYE-HNKMAPSRYHDKEL-NLEMIIITTPYSPQSFYHNSRIF--NPKIDSFEQL
KRRIFP-----
>pCRESS8_WP_008472153
NFMYVQQTKYLKKPEEFSEVLNGIEG----------VEEHYHVILHYEHSSRIST----VANIFD-DDP---ERVQIWDN
RWNNA----------CGYLIHATKNSQSRVSG----A---K---QIENVIKEYGNGDIDRDELELKLGDAELAKNHIWIS
RIDDIRAQRKHEEFLKDFE--G---------HAQETIWLWGAAGVGKSRYADFLTQGAK-----LGSSRDYFQDYHGE--
-SFVILNDLRPNEFSYA--DLLRITDPYQ-HDKSAPRRYHDLKL-NLKMLIITSPYSPDDFYEYCKVN--NYQIDTFDQL
KRRIHA-----
>pCRESS8_WP_010817837
NVMYEQQLHHLPTKRRTLNSIIKQVND-----EGVPVEKHVHVVLQFQHARSLEN----LARLIKEPQ---VSAFQKWHG
NINNA----------YSYLVHRTTDASKKINK----SN-KQRNEIIKQLLDRLGAGELTREEVISNLTGSQFAKAKKHIQ
DVHEQVQADKAKIWLEKRKEKG---------EPITVIWIYGQSETGKSLLAKKYAAKFI-----TGSSKDSFQYYQG---
EHIVILDELRPKTFPYD--DLLKMLDPFG-ENPKAPSRFFDKSL-MVDVFIITSPYSPKQFYDEIFKR--KKTIDSFKQL
QRRISYV----
>pCRESS8_WP_011254167
QFTYVQDTDHLKEPKQFQDFLSKSADQ-----NNK-IRDHYHVVLKYANPQTISK----IANIFK-DKE---QYVQIWNG
RIDNA----------YSYLIHETSDARTSIKR----KSSKN---DIEEKLKDYAENIISLYELRKSIGAFTTPSIQRHIK
EIKKLHDEDNHQKWLRSFD--G---------KKMKVIWLYGEGGTGKTRCARAMTKDVV-----LGSSNDYFQAYDGQ--
-RVIIINDLRPSDFKFG--DLLKLLDPYE-HSKEAPRRYRNVKL-NLEKIIITTPYSPISFYNHCYIE--DKKIDKIEQL
TRRITQT----
>pCRESS8_WP_012845653
NFMYTQDLDHLPNKDTLKTRLEKSVDE-----NNKKIRPHFHVMLRFKDAKTISR----ISKIFN-DKQ---QYIEVWKN
SINNG----------YSYLIHETSKARRKINK----PS-KQ---AVDNYIEDYANEIISKEDLQNNIGVLEMAKHKNLLD
HIEDILAFKKHQKFLKEFK--G---------KQCKVIWLYGKAGVGKTRLIRNFLEHII-----LGSQRDHFQEYKGQ--
-NYIVINDLRPNDYEYG--QLLTLLDPWE-NDKMAPARYHDKYL-NAKAIFITTPYSPKDFYNTCNIE--NIFIDSFDQL
KRRIIS-----
>pCRESS8_WP_013641468
NFMYVQQTKYLKKPDQFIELLNGRDG----------VEEHYHVILHYKYAARLST----VSHLFD-DDP---ERIQIWDD
RWNNA----------CGYLVHATSNSQSRVSG----S---K---NIEKVITQYGNYEISREELELKLGDAELAKNHVWIS
RIDDLHAEREHENFLKEFK--G---------RAQETLWLWGEAGVGKSRYADFLTKGAK-----LGSSRDYFQDYKGE--

-NYVILNDLRPNEFSYA--DLLRLTDPYQ-HDKAAPRRYHDLKL-NLKTLIITSPYSPEDFYEYCKVD--NYQIDTFEQL
KRRLHV-----
>pCRESS8_WP_013641481
QFMFVQDVEHMNKFEELPNILKSENDK-----EDTKVRDHIHVVLKYRNPQTVAH----VAKLFK-DKS---NNVQIWIG
RINNA----------YSYLVHNTDNAEENVSL----AK-QR---NIKEAINGFAEGDIDYKELIETLGIVNVAKNRNLID
SIQKIREQVIQHEWWNQFN--G---------KQMASLWLWGEAGVGKTTYAERILSNIV-----LGNSNDYFQYYNGE--
-HYIILNDLRPGDLKYA--DLLRILDPYA-IKYTFG-RYHNHPL-LAEMIIITTPYSPREFYKNTRIA--NRKIDSLTQL
QRRIFE-----
>pCRESS8_WP_014567781
QFMYMQDVDHLKKEKSLKSILNKSIDE-----NGKLIRPHYHVILKYEYPRSILS----VAQIFK-DQT---QYVEIWSG
RIANA----------YSYLIHETEEARKKIKK----S--SK---YVMEMIERYANNKLTYEELAKELGVMPMAKHQQLID
RISQVQEEEAHKRWLTKMK--G---------KSMKVIWLYGAAGVGKTRMAEIMLSKVI-----LGSSRDYFQDYHGE--
-HYIVLNDLRPNDFPYS--DLLRLLDPYQ-HDKAAPSRYHDKKL-SAEEIIITTPYSPYDFYRNISIH--DKSVDTIDQL
LRRVIP-----
>pCRESS8_WP_016226904
NMMYTQQIRHLPVSTDQLVSRVK-VNE-----QGEPAEDHVHVMLSFENARSINS----IAKELGDE----PQSIEMWKG
KAENG----------YSYLIHATKDSTEEVER----S----RQTASKILLDSLYKGEISKEELEKRLSGSQYGRMRRQIE
DVWCKHLQFQAAKWREEMKKSG---------KRIKVIWISGEAGTGKTSLAKEYAEKFI-----TGSSRDIFQNYSG---
EHTIILDEFRADMMKYP--DLLRILDPFG-SQVMAPSRYNDKPL-ACDLILITSPYNPVEFYRQLFS---RAFVDSLEQL
LRRISLT----
>pCRESS8_WP_016356676
NFMYENQLKYMKEDINMLAKYVEETIN-----ESELVAPHYHIALKFENPRNVNS----VAKKFN-DLP---QNFEIWLN
RPNNL----------YSYLIHKTSDATKSIHR----GRNEE----IRHLIDSFGNGEITLKLLMEELSPTEYARNENQIN
IIKKLLANKRFEAFKKRMDSEE---------KRIEVFYLFGGTGTGKTRFAKTRYK-YI-----TGSNRDLFANYDGE--
-TVVILDELRPNSISYN--ELLKITDPFN-FENVVGSRYLDKKL-VAETIVITTPFSPEEFYQTLKGE--QTNIDKKEQL
FRRINV-----
>pCRESS8_WP_016622553
NFMYENQIEYMKKEIDKLSKYVKEFIT-----ENKLVAPHYHIALKFENPRKVNN----VAKVFN-DSP---QNFEIWLN
RPNNM----------YSYLIHKTNQATKSIKR----NRKEE----IQHLIDSFGNGKVTLNQLMNELSPTEYARNEHQIT
IVKKLLANQRFEEFKSRMENEQ---------KKIEVFYLFGDTGTGKTRFAKSRYK-YI-----TGSNRDLFANYDGE--
-SILILDELRPNSISYN--ELLKLTDPFN-FENVAGSRYFDKKI-VAEKIIITSPFSPNDFYRALKSD--KSDVDKKEQF
FRRINV-----
>pCRESS8_WP_024410839
NVMFVQQIEYLNSS--NLQGIIHDKGQ-----NGDTVEPHIHLVLQFESARSLNN----LAKLTEQP----IQCFEQWRG
SVNNA----------YSYLVHHTESNEKNVTK----RH-EVNTMIIDNLLDLLYTGEITKSEIEQRLTGSQYAKAKAKIE
AVHLKQLENKSCEWQKEMREKN---------EKSIVIWLFGKAGTGKTRLARRYAKQFI-----TGSIRDPFQHYQM---
EPVIILDELRPHQLDYS--DLLKMFDPYN-VKAIASSRYFDKPL-LANVFIATSPYSPYDFFLELRKG--RHDVDNYQQL
MRRLTLV----
>pCRESS8_WP_033683822
NVMFVQQIEYFQSS--NLQDIIEYIGQ-----DGNLVAPHIHLVLQFESARSLNN----LAKLTKQP----IQCFEQWRG
SVNNA----------YSYLVHHTESNERNVTK----RY-EINTMIIDNLLDLLYTGNITKSEIEQRLTGSQYAKARQKIE
TVYLKRLETQAELWRQEMIDKN---------EIVTIIWLFGKAGTGKTRLARQYAEQFI-----TGSIRDPFQQYNL---
EHVIILDELRPHQFDYS--DLLKMFDPYN-VKAMASSRYFDKPL-LANIYIITSPYSPYDFFLELTKK--RKHIDSWGQL
MRRLSLV----
>pCRESS8_WP_034540695
VLMFEEQDQHFKDSSSIEEFLTDRCEVT---NNGQPVKPHYHLTMYFDNRPMVSS----IADALDTT----ENQIEKVET
ARVNA----------LMYLIHATRNAM-LNDD-----P--------TDILDDLGNGKIVRTQARSRMMAQVLAKYNRKID
EIAEASLAIQNEVWRKEHEDSH---------SELKVYWFFGQTGTGKTRFAKYLAKEFV-----TGARRDAMQDYEG---
QHLIIWDELR-DDVEYS--ELLRLLDPYN-FDKAISSRYYNKNL-MPDIVIITSPYSPDELYSVMRIT--DRKIDKVDQL

VRRVPII----
>pCRESS8_WP_042900192
AIMYTQQMRLAILS--DWKKEIDRVNE-----DGETVEPHIHLMMYFKHARSPHS----IAWEINERNGKQIERLEFFK-
HPNNG----------FSYLVHQTKDARKQVER----NQ-SKKGELIREYLDMLYDGLLTLEEIESELTGSQYAKASTRLK
AVAEKRQERLGREFLNRMKYEQ---------KTKQVVYIYGESGLGKTRLAKTYAENFV-----TGSSRDPFQSYQN---
QETIIIDELRPDSFRYD--DLLKILDPYN-FDVFLPSRYIDKAL-TAELIFITSPYSPKELY-DNFQT--SKRIDRFDQL
ERRIQTA----
>pCRESS8_WP_046025501
VMMFEEQDKFF-EASDIGEFLKQSCQK-------EPAEPHYHLTMYFKGRPMVSS----IAEMLGTT----PKQIEKVET
ARKNA----------FMYLIHATLNAL-MQVT-----P--------EGILEDLGVGKLTKTQAREKFMATVLAKYNRKIN
DVAEASLAIQYTKWRKEREAKH---------YQLMTFWFCGPTGTGKTRYAKYLAENFV-----SGGQRDAMQDYEG---
EHLIMWDELR-NNVYYL--ELLRLLDPYN-YDKAISSRYFNKNL-MPEVMIITSPYRPDELYKIMDIS--DRKQDKLNQL
TRRVPLI----
>pCRESS8_WP_046324376
QFMYMQDFAHLKDFTELTNILTKALDQ-----GNRLIRKHLHVVLKYANPQILSH----VVRLFN-DKP---QYLEVWQG
RISNA----------YSYLIHATLEAQSSVNQ----SRLSK---VVANFLTQYANEEIDYQELADIIGLAEVAKRKSVID
NITKLIADKKHEEWLHEYH--D---------RKAETIWLWGEAGVGKTRYANKLVRGAI-----LGSSRDYFQDYHGE--
-HYVILNDLRPNDFRYA--DLLRLLDPYE-HDKVAPRRYHDVKL-NVEMLIITTPYSPFEFYQHVKIA--DEKTDTFEQL
KRRVHA-----
>pCRESS8_WP_046923918
VFMFEQQVGYMPPIDQLYEKRKE---------DGTLKTPHYHMSMYFDHRKTVNS----VAKMLNSK----PQQIESTVE
AKNNA----------FAYLCHRTKNAE-SKVN----NA--------NDILELLNDRHITKDQAIERIKGVAYSRNAKKIE
TIAYANLRADYKDWLKRMKEKK---------RDVKVLWLYGYAGTGKSHFATDLATDKK-----LTSKHDDLTDLSIK-D
QDVLIFDDFRPDTLPYS--SILQIFDPLN-LGVSLDARYHNAYL-MSEYIIVTTPFSPYEFYQSMYIR--NRKIDTFEQL
SRRIYAT----
>pCRESS8_WP_049150683
QWMYVQYFKYLPKINNLTEILTKDTKE-----DGSLISPHVHVLIKYSNPQTLKH----VANLFK-DKP---QYFDIWKG
RINNG----------YSYLIHSTSEAQRQVLN----KKISN---NSNLFIEKYAEGEISYNDLENIIGVVQVARHKTIID
HINQINASNKHKEWLKSFK--G---------KPMETHWLWGAAGVGKTRYAKWLAKNAI-----LGSSRDYFQEYHGE--
-HIVILNDLRPNDFNYG--DLLRLLDPYE-HNKMAPRRYHDVYL-NLEMLIITTPYSPWSFYKQCKID--
NPEVDTYKQL
NRRVHA-----
>pCRESS8_WP_049523992
NVMFVQQIDYFKTP--NIQAIIKEIGS-----DGTAVAPHIHLILQFESARSLNN----LAKLTSQP----IQCFEQWRG
SINNA----------YSYLVHHTSSDEKNVRK----RN-EVNSMIIDNLLDLLYAGEITKSEIEERLTGSQYAKAKHKID
TVHLKRLEIQAESWRHEMKNKN---------AIVTIIWLFGKAGTGKTRLARQYAEQFI-----TGSIRDPFQQYNL---
EHVVILDELRPHQFDYS--DLLKMFDPYN-DRAMASSRYFDKPL-LANVYIITSPYSPYNFFLELTKK--
KQHIDSWGQL
MRRLTLV----
>pCRESS8_WP_050444210
NVMYEQQLRYLPTKRKTLNSIIKLIND-----KGEVIEKHVHVVLQFQHARSLKN----LARLLKEPQ---VSAFQKWHG
NVNNA----------YSYLVHRTADASKKITG----SN-RQRNEIIKQLLDRLGAGELTREEVISNLTGSQFAKAKKNIQ
DVHEQVQADKAKIWLEKRKEKG---------ESTTVIWIYGQSGAGKTLLAKKYATKFI-----TGSSKDSFQHYQG---
EHIVILDELRPKTFPYD--DLLKMLDPFG-ETPKAPSRFFDKSL-MVDVFIITSPYSPKQFYDEIFRR--KKTIDSFKQL
QRRINYV----
>pCRESS8_WP_050492321
AIMYTQQMRLAILS--DWKKEIDRVNE-----DGETVEPHIHLMMYFKHARSPHS----IAWEINERNGKQIERLEFFK-
HPNNG----------FSYLVHQTKDARKQVER----NQ-SKKGELIREYLDMLYDGLLTLEEIESELTGSQYAKASTRLK
AVAEKRQERLGREFLNRMKYEQ---------KTKQVVYIYGESGLGKTRLAKTYAENFV-----TGSSRDPFQSYQN---

QETIIIDELRPDSFRYD--DLLKILDPYN-FDVFLPSRYIDKAL-TAELIFITSPYSPKELY-DNFQT--SKRIDRFDQL
ERRIQTA----
>pCRESS8_WP_051448806
--MYQQQLSHLPSKRKTLNSIVKSVNE-----EGDSIEKHVHVVLQFENQRSLER----LAKVLNEPQ---ISSFQQWRG
NVNNA----------YSYLVHQTKEATKKIEQ----KI-KMKSEIIKSLLDQLGAGELSKDEVILNLTGSQFAKAKKQIN
DVYQQVQEQKSKFWLEEQKSKN---------EPITVIWIYGSSGTGKTALAKKYADEFI-----TGSSRDSFQHYDG---
EHLVILDELRPTTFNYD--DLLKMLDPFG-ENPKAPSRFFDKSL-MIDIFIITSPYSPKQFYDEIFRH--KKTVDSFGQL
QRRITFV----
>pCRESS8_WP_056985318
QYMMVQDLDKLPDLDKLKEILSGLKSE-----NGGLVTPHVHAVIKFENERMLDT----LADTLK-VKP---QYLQVWKG
RINNA----------YSYLIHLTSGATNRVSK----Q-------EIKDALNMYANGGLSQTELKTKIGNLAYAQNLETIK
KLNTVLDNQAHQEWLKSFQ--G---------QKMTVDWYYGKAGVGKTRLALKEAKECV-----LGSSNDYFQDYDSQD-
-HVVILDELRPNDLKYG--DLLKIMDPYQ-HDKHAPRRYRNVAL-NIEKLIITTPYKPETFYKMTKIA--DRRVDTVEQL
KRRISKV----
>pCRESS8_WP_057827085
GVMYVQQLDKMKFS--SLDALKRRTNG-----DNELVKPHVHVMLEFESPRMLSA----IAKELDDA----PEHFEHNKN
GINNG----------FAYLVHRTKNAKTEVNE----G--RYEKMGISNLLGDFINGKLTKIEAKEIAKPGQFAHICKRID
ESEVQMEELKADKWIQEMQGKH---------EAKKVFWIYGPAGVGKTTLAKMITESFT-----SGSSRDYFQNYHG---
EHCILIDELRPNVIDYS--DLLRILDPYN-YNCNVPSRYHDHRL-TANAIVITSPFSPGDFYIHQRDL--NTEVDAFKQL
HRRLNYV----
>pCRESS8_WP_057827851
AYMYVQQVDNLPAKGQSLDALIKRSDE-----GGNTIKPHIHVMLELDKQRSVNK----IAKALDDS----SERLEYKRH
GIENG----------YAYLIHRTQGAQQKVRV----VN-KKSKEFIKEVLNNYLAGRISEIEAKRQVLPLMLPRFLRQLD
AIKNTKLEVEADEWFQSRSKGD---------TPKSVIWISGSGGTGKTVLAEMVARNYL-----SGSDRDYFQDYNG---
EHCVILDEFRPDKITYS--DLLKMLDNNR-FDVSAPARYHDKKI-LADLIVITSPYDPARYYQNVEDI--RPAIDGFDQL
DRRLTMV----
>pCRESS8_WP_057906729
AYMYVQQLDKLPAKDQSIDVLIKRSDK-----DGNAIKPHVHVMLELDKQRSVNK----IAKALDDS----SERLEYKRH
GIENG----------YAYLIHQTQGAQQRVRV----TN-KKSKEFIKEVLNNYLAGKISEIEAKRKVLPLMLPRFLRQLD
AVKSTKFEIESDEWFKNRSENN---------KSKSVVWISGHGGTGKTVLAKMIAENYM-----SGSDKDYFQDYNG---
EHCVILDEFRPDKISYS--DLLKMLDNNR-FDVNAPSRYHDKKI-LADLIIITSPYNPARYYQNDESI--RPTVDGFEQL
DRRITMT----
>pCRESS8_WP_060461663
QFMYVQDLDHLKDEKDLSTILKQSKDQ-----DGKTIRPHIHVVLKYENPQKISS----VASMFK-DQP---QYVGVWKG
RIANA----------YSYLLHETEEARAKVTK----S--PK---YVSSLIDQYAESKITYDELESQIGISQLARRKKLVD
QITELRAEKEHEKWLKDFK--G---------KKMKVLWLYGAAGVGKTRFAEYLFRNSI-----LGSRRDYFQDYCGE--
-HFVILNDLRPRDFSYS--DLLRILDPYQ-HDKSAPSRYHDKKL-NLEEIIITTPYAPTDFYKYVFID--DRRVDTDEQL
LRRIQP-----
>pCRESS8_WP_062359070
TFGYTQQLSHLN-RLEDFPRLMEERTA-----DGVLKASHVHVVMKFENPRSLQA----VSKIFHDS----PQYVEKTRN
GYNNM----------LAYLTHRTKGATAKVEQ----RD----SKTLDELLERLMDYSITYEEAVGELTASEFSRYHQRLK
VTAEYGRQQYAKRWIKEHEDNH---------KPIEVIWITGPAGSGKTVEAKKIAREYV-----AVGVRDPFQLYTD---
ENVIILDDLRAKTFDYN--TLLQLLDPFN--STMADARYSNKTL-IADTIIITSVYSPLDFYHMLVPE--IYDVDTYKQL
ERRIS------
>pCRESS8_YP_006939186
TQQLKYLNSIEQLKNNLENDAYIQDFAMILDENNQNVAEHLHVFIKLNQQKTIDY----VADLVDDKA----QYIENKSR
NEQNG----------YLYLLHKTKSAEHKHYENNLKKYQSKRKTVVQSILNDYADRIIDEKELKDSLTNLELAKNKKLIN
DIKQVLIEFDFQTYLEQERYKN-----------KQVVWIFGKSSTGKSMMSQLLAKDYV-----TSSNRDPFEDYQNQ--
-KVLIIEEFRNENIGTN--ELLQLLDKTN-GQVRVGSRYSNKKIMADLIIINTIYEPKYFMFDEPIYQLLRRIDKLVKLD

NQKIET-----
>pCRESS9_ATL14544
NTKDIFLTYSKCPLGKDKIHNHIKELMVSENHQDHKE-IHTHVLFQLTKRFNIKS-----ERFFDIEG--FHPKIENARD
IEKSI------------DYIKKDG----DFIV---------RKSLDNFIKNLDRDFYYEQIDLIEKILKKKFIKQTELLD
KGYDLDTFKVDKSTQEIGSDQL--KVK-----RPLSIVIEGPSRLGKTEFIISYNHFNY-----IRGSFDFSKENYND-S
FKVDVYDDISINYISAG--LLKNIIGGQR--GFIVDVKYSPKRLLSGNKLSIFLVNPD--ISFESWCEEDEKYAGETYIK
DNCIF------
>pCRESS9_BAD36752
KAQNIFLTYSQCDLSKEEIKTFIINICDEENHQDHKG-KHHHVFFQLNKRLQTRD-----LTIFNIPKNNYSPHIEPIKD
TTDVR------------NYVKKDG----DFII---------FKKLKLYAESLEPNYAFKN--------AKRFKNMVFVFE
SIFDFCTFKKIPILTSTYETQK--EQSS-ISKRFKTLIVEGNSKSGKTQFFKSVTPFNY-----IKDDVDFSDENYDE-D
KYVNIYDDIDIYDIARN--LTKVVIGNQK--DSIVNMRYKPRTKIKGSDISIMLVNED--TSIEKYCFDNFKRGRKEYIR
ENAIF------
>pCRESS9_CBX25033
RTKDFFMTYSQCDLGKEKIFNHLKQLMASENHADNNG-VHSHVFLQLKKYLDIQS-----GRFFDIDG--KHPEIERART
VQGSV------------DYVKKDG----DFII---------KKSLNDFILKTDRDFYYEKIDLIKKILNERFTKKKELED
EDYSFNSFKSNSKTNEIIQTQL--SVS--LGKRPKSIVIEGPSRLGKTEFILSYTHYNY-----TRGEFNFSKQSHKN-A
YKINIFDDISLTKIKEG--LIKDIIGGQK--GFTVDVKYAPKRNISGKKLSIFLVNPD--ISFENYCEWSRNNGHKFYIE
DNCIF------
>pCRESS9_KXT29014
KTKMVGLTYSRFPVKEKFIQLLKELFNSKCNDYHMDSGLHIHALLVFDKKIQLRD----AQKVFALPN------YQTAKF
GHLDR----------FRAYIIKKGNFIENGIP----DIERLVLKSGLIVAEKQLSFGYKLDNVLYWDKLRDKMSKTFVFE
LIYDFDSLFEPFNMVEKMVLKLMNSG-----VRKKSLFIVGGTGIGKTRMIKTILKYSY-----IKGKIDFSPKKFDD-S
RPVVIMDDITLQYLPED--GFKNFIGNGD--
TTEVDVKMQKTATITGGKLFIYIVNKHPEKWVKNDEYDHIYIRKNIEVI
EFDEKDKL---
>pCRESS9_KXT29032
HSRNIGFTYPNLSLSKEEVQKIFIQKESRSRELHEDGEPHIHILIQLNKKTEFCN----AREFFALPT------FNKNNE
TPEHWY-----------RYIGAYGDVLDDGIFKLLISAFEAEKALNQYLQELDVVIYYKQFPIRDRVIQENFYPKSPVVK
REHSLQTFRLYHEKVQLIIKEQFN------SKSPLTIVLEGLTQIGKTDLAELIQPYN------YTKIDFNFSRENYNDS
YKICIYDDMGMEEVSKK--LMHALIAGRG--
SFQTREPYGKKRTISGNKLNIFIVNRNKSFKGWIEKNKEWKRFEYVEPN
VIIIDLF----
>pCRESS9_WP_011264167
QAQNIFLTYSQCDLSKEVIKTFNINLCNEENHQDHKG-KHHHVFFQLNKQFRTRD-----LTIFNIPKNIYSPHIEPIKD
TTDVR------------NYVKKDG----DFII---------FKKLKLYAESLEPNYAFKN--------AKRFKNMVFIFE
SIFDFCTFKKIPILISIYETQK--GQSS-ISKRFKTLIVEGNSKSGKTQFFKSVTPFNY-----IKDDVDFSDENYDE-D
KCVNIYDDIDIYDIARN--LTKVVIGNQK--DSIVNMKYKPRTKIKGTDISIMLVNED--TSIEKYCFDNFKRGRKEYIR
ENAIF------
>pCRESS9_WP_011412950
NTKDFFMTYSQCDLGKEKIYHHIKQLMASENHADNNG-VHSHVFLQLKKRYQVVN-----NRFFDIDG--
KHPEIERART
VQGSV------------DYVKKDG----DFIV---------KKSLDDFILKTDRDFYYEKIDLIKKILNERFTKKKELED
EDYSFNSFKTNSKTNEIIQTQL--SVS--LGKRPKSIVIEGPSRLGKTEFILSYTHYNY-----TRGEFNFSKENHKN-A
YKINIFDDISLTKIKEG--LIKDIIGGQK--GFTVDVKYAPKRNISGKKLNIFLVNPD--ISFENYCEWSRNDGHKFYIE
DNCIF------
>pCRESS9_WP_011412958
NAKDIFLTYSKCPLGKDKIHNYIKEIMISENHQDHKE-IHTHVLFQLTKRFNIQS-----DRFFDIEG--FHPRIETARN
IEKSI------------SYIKKDG----DFIV---------KKSLNDFIKILDRDFYYEKIDFVEQVLNKKFTKKKELED

EDYSFDSFKSNSKTNEIINQQL--SVS--LGKRPKSIVIEGPSRLGKTEFILSYTHYNY-----TRGEFDFSKQNHKN-A
YKINIFDDISLTRIKEG--LIKDIIGGQK--GFSYNVKYAPKRTIAGKKLSIFLVNPD--ISFENYCEWSRNKGYKYYLE
DNCIF------
>pCRESS9_WP_012662291
QAQNIFLTYSQCDLSKEEIKNFIINLCNEENHQDHKG-KHHHVFFQLNKRFKTRD-----LNIFNIPKNIYSPHIEPIKD
TTDVR------------NYVKKDG----DFII---------FKKLKLYAESLEPNYAFKN--------TKRFKNMAYIYE
SIFNFCTFKKIPILICIYKTQK--EQSV-ISQRFKTLIVEGNSKSGKTQFFKSVTPFNY-----IKDDVDFSDENYDE-D
KYVNIYDDIDIYDIARN--LTKVVIGNQK--DSIVNMKYKPRN------------------------------------
----
>pCRESS9_WP_013747472
QTKDIFLTYSKCPLGKDKIHNYIKELMISENHQDHKE-IHTHVLFQLTKQLSIRN-----QRFFDIEG--FHPKIENARD
IEKSI------------DYIKKDG----DFIV---------RKSLDDFILNIDRDFYLEQSELIDRILNKKFTKKKELED
EDYSFDSFKSNSKTNEIIQTQL--SVS--LGERPKSIVIEGFSRLGKTEFILSYTHYNY-----TRGDFDFSKQSHKN-A
YKVNIFDDISIPQIKEG--LFKQIIGGQK--GFKYNVKYAPKRTIAGKKLSIFLVNPD--ISFENYCEWSEDNGHKFYIK
DNCIF------
>pCRESS9_WP_015060110
QAQNIFLTYSQCDLSKEEIKTFIINLCNEENHQDHKG-KHHHVFFQLNKQFRTRD-----LTIFNIPKNIYSPHIETIKD
TTDVR------------NYVKKDG----DFII---------FKKLKLYAESLEPNYAFKN--------AKRFKNMVFIFE
SIFDFCTFKKIPILISIYETQK--EQSS-ISKRFKTLIVEGNSKSGKTQFFKSVTPFNY-----IKDDVDFSDENYDE-D
KCVNIYDDIDIYDIARN--LTKVVIGNQK--DSIVNMKYKPRTKIKGTDISIMLVNED--TSIEKYCFDNFKRGRKEYIR
ENAIF------
>pCRESS9_WP_015083745
QTKDIFLTYSKCPLGKDKIHNYIKELLVSENHQDHKE-IHTHVLFQLNKIIQIEN-----QRFFDIEG--FHPKIENARD
IEKSI------------DYIKKDG----DFIV---------KKTLDEFIINLDRDFYSEQIDLIEKILKKKFIKQAELID
KGYDLNTFKVDKSTQEIGTNQL--KVK-----RPLSIVIEGPSRLGKTEFIISYNHFNY-----IRGSFDFSKENYND-S
FKVDVYDDISMNYISSG--LLKNIIGGQR--GFIVDVKYSPKRLLSGNKLSIFLVNPD--ISFESYCEEDEKHAGETYIK
SNCIF------
>pCRESS9_WP_017193171
NSKDIFLTYSKCSLGKNVIHNHIKNLMNEENHADHKE-IHTHVLLQLEKRSNIKD-----ARFFDIEG--FHPRIENAQH
IEKSI------------DYIKKDG----DFIV---------KKTLDEFIKKMDRDFYYEQIELIERILKKKFIKKEEMNE
EIYDFDSFKDNEITRKIIQKEK--DVH-----RPRSLVIEGLSRIGKTEFIISYNPFNY-----IRGSLDFSKEIYKN-E
YKINVFDDISIFEIKHG--LLKNIIGGQR--GFNADIKYAPKRRIAGNKLNIFLCNED--ISFVRFCKKNKEMGGKEYIE
KNCLF------
>pCRESS9_WP_017193695
SSKDIFLTYSKCSLGKNVIHNHIKNLMNEENHADHKE-IHTHVLLQLEKIFQTEN-----ARFFDIEG--FHPRIENAQH
IEKSI------------DYIKKDG----DFII---------RKNLDEFMIKIDRDFYLEQIELIERILKKKFIKKAEMND
NIYDFDSFKDNEITRKIIQKEK--DVH-----RPRSLVIEGLSRIGKTEFIISFNPFNY-----IRGSLDFSKEIYKN-E
YKINVFDDISIFEIKHG--LLKNIIGGQR--GFNADIKYAPKRRIAGNKLNIFLCNED--ISFVRFCKKNKEMGGKEYIE
KNCIF------
>pCRESS9_WP_042068233
KAQNIFLTYSQCDLSKEEIKTFIINICDEENHQDHKG-KHHHVFFQLNKRLQTRD-----LTIFNIPKNNYSPHIEPIKD
TTDVR------------NYVKKDG----DFII---------FKKLKLYAESLEPNYAFKN--------AKRFKNMVFIFE
SIFDFCTFKKIPILTSTYETQK--EQSS-ISKRFKTLIVEGNSKSGKTQFFKSVTPFNY-----IKDDVDFSDENYDE-D
KYVNIYDDIDIYDIARN--LTKVVIGNQK--DSIVNMKYKPRTKIKGSDISIMLVNED--TSIEKYCFDNFKRGRKEYIR
ENAIF------
>pCRESS9_YP_001708784
KTKDIFLTYSKCPLGKDKIHNHIKQLMASENHKDHKE-IHSHVLFQLTKSATFNG-----ERFFDIEG--FHPEIEVARD
IEKSI------------SYIKKDG----DFIV---------KKTLDEFIINLDRDFYLEQIDLIEKILKKKFIKKDELLD
KGYDLNTFKFNKSTQEIEVEQL--KVK-----RPLSIVIEGASRLGKTEFIISYNHYNY-----IRGSFDFSKENYND-N

FKVDVYDDISINYISAG--LLKNIIGGQG--GFIVDVKYSPKRLLSGNKLSIFLVNPD--ISFESWCEEDEKYAGETYIK
TNCIF------
>pCRESS9_YP_001708790
NAKDIFLTYSKCPLGKDKIHNHIKELMASENHKDHKE-IHSHVLFQLTKRTTFNG-----ERFFDIEG--FHPEIETARD
IEKSI------------NYIKKDG----DFIV---------KRTLDKFIINLDRDFYYEQISFIKTILNEVFTKKKELED
EDYSFDSFKSNSKTNEIINQQL--SVS--LGERPKSIVIEGFSRLGKTEFILSYTHYNY-----TRGDFDFSKQSHKN-A
YKVNIFDDISIPKIKEG--LFKDIIGGQK--GFKYNVKYAPKRTIAGKKLSIFLVNPD--ISFEDYCEWSENNGHKFYIR
DNCIF------
>pCRESS9_YP_001965305
RTRDIFLTYSKCPLGKEKIHNHLKQLLASENHQDHKE-IHTHVFIQLKKQIEITN-----QRFFDIEG--YHPKIETARD
VEKSV------------SYIKKDK----DFII---------RKEIDDFILKIDRDFYYEQIELIDRILNRRFIRKKELAD
THYQFNSFKTNSETNEIINSQL--LSH-----RPKSIVIEGESRMGKTQFILSYTQYNY-----IKGEFDFSKKTYKD-Y
YKIDVYDDFGVPEISQG--LQKNIIGGQE--CFTCNVKYAPKRQLSGNKLSIFLVNPD--NSFKGYCEWSRNNGHKFYIE
ENCIF------
>pCRESS9_YP_001965310
QTKDIFLTYSKCPLGKEKIHNHIKQLMESENHQDHKE-IHTHVLFQLNKRCNLTS-----QRFFDLDG--YHPKIENTRD
VEKAI------------EYIKKDG----DFVV---------KKTLDDFIINLDRDFYLEQIEFIKRVLKEAFAKKEELAD
DDYSFESFKTNSTTNEIIQSQL--SVS--LSKRPKSIVIEGPSRIGKTEFLLSYTHYNY-----IRGEFDFSKESHKN-A
YKINIFDDISIPQIKEG--LFKNIIGGQR--GFRFNVKYAPKRFIAGKKINIFLINPD--ISFKGYCEWSYKKGHKFYIE
DNCIF------
>pCRESS9_YP_006959585
QAQNIFLTYSQCDLSKEEIKTFIINLCNEENHQDHKG-KHHHVFFQLNKQFRTRD-----LTIFNIPKNIYSPHIEPIKD
TTDVR------------NYVKKDG----DFII---------FKKLKLYAESLEPNYAFKN--------AKRFKNMVFIFE
SIFDFCTFKKIPILISIYETQK--EQSS-ISKRFKTLIVEGNSKSGKTQFFKSVTPFNY-----IKDDVDFSDENYDE-D
KCVNIYDDIDIYDIARN--LTKVVIGNQK--DSIVNMKYKPRTKIKGTDISIMLVNED--TSIEKYCFDNFKRGRKEYIR
ENAIF------
>pCRESS9_YP_006961991
NTKDIFLTYSKCNLGKDKIHNHIKELMASENHQDHKE-IHTHVLFQLTKRLNIKS-----ERFFDIEG--FHPKIENARD
IEKSI------------DYIKKDG----DFIV---------KKTLDEFIINLDRDFYLEQIDLIEKILKKKFIKKDELDE
EAYSFDSFKTNSETNKIIDSQL--NINSKTNKRIKSIVIEGPSRLGKTEFILSYTQYNY-----IRGEFNFSKESHKN-A
YKVSIFDDISIPEIRAG--LLKNIIGGQR--GFEYNVKYSPKRTVMRNRLSIFLVNPD--ISFESYCEWSRDNGHKFYLE
DNCIF------
>pCRESS9_YP_007008175
NTKDIFLTYSKCPLGKDKIHNHIKQLIISENHQDHKE-IHTHVLFQLTKRTTFHG-----ERFFDIEG--FHPKIETARD
IEKSI------------DYIKKDG----DFIV---------KKTLDDFIKNLDRDFYYEQIDLIEKILKKKFIKQAELID
KGYDLNTFKVDKSTQEIGTNQL--KVK-----RPLSIVIEGPSRLGKTEFIISYNHFNY-----IRGSFDFSKENYND-S
FKVDVYDDISMNYISSG--LLKNIIGGQR--GFIVDVKYPPKRLLSGNKLSIFLVNPD--ISFESYCEEDEKHAGETYIK
SNCIF------
>pCRESS9_YP_007008179
NTKDIFLTYSKCPLGKDKIHNHIKQLLVSENHQDHKE-IHTHVLFQLTKPTTFNG-----ERYFDIEG--FHPKIENARD
IEKSI------------DYIKKDG----DFIV---------KKTLDNFIKNLDRDFYYEKIDFVEQVLNKKFTKKKELED
EDYSFDSFKSNSKTNEIIETQL--SVS--LGERPKSIVIEGFSRLGKTEFILSYTHYNY-----TRGDFDFSKQSHKN-A
YKINIFDDISIPRIKEG--LFKQIIGGQK--GFKHNVKYAPKRTIAGKKLSIFLVNPD--ISFENYCEWSENNGHKFYIK
DNCIF------
>PpulchraPlasmids_AAF36422
AAKTFFLTYPQSGL-TKELILLELGKIITDERGED-GYDHFHVLLEAKSRKNYKS-----PRCFDILG--VHGKYETVKS
KKKAL------------KYICKEGDVLLS--------------ENPSVLIF-----EIYNDYLISPKKYKQYILESVKVS
TSPRLWIHKLKMDELMIWAKTI------VSDHISKSLYIHGASGVGKTNMARLMFDDMF-----IVKHVDKLKEADVE-S
SVAIGFDDVNLNKYTRE--DCINIVDPEV--GSQINVKNSMVSLESYVPKVFISNFLPER-VYKG--YDEAVERRLKVIY

LE-----QAIY
>PpulchraPlasmids_AAF36423
NAKIMFLTYPQSGL-TKEVILQELKKKIFDERGED-GYDHFHVLLETKTKKNYKD-----PRCFDILG--VHGKYESTRN
KKRAM------------KYICKEGNVLLG--------------ENPSELIF-----EIYNDYLISPKKYKQYILESVKVS
SSPRLWIHKLKMDELMIWAKTI------VSDHISKSLYIHGAPGVGKTNMARLMFDDMF-----IVKHVDKLKEADVE-S
SVAIGFDDVNLNKYTRE--DCINIVDPEI--GSQINVKNSMISLEPYVPKVFISNFLPER-VYKG--YDEAVERRLKVIC
LE-----QAIY
>PpulchraPlasmids_AAF36424
NARLFFLTYPQSGL-TKELILRELRKIVSDERGED-GYDHFHVLLEAKTKKNYKD-----PRCFDILG--VHGKYETVRN
RKRSL------------KYICKEGDVVLD--------------ENPSRLIF-----EIYVDYLSSPKRYEKFIAEYRKDS
TIPRLWIHKLKMSELMDWAEKI------VEGVVEKSLYIHGKPGIGKTNMARLMFNDIA-----IIKHLDKLKEASVD-Q
SVAIGFDDVNLKKYTRE--DCINIVDGEV--GSQIDVKYGMVVLEPYVPKVFISNLLPEM-VYKG--YDKAVERRLRIIY
LE-----QAIY
>PpulchraPlasmids_ETO15557
QAKNLFLTYPQCTL-QPEEALAILETKLQENHIQ--KGRHLHCYINCKKKVDIRS-----AHRLDLDG--FHGNYQGCRS
LTAVK------------KYCTKEKNYIVN--------------SAINIIIA-----NLARDYIRDSLRIEQSIKRLAKSN
DE---------NYKFKNVNNVL------NWNRSKHVLWLHGKTNTGKTQFALSLFKNPL-----LVRHIDQLKKLDN--S
YDGIVFDDMNFSYWKRE--EQIHIVDVEE--TTAVNVKHGHVEIKKGLPRVFTSNNR----IFN----DPSIKRRIRYIK
I------DDIR
>PpulchraPlasmids_OLY79389
---------AVCLL-TKQEVYDELKKPFTEVSGA--GNPHIHVYLKTGSKLNIKN-----ARYFDIRG--YHGDYRSCLS
LKKIA------------CYVTKGGNFIFE--------------EAMEVIMY-----NLGRDWLRNPMAYEQGIRYIKRRG
TK-------NPNYKFVDLPILS------SWDPLKYSLWLFGGAGLGKIEFAKTLFKNPL-----LVKHIEQLKGLHS--D
HDGIIFDDTKFDRWTRN--DQLLLTDIEN--TNAFGVKYGSVTIPRNTGRIFISNNK----IFQ----YPEIIKRLFIVR
M------KDLK
>PpulchraPlasmids_OLY79419
SAKSFFLTYPNCSL-TKQEVYDEMKKPFEESHEA--GHPHVHVYLKSKNKLNVKS-----ARYFDIGG--YHGEYGTCES
ASAVS------------GYVIKGGDFIFE--------------EAMEIVMY-----NMGRDWVKNPLAYEQGVRYIKRKS
KK-------NPNYKFRELPLVS------FWNPDKRVLWMHGGTGLGKTEFAKTLFYNPL-----LVKHIEQLKDLHS--D
HDGIIFDDTKFAAWDRN--DQLLLTDVEN--DNAFGVKYGAVHIPRLTGRIFISNNP----IFL----DSAIRRRIFYIY
I------DDLR
>PpulchraPlasmids_OLY79699
SGKKAIVVYPGILT-DRDLVLNELKKDRPNLVNEENGEINMHVFLEFSTKVNIRN-----EGYFDILG--IHGIAKTVKD
KTPVL------------RKIFRNDDFLTNGTPT-EILRSKSLQNSLKILLR-----ILSRSLLMNPSRLFENIRKLTPRK
NS-------ELNLRFREVPQIS------IWDRKKSSLWLKGPPGTGKTTFAKSLFNNPL-----IVNINEALLNFNI--F
HDGIIFDDMNFTKYTVE--FQIHMTDVTD--EKVIKIGNSKINIPAETPRVFTSNVP----IFS----EYRVRRRVALIK
INKLQLTEDGR
>PpulchraPlasmids_OMJ09562
EGKSFFLTYPRCNL-DLNVILTELEKIMSEKHAS--GEPHIHVFLSRFEKKATRN-----PKFFDILG--FHGNYQTCRS
SKSVM------------AYVAKDKDYIVE--------------EALEVAKN-----DLGRDYIRNAELFEKSARYLKERP
VEP-----FDPSHRFIPPHGIR------KWDRTKQALMLFGKTGTGKSSYAETLFNNPL-----VVNSLEDLKRLRP--V
HDGIVFDEFNPLKLPRE--TMIMLTNVEF--HSTVSVKHGSVTIPRLLPRVFLTNVK----IFN----DPAIVRRVKTIP
VN-----KDVI
>PpulchraPlasmids_OMJ21113
ASMSFFLTYPKCAL-SKETVLAELKKICDELHAD--GTPHIHAYLKVKKTINSKN-----PRYFDIMG--YHGNYESCRY
PKNVM------------KYVMKDKNYIVE--------------EAIAVIRY-----NLARDYLRNPIGYEQGLAHILHPP
KP-------NPAHRFRDVPELL------NWDRQDKTLWLYGVTGTGKTSFAKTLYTMPL-----FVTHLEDLKKIKP--Y
HNGIIFDDMNFVTFSRE--EMIKLTDLDD--DRSFSVKYTSKTIPAKMSRVFCSNVS----IFD----DKAIRRRLKIVL
V------TDLR

>Smaco_ADB24799
QR--THTNA---ERWFKIFRDDIHKWVIGLEEG-KGGYGHWQVRCNI---EYLRA----VFSWLGPIS-------IWTEE
CSDKY------------TYETKEGRYWAS--------WDTMGARQQRG------KMRNQ------------------GAV
QALQRTNDRE------------------------IVVWYD-EQNMGKSWLCGHLFETY-------------MSTVASLVL
QRPLIVIDIPRS--------LYTAIEAIK-DGLIMDPRYGARPVNIH-GIKVIVLTNT---KPKLD-----------KLE
-DRWVLYD---
>Smaco_AEW47007
SRTSIPEHH----LVRLLK-LDLHEAYIGRETG-ARGFEHYQCCIDC---LVRFN----TEHQLGWHI-------EECS-
WEAS-------------NYCRKTDNYRYV--------GDSIEEREYS-IATRN----VA------------------RIQ
FHIDHRNDRA------------------------ISICVDTIG-TGKSTYGYLCARRTA------T-------YIAMHYD
NQPVIWIDLPRS--------LAECLEDIK-DGLVASAKYEGCLRFIR-GVKVLVTTNH---KTTYK-----------ML-
ADRWDIFT---
>Smaco_AIY31243
WKHLTWTME---QLA-EWFKQHAKEGVIGLEISPTSGKEHYQFKIHLDRGETLEG----WKALIGPMG------HIEIAV
DKNF-------------GYEEKDGNFIKWPTS----PLEKYK-LKLR---------WQ-------------------DVV
ETFERQDDRR------------------------ILVVVDKQG-NGKSYLSRFMEATVI------S--------EYCLDN
PC--YVFDLPR--------AMWMGIEQIK-NGLLYEKRYRPRKMWIE-PPKVLVFTND---DVPWD-----------ML-
RDRWEAYE---
>Smaco_AIY31246
PRKGSGTGK---EKWRTFFYIDVHKWTLGAETG-KGGYKHWQVRLQISDNEVFNK----IKDEFPTAH------IEKGS-
--DTW------------SYECKEKMHWTS--------NDTPNILRVRG------HMRYQ------------------DVI
EAVRRQNDRQ------------------------IAYWYD-PENKGKSWLVNHLFEST-------------LSWVASVYI
NREILVIDIPRS--------LYTAIETIK-DGLVYDPRYHAQMINIR-GVKILVLCNH---EPKLD-----------ALA
-DRWYAVA---
>Smaco_AIY31250
PR------TIHKRIIRELFDQDVKEYIIAKETG-KGGYEHWQIRCKASRPDFFEY----VHDREPRFN---------IQK
ATEDF------------DYERKDGHFWCS--------EDNNEILKCRG-----ELGMWQ------------------QLM
KRLKKQSVRE------------------------IDVVLD-PANKGKSFATIALWERA-------------QTFVCSAWK
GEWLIIIDIPRS--------LYETMEEIK-DGLVFDWRYSGKTRNIR-GTKLVVFTNE---PLNLK-----------GLS
-DRWKLHG---
>Smaco_AIY31256
WKDKYGSKE---QLA-EWFRLHAKEGVIGEEISPTSGKIHYQFRIHLDRGETLKG----WQELIKAVG------HVEVCQ
EKKF-------------DYEKKDGNWIEWPES----YLGKFK-MPLL---------WQ-------------------QLL
EQWNEQDDRQ------------------------ILFVKDVKG-NGKSTFGKIMEARVC------S--------DFCLEY
PA--YIFDIPR--------ALWSGIEQIK-NGLLYEKRYKPRKVWIE-PPKVIVFTND---EPPWD-----------LL-
LDRWRVFD---
>Smaco_AJD07511
SREETWHAP---DVGKQLMEHGAERCVVGEEVG-EDGYQHFQIRVVFKKPTSFEK----ATAILPGHW------SETSQF
GRNF-------------DYVEKEGHFWRS-------WETALGRFMLDM-------YWQ-------------------EIM
TRLEKQNDRK------------------------VMVIVDRYN-SGKTAIAMRLTAELD------W---------ALAHK
--NCFCLDIPR--------AIWKAVEQMK-NGYLWDKRHHWQEAFIM-PSKGLVLTND----EPDR-----------NLL
RDRWDIGH---
>Smaco_AJE25845
SKAK--CPE---EILRKWLDENGERYAYGRERG-EDGYEHFQVRVVLRNPTSWET----MREIWGNSG------HCSPTS
IRNF-------------DYVLKEGDFVCS-------WIKVPDAIRAQL-------RWQ-------------------HLV
NLK--QNDRE------------------------VDCIIDLRN-TGKSFVTKYMCMKGT------M---------CMKR-
-AKTYIFDIPR--------ATWSAIESIK-NGYMWDDRYTWEEMWID-SPRVFVFTNE----YPKY-----------DLL
EDRFRFWA---
>Smaco_AJE25847

SADK--VPE---KTMVEWLNKYCERYAYGRETG-ENGYKHYQIRLVLKVGADISE----MRKVWSAFG------HVSCTS
VRNF-------------DYVLKEGDYVCS-------WIKIPESMVPEF-------RWQ-------------------ALL
DLE--QDDRQ------------------------IDVIIDLQN-SGKSWLTKYCALKGE------M---------CLKRG
-AQLYIFDMPR--------AVWSAIESIK-NGYLWDDRYTWEEKWID-PPRIWVFTNE----YPKY-----------DLV
EDRLRFWA---
>Smaco_AJE25851
SADK--VPE---KTMVEWLEKYCERYAYGRETG-ENGYKHYQVRLVLKVGADISE----MRKVWSAFG------HVSCTS
VRNF-------------DYVLKEGDFVCS-------WIKVPDAIRAQL-------KWQ-------------------HLV
DLK--QNDRE------------------------VDCIIDVRN-TGKSFVTKYMCMKGT------M---------CMKR-
-AKTYIFDIPR--------ATWSAIESIK-NGYMWDDRYTWEEMWID-SPRVFVFTNE----YPKY-----------DLL
EDRFRFWA---
>Smaco_AJF23060
SKEK--LPR---SILEKWLNERCERWAYGNEVG-EGGYEHYQIRVVLKEPTDEYE----MRKMWAAFG------HVSPTH
VRNF-------------DYVLKEGDYVCS-------WIKIPENMQATF-------RWQ-------------------ALL
DLE--QDDRQ------------------------IDVIIDLAN-TGKSWLTKYCALKGE------M---------CLKRG
-AQLYIFDMPR--------AVWSAIESIK-NGYLWDDRYTWEEKWID-PPRIWVFTNE----YPKY-----------DLV
EDRLRFWA---
>Smaco_AJF23062
SKEK--LPR---SILEKWLNERCERWAYGNEVG-EGGYEHYQIRIVLKEPTDEYE----MRKMWAAFG------HVSPTH
VRNF-------------DYVLKDGDFVCS-------WIKYPESMVPTF-------RWQ-------------------CLL
ELE--QSDRE------------------------IDVIIDLKN-TGKSWITKYLALKGE------M---------CLKR-
-PTWYIFDMPR--------ATWSAIESIK-NGYLWDDRYTWEEKWIE-PPKVTVFTNE----YPKY-----------DLV
EDRLRMWA---
>Smaco_AMR73071
G---RTGYS---GEIMAIIREDIHKWIIGAEVG-AGGYQHWQCRFKTSPEEMTEK----LRARIGGPS-------IYTAE
CSDNW------------EYEAKEGAYLAS--------WDTMKVRSTRG------KPSQQ------------------VAL
QALRDTNDRQ-------------------------VVVWYD-PQNIGKSWLTNHLYEQT-------------MNTVASLAV
KRPYVIIDIPRS--------LYTAIESIK-DGLIMDPRYSAQPINIR-GVKVMVMTNT---MPKID-----------KLS
-DRWVIHT---
>Smaco_AMR73073
PR--THVSK---RAIRIMIDTDCKKWIVAKERG-KNGYEHWQIRIESSNTGFFEW----CKLYIPTAH------VEKAER
GVDES------------RYETKEGQYVLY--------TDRVEILKQRG------KMRNQ------------------RAL
EALESTNDRE-------------------------VLVWYD-EGNVGKSWLTGALWERT-------------VSWVASCYQ
SRPYVIIDIPRS--------LYVAIESIK-DGLVYDTRYHAQCINIR-GVKVLVLTNT---QPKLD-----------KLQ
-DRWRICV---
>Smaco_GQ351275
QR--THTNA---ERWFKIFRDDIHKWVIGLEEG-KGEYGHWQVRCNV---EYLRA----VFGWLGPIS-------IWTEE
CSDKY------------TYETKEGKYWAS--------WDTMGARQQRG------KMRNQ------------------GAV
QALQRTNDRE-------------------------IVVWYD-EGNMGKSWLCGHLFETY-------------MSTVASLVL
QRPLIVIDIPRS--------LYTAIEAIK-GGLIMDPRYGARPVNIH-GTKVIVLTNT---KPKLD-----------KLE
-DRWVLYD---
>Smaco_KJ547633
DNFEGVKDE---SEWRSRFAGLFERYAYGRETAPETGRRHFQFRGVLKVACDASC----LAYLSSLGF------NISPTH
VRDF-------------DYVYKDRDFFCS-WD----VYRPEYDVRNSH-------VWQ-------------------QLE
DME--RDDRT------------------------IEIVWDERN-SGKTAWAMYQDYLPR------C---------VLGKR
--EWYIIDTPR--------ADWASIEQLK-NGYVFDTRYSFRDRYLS-RPRVTVLCNT----LPEY-----------EYF
ADRVLPFR---
>Smaco_KJ577810
PR---TVSK---RALRIMIEKDCKKWVIGKEEG-KNGYKHWQIRIETSNDDFFEW----MQDHIPTAH------VEKSEN

```
GVDAC------------RYETKEGQYVMY--------SDRVQNLMQRG------AFRNQ------------------RAM
QALQATNDRQ-----------------------VVVWYD-ETNVGKSWFTGALWERT-------------ITWVASCYI
DRPYVIIDVPRS--------LYSAIESIK-DGLIYDTRYHSRMINIR-GVKVLVMTNT---MPKLD-----------KLK
-DRWCICT---
>Smaco_KJ577813
PR--NNTAK---EAISKWLRADVHKWICAMETG-ADGYDHWQIRLQVN--KTWEK----LKEEWGPKA--------HIEE
ASDVW------------DYERKSGLFFSS--------QDTPEVRKCRG------HLTRQ------------------AVL
RAVQSTNDRQ-----------------------VVVWYD-PDNKGKSWLLGHLYET--------------QTDVASEYI
NRPMVVIDIPRT--------LYVAIERIK-DGLIKDPRYSSKTVHIR-GVKILVTCNT---MPKLD-----------KLE
-DRWIIID---
>Smaco_KM573771
PTK-----HACKRQVKMILEIDTKRYIFAQEKG-KNGLDHWQLRIQIKEENFERL----QKLFCNKAH---------IEV
ANDSW------------EYERKEGKFWSS--------EDTKEILKIRG-----KL-REQ------------------KIL
QILSSQGDRE-----------------------IDVWLD-PTCHGKSWLTIHLWETS-------------STFVCSSWR
GEDIIVIDVPRA--------LLETMEELK-DGLVFDHRYTGKTRNVR-GTKLMVFTNS---PLPLN-----------KLT
-DRWRLHG---
>Smaco_KM573775
P---------AKRHWLKLFEVNVKKHVVGLEEG-KNGYKHWQARIQLSEISFFQY----MKIYYPKAH---------IEE
ASNTW------------EYERKEGKFWTS--------EDTASILAVRG-----SL-REQ------------------KIL
QILESQGDRE-----------------------IDVWLD-PSNHGKSWLTVHLWETS-------------STFICSSWK
GEPIVIIDIPRS--------LLETMEELK-DGLVFDHRYTGRTRNVR-GVKVMVFTNS---ELPLK-----------KLK
-DRWRLHG---
>Smaco_KM598409
SAEDWNEDG----IIRVFE-NDGHELYIGREIG-KHGFRHYQFCMDC---LEKYT----ADNRTGWHV-------ERCS-
WEMS-------------GYCRKTGDYRYI--------GDSREERYYA-LRARL----IW------------------SFG
ASVVKQNDRS-----------------------ITVWVDTEK-AGKSTFSYILERRTE------N-------FVAMHYK
GEPLIIVDIPRD--------LCRALETIK-DGVITSAKYQGTKMFIK-GVKILVFTNH---KTTYA-----------AL-
EDRWDVKS---
>Smaco_KP233175
SADK--VPE---KTMVEWLNKYCERYAYGRETG-ENGYKHYQIRLVLKVGADISE----MRKVWSAFG------HVSCTS
VRNF-------------DYVLKEGDYVCS-------WIKIPENMKPEF-------RWQ-------------------ALL
DLE--QDDRQ-----------------------IDVIIDLQN-SGKSWLTKYCALKGE------M---------CLKRG
-AQLYIFDMPR--------AVWSAIESIK-NGYLWDDRYTWEEKWID-PPRIWVFTNE----YPKY-----------DLV
EDRLRFWA---
>Smaco_KP233189
PR--DKVSK---RELRIMLDKDCKKWIIGKETG-KNGYKHWQIRLETSNEEFFDW----CKKHIPTAS------IRKAE-
-VPKW------------DYEAKEGQYWTS--------SDRTDNLIQRG------EFRNQ------------------RAI
QALRATNDRE-----------------------VLVWYD-EGNVGKSWFTGALWERT-------------VTFVAS---
--PYVFIDIPRS--------LYCAIESIK-DGLVYDSRYQGRMVNIR-GVKIIVMTNN---KPDLD-----------KLY
-DRWRMVV---
>Smaco_KT862218
PR------TIHKRIIREIFKKDVKKYIIAMETG-NGGYEHWQIRCTASRPDFFEY----VHDREPRFN---------IQK
ATESM------------EYERKEGRFWSS--------EDTTEIRQCRG-----ELGRWQ------------------QIL
KVLKNQDVRT-----------------------IDVVLD-PVARGKSHFTIALWERY-------------STFVCSAYK
GEKIIIIDIPRA--------LYESMEEMK-DGLVFDPRYSGKTRNIR-GTKVLVFTNN---PLDLK-----------KLH
-DRWNLHG---
>Smaco_KT862221
PRR----IICSKKLLSYIFEQDVKRYIVAIEKG-KNGLDHFQIRLSCSDPDFFEH----MKDWCEWAH---------VEK
ATDNF------------DYERKEGRFWTS--------DDTTEIRICRR-----ELGRWQ------------------QIM
```

QVLKKQDVRT-----------------------IDVVLD-PVARGKSHFAIALWERY------------STFVCSAYR
GRKIIIIDIPRA--------LYETMEEMK-DGLVFDPRYSGKTRNIR-GTKVLVFTNN---PLDLK-----------KLH
-DRWNLHG---
>Smaco_KT862224
----HGTTQ---RL---AYQ-HAKEGVIGEEISPTSGKTHYQCKWHLSRGESIDG----WKLLIGPMG------HVDIAV
EKRF-------------GYEEKDGKFVKWPES----PIAKHK-LALK---------WE------------------ALL
DSIKNQDDRH-----------------------ITVVVDKQG-NGKSTFSKYLEANVV------S--------EYCMEF
PK--YVFDLPR--------AMWSGIEQIK-NGLLYEKRYKPRKMWIE-PPSILVFTND---DIPWE-----------LL-
EDRWDAYR---
>Smaco_KU043403
ER--RETYK---EWFLFAIRKDIHKWIIAAEEG-KGGYKHWQIRIAS-DAKWTRA----LLIQFGQRS-------IHTEN
ASNTW------------TYEAKEGCYLAS--------WDTLEVRQQRG------KLRVQ-----------------YAL
NVLEGTNDRQ-----------------------VMVWVD-EENSGKSWLIGHLYETY------------LSTIASLAV
KKKYALIDIPRS--------LYTAIEAIK-DGLIMEPRYSAQPINIK-GIKVLVVTNT---RPKLD-----------KLK
-DRWEIFE---
>Smaco_KU043420
TR--KSVLIKRCRIKSSFKNLDCKRWTLGIETG-KGGYKHCQWRVECGDDQFFEH----FSEFTGWGIE----KKSHIEK
-SDKW------------DYETKEGHYVKS--------DDRVENIIQRG------KYRAQ-----------------RVI
KALRRTNDRE-----------------------VVLWYD-KVNCGKSWFTGALWERE-------------KDIASDFL
DRPFVIIDLPRT--------LYLAIERIK-DGLIKDPRYNSRTVNIR-GVKVLVCCNS---LPKLD-----------KLK
-DRWVRLE---
>Smaco_KU043422
PKN---ARN----LIKNLIETDLHKWTYGYEIG-KNGYKHIQARIRCD----FDY----LQEYFGQAH---------IEE
ASDEW------------DYETKGGIYFTS--------EDWGERLKQRL-----PLKVQK-----------------AL
EGLEATNDRE-----------------------VYVWYD-EKNAGKSWLCGHLWETKD------V-ANEYIN-------
-RPYVIIDLPRT--------LYYAIEKIK-DGLLKDPRYQSKTVNIH-GVKVMVMCNH---RPNVS-----------KLA
-DRWKMFT---
>Smaco_KU043428
PR--RKTAR---EGISKWLRDDVHKWTVAMETG-DNGYDHWQIRLQVN--KTFKQ----LKKEWGPKA--------HIEE
ASDTW------------NYERKSGLFFSS--------NDTPEVRKCRG------RLTRQ-----------------AVV
QAVQGSNDRQ-----------------------IVVWYD-PNNAGKSWLLGHLYET-------------QTDCASEYI
QRPFVVIDIPRS--------LYVALERIK-DGLIKDPRYGSKTVHIR-GVKVLVTCNT---KPKLD-----------KLA
-DRWVIMN---
>Smaco_KU043430
PR--NDTYR---EKIFAWFRKDIHKWVLGAEKG-SGGYEHWQMRVQCR--FGFEE----LKVLF-PTA--------HIEE
CSDKW------------TYEAKEGVYWRS--------NDRRENIQQRG------KLRAQ-----------------RVI
ERADGTNDRE-----------------------VVVWYD-QEKAGKSWLTGAMWER-------------DSDIASEYL
KRPYIIIDIPRA--------LYEAIERIK-DGLIKDPRYSSEAVNIH-GVKVIVMTNT---MPKLD-----------KLA
-DRWKII----
>Smaco_KU058671
PR--NDTYK---ESIWMWFVKDVKKWVFGLEKG-RFGYKHWQIRFKSN--LKFED----LKVMF-PTG--------HIEE
ASDTW------------TYETKEGVFWKS--------TDRPENRAQRG------KLNDQ-----------------GVI
RALQSTNDRE-----------------------VVLWYD-SEKKGKSWLTRALWER-------------DNDVACEFI
KRPYVIIDIPRA--------LYEAIERIK-DGLIKDPRYSSESINIS-GVKVLVNSNS---LPELD-----------NLK
-DRWKIYP---
>Smaco_KU203352
DANENVMKL---EDWKNGLSGYFERYAFGCEVAPETQRRHYQFRGVLKADLSNDI----ALALSDLGL------HITPTH
VRDF-------------EYVYKDRNFYCS-WE----VFRPEYEVQNSH-------VWQ------------------QLE
ELD--RDERS-----------------------IEIIWDEKN-SGKTAWAMYQDYSLR------C---------VLGKR

--EWYIIDTPR--------ADWASIEQLK-NGYVFDTRYSFRDKYLS-RPRVTILCNH----MPDY-----------EYF
ADRVLPFR---
>Smaco_KX838317
PRSESRISK---KAIMIMLSKDCKKWTIGQEVG-KNGYKHWQIRVESSNTNFFKW----CKQHIPSAH------VEKAEK
GVDES------------RYETKEGQYTQY--------TDRVEILKQRG------KMRNQ------------------RAL
EALEATNDRE------------------------VLVWYD-ESNVGKSWLTGALWERT-------------VSWVASCYQ
SRPYIIIDIPRS--------LYAAIESIK-DGLVYDTRYHAQCINIR-GVKVLVLTNT---KPKLD-----------KLA
-DRWRICV---
>Smaco_KX838318
PR--AHVSK---RAIRIMIDTDCKKWIIAKEKG-KNGYEHWQIRVESSNTGFFEW----CKQHIPTAH------VEKAER
GVDEC------------RYETKEGQYTCY--------SDRIPVLKQRG------KLRNQ------------------RAL
TALEATNDRE------------------------VLVWYD-EGNVGKSWLTGALWERT-------------ITWVASCYQ
DRPYLIIDIPRS--------LYVAIESIK-DGLVYDTRYHSRLINIR-GVKVLVLTNT---KPKLD-----------KLE
-DRWRICV---
>Smaco_KY086298
PR---KVHK---RTLKIMLEQDVKKYIIAKERG-FGGYEHWQIRLKTSNKNFFIW----CKINIPEAH------VEEAM-
--DTW------------DYERKEGVYWTS--------DDTNEIRALRG------KPNKQ------------------RVL
KLLKYQGDRN------------------------ILVWYD-PVKAGKSWIVGHLWEQT-------------LTWVHSAYD
NEGLIIIDIPRS--------LYTAIETIK-DGLVYDPRYSARMKNIR-GVKVLVMTNT---YPRVS-----------ALE
-DRWDIIN---
>Smaco_KY086301
SKAK--CPE---EILRKWLDENGERYAYGREKG-TDGYEHFQVRVVLKNPTSEQD----MWKVWAPYG------HVSPTH
TRNF-------------NYVLKEGDFVCS-------WIKIPESMKPVF-------RWQ-------------------ALM
DLE--QNDRE------------------------VDVIIDLKN-TGKSWITRYLALKGG------M---------CLKR-
-AVWYIFDMPR--------ALWSAIESIK-NGYLWDDRYTWEEKWID-PPKVTVFTNE----YPKY-----------DLI
EDRLRFWA---
>Smaco_QBP37051
PHSGITKRA----FFKMIRDYDIHKWTYAVERG-RGGYKHIQCRFRTNK--SFEE----IRKALICGH---------IEE
ASDNW------------EYEKKDGNYMTS--------EDNHEILKLRYG---KLTKVQE------------------WAL
VLLESTNDRE------------------------VVVWVDKDGNSGKTWLTAHLWERLS------T-PKELISWVHSAYN
HEPYIIIDIPRTWKWDD--ALYTAIETIK-DGLVYDPRYSAHMRNIR-GVKVMCMTNT---EPKLS-----------KLS
EDRWVMYR---
>Smaco_QBP37113
PHSGITKRA----FFKMIRDYDIHKWTYAVERG-RGGYKHIQCRFRTNK--SFEE----IRRALICGH---------IEE
ASDNW------------EYEKKDGNYMTS--------EDNHEILKLRYG---KLTKVQE------------------WAL
VLLESTNDRE------------------------VVVWVDKDGNSGKTWLTAHLWERLS------T-PKELISWVHSAYN
HEPYIIIDIPRTWKWDD--ALYTAIETIK-DGLVYDPRYSAHMRNIR-GVKVMCMTNT---EPKLS-----------KLS
EDRWVMYR---
>smaco_YP_009252318
LPDEHGTTQ---RL---AYQPHAKEGVIGEEISPTSGKTHYQCKWHLSRGESIDG----WKLLIGPMG------HVDIAV
EKRFT------------GYEEKDGKFVKWPES----PIAKHKNLALKT--------WEV------------------ALL
DSIKNQDDRH------------------------ITVVVDKQGGNGKSTFSKYLEANVV------SDEYN-DYTSYCMEF
PKKAYVFDLPRATSIKRRTAMWSGIEQIK-NGLLYEKRYKPRKMWIE-PPSILVFTND---DIPWE-----------LLS
EDRWDAYR---
>Smaco_YP_009022025
PR---SVPK---KALKIMIDVDCKKWIIGKERG-KNGYEHWQIRIETSNDEFFKW----CKHHIPAAH------IEEAQQ
GVDEC------------LYERKEGQFWTS--------SDRVETLHQRG------TLRRQ------------------RAL
LALQSTNDRE------------------------VMVWYD-ANNVGKSWFCGALWERT-------------VTWVASCYM
DRPYVIIDIPRS--------LYCAIESIK-DGLIYDTRYHARMINIR-GVKVLVLTNT---LPKLD-----------KLR

-DRWCIFE---
>Smaco_YP_009030025
PR---TVSK---RALRIMIEKDCKKWIIGKEKG-KNGYEHWQIRIETSNDNFFQW----IQDHIPTAH------VEKSDN
GVDEC------------RYETKEGQYVTY--------SDRVQNLIQRG------AFRNQ------------------RAI
QALEATNDRQ-----------------------VVVWYD-ETNVGKSWFTGALWERT-------------ITWVASCYI
DRPYVIIDVPRS--------LYSAIESIK-DGLIYDTRYHSRMINIR-GVKVLVMTNT---MPKLD-----------KLK
-DRWCICT---
>Smaco_YP_009054985
PR---TVSK---RALRIMIDVDCKKWIIGKEEG-KNGYKHWQIRIETSNDSFFEW----MQDHIPTAH------IERTEC
GVDAC------------RYEAKEGQYVMY--------SDRPQNLMQRG------EFRNQ------------------HAL
QALQQSNDRE-----------------------VVVWYD-ETNVGKSWFTGALWERT-------------ITWIASCYI
ERPYIIIDIPRS--------LYSAIESIK-DGLIYDTRYHSSMMNIR-GVKVLVMTNT---MPKLD-----------KLK
-DRWCIRT---
>Smaco_YP_009054987
PR--NNTAK---EAISKWLRADVHKWICAMETG-ADGYDHWQIRLQVN--KTWEK----LKEEWGPKA--------HIEE
ASDVW------------DYERKSGLFFSS--------QDTPEVRKCRG------HLTRQ------------------AVL
RAVQSTNDRQ-----------------------VVVWYD-PENKGKSWLLGHLYET--------------QTDVASEYI
NRPMVVIDIPRT--------LYVAIERIK-DGLIKDPRYSSKTVHIR-GVKILVTCNT---MPKLD-----------KLE
-DRWIIID---
>Smaco_YP_009054993
PR--ERARIGTEPRLMRYIREDVKKWIVAMETG-RRGYEHWQVRLQAVTE----K----QKVGVGWIIP-----QAHVEE
CSDNW------------EYEAKEGRYWAS--------WDTVEVHRMRG------RPRYQ------------------ALL
NKLRTTTDRE-----------------------VMVWYD-PENNGKSWLVGHLFETT-------------LSTMASLAH
QKPYVIIDIPRT--------LYCAIESIK-DGLIMDPRYSARPINIR-GVKVLVLTNE---MPKLD-----------ALE
-DRWIIEN---
>Smaco_YP_009118276
PR--RKTEK---EGIYKWLRDDVHKWTVAMETG-NNGYDHWQIRFQVG--KTFKQ----LKKEWGPKA--------HIEE
ASDTW------------EYERKSGMFFSS--------DDTPEVRKCRG------RLNRQ------------------TIV
RAVQDTNDRE-----------------------IVVWYD-PNNKGKSWLLGHLYET--------------QTDCASEFI
NRPIVVIDIPRT--------LYVAIERIK-DGLIKDPRYNSKTVHIR-GVKVLITCNT---MPTRD-----------KLA
-DRWVIVE---
>Smaco_YP_009118278
PR---KGYS---GAIMRFFRKDVHKWIVSPEKG-AQGYEHWQIRFRCTPENAMIA----WRQWVCNGFN-------MLEA
SDNGW------------EYEGKEGKFLAS--------WDSKGARSVRG------KMERQ------------------ATV
LRARATNDRE-----------------------IVVWYD-PKNSGKSWLVGHLVETY-------------LTTLASMVK
ARPLVVIDIPRS--------MYVAIEAIK-DGIIVDPRYSATVENVK-GIGVIVITNE---KPQVG-----------KLA
-DRWDIVD---
>Smaco_YP_009163761
PR--DEEMY----RIWRYINSDVHKWIIASEIG-RNGYKHWQIRIKTSDPDEYRK----VKIGTGWAIP-----RSHVEE
CSDDW------------DYETKEGRYLAS--------WDTPEVRKLRG------QPRHQ------------------AII
NRLESTNDRE-----------------------VMVWYD-PTNSGKSWLVGHLYETT-------------MSMMASLAI
QRRYVVIDIPRT--------LYCAIETIK-DGLIVDPRYSARPINIR-GVKVLVLSND---RPSLD-----------KLV
-DRWVVNT---
>Smaco_YP_009252308
PW--TGQSK---RMINLYITRDIHEWIIGYEVG-RDGYRHIHVRFNGD----FGD----VQRAFPGAH--------IEEG
TTMET------------EYEKKDGHFVSY--------EDSPDVLRCRG------KLRHQ------------------RVV
KLLEKQSDRG-----------------------ILCWYD-ETSIGKSFTCRHLVERT-------------VNWVCSGYQ
RQRYLIIDIPRS--------LYTGLEAIK-DGLIYDTRYSAKLRDIW-GVKILVLTNS---LPNLD-----------ALQ
-DRWMIIN---

>Smaco_YP_009252310
PR--------EADLRPICRMLDAKKWTIGFEVG-EHGFRHYQIRLVSSDHDFFEW----CKAFLPTAH---------IEE
ATEER------------DYERKSGNFLCS--------DDTDQIRQIRG-----DL-RIQ-----------------KIL
KLADDQNDRQ-----------------------ISYFYD-PDGAGKSWLTIHLWERS-------------KTFICSGYK
AEEYIVIDLPRA--------LYELLEDTK-NRLIFYSRYQPITRNIR-CPNLIVFSNH---KLDTK-----------RLA
-DRCQYYD---
>Smaco_YP_009252314
SADLWKERD----VVRLLD-MDLREYYIGREIG-KGGYHHYQCAIDC---LERFN----GQHQLGWHI-------EDCS-
WDKL-------------RYCRKGGDYRYI--------GDSIEEQSYR---SRT----VG-----------------TID
THLKKQNDRQ-----------------------ISICVDTKG-SGKTTHGYDRSRTNA------T-------YVAMNYD
NEPVIWIDLPRT--------LATILEDMK-DGLIYSAKYEGQVRHIK-GVKVLVTTNH---KPAYK-----------LL-
ADRWDVFT---
>Smaco_YP_009252316
FFPE--FLE---KTLQEALDKICERYAYGNEVG-KDGYEHFQCRIVCSKPTDERA----LRVFLLSNG------HTSPTQ
VRNF-------------EYVQKEGNLYCS-------WEAVLKQFPGTP-------YWQ------------------IAF
SMWKKQNDRE-----------------------ILVITDDKR-HGKSWLRKYMVACLK------C---------AMAKP
---SYIIDMPR--------AMWSAIEQMK-DGYLYDKRYSWQEKWIE-PPKIMVFCND----FDP------------LML
TDRWQSFD---
>Smaco_YP_009252320
PR--RTVCSK--KLLNYILEQDTKRYIIGIEKG-KNGLEHFQIRLSCSDPEFFEH----MKEWYPYAHI----EKSDVGI
NSESM------------EYERKEGRYWTS--------MDTTEIRIQRG------KPNTQ-----------------RVL
EVLRRTNDRE-----------------------IVLWYS-DKSIGKSWLVGHLWETQ--------------TDVASEYI
KRPCIVVDLPRT--------LYCALESIK-DGLLKDTRYSSDTINIK-GVKVLVTSNT---LPKFD-----------SLF
-DRWIVIE---
>Smaco_YP_009252326
PQAV--LSA---ENVETILEQECERYAYADEVG-EGGYKHWQIRYVLRKGSPVEE----QIMIWSMWK------HVSPTH
VRNF-------------NYIMKTDNYFCS-------WESELREYRLEY-------RWQ------------------YTL
EMIEQQNDRE-----------------------IFVIQDPLG-IGKTTFAKHLVANLE------M---------AMAKA
RRETFIIDVPR--------AMWSAVEQIK-NGFLYDKRYQWSEKWIK-SPKIVVLTNE----LPK------------DKL
NDRWNVYQ---
>Smaco_YP_009508826
SRTSIPEHH----LVRLLKLLDLHEAYIGRETG-ARGFEHYQCCIDCAGDLVRFN----TEHQLGWHI-------EECVS
WEASK------------NYCRKTDNYRYV--------GDSIEEREYSRIATRPKNIVAD-----------------RIQ
FHIDHRNDRA-----------------------ISICVDTIGGTGKSTYGYLCARRTA------ETPTRIMDYIAMHYD
NQPVIWIDLPRSRLVDK--DLAECLEDIK-DGLVASAKYEGCLRFIR-GVKVLVTTNHWVDKTTYK-----------MLS
ADRWDIFT---
>UJSL001_MN621482
QRTVSPSGSRSVSPPSRPYNTKEGETALAPIVAEEPEKTRWQGRIFLITLNQEEK----
WPRLREYLHTRPVEYMYAAHE
VAPTTN---------ALEYCKKQG---------------MLVEEYGECPIRSANKKGISIKDVMEMDQTQILELRASSFN
YVRNIRSELLNTQIRNAKHYAP-----------IDFEWYYGPTGTGKSRKAFEEGATP----------IQYANGFFTDWA
GSKVLVYEEFRGQVPYH--LILQLTDAYH--GYYCLNIKGGFRVLDIDKLIVTSPLRPEECYPRQCQK-----RDSIQQL
VRRITKMLHFT
>UJSL002_MN621468
KDRYKGTKE---ELKAWLIAHHAKEGVIGEEISPTSGKMHYQCKIHLSRGETLEG----WKALIGPFG------HVDIAH
DKNFS------------GYEEKDGNFIRWPES----PIEKFKDIGPLY-------FWEQ-----------------VVL
AELEKQDDRK-----------------------MLVVLDTTGKGGKTTFSKHLEAKVV------SDEYN-DYTGYCMEF
PAKGYIFDIPRASSIKRRCAMWSGIEKIK-DGLLYEKRYKPRKKWIE-PPKVLIFTNEP--DIPYE-----------MLS
RDRWVVVD---

>UJSL003_MN621469
KNWCWTLNNYTEEEVTR---LQALAYD--EFETGEQGTEHLQGFTMFTRRLRLTQ----VKDLLG--HR---IHAEPAHG
-TPKQ----------AADYCKKDGNTDNT----------GKNQ--WDAIRRLELAGNL---EAIKEQFVGTYHKYRKVIE
YECALHTVCHTVDGDLQ----------------NKNKWIWGAPGLGKSRFVRE---------IYTKMSNKWWDGYEGE--
-PAVIVEDLDPTRAEMLTQQLKIWADRYP---FTAEIKGGARKMEPSYRLYITSNYPPEACFK---------NDVDLQAI
RRRFQVIHYD-
>UJSL004_MN621481
SRESIKEHQ----LVRLLNLLDLHEAYIGRETG-NRGFQHYQCCIDCAGDLERFN----REHNLGWHV-------ETCIS
WECSK------------NYCRKTGNYRYV--------GDSIEEREFNRISRRPQNIVGK------------------RIC
EHLRSQGNRK------------------------ISICVDTDGGTGKSTNGYIHVRNTA------ETPVRIMDYIAMKYN
NEPVIWIDLPRTRSVDN--DLAECLEDIK-DGLIASAKYEGNLKLIR-GVKVLVTTNHWIKKETYK-----------LLS
QDRWDVFT---
>UJSL005_MN604398
YNTSQATVEEWFAKNKPKIQSMAACLDVGGKFLKDSFGPECHMEVAKRYLQAYIY---
VLGEGEHAANKKVFIEYRTPKA
GRSTT------------KKVQSDVFIRETLSR--------NMVRPIPRMVVWINGHTGTGKSLLANKFMDLHQPSSAYMC
SPSGQVSGPDNDPRSVLLDDFN-------IALTPLTTLFNMTDQYNKTIDCKGSYMWFIYSPYNVVGMEQTYTTPHRKIT
DHDQDQLSRRINLYIKTASPVPNNVDGYR--
VMHPDGTPDELIPKMNLDEMIAYIDSIYKSWEQKYYEEQLNNEENYHEV
ENNNINTA---
>UJSL006_MN621480
AVKTWFCTWPKCTIPKEFALKLLEKHG--LKEKHEDGSPHLHAYLKLIKRKKFNEK---
MFDLIDVDKKVFHGNYQKVKD
VLAVE------------KYVTKG-----------------QHKSKLLKEDYLKDPLELLEEGKINFFQINNFLKNQDCYK
MLLNRKQNRPLKPLEK-----------------RRHHWIFGESNTGKTERLWKAMDTEG--GWFQIPTNNDWKGYNGE--
--INLYMDEYKGQLTIQ--ELNRICDGGA----KVNTKGGSTQLSWEVTVWICSNYSIDNCYNKAEK-------MLLMSL
WNRFNEEEML-
>UJSL007_MN621470
PHSGITKRA----FFQMIRDFDIHKWTYAVERG-RGGYKHIQCRFRTNK--SFDE----IRHALICGH---------IEE
ASDTW------------EYEKKDGNYMTS--------EDNHEILKLRYG---KPTKVQE------------------WAL
RLLESTNDRE------------------------VVVWVDKEGNSGKTWLTAHLWERLS------T-PKELISWVHSAYN
HEPYIIIDIPRTWKWDD--ALYTAIETIK-DGLVYDPRYSAHMRNIR-GVKVMCMTNT---EPKLS-----------KLS
EDRWVMYR---
>UJSL017_MN621476
----FRAVCFTSFVPEAMWDRNWEQLLGHKEICPETGRLHIQGYVEFSGQKKMKQ----IKLIFGD-PA---IHLEPRRG
-TQEE----------AIIYCKKEGQQ-------------QGQRTDLEDTYERIKVGQS--LLDIAEAHPGTYIRYFKGIE
RLRDLMQQRAQKLEAR--V-------------PPEVIVYIGKSGVGKSHACYNDPD-----YPVQQTGKVYFDGYQGE--
-RVIWFDEFGGSVLPFH--VFLRLADKWE---TRVETKGSSICILGLKKILISTTTYPKDWWPNSEK-----FREDPNQL
WRRLTRVYYI-
