# Supplementary material for: Viral metagenomics revealed diverse CRESS-DNA virus genomes in faeces of forest musk deer
Source: Virol J. 2020 Apr 25;17:61. doi: 10.1186/s12985-020-01332-y (PMC7183601; doi:10.1186/s12985-020-01332-y)
Supplement: Supplementary file 3 — Additional file 3. [file 12985_2020_1332_MOESM3_ESM.pdf]

>CRESS_AXH77830
MSKSWCFTINNPTGNDDWELEALKT---ETGEQGTRHYQG---YVVMNGQRTLRVMKTLL-TRAH----
LEARRGTHQQA
ADYCKKDGQMSAADKNKCM---WRNMIEWAEKGELDKIKEEYP-SFYLRY---LEKFRSLSRPTLSILPE--
LENEWWWG
ATGTGKSRKLWA---HYAKQLNKWWDGYDGEDVVAIEEWAP-KNECTASLLKIWADRYP--
FPAEVKGGKLNRIRPRKLI
VLSNYSPEQCFGQEED-------LGPIRRRFRVIHFE
>CRESSV2_FJ959082
RSRNWCFTYNNYDSSPQKCMK-----YEVG-ESGTPHLQG---FVIFKN--AVAKPSQYF-KPAY---HFEKARGTPQQA
LEYCQKDGNKTVADGGEANKRRYEEAFSAAKEGRMDDIPAD----IYIRHYSTLKKIRFDHAPPAQNNDVL--
NNYWVYG
PSGTGKSKSVREF--LYVKNQNKWFDGYEGEDFVLIDDVHP--NWS-GKTLKIWSDHYP--FSPETKGGHIKMIRPEGII
VTSNYTIEEMYEAEED-------RQPIRRRFKVIKEY
>CRESSV2_JX904107
KSRHFAWTMNNPGPDTEAAVIA----REVG-ESGTPHLQG---MTSFEHARSLSAIIKLL-QKAHPGTHVEICR-DAYKS
MVYCQKDGDKKPADGGGMERDRWKRAYEMAAEGNLEDIDAD----IKLRFYGTLKKIKEDHQVTPTSLPTL--
DFHWYQG
SSGSGKSKFAHD---YYLKSPNKWWDQYEPGQTVIIDEWDP--NHKVLASLKKWADHHP--FAAEIKGGTR-
MLRPPKLI
ITSNYTIKECFPQEND-------HLPLLRRFTVKQFG
>CRESSV2_JX904562
KARSFSFTWNNYTEADIARLKG----KEVG-ESGTPHLQG---MINFKSPRSFASVMKEL-EGAH----VEKTI-SSYDS
MVYCKKDGDVSQKAKGDMEKARWKRTRELAAAGKIEEVDDD----IYVRFYGTLKRIKEDHQQVPPAQAEL--
NFHWFYG
ASGTGKSRAAYA---LYIKNSNKWWDGYVDQPCVLIEEWDP--NLAMMASMKKWADHHP--FSGEIKGGTK-
MLRPPKII
VCSNYTIQECFPNEQD-------WKPLERRFKVRKFG
>CRESSV2_KF738877
RSRGWVFTINNYNEWDFVNISKLEK---ERGEEGTPHLQG---FAYFKQRISFNGIRDIL-TRAH----VEIQRGFNAQA
IDYCKKEGERGAGQKDK-----WKDVLQLARQGKVQEIEERYP-AIFLRY---FQKLCGFYRPEHSIILEN-FTNEWWWG
PTGTGKFKKLND---PYEKSLDPWWDNYQREEIVAIEEFEP-RCKIN-SFLKRWADRYP--FRCEVKGAFLSKLRPLKII
VISNYQLDECFPNSKD-------LDPLKRRFKEIHFP
>CRESSV2_KP153377
GNRAWCYTLNNYTEEERDSLRSYER-----GAADTPHLQG---YVQFAHQKTLSAVKKLL-PRAH----LEERRGTIDQA
VEYCKKDGDMSQKEKGKEEKNRWKRILEKADEGDEEWLRENEP-NVAFKH---
MATFRSHKKPRVGTLQYEETPHEWWVG
PTGTGKSRKAHE---HYAKEKNKWWCGYTGQETVIIEEADP--KTMELAALKVWADRYP--
FPGEIKGGRIEGIRPLRVI
VISNYTIEECFANQND-------VEPLRRRFKEVKFG
>UJSL001_MN621482
VSPSGSRSVSPPSRPYNTKEGETALAPIVAEEPEKTRWQGRIFLITLNQEEKWPRLREYLHTRPVEYMYAAHEVAPTT
NA
LEYCKKQGMLVEEYGECPIRSANKKGISIKDVMEMDQTQILELRASSFNYVRNIRSELLNTQIRNAKHYAP-
IDFEWYYG
PTGTGKSRKAFEEGATPIQYANGFFTDWAGSKVLVYEEFRG---QVPYHLILQLTDAYHGYYCLNIKGGFR-
VLDIDKLI
VTSPLRPEECYPRQCQKRDS--IQQLVRRITKMLHFT
>UJSL003_MN621469

LHKNWCWTLNNYTEEEVTRLQALAYDEFETGEQGTEHLQG---FTMFTRRLRLTQVKDLLGHRIH----
AEPAHGTPKQA
ADYCKKDGNTDNTGKNQ-----WDAIRRLELAGNLEAIKEQFV-
GTYHKYRKVIEYECALHTVCHTVDGDLQNKNKWIWG
APGLGKSRFVRE---IYTKMSNKWWDGYEGEPAVIVEDLDPTRAEMLTQQLKIWADRYP--
FTAEIKGGARKMEPSYRLY
ITSNYPPEACFKNDVD-------LQAIRRRFQVIHYD
>AUM61936
INMKQVFNFMNMIINQTNKVTKAHP--EVTPFYKYIMFGG----
IEFGKKTNRMHVHGYVYCTKQKSIKELKKHWGTEAH
FDVALMCDPIYEEGERPKQGNRSDIASAIEECETIGELMDSNP-ELYCKYRNGLKDIYARKEALKPKTYEQ-
PEIIWNYG
QTGLGKTRMAFED-EECVNYDNSFFSDWHESKVISLEEFNG---KIPYKTLLQLTDGYHNYYRINIKGGDK-
LVDLKRIY
ISSSVHPRDIYRQQDMKENEGGIDQLMRRITKINHF-
>CRESSV2_KP153404
RSRAFIFTWNNPTADTEAALES----RETAPTTGTRHLQG---YIRFTDGKSLRSARRLL-NGAH----VEVAR-TIRQA
IEYCHKEGDVDDAARGDMEKARWEIAWTKAKTADLEEIDAD----IRVRCYSALTRIQKDYMVPPLPLPAP--
CGLWIHG
LSGVGKTFAVYQ---LYSKNASKWWDGYQNQDHILFDDMDP--DVGKAGRFKIWADERP--FIADIKGGSI-SIRPKIFI
VTSQYTIDECFGEIQT-------RMALSRRFRIIEKL
>CRESSV2_KP153364
SARNFTFTQNNYGDTELDG-----VQKEVG-ESDLLQLQG---VICFNEKIRESAVRKKL-PGCH----IEIAK-VLPAA
IEYCKKDGDMTPKEKGALEKRKWDEIRVACEEGRFEDLPDD----IRYKNLRLNKMHRCEALRSRELESEA--
QHLWYWG
EAGTGKSRKARE---AYLKMCNKWWCGYTEEETVLIEDFDK--KHDVLGHLKIWGDRYP--FLAELKGDTM-
KIRPKQII
VTSNYHPSAIWFDEET-------LLPILRRFKCVEFK
>CRESSV2_KC248416
RNRNYVFTLNNYTPVHEITLNS----REVAPTTNTPHLQG---YICFPNAKTISAVRRIL-AGCH----VEVARGSHAQC
RTYCIKDGDADPREIGNAEADRWEDAWEKAKAGAIEEIPAD----IRIRSYSVLRRIGRDYQPNLALLPAT--CGYWIKG
ESGAGKSHSCFT---LYPKGPSKWWCGYQNEEVVLLDDVDP--SHGLIGGLKRWADKYP--FIGESKGGSF-KIRPKKFI
VTSQYSIEDCFQDVET-------RVALNRRFRVINKL
>CRESSV2_JX904420
QSRNFTFTLNNYTQEHLTTLEILVEQKEVG-ESGTPHLQG---FICFTTTKSMKQCIKAI-PGAH----VEFMKGTIEQN
VAYCSKDNDMTQKKKGEAEKERWEEALAAAKEGRFDDIPAD----IQFRYDRNIKRIYAENKPKPQTLNKL--
TNEWYCG
PSGTGKSKQARD---AYVKLNNKWWDGYADEETVIIDDFDK--YDIALSGLKRWSDHYP--FPAEFKGGVK-VIRPKRII
ITSNYTPEEIWEEEAT-------LGPIRRRFHITQFK
>CRESSV2_KT149398
RTRRIVFTYNNYPPEYNAWLDS----REVAPGTGTPHLQG---YAQYVNPKSITAFRRLF-MGCH----VEPARGTGSQS
RTYCSKDGSTD-ENPGNREKQRWEDARSLAKEGKFDQIPAD----IYIRYIGNLHRIYREILPPLEPLPAT--CGRWLLG
RTGSGKSKGVRS---VYPKPLNKWWDGYDDHTHVLLDDVDH--NQSSIGNLKIWSDHYP--FIAEKKGGSR-LIRPELII
VTSQYSIRELFNDNEL-------VLALERRFQVINVN
