# Supplementary material for: Viral metagenomics revealed diverse CRESS-DNA virus genomes in faeces of forest musk deer
Source: Virol J. 2020 Apr 25;17:61. doi: 10.1186/s12985-020-01332-y (PMC7183601; doi:10.1186/s12985-020-01332-y)
Supplement: Supplementary file 4 — Additional file 4. [file 12985_2020_1332_MOESM4_ESM.pdf]

>Smaco_AEW47007
YVATISRTSIPEHHLVRLLK-LDLHEAYIGRETG-ARGFEHYQCCIDC-----
LVRFNTEHQLGWHIEECS-WEAS-NYC
RKTDNYRYVGDSIEEREYS-IATRN----VARIQFHIDHRNDRAISICVDTIG-
TGKSTYGYLCARRTAT-------YIA
MHYDNQPVIWIDLPRS--------LAECLEDIKDGLVASAKYEGCLRFIRGVKVLVTTNH---
KTTYKML-ADRWDIFT
>Smaco_AIY31243
CGTIWKHLTWTMEQL-
AEWFKQHAKEGVIGLEISPTSGKEHYQFKIHLDRGETLEGWKALIGPMGHIEIAV-DKNF-
GYE
EKDGNFIKWPTSP-LEKYK-LKLR----WQ-DVVETFERQDDRRILVVVDKQG-
NGKSYLSRFMEATVIS--------EY
CLDNPC--YVFDLPR--------AMWMGIEQIKNGLLYEKRYRPRKMWIEPPKVLVFTND---
DVPWDML-RDRWEAYE
>Smaco_AIY31256
CGTIWKDKYGSKEQL-
AEWFRLHAKEGVIGEEISPTSGKIHYQFRIHLDRGETLKGWQELIKAVGHVEVCQ-EKKF-
DYE
KKDGNWIEWPESY-LGKFK-MPLL----WQ-QLLEQWNEQDDRQILFVKDVKG-
NGKSTFGKIMEARVCS--------DF
CLEYPA--YIFDIPR--------ALWSGIEQIKNGLLYEKRYKPRKVWIEPPKVIVFTND---
EPPWDLL-LDRWRVFD
>Smaco_KM598409
YIGTISAEDWNEDGIIRVFE-NDGHELYIGREIG-KHGFRHYQFCMDC-----
LEKYTADNRTGWHVERCS-WEMS-GYC
RKTGDYRYIGDSREERYYA---LRAR--LIWSFGASVVKQNDRSITVWVDTEK-
AGKSTFSYILERRTEN-------FVA
MHYKGEPLIIVDIPRD--------LCRALETIKDGVITSAKYQGTKMFIKGVKILVFTNH---
KTTYAAL-EDRWDVKS
>Smaco_KT862224
MD------HGTTQRL---AYQ-
HAKEGVIGEEISPTSGKTHYQCKWHLSRGESIDGWKLLIGPMGHVDIAV-EKRF-GYE
EKDGKFVKWPESP-IAKHK-LALK----WE-ALLDSIKNQDDRHITVVVDKQG-
NGKSTFSKYLEANVVS--------EY
CMEFPK--YVFDLPR--------AMWSGIEQIKNGLLYEKRYKPRKMWIEPPSILVFTND---
DIPWELL-EDRWDAYR
>Smaco_QBP37051
YMATIPHSGITKRAFFKMIRDYDIHKWTYAVERG-RGGYKHIQCRFRTNKS---
FEEIRKALICGHIEEAS---DNWEYE
KKDGNYMTSEDNHEILKLRYGKLTKV---
QEWALVLLESTNDREVVVWVDKDGNSGKTWLTAHLWERLST-PKELISWVH
SAYNHEPYIIIDIPRTWKWDD--ALYTAIETIKDGLVYDPRYSAHMRNIRGVKVMCMTNT---
EPKLSKLSEDRWVMYR

>Smaco_QBP37113
YMATIPHSGITKRAFFKMIRDYDIHKWTYAVERG-RGGYKHIQCRFRTNKS---
FEEIRRALICGHIEEAS---DNWEYE
KKDGNYMTSEDNHEILKLRYGKLTKV---
QEWALVLLESTNDREVVVWVDKDGNSGKTWLTAHLWERLST-PKELISWVH
SAYNHEPYIIIDIPRTWKWDD--ALYTAIETIKDGLVYDPRYSAHMRNIRGVKVMCMTNT---
EPKLSKLSEDRWVMYR
>smaco_YP_009252318
MERALPDEHGTTQRL---
AYQPHAKEGVIGEEISPTSGKTHYQCKWHLSRGESIDGWKLLIGPMGHVDIAV-
EKRFTGYE
EKDGKFVKWPESP-IAKHKNLALKT---
WEVALLDSIKNQDDRHITVVVDKQGGNGKSTFSKYLEANVVSDEYN-DYTSY
CMEFPKKAYVFDLPRATSIKRRTAMWSGIEQIKNGLLYEKRYKPRKMWIEPPSILVFTND---
DIPWELLSEDRWDAYR
>Smaco_YP_009022025
YIMTVPR-SVPKKA-LKIMIDVDCKKWIIGKERG-KNGYEHWQIRIETSNDE-
FFKWCKHHIPAAHIEEAQQGVDECLYE
RKEGQFWTSSDRVETLHQR-GTLR-----RQRALLALQSTNDREVMVWYD-
ANNVGKSWFCGALWERT-------VTWVA
SCYMDRPYVIIDIPR--------SLYCAIESIKDGLIYDTRYHARMINIRGVKVLVLTNT---
LPKLDKLR-DRWCIFE
>Smaco_YP_009030025
YMMTIPR-TVSKRA-LRIMIEKDCKKWIIGKEKG-KNGYEHWQIRIETSNDN-
FFQWIQDHIPTAHVEKSDNGVDECRYE
TKEGQYVTYSDRVQNLIQR-GAFR-----NQRAIQALEATNDRQVVVWYD-
ETNVGKSWFTGALWERT-------ITWVA
SCYIDRPYVIIDVPR--------SLYSAIESIKDGLIYDTRYHSRMINIRGVKVLVMTNT---
MPKLDKLK-DRWCICT
>Smaco_YP_009054985
YMMTIPR-TVSKRA-LRIMIDVDCKKWIIGKEEG-KNGYKHWQIRIETSNDS-
FFEWMQDHIPTAHIERTECGVDACRYE
AKEGQYVMYSDRPQNLMQR-GEFR-----NQHALQALQQSNDREVVVWYD-
ETNVGKSWFTGALWERT-------ITWIA
SCYIERPYIIIDIPR--------SLYSAIESIKDGLIYDTRYHSSMMNIRGVKVLVMTNT---
MPKLDKLK-DRWCIRT
>Smaco_YP_009252314
YSVTISADLWKERDVVRLLD-MDLREYYIGREIG-KGGYHHYQCAIDC-----
LERFNGQHQLGWHIEDCS-WDKL-RYC
RKGGDYRYIGDSIEEQSYR---SRT----VGTIDTHLKKQNDRQISICVDTKG-
SGKTTHGYDRSRTNAT-------YVA
MNYDNEPVIWIDLPRT--------LATILEDMKDGLIYSAKYEGQVRHIKGVKVLVTTNH---
KPAYKLL-ADRWDVFT
>Smaco_YP_009508826

YVATISRTSIPEHHLVRLLKLLDLHEAYIGRETG-ARGFEHYQCCIDCAGD--
LVRFNTEHQLGWHIEECVSWEASKNYC
RKTDNYRYVGDSIEEREYSRIATRPKNIVADRIQFHIDHRNDRAISICVDTIGGTGKSTYGY
LCARRTAETPTRIMDYIA
MHYDNQPVIWIDLPRSRLVDK--
DLAECLEDIKDGLVASAKYEGCLRFIRGVKVLVTTNHWVDKTTYKMLSADRWDIFT
>UJSL002_MN621468
LTIWKDRYKGTKEELKAWLIAHHAKEGVIGEEISPTSGKMHYQCKIHLSRGETLEGWKAL
IGPFGHVDIAH-DKNFSGYE
EKDGNFIRWPESP-IEKFKDIGPLYF--
WEQVVLAELEKQDDRKMLVVLDTTGKGGKTTFSKHLEAKVVSDEYN-DYTGY
CMEFPAKGYIFDIPRASSIKRRCAMWSGIEKIKDGLLYEKRYKPRKKWIEPPKVLIFTNEP--
DIPYEMLSRDRWVVVD
>UJSL004_MN621481
YCATISRESIKEHQLVRLLNLLDLHEAYIGRETG-NRGFQHYQCCIDCAGD--
LERFNREHNLGWHVETCISWECSKNYC
RKTGNYRYVGDSIEEREFNRISRRPQNIVGKRICEHLRSQGNRKISICVDTDGGTGKSTNG
YIHVRNTAETPVRIMDYIA
MKYNNEPVIWIDLPRTRSVDN--
DLAECLEDIKDGLIASAKYEGNLKLIRGVKVLVTTNHWIKKETYKLLSQDRWDVFT
>UJSL007_MN621470
YMATIPHSGITKRAFFQMIRDFDIHKWTYAVERG-RGGYKHIQCRFRTNKS---
FDEIRHALICGHIEEAS---DTWEYE
KKDGNYMTSEDNHEILKLRYGKPTKV---
QEWALRLLESTNDREVVVWVDKEGNSGKTWLTAHLWERLST-PKELISWVH
SAYNHEPYIIIDIPRTWKWDD--ALYTAIETIKDGLVYDPRYSAHMRNIRGVKVMCMTNT---
EPKLSKLSEDRWVMYR
>Smaco_KY086298
YMLTIPR-KVHKRT-LKIMLEQDVKKYIIAKERG-FGGYEHWQIRLKTSNKN-
FFIWCKINIPEAHVEEAM---DTWDYE
RKEGVYWTSDDTNEIRALR-GKPN-----KQRVLKLLKYQGDRNILVWYD-
PVKAGKSWIVGHLWEQT-------LTWVH
SAYDNEGLIIIDIPR--------SLYTAIETIKDGLVYDPRYSARMKNIRGVKVLVMTNT---
YPRVSALE-DRWDIIN
>Smaco_KP233189
YMLTIPRDKVSKRE-LRIMLDKDCKKWIIGKETG-KNGYKHWQIRLETSNEE-
FFDWCKKHIPTASIRKAE--VPKWDYE
AKEGQYWTSSDRTDNLIQR-GEFR-----NQRAIQALRATNDREVLVWYD-
EGNVGKSWFTGALWERT-------VTFVA
S-----PYVFIDIPR--------SLYCAIESIKDGLVYDSRYQGRMVNIRGVKIIVMTNN---
KPDLDKLY-DRWRMVV
