# Supplementary material for: Viral metagenomics revealed diverse CRESS-DNA virus genomes in faeces of forest musk deer
Source: Virol J. 2020 Apr 25;17:61. doi: 10.1186/s12985-020-01332-y (PMC7183601; doi:10.1186/s12985-020-01332-y)
Supplement: Supplementary file 5 — Additional file 5. [file 12985_2020_1332_MOESM5_ESM.pdf]

>pCRESS4_WP_000186194
VRRVKIIQYENNPTESLNFDENILNALAYFSHVITEGKPKGTHYHVVMELTNPASISA-IAKRFGVPAQYVEVIEGAV-L
DCIAYLTHEDAKQVDNQKA---------REALTKRADARVIIEKISQGMTLSQVYEFDNVMAVENKNLFKTARQEYLKNA
PVPPVRTN-YYVYGEGGTGKSLSAKVLARSFVVGDGAVPFDGYDGQPIIIWDDWRALHFDRSLVWKLFAINPERISVNVK
YGSTSLINAVNIVTCVDPYLKFMEELEYTDKRKKEDSRQAFRRFPFF
>pCRESS4_WP_000818357
ARVYSVVQYEFNPTEDLHFNEVIKNAILNKETKYVEGDVRPPHWHVLLKFKNQIEFST-IAKLFNVPENLVEKKTGAF-F
DYLYYMTHEDDKQVDIRED---------RRAK-KLSKVEVFLDKLTSGMTMKQVFERDSVVFAENATLFRRARRSYLKYA
PTPLVRTN-YHISGAGGTGKTLIAKSMARAFVVGDGRVAFDEYDGQPIIIWDDFRAKAFERGTMWKIFAIHPDKVSVHVK
NGETTLINTVNIITSVEPFTEFVNGLAFKDN-KSEDVGQAYRRFPIF
>pCRESS4_WP_044572803
GRCFEIVQDEKHPTAIL-LTRRIEKVLAKRPNQYTDGEYKVPHFHIAEKRKNEASVGQ-VARAYDVAPQYVRVKP-AF-L
DLVDYQTHGLERQRVAKRI---------NSPRKQTPIDKLAMRIQEDGLTLRLAKEEDPLSFNRAPGRMEKSRATYLRHL
PPPSSRIN-FYFEGEGGVGKDLLAKALARTFSVGGENVGLDGYDGQPVIIFEEARAGSMGRKELFAFMNPFPEKQSLNVK
YGATQPVNTITIFTGPDDYDTFLDGLEFIDKSKAENKPQARRRIPII
>pCRESS5_WP_024393234
FFGQRNYDFEHSDSKDDWKEKIKKELFEIDIKESN--ELKALHCHFVIRFNNPRSYSN-ILELTKCEERNFERSTNE--G
AILRYLTHTTPEAIGKKEK----VVDFVNDLAYRLSVGEFKPINAIAEFGQSIYRKEKKKFQED----YADFLETKKKDL
LLNGKELSTIYIDGFSEVGKSTFAQDLANAYLAAKKKDWISKYKDEYITIFNDVDPYDFNFTYFLGTFE-TKILVDVGSR
YKDKTWFSEYAIITKSTDIHEFVNKIELREDNHFNIRYQVQRRFSLI
>pCRESS5_WP_050238550
FFGQRNYEYEHTETESEWKQRILEELFSISIKEHN--ELKGLHCHFVIRFDNPRSYDS-ILELTSCQERNFQRSTNQ--G
AILRYLTHTTPEALGRKEK----VTDFVADLAYRLSIGEFKPHTAIAEFGQSIFRKEKKKFQED----YNDFLDSKKRDM
LLNGKELSTIYIEGPSEVGKSVFAQDLANSYLASKHNDWISKYKDEFVTIFNDLDANLFSFTDFLGTFE-QKILVDVSSR
YKDKTWFSEYAIITKSSDIDEFVNKLEIREDNHQNIRYQVQRRINLI
>pCRESS5_WP_061866456
FSISRNKQYEHQESDTDWKEKVKSETFYLATELTSDGERKGLHCHMILEFRNPVTITSFKFEAGKFQSRNVEASKSS--S
GSYRYLTHTTDKAIRPEK------DEALQEAFFKVRTGEIFDEEVRERFTTELVIKNKKFIDNS----RQMYQKEVFEDM
QNNGRNLKTFFISGSSGLGKSRFAKDLARRYTAPTAKDFISEYKAQDVTIFDDVDAKSFGFQEFLNIFD-KDNITKISSR
YTNKAWVSHYAIITKASKIRNWIERVEYEKD----KEVQVSRRFDLW
>pCRESS6_WP_017371219
KTFFGMQYLEKEYWETRNQKEIFALVCEKFRQINENGKLEAPHLHWAIHLKERTTLNK-IAKAFQVEPQYIETGNQGAMI
GRLAYLTHQTEKMKFKKLLA-TRAKYELNHLLQQVQVGKLFIEDILSDEKYFVYANNLAKFKEAFEAYAQRNSLLTIQDR
VAQKFEFTSIYIYGKSGSGKSEIAYDILKQYFGGS-KNAVDDYKGEELLLFDDVRPETFSPADWTKILD-YKNKSALSGR
FHNRPLSNRLVLMTNTQSPFEFFK--ENEPIE------QYLRRLTYV
>pCRESS6_WP_039670385
TTFIFEQQLKGEYWKAKNKQEIFQEIYNRVKKTFN--NLVFPHLHGYIEFSNKRDLSV-LALNLGLYPQYIEPSGRGGKI
NSKAYLIHAKSLDSFTKQSA-KEQDESLDMIFQEIIKGKLTEDDIFADEKTFLWAYNQQKFDEAFKAYGKISAKTTLRQL
ENGEFKPTILYIHGQSGIGKTSLAYDLVAEYNAGS-KNIFDEYFGEEIIVLDDPRYDSLLPSDWLKLLD-PLNKSYLSAR
YRNKLVIGRVIVITNYMSLSEFFRQIPKEDIN------QYLRRFNNV
>pCRESS6_WP_061343647
KSFYGTQQLEQEFWDNRNQRRIFEIIATRFLRKDDTGNFIKPHVHWLLELKNKRDLDE-IAYKFFVHPQQIEKGSKGCFL
GRIGYLTHQAESAYFKKRLA-FQQKMDVDYYLQQVQQGILFLDDIFLDLNYNVYANNKQKFREAFDAYSELNSFRTNRDK
RLGIFDFTTIFIYGRSGLGKTTIAMAILDRYSGSA-KNAVDDYKAEELILFDDLKQDSFLIADWLKILD-SRNESTISGR
FHNKPLSARLIILTTIESPFKYFDFGKDEPKE------QFIRRLSYI
>pCRESS7_CDE19587
---EVVTQPEYLKSPLQDILRKYRTIKQWAYILHDKDKDASSHYHIYINFQQTVDSKD-VAGWFGIPEQFVNKVEGRK-T
DMLMYLTHSNDSYDFSEVVAN-------FDFKSEIEQAKIIGDFEKYSYIHSLAVSEQPKCFDR---LQKLWKLQCQWLS
LNSDRNLKVIFVTGKSGTGKTYFARKYMRAYVSSSSNDPLDMYMGQKGIIFDDLRDEAFEFADILKLLD-NNTSTAMKSR

FTNKVLNCKVMIITSFIPIKYWYKSVRYSCDG----IEQLYRRINMY
>pCRESS7_WP_002578150
-RVICEFQNQIEYTSESSFKNIVNNLYKRG--TYTEGNLKKTHIHGMLRLNNSYKFST-IANWFDVTAQRIRKIETSY-A
AACAYLIHRNNQLTNENHMK-----KLKKRILEEVEAGTLRGYNFHENYAFSDRVALRSYLNNAIEIIKTKLNS------
-NKERDLEVIYIHGSSGAGKT--TYAKMTAATSGEDRDPVETYDSHPCMILDELRPSSMKLTSFLKLVDNNTESMAGARY
HGKAFIECKLIIITSILPIEEFFKNLQANDNE---TAIQIKRRCKTM
>pCRESS7_WP_028509833
TCEITQDCCGWTPENVHKFIDGWSSVKDYAYKKDD-TTPREPHIHLMLRFSCAVHTSN-ILARAKITENRIQKMKSWS--
AALNYLTHRDEHKWKHVYHQ------AKQLRADKGREKEIVEAIASGEIRLFNLSEHITSYEENLYAIKTAFNRRTRDLK
LKNERNMEVIFISGESGVGKDTFAREWCKYFTTGNNDSPFDDYMGQDVIIWSDARDDVYKPAQIHTMLD-NHWSSTQKAR
FVDQVLNCQYFIITSVKPLNEWYKNFYSKEGE---DIKQLYRRIKTW
>pCRESS8_YP_006939186
FMYTQQLKYLNSIEQLKNNLENDAYIQDFAMILDENNQNVAEHLHVFIKLNQQKTIDY-VADLVDDKAQYIENKSRNE-Q
NGYLYLLHKTKSAEHKHYENNSKRKTVVQSILNDYADRIIDEKELKDSLTNLELAKNKKLINDIKQVLIEFDFQTYLEQ-
-ERYKNKQVVWIFGKSSTGKSMMSQLLAKDYVTSSNRDPFEDYQNQKVLIIEEFRNENIGTNELLQLLDKTNGQVRVGSR
YSNKKIMADLIIINTIYEPKYFMFDEPIYQLLRRIDKLVKLDNQKIE
>pCRESS8_WP_062359070
---YRTFGYTQQLSHLNR-LEDFPRLMEERTADGV---LKASHVHVVMKFENPRSLQA-VSKIFHDSPQYVEKTRNGY-N
NMLAYLTHRTKGATAKVEQR---DSKTLDELLERLMDYSITYEEAVGELTASEFSRYHQRLKVTAEYGRQQYAKRWIKEH
EDNHKPIEVIWITGPAGSGKTVEAKKIAREYVAVGVRDPFQLYTDENVIILDDLRAKTFDYNTLLQLLDPFNST-MADAR
YSNKTLIADTIIITSVYSPLDFYHMLVPEIYD-VDTYKQLERRIS--
>pCRESS8_WP_016356676
---YRNFMYENQLKYMKEDINMLAKYVEETINESE---LVAPHYHIALKFENPRNVNS-VAKKFNDLPQNFEIWLNRP-N
NLYSYLIHKTSDATKSIHRG---RNEEIRHLIDSFGNGEITLKLLMEELSPTEYARNENQINIIKKLLANKRFEAFKKRM
DSEEKRIEVFYLFGGTGTGKTRFAKTRY-KYITGSNRDLFANYDGETVVILDELRPNSISYNELLKITDPFNFENVVGSR
YLDKKLVAETIVITTPFSPEEFYQTLKGEQTN-IDKKEQLFRRINV-
>UJSL005_MN604398
LYNTSQATVEEWFAKNKPKIQSMAACLDVGGKFLKDSFGPECHMEVAKRYLQAYIYVLEHAANKKVFIEYRTPKAGRSTT
KKVQSDVFIRETLSRNMVRP-------IPRMVVWINGHTGTGKSLLANKFMDLHQPSSAYMCSPSGSGPDNDPRSVLLDD
FNIALTPLTTLFNMTDQYNKTIDCKGSYMIYSPYNVVGMEQTYTTPHRKITDHSRRINLYIKTASPVPNNVDGYRVMHPD
GTPDELIPKMNLDEMIAYIDSIYKSWEQKYYEEQLNNEENYHEVENN
