# Supplementary material for: Viral metagenomics revealed diverse CRESS-DNA virus genomes in faeces of forest musk deer
Source: Virol J. 2020 Apr 25;17:61. doi: 10.1186/s12985-020-01332-y (PMC7183601; doi:10.1186/s12985-020-01332-y)
Supplement: Supplementary file 6 — Additional file 6. [file 12985_2020_1332_MOESM6_ESM.pdf]

>UJSL006_MN621480
KGDAVKTWFCTWPKCTIPKEFALKLLEKHGL--KEKHEDGSPHLHAYLKLIKRKKFNEK--MFDLIDVDK-
KVFHGNYQK
VKDVLAVEKYVTKG----------QHKSKLLKEDYLKDPLELLEEGKINFFQINNFLKNQDCYKMLLNRKQNRPLKPLEK
--------RRHHWIFGESNTGKTERLWKAMDTEGGWFQIPTNNDWKGYNGEINLYMDEYKGQLTIQELNRICDG--
GAKV
NTKGGSTQLSWEVTVWICSNYSIDNCYNKAEKMLLMSLWNRFNEEEML-
>pCRESS9_KXT29014
YRLKTKMVGLTYSRFPVKEKFIQLLKELFNSKCNDYHMDSGLHIHALLVFDKKIQLRDAQKVFALPN--------
YQTAK
FGHLDRFRAYIIKKGNFIENLKSGLIVAEKQLSFGYKLDNVLYWDKLRDKMSKTFVFELIYDFDSLFEPFNMVEKM
VLKL
MNS--
GVRKKSLFIVGGTGIGKTRMIKTILKYSYIKGKIDFSPKKFDDSRPVVIMDDITLQYLPEDGFKNFIGNGDTTEV
DVKMQKTATITGGKLFIYIVNKHP--EKWVK--NDEYDHIYIRKNIEVI
>pCRESS9_KXT29032
YRMHSRNIGFTYPNLSLSKEEVQKIFIQKESRSRELHEDGEPHIHILIQLNKKTEFCNAREFFALPTFNKPEHWYRYIG
A
YGDVLDDGIFKLLISAFEAEKALNQYLQELDVVIYYKQFPIRDRVIQENFYPKSPVVKREHSLQTFRLYHEKVQLIIK
EQ
FNS---
KSPLTIVLEGLTQIGKTDLAELIQPYNYTKIDFNFSRENYNDSYKICIYDDMGMEEVSKKLMHALIAGRGSFQT
REPYGKKRTISGNKLNIFIVNRNKSFKGWIEKNKEWKRFEYVEPNVIII
>pCRESS9_CBX25033
FRLRTKDFFMTYSQCDLGKEKIFNHLKQLMA-SENHADNNGVHSHVFLQLKKYLDIQSG-RFFDIDG------KHPEIER
ARTVQGSVDYVKKDGDFIIKKSLNDFILKTDRDFYYEKIDLIKKILNERFTKKKELEDEDYSFNSFKSNSKTNEIIQT
QL
SVS-LGKRPKSIVIEGPSRLGKTEFILSYTHYNYTRGEFNFSKQSHKNAYKINIFDDISLTKIKEGLIKDIIGGQKGFTV
DVKYAPKRNISGKKLSIFLVNPDISFENYCEWSRNNGHKFYIEDNCIF-
>pCRESS9_WP_011412958
FQKNAKDIFLTYSKCPLGKDKIHNYIKEIMI-SENHQDHKEIHTHVLFQLTKRFNIQSD-RFFDIEG------FHPRIET
ARNIEKSISYIKKDGDFIVKKSLNDFIKILDRDFYYEKIDFVEQVLNKKFTKKKELEDEDYSFDSFKSNSKTNEIINQQ
L
SVS-LGKRPKSIVIEGPSRLGKTEFILSYTHYNYTRGEFDFSKQNHKNAYKINIFDDISLTRIKEGLIKDIIGGQKGFSY
NVKYAPKRTIAGKKLSIFLVNPDISFENYCEWSRNKGYKYYLEDNCIF-
>pCRESS9_WP_012662291
-MFQAQNIFLTYSQCDLSKEEIKNFIINLCN-EENHQDHKGKHHHVFFQLNKRFKTRDL-NIFNIPKNI----YSPHIEP
IKDTTDVRNYVKKDGDFIIFKKLKLYAESLEPNYAFKN--------TKRFKNMAYIYESIFNFCTFKKIPILICIYKTQK
EQSVISQRFKTLIVEGNSKSGKTQFFKSVTPFNYIKDDVDFSDENYDEDKYVNIYDDIDIYDIARNLTKVVIGNQKDS
IV
NMKYKPRN--
>pCRESS9_WP_013747472
FRLQTKDIFLTYSKCPLGKDKIHNYIKELMI-SENHQDHKEIHTHVLFQLTKQLSIRNQ-RFFDIEG------FHPKIEN
ARDIEKSIDYIKKDGDFIVRKSLDDFILNIDRDFYLEQSELIDRILNKKFTKKKELEDEDYSFDSFKSNSKTNEIIQTQL
SVS-LGERPKSIVIEGFSRLGKTEFILSYTHYNYTRGDFDFSKQSHKNAYKVNIFDDISIPQIKEGLFKQIIGGQKGFKY
NVKYAPKRTIAGKKLSIFLVNPDISFENYCEWSEDNGHKFYIKDNCIF-
>pCRESS9_WP_042068233
-IFKAQNIFLTYSQCDLSKEEIKTFIINICD-EENHQDHKGKHHHVFFQLNKRLQTRDL-TIFNIPKNN----YSPHIEP
IKDTTDVRNYVKKDGDFIIFKKLKLYAESLEPNYAFKN--------AKRFKNMVFIFESIFDFCTFKKIPILTSTYETQK

EQSSISKRFKTLIVEGNSKSGKTQFFKSVTPFNYIKDDVDFSDENYDEDKYVNIYDDIDIYDIARNLTKVVIGNQKDS
IV
NMKYKPRTKIKGSDISIMLVNEDTSIEKYCFDNFKRGRKEYIRENAIF-
>pCRESS9_YP_001965305
FRLRTRDIFLTYSKCPLGKEKIHNHLKQLLA-SENHQDHKEIHTHVFIQLKKQIEITNQ-RFFDIEG------YHPKIET
ARDVEKSVSYIKKDKDFIIRKEIDDFILKIDRDFYYEQIELIDRILNRRFIRKKELADTHYQFNSFKTNSETNEIINSQL
----LSHRPKSIVIEGESRMGKTQFILSYTQYNYIKGEFDFSKKTYKDYYKIDVYDDFGVPEISQGLQKNIIGGQECFTC
NVKYAPKRQLSGNKLSIFLVNPDNSFKGYCEWSRNNGHKFYIEENCIF-
>pCRESS9_YP_001965310
FRLQTKDIFLTYSKCPLGKEKIHNHIKQLME-SENHQDHKEIHTHVLFQLNKRCNLTSQ-RFFDLDG------YHPKIEN
TRDVEKAIEYIKKDGDFVVKKTLDDFIINLDRDFYLEQIEFIKRVLKEAFAKKEELADDDYSFESFKTNSTTNEIIQSQ
L
SVS-LSKRPKSIVIEGPSRIGKTEFLLSYTHYNYIRGEFDFSKESHKNAYKINIFDDISIPQIKEGLFKNIIGGQRGFRF
NVKYAPKRFIAGKKINIFLINPDISFKGYCEWSYKKGHKFYIEDNCIF-
>pCRESS9_YP_006959585
-MFQAQNIFLTYSQCDLSKEEIKTFIINLCN-EENHQDHKGKHHHVFFQLNKQFRTRDL-TIFNIPKNI----YSPHIEP
IKDTTDVRNYVKKDGDFIIFKKLKLYAESLEPNYAFKN--------AKRFKNMVFIFESIFDFCTFKKIPILISIYETQK
EQSSISKRFKTLIVEGNSKSGKTQFFKSVTPFNYIKDDVDFSDENYDEDKCVNIYDDIDIYDIARNLTKVVIGNQKDS
IV
NMKYKPRTKIKGTDISIMLVNEDTSIEKYCFDNFKRGRKEYIRENAIF-
>pCRESS9_YP_007008175
FKLNTKDIFLTYSKCPLGKDKIHNHIKQLII-SENHQDHKEIHTHVLFQLTKRTTFHGE-RFFDIEG------FHPKIET
ARDIEKSIDYIKKDGDFIVKKTLDDFIKNLDRDFYYEQIDLIEKILKKKFIKQAELIDKGYDLNTFKVDKSTQEIGTNQ
L
KV----KRPLSIVIEGPSRLGKTEFIISYNHFNYIRGSFDFSKENYNDSFKVDVYDDISMNYISSGLLKNIIGGQRGFIV
DVKYPPKRLLSGNKLSIFLVNPDISFESYCEEDEKHAGETYIKSNCIF-
