# Supplementary material for: Viral metagenomics revealed diverse CRESS-DNA virus genomes in faeces of forest musk deer
Source: Virol J. 2020 Apr 25;17:61. doi: 10.1186/s12985-020-01332-y (PMC7183601; doi:10.1186/s12985-020-01332-y)
Supplement: Supplementary file 7 — Additional file 7. [file 12985_2020_1332_MOESM7_ESM.pdf]

>CRESSV1_KF133822
--LRNVCFTLYNADPEDVASK-----LSELEEKCPESDRLHLQGYLECTKPVRFGTLKDFLGS--TVHLERRRGNRQQAR
DYCRKEET-KQGQRTD-LDAIREMVVAGATEESIADEYFGTWARNHRAIARYKFLKSKPRD-----FKPRVIIRWGVAGS
GKTRGVYDTHEPR-PNGGTVWFDGYDPHEVVLLDDFYG-WLPWSMLLQMLDRYPMSVPKKGSTCNFR-
AKFIYITSNADP
ETWYD-YSKPGIEFEALKRRVDETHHF
>CRESSV1_KF246569
-QVKMWALTVKNWDLFDLEE--
IGNYQDGQKHTGEKTGYQHCHLNLELEKRQYMTWIKRVLFNIPDMHCEKRQGTREQCD
TYLAKDGEFERGHRSD-
LDEIHDMIKEGKDLFDVYESHFASTVRYSSGLEKYIVLHDSKRARNSETTAPQVIVYVGPAGS
GKSWHCFNDEDYPIQMYEKVYFDGYNREKTIWFDEFNGRSMPFGKFCQLADRFPGIYETKGGSVLISGLKKILISTI
SYP
ATWWG-SNRFNLDPDQLYRRLTKCYYL
>CRESSV1_KJ206566
-MVKYRKVFVTSFNTSAMEQ-----DLVDCMEHAPTTGRIHYHLYIEFYSQKTMNTIKKILKD-ATANIQPARGTPQEAV
DYIKKDGATNQGHRSD-
LDDVFQRLQVGDNILDIIEGHPGTCIRYIKGILAVKGLVDQRRQRQEARQMPTVLVYIGKSGA
GKSHACSQDPDYPVQGPSKVYFDGYTGESTIWFDEFGGSVLPFHVFLRLADKYETRVETKGGSVCITGLQKILISTT
TPP
KLWWE-SRKFNEDPYQLWRRLTRVYYI
>CRESSV1_KM573766
MPARYSNWILTTWDPYLKEN--WWEMDNHEHCHEEQR--EHWHIYFELNHRKNMASIK-
EWLNDLTVHCEVRRGTGQQAI
KYCQKGFDFQ-GSRSD-
LVNCKQLLEEGVSMIELAQAHFGDFVRYYRGLYQYADLLSREKQRTAGLSEVEVTVFIGPAGS
GKTYNCSKKAEYMQQQNGKCYFDGYEKQKCIWFDEFTGSTMQFNHWCRLADKYGVRVETKGGSVQISGLKRIII
STIIPP
GEWWN-SQSFRDDPEQLWRRITEIYYC
>CRESSV1_KM874309
LMRRNVVGTLTNERPHEITS----------YEICPSSKRRHLQFYCEFVGQQRLGAVRQLFGP--TVHVEPRRGTVDEAR
TYCSKEDTPKSGKRSD-LADIKEELDAGSSLKSISQQHFGQFLRYRKSFEAYIVLNQDPRT-----WEMENSILWGEPGT
GKTKLAYDLKEMR-NQNGNVWFDGYHGQEILLIDDYYG-WIPLAFLLQLLDRYPMNVQTKGGSVPFT-
SKKIIITSNKSP
ECWYN-WSKFGKNFGAFERRINQVFHY
>CRESSV1_KP153497
-MSRGKSWCFTIHSRGEGD--CDWLHPIG-MERCPETGKLHLQGALQLDKQQRLSFMKKLHKT---
AHWEVMKGNWTQSI
EYCSKSEK--MGQRTD-LETIGKMVKENKTNLELVDTLGAGVSKFQKHIS--FTYSEKDSDRQA--
TGVKVIVLYGPTGT
GKTYAAVNS---NSQKATKLWFDGYENQKVLVIDDFDG-SVEFRYLLRMIDVYKFPAEVKGG-
MVWGVWDTVIITSNVHP
ASWYT-------DTSPLKRRIAEIRLC
>CRESSV1_KT149404
-MS-SRRWVYTLFAAAEDD--YSNLDDFERLECCPTTSKVHLQGYVEFEKPQRMAALKKLNST---
VHLETAKGTREHCV
RYCTKDETT-QGRRNDLLETTLAIVNGELSRDDVFDTRPDLICKYARGINELLTYRAK-KERQG--
DELHTEVLWGDAGV
GKTRYAYG-----RCKGGALWWDGYDGQSILVIDDFYG-WVEHSVLLRILDRYPFKIDIKGS-

STYANWKEVYITSNRHP
STWYTRAPWT--EDKALQRRLGAIYEC
>CRESSV1_KT862256
-QAKVWTMTVKNWDLFDEER--
IQNQTEGSKHTGQQTGYQHCHFNLELEKKKTMAWIKRELFNREDIHCEPRRGSREQCD
SYLNKDGEFKPGRRTD-
LDDIHDMIKEGASLYDCYEEHFGTVVRCERGLRDYIALRDTILATKKKYPAPEVIVYVGPSGS
GKSWHCSEDPDYSIQMDSKIYFDGYNNQKTLWFDEFSGKTMPFTKFCQIADRYPGRYETKGGSVLIYGLKKILISTV
EYP
ALWWG-SDRYNKDPEQLFRRITKCYYL
>CRESSV1_KU043411
TMNRMRCATVTSFNIDYIKR-----DVINNLEVCPETGKLHYQMYMEFEEKVTVKQIKRLLRD-
NGAHVEPRYGTAKEAI
EYCKKDGNQ-QGERTD-
LADVYESLKVGRSLLDIIEEHPGTYIRYFRGIERVQDLFRRKQQKLEERVQPTVLVYIGKSGT
GKSHHCYHDPDYPVQQAGKVYFDGYDGESTIWFDEFGGSVLPFGVFLRLCDKWETRVETKGASVCITNLRKILIST
TTYP
KNWWD-SRKYQEDPKQLWRRLTHVYYI
>CRESSV1_KU043424
RPKRFRFACITAWNMDAFDP-----
QRMHDRETCPRTGRHHLQCYFEAPNPKTVEQWQDVVCDKPRAHVECRHNEGDRAA
DYCKKDGDG-QGARMD-
LADCKTMIDEGKQMIDLYEKHFGTCVRCHRGLMLYKDLVDRKRRKEAEPEPKEVVVYVGASGS
GKSHHCWHDPDYPLLAENKVWFDGYEGEEVLWIDEFRGSVFPFGLFLQVTDKWGARVEVKGGSVETF-
FKKILISTTVPP
GEWYK-CPNFLSNPQQLWRRLTKVYWL
>CRESSV1_KX388513
RHKQLAEWT-TYG-----------DPTTMNLEYTQNQDNFHWQGYVQFHKSLRFTQVKKYLKSY-TAHVEQANGTLEENI
QYCSKSASE-SGERTD-IERLYVDVKSGKSMACIIDHHTNTWFKYHGAVDKYLEQKVACQKRTC--
TVTVWVDQGQTGKT
TRALYDINE---RTHEEGRLWWDGYDGQPTLVIDDCVH-LIKFDYWKSLVDGHPMRIQIKNG-
WKNALWNRVIINSNRHP
NNWWPDNSEANLSMPYFKGRIALINHV
>CRESSV1_KX388515
RPQQLRHWTFTIGGEEAQTITEDWDATTMNLEYTQNEDNFHWQGYVQFHKSLRFTQVKKYLKS--
TAHVEQAKGTLEENI
QYCSKSASE-SGERTD-IERLYVDVKSGKPMASIIDHHTNTWFKYHGAVDKYLEQKTACQKRNC--
KVTVWVDQGQTGKT
TRALYDIDE---RTHEEGRLWWDGYDGQPTLVIDDCVH-LIKFDYWKSLVDGHPMRIQIKNG-
WKNALWNRVIINSNRHP
NNWWPDNTEANLNMPYFKGRIALINHV
>UJSL017_MN621476
PTPRFRAVCFTSFVPEAMWDR-NWEQLLGHKEICPETGRLHIQGYVEFSGQKKMKQIKLIFGD-
PAIHLEPRRGTQEEAI
IYCKKEGQQ-QGQRTD-
LEDTYERIKVGQSLLDIAEAHPGTYIRYFKGIERLRDLMQQRAQKLEARVPPEVIVYIGKSGV
GKSHACYNDPDYPVQQTGKVYFDGYQGERVIWFDEFGGSVLPFHVFLRLADKWETRVETKGSSICILGLKKILIST
TTYP
KDWWPNSEKFREDPNQLWRRLTRVYYI
